# High-Throughput Phenotypic Screening and Machine Learning Methods Enabled the Selection of Broad-Spectrum Low-Toxicity Antitrypanosomatidic Agents

**DOI:** 10.1021/acs.jmedchem.3c01322

**Published:** 2023-11-03

**Authors:** Pasquale Linciano, Antonio Quotadamo, Rosaria Luciani, Matteo Santucci, Kimberley M. Zorn, Daniel H. Foil, Thomas R. Lane, Anabela Cordeiro da Silva, Nuno Santarem, Carolina B Moraes, Lucio Freitas-Junior, Ulrike Wittig, Wolfgang Mueller, Michele Tonelli, Stefania Ferrari, Alberto Venturelli, Sheraz Gul, Maria Kuzikov, Bernhard Ellinger, Jeanette Reinshagen, Sean Ekins, Maria Paola Costi

**Affiliations:** † Department of Life Sciences, University of Modena and Reggio Emilia, Via Campi 103, 41125 Modena, Italy; ‡ Collaborations Pharmaceuticals, Inc., 840 Main Campus Drive, Lab 3510, Raleigh, North Carolina 27606, United States; § Institute for Molecular and Cell Biology, 4150-180 Porto, Portugal; ∥ Instituto de Investigaçao e Inovaçao em Saúde, Universidade do Porto and Institute for Molecular and Cell Biology, 4150-180 Porto, Portugal; ⊥ Brazilian Biosciences National Laboratory (LNBio), Brazilian Center for Research in Energy and Materials (CNPEM), 13083-970 Campinas, São Paulo, Brazil; # Scientific Databases and Visualization Group and Molecular and Cellular Modelling Group, Heidelberg Institute for Theoretical Studies (HITS), D-69118 Heidelberg, Germany; ¶ Department of Pharmacy, University of Genoa, Viale Benedetto XV n.3, 16132 Genoa, Italy; ∇ TYDOCK PHARMA S.r.l., Strada Gherbella 294/b, 41126 Modena, Italy; ○ Fraunhofer Translational Medicine and Pharmacology, Schnackenburgallee 114, D-22525 Hamburg, Germany; ⧫ Fraunhofer Cluster of Excellence Immune-Mediated Diseases CIMD, Schnackenburgallee 114, D-22525 Hamburg, Germany

## Abstract

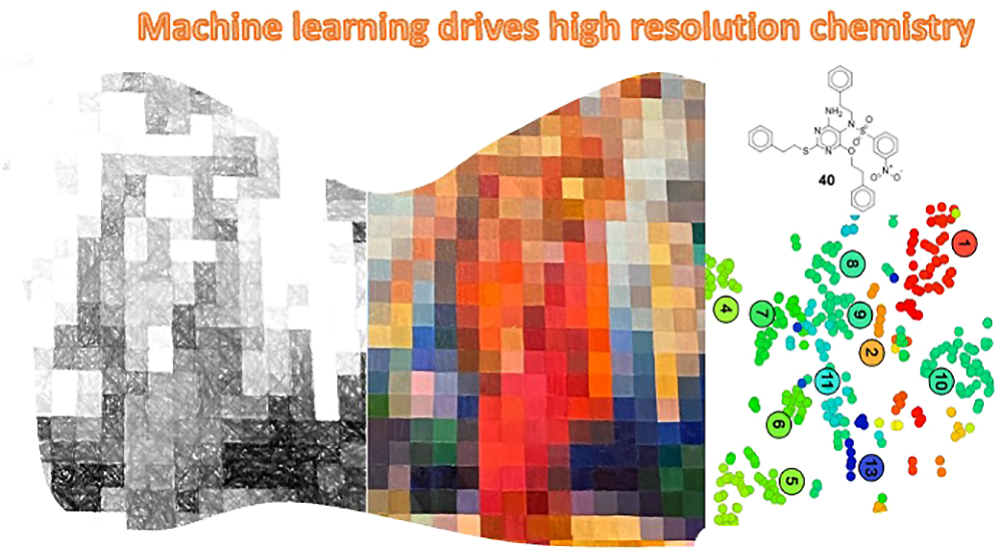

Broad-spectrum anti-infective chemotherapy agents with activity against *Trypanosomes*, *Leishmania,* and *Mycobacterium tuberculosis* species were identified from a high-throughput phenotypic screening program of the 456 compounds belonging to the Ty-Box, an in-house industry database. Compound characterization using machine learning approaches enabled the identification and synthesis of 44 compounds with broad-spectrum antiparasitic activity and minimal toxicity against *Trypanosoma brucei*, *Leishmania Infantum,* and *Trypanosoma cruzi*. In vitro studies confirmed the predictive models identified in compound **40** which emerged as a new lead, featured by an innovative *N*-(5-pyrimidinyl)benzenesulfonamide scaffold and promising low micromolar activity against two parasites and low toxicity. Given the volume and complexity of data generated by the diverse high-throughput screening assays performed on the compounds of the Ty-Box library, the chemoinformatic and machine learning tools enabled the selection of compounds eligible for further evaluation of their biological and toxicological activities and aided in the decision-making process toward the design and optimization of the identified lead.

## Introduction

**Figure 1:**
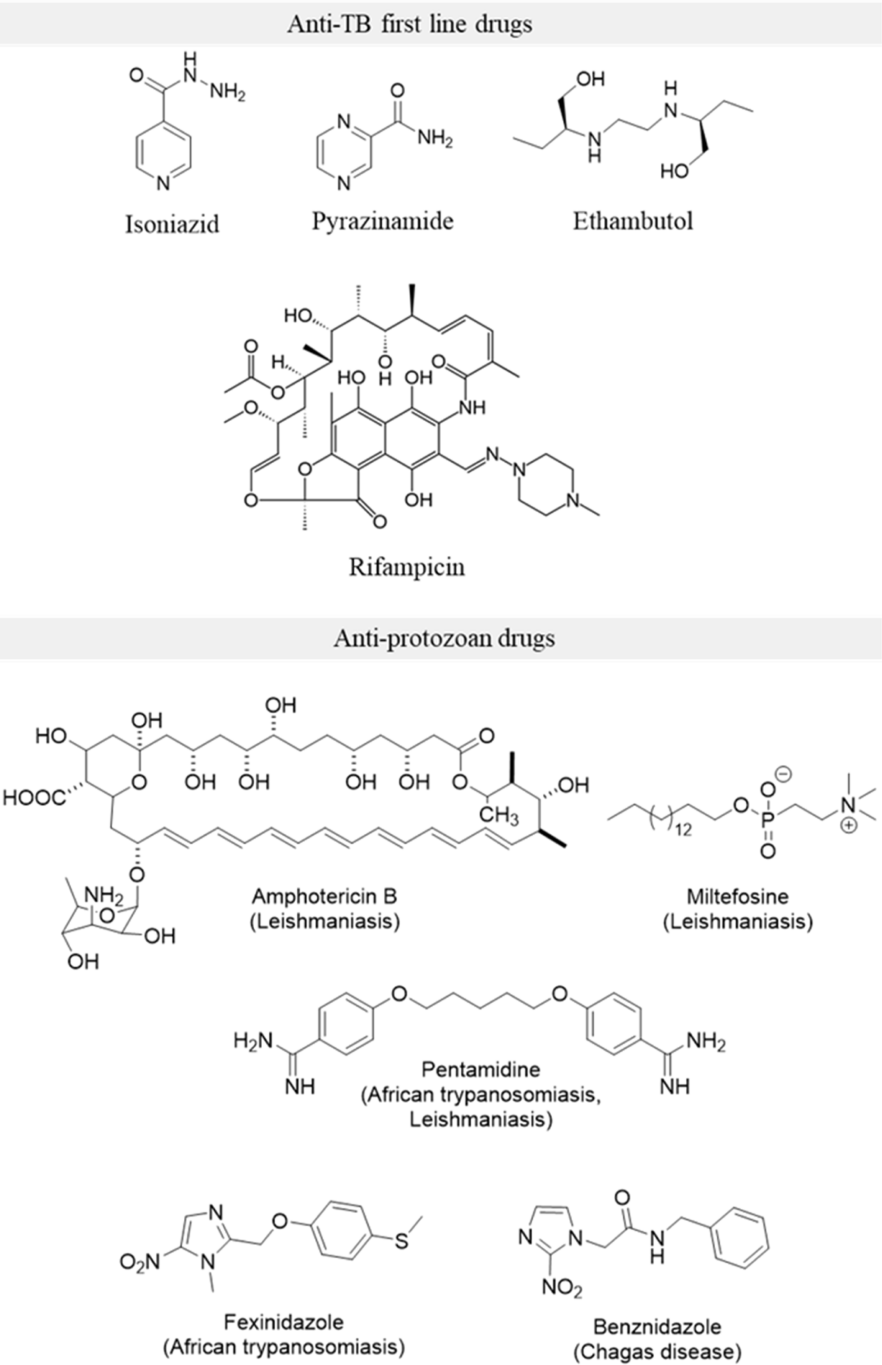
Representative chemotypes of known drugs used for the therapy of tuberculosis and protozoan (*Leishmania* and *Trypanosoma* spp.) infections.

Poverty-related infectious diseases such as tuberculosis, malaria, trypanosomiasis, and leishmaniasis afflict a massive global population. It has been estimated that, overall, over 200 million are affected or are at risk. The common problem among all these infectious diseases is the limited number of therapeutic drugs (Figure 1), their poor safety profile due to their toxicity, low compliance by patients, low accessibility, and drug resistance development.^1,2^ In the case of tuberculosis (TB) infections, the current standard treatment is effective, even though clinical practice suggests that patients with uncomplicated drug-susceptible TB are required to take multiple antibiotics for 6 months. Since compliance is low, WHO recommends that this must be directly supervised and possibly changed with a therapy that not only ensures higher compliance but is also shorter in duration and demonstrates effectiveness in the short term. This concept has generated a huge layer of infrastructure to the long treatment program. With the rise of drug resistance, treatment failure rates have also increased along with more toxic therapies that are far more costly and hence less accessible. Improved interventions could have a substantial effect on our ability to decrease the morbidity and mortality associated with the disease and to limit the further spread, as treatment of active TB is the major modality for preventing transmission in most of the world.

Recent analysis reports that 75% of all emerging human infectious diseases in the past three decades worldwide originated in animals.^3^ Poor and disadvantaged populations (subtropical regions), European Mediterranean countries may spread new infectious agents.^4^ Infections caused by Trypanosomatidae such as Chagas diseases, Human African Trypanosomiasis (HAT), and Leishmaniasis account for 17% of the estimated global burden of all infectious diseases (700,000 deaths/year).^5^ Chagas disease is caused by *Trypanosoma cruzi* (*Tc*) that is more diffused in South America; in its chronic form Chagas disease leads to severe organ damage, affecting mainly cardiovascular and digestive systems.^6^ HAT is mostly diffused in African countries and is caused by *Trypanosoma brucei* (*Tb*) subspecies; while *Tb rhodesiense* is responsible for a more acute infection and a faster-progressing disease that also affects the central nervous system, *Tb gambiense* establishes a chronic infection and a slow progression disease.^7^ Leishmaniasis is endemic in many tropical and subtropical countries and is caused by the infection of diverse *Leishmania* spp. inducing three main clinical forms, namely, cutaneous, mucocutaneous, and visceral diseases.^8^ All the aforementioned infections, addressed as neglected infectious diseases (NID), cause severe population burden if the drug candidate pipeline is not enriched. Therefore, there is an unmet medical need for novel medicinal chemistry efforts to develop new treatments. To accelerate the drug discovery of hits or leads, different technologies have been adopted. High-throughput screening (HTS) or medium-throughput screening (MTS) technologies have been largely performed as the preferred approach, in particular for phenotypic screening,^9,10^ and this has generally been more successful than a target-based approach, although both can be complementary for infectious diseases.

The management of the large amounts of data available from HTS approaches may require a powerful method of analysis. Machine learning approaches are being used increasingly in pharmaceutical drug discovery^11,12^ including application invvirtual screening for NID such as Ebola,^13^ Chagas,^14^ and against *Mycobacterium tuberculosis* (*Mtb*).^15−17^ Several groups, our own included, have employed these techniques, utilizing Bayesian models and other computational methods to analyze data sets and facilitate the drug discovery process.^15−22^ This has enabled the prioritization of compounds for testing^23^ and, in several cases, molecules with in vivo efficacy.^14^ In recent years, we have developed our own in-house machine learning software called Assay Central and demonstrated it by curating the *Mtb* data leading to 18,886 molecules (with activity cutoffs of 10 μM, 1 μM, and 100 nM).^21^ The 100 nM cutoff model was tested with an evaluation set (153 compounds) and showed statistics in line with those observed with 5-fold cross validation (accuracy 0.83).^21^ A more recent analysis of over 100 active leads for *Mtb* that are representative of thousands of active compounds generated over the decade suggested a very limited chemical diversity and this in turn will be reflected in any machine learning models generated with such data.^24^ The conventional approaches to drug discovery are identifying compounds that are the same or very similar to those preceding them, suggesting that our cheminformatics approaches need to evolve to go beyond the current chemical property space to find new leads. Databases related to structure activity relationships for NIDs and parasitic diseases are rare and ChEMBL^25^ represents a curated source of such data. Similarly PubChem^26^ is another source of such public domain data that can after some clean up and preparation be used for machine learning.^23^ In the present work, we apply various commercial (Discovery Studio) and proprietary (Assay Central) tools to machine learning to model all the data generated and use this to predict new compounds to test. In this process, we also compared multiple machine-learning algorithms with these data sets and demonstrate how such Bayesian machine learning models can used for lead optimization.

Some large compound libraries were collected from different consortia (More Medicines for Tuberculosis (MM4TB)^27,28^ and New Medicine for Trypanosomatidic Infection (NMTrypI)^29^ with the aim of finding a new antitubercular and/or an antiparasitic agent^30−36^ to overcome the unmet medical needs still represented by tuberculosis and parasite infections. The compounds come from a drug discovery and development studies aimed at optimizing the translation of compounds from the discovery phases to the preclinical and clinical models. One of the approaches in searching for new potential chemical hits adopted relied on the screening of noncommercial available libraries of compounds using a target-based or a cell-based screen. The in-house chemical libraries are usually assembled from compounds and intermediates produced during other medicinal chemistry investigations performed within the academic or industrial research group. This validated screening approach was also used to fish out from the unique Italian Institute of Technology (IIT) LIBRA compound library novel compounds targeting the pteridine reductase 1 (PTR1) enzyme from *T. brucei* (*Tb*PTR1) and from *Leishmania major* (*Lm*PTR1), validated parasite targets, which offer the potential to be progressed as African trypanosomiasis and Leishmaniasis drugs, respectively.^31^ Another application was developed to the identification of new antituberculosis compounds.^37^ These libraries might have the limitation to cover a small part of the putative very large chemical property space. To explore greater chemical diversity, we decided to investigate the antiparasitic and antitubercular potential of another in-house chemical library provided by TYDOCK Pharma, namely, Ty-Box library. The Ty-Box library herein disclosed (see Supporting Information) consists of 456 compounds characterized by a subgroup of sulfonamide derivatives. Many of these compounds were previously explored for their anti-infective activity. This characteristic is important as we were looking for anti-infective agents. The library profile with the respective molecular properties, including chemical-physical properties and human toxicity profile have not been tested previously. Therefore, this work also provides information that will be useful for future studies with these molecules for additional targets or diseases.

**Figure 2:**
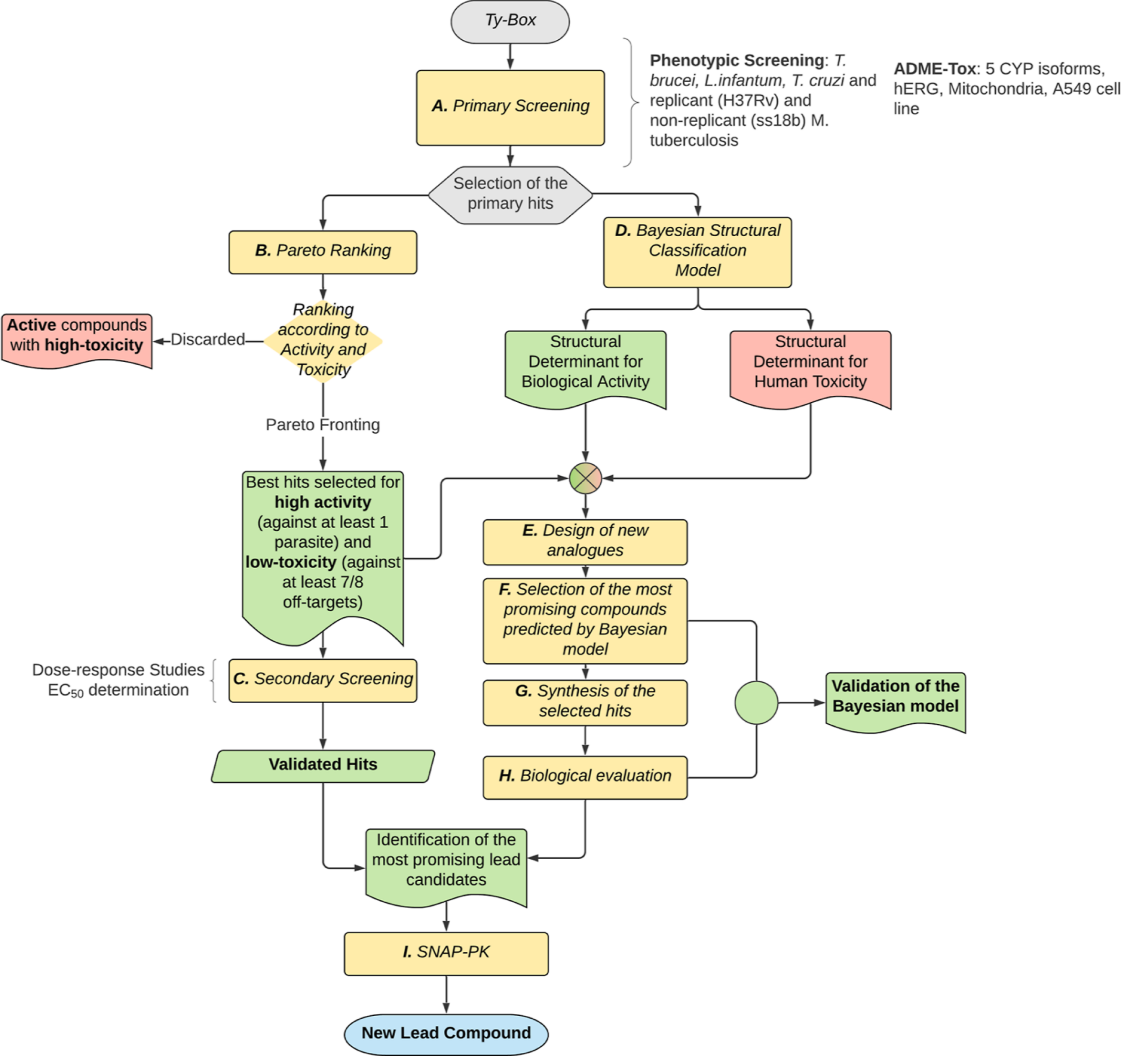
Workflow of the key actions of the study. Starting from the in-house Ty-Box library, the 456 noncommercial compounds were assessed (A) in a whole cell-based HTS primary screening against *T. brucei*, *L. infantum*, and *T. cruzi* and against replicant (H37Rv) and nonreplicant (ss18b) strains of *Mtb* and in parallel for in vitro human metabolic interference (five CYP isoforms) and toxicity (*h*ERG, mitochondria, and A549 cell line). The primary hits were prioritized using chemoinformatics and Pareto ranking (B) which identified the best hits with high activity and low toxicity from the active compounds. The selected primary hits were validated in secondary screening (C) for the determination of the dose–response curve. In parallel, Bayesian classification models (D) were generated to discriminate the structural determinants responsible for biological activity from those accounting for human toxicity. This information was exploited to design, prepare, and assess a second generation of hits (E–H) with the main purpose to validate the Bayesian models and to identify the most promising compounds to be evaluated in vivo for pharmacokinetic properties (I). One lead candidate was identified at the end of the discovery pipeline.

The workflow of the key actions of the study to identify investigational leads, is reported in Figure 2. The compound library was screened in whole cell-based HTS campaigns against three kinetoplasts (*T. brucei*, *Leishmania infantum,* and *T. cruzi*) and against replicant (H37Rv) and nonreplicant (ss18b) strains of *Mtb* (Figure 2A) with the objective to identify potentially pan-antiparasitic compounds or promising in vitro single compounds or class with a promising lead feature profile.^38^ Since the potential liability and toxicity represent a limiting bottleneck in the progression of the compounds in the preclinical phase, the entire library was evaluated at an early stage for drug–drug interactions involving cytochrome P450 enzymes (CYP) inhibition considering that compounds altering the CYP enzymes can alter the metabolic transformation of other drugs coadministered to the patients thus generically addressed as influencing drug–drug interactions.^39^ Other aspects of drug evaluation are associated with assessing human toxicity. This includes examining the *h*ERG (the human ether-à-go-go-related gene) for the evaluation of a potential cardiotoxicity due to the inhibition of potassium voltage gated channel; additionally, toxicity assessment against the A549 a human nonsmall lung cancer cell-line serve as a model to study compound cytotoxicity, whereas mitochondrial toxicity addresses the potential effects of investigational drugs on compounds metabolizing systems.^40^ These data have been achieved adopting in vitro HTS technologies for the antiparasitic activities (six strains). The large number of parameters generated in vitro in the study of the compounds of the Ty-Box library provided substantial data to support the use of the machine learning approach.

More than 20,000 data points have been generated that were then further processed using a machine learning methodology to generate a predictive model to identify and optimize compound features. The prioritization of compounds was guided by these chemoinformatic approaches to identify the best primary hits for antiparasitic potency and reduced/null human toxicity (Figures 2B,C and 1B,C). In addition, Bayesian classification models (Figure 2D) were generated to identify the structural features of each chemotype accounting for activity and toxicity to guide the design and synthesis of a second-generation compounds library of optimized hits to provide a quality lead compound with a potential for further refinement toward a preclinical candidate (Figure 2E–H). In summary, we first generated a large amount of in vitro biological data and then used machine learning methods as part of a combined virtual (chemoinformatic) and experimental biological HTS approach to identify new potential drug candidates for the treatment of broad-spectrum kinetoplastid infections.

## Results and Discussion

### Ty-Box Compound Library Properties

**Figure 3:**
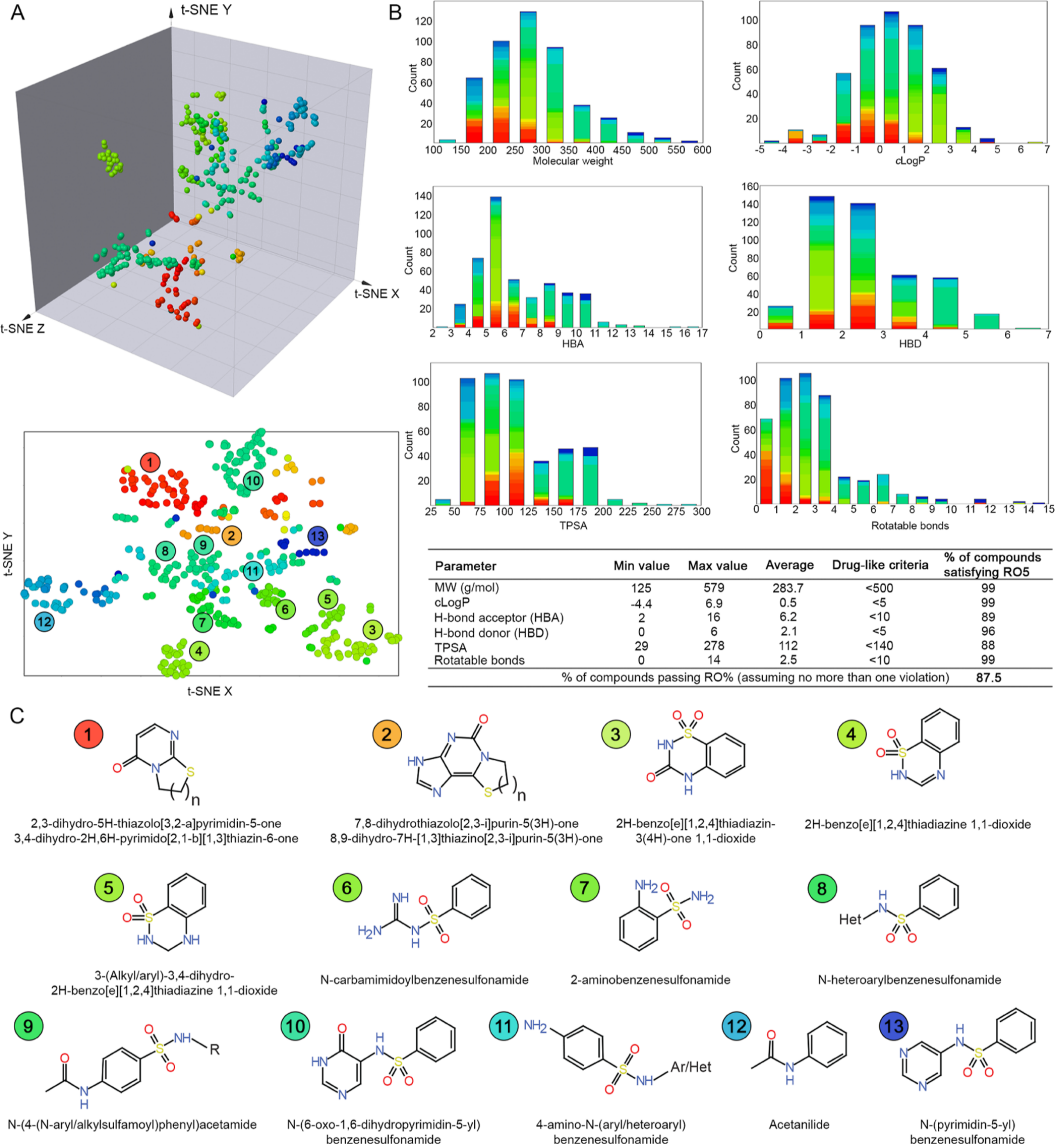
Chemical and physicochemical characterization of the Ty-Box library. (A) 3D and 2D *t*-SNE of the Ty-Box compound library. Clustering of the library compounds was based on the chemical similarity. Thirteen clusters were identified; the common chemical scaffold of the compounds of the cluster is reported in panel (C). (B) (Top) distribution of the extended rule-of-five parameters (RO5) for the 456 compounds of the Ty-Box library. The bars are color-coded according to the chemical clusters identified in the *t*-SNE; (bottom) resume of the physicochemical parameters of the Ty-Box compound library. (C) Scaffold analysis of the compounds from Ty-Box library. The most frequently occurring atomic frameworks (Murcko scaffolds) in more than ten representative compounds are reported.

The in-house Ty-Box compound library consists of 456 noncommercial small molecules synthesized and assessed during several drug discovery projects performed in the past several years by our research group. The chemical property space covered by the Ty-Box library was explored using extended-connectivity fingerprints of maximum diameter (ECFP-6) method and it was represented using 3D and 2D *t*-stochastic neighbor embedding (*t*-SNE) algorithm (Figure 3A). For all the 456 Ty-Box compounds, the drug-likeness, in accordance with the Lipinski’s “rule of five (RO5)”,^41^ was determined by computing molecular weight (MW), *c* log *P*, number of H-bond acceptors (HBA), number of H-bond donors (HBD), total polar surface area (TPSA), and number of rotatable bonds. Assuming no more than one violation of the rule, the 87.5% of the compounds were in accordance with the RO5 (Figure 3B). Moreover, the chemical space was analyzed for the maximal common substructure. Cluster analysis revealed 10 main clusters each containing >10 compounds (Figure 3C). Four main chemical families are represented in the Ty-Box: (i) the thiazole/thiazinepyrimidone, distinct in dihydrothiazolopyrimidinone/dihydropyrimidothiazinone (Figure 3C_1), and dihydrothiazolo (thiazino)purinone (Figure 3C_2); (ii) the acetanilide (Figure 3C_12); (iii) the benzothiadiazine (Figure 3C_4) and its congeners benzothiazinone (Figure 3C_3) and dihydrobenzothiadiazine (Figure 3C_5); and (iv) the benzenesulfonamide (BS). The latter is the most heavily represented family of compounds. The sulfonamide is decorated and derivatized with different and complex chemical functions or aromatic scaffolds that mask and deeply influence the chemical-physical-structural characteristics of these compounds so that it is possible to distinguish several independent subclasses, namely, benzenesulfonguanidine (Figure 3C_6), 2-aminobenzenesulfonamide (Figure 3C_7), *N*-heteroaryl-BS (Figure 3C_8), *N*-aryl/alkylsulfamoylphenylacetamide (Figure 3C_9), pyrimidonyl-BS (Figure 3C_10), 4-amino-*N*-arylbenzensulfonamide (Figure 3C_11), and *N*-pyrimidinyl-BS (Figure 3C_13). Besides, sulfonamide is a chemical function that is the basis of several groups of drugs, such as the antibacterial sulfonamides. The sulfonylureas and thiazide diuretics are another example of drugs based upon the antibacterial sulfonamides. Over time, the application of sulfonamides has been extended from their use as antimicrobial agents to anticarbonic anhydrase, antiobesity, diuretic, hypoglycemic, antithyroid, antitumor, and antineuropathic pain activities, among others.^42^ Thus, the abundance of sulfonamide compounds in the Ty-Box could be useful for the investigation of the potential of this functional group for the design of antiparasitic or antituberculosis drugs and represent a good premise for a pan-antiparasitic agent.

### High-Throughput Primary Screening against Kinetoplastids and *M. tuberculosis*

**Figure 4:**
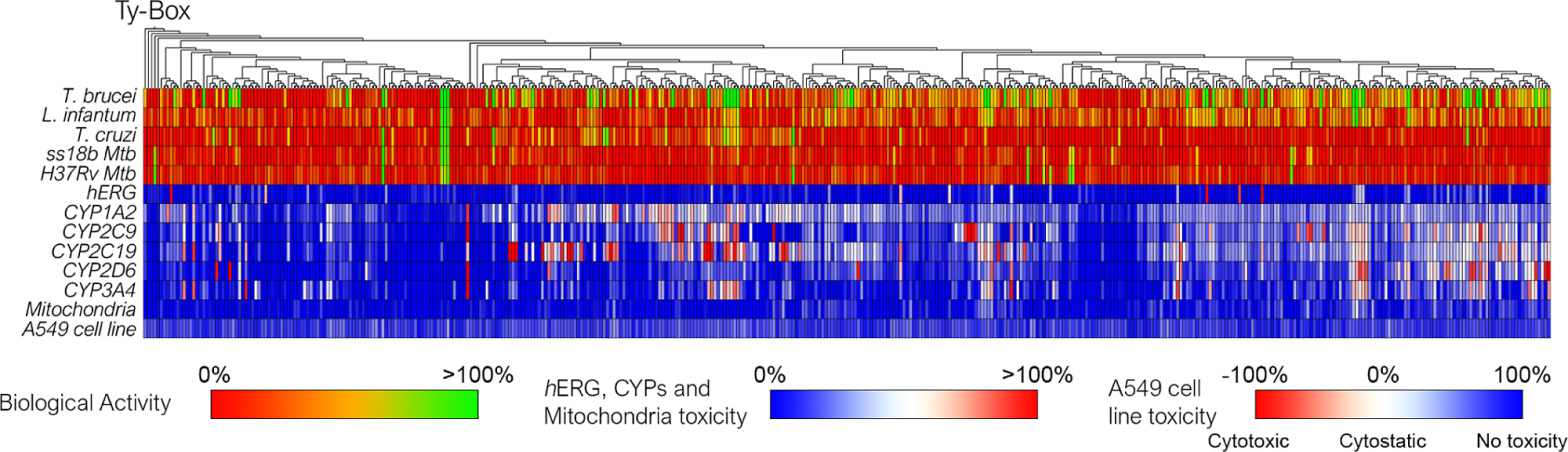
Hierarchical clustering groups hit compounds with a similar chemical structure based on the ECFP_6 fingerprint. Antiparasitic activity, drug–drug interactions, and early human toxicity emerged from primary screening for each compound is represented through a heat-map. All the data of the primary screening are reported in Table S1.

The Ty-Box library was tested against three kinetoplastid parasites (bloodstream form of *T. brucei*, amastigote *L. infantum,* and amastigote *T. cruzi*) and against replicant (H37Rv) and nonreplicant (ss18b) strains of *Mtb* using a primary whole-cell high-throughput phenotypic screening assay (step 2A, Figure 2). Results from the *Mtb* screening has been previously reported in Neres et al.^37^ However, no early toxicity data were obtained nor was chemoinformatics analysis performed. In the present work, all compounds were screened at 50 μM in the *T. brucei*, *L. infantum,* and *T. cruzi* assays, and at 20 μM in the *Mtb* assays. The overall results of the primary screening against the three kinetoplastids and *M. tuberculosis* are reported as a heatmap in Figure 4.

**Figure 5:**
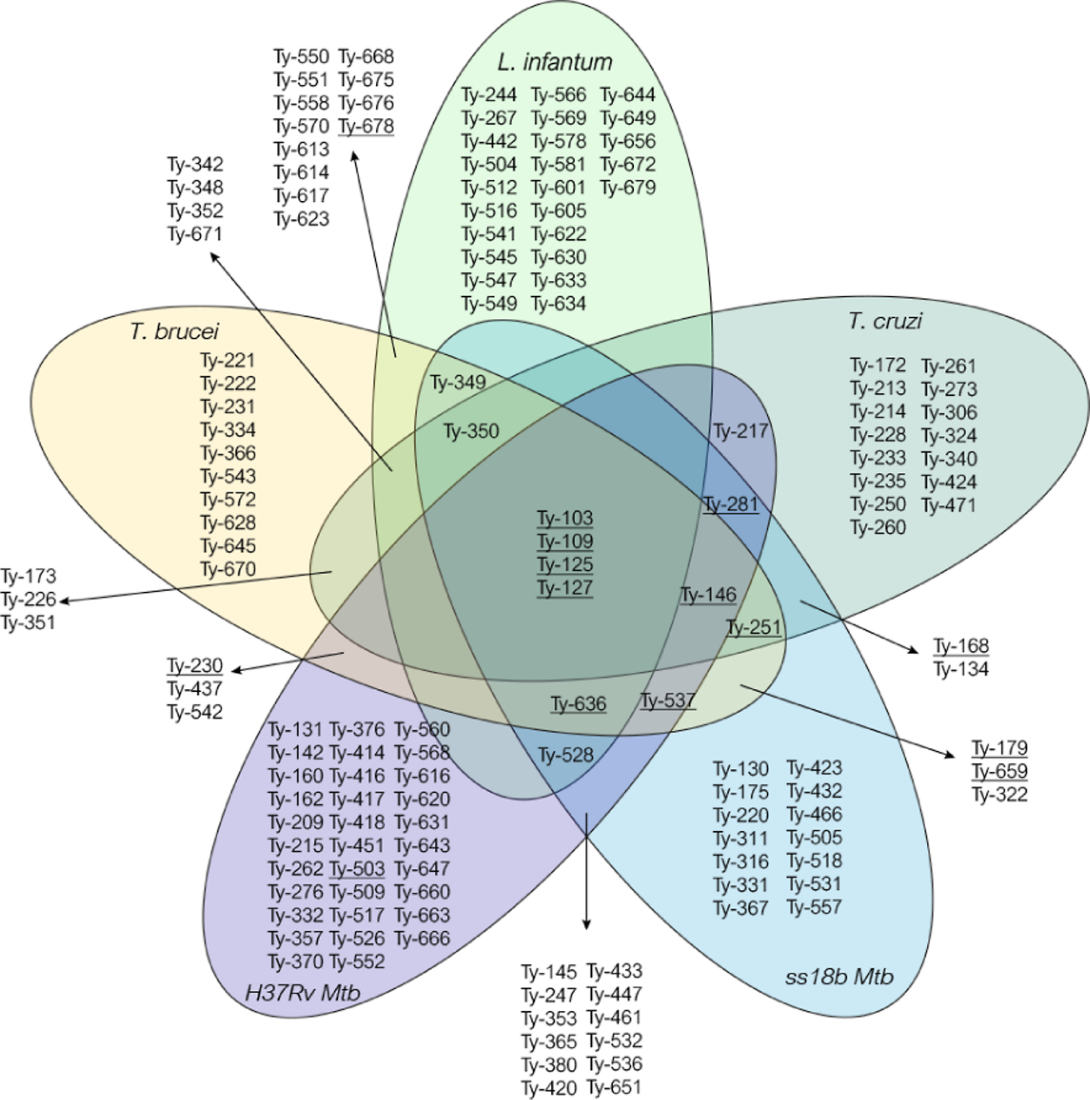
Venn’s diagram reporting the selectivity profile of the primary hits fished out from the HTS according to the cutoff: % of cell growth inhibition >80% at 50 μM against *T. brucei*, >40% at 50 μM against *L. infantum* and *T. cruzi*, and >20% at 20 μM against replicant and nonreplicant strains of *Mtb*. The hits prioritized by Pareto ranking for dose–response studies are underlined.

Among the 456 compounds of the Ty-Box library, 153 hits possess single or multiple antiparasitic and/or antitubercular activity (Figure 5). The *T. brucei* assay relies on indirect determination of parasite numbers by quantification of total DNA released from cells present in the wells of plates using the SYBR Green I DNA fluorescent dye. Pentamidine was used as reference compound exhibiting an EC_50_ of 1.6 ± 0.2 nM, which is comparable with the value reported in literature.^43,44^ The results are expressed as a percentage of cell growth inhibition at 50 μM. A cutoff of 80% of cell growth inhibition at the tested concentration was set for picking out the most active compounds (Figure 5), resulting in 48 primary hits (11% of the Ty-Box). In addition, 144 molecules (31% of the Ty-Box) showed a moderate activity in the range 30–78% of cell growth inhibition, whereas the remaining 266 compounds (58% of the Ty-Box) were found to be inactive. *L. infantum* antiparasitic activity was determined according to the method of Siqueira-Neto et al. procedure with some modifications.^45^ These compounds were screened in an intracellular assay of amastigote *L. infantum*-infected THP1-derived macrophages, which is a more physiological and disease-relevant model than those assays that rely on insect stages or axenic amastigote screens. Miltefosine and amphoterocin B were the positive controls, yielding EC_50_ 2.5 and 0.2 μM, respectively.^30^ A cutoff of 40% of cell growth inhibition at 50 μM was set since it is usually more difficult to identify antiparasitic agents able to hit the intracellular amastigote form of this parasite. Also in this case, a total of 48 primary hits were identified corresponding to an overall hit rate of 11% of the Ty-Box (Figure 5). Anti-Chagas activity was determined using human osteosarcoma U2OS cell-line infected with trypomastigote forms of the Y strain of *T. cruzi* in the presence of compounds. As for *L. infantum*, since *T. cruzi* is an intracellular parasite, the compounds were tested against the amastigote stage of the parasite for a more reliable antiparasitic evaluation. Infected cells were incubated for 72 h prior to fixation, staining, and quantification of antiparasitic activity by image analysis. Benznidazole was used as the reference, exhibiting an EC_50_ 2.4 μM, which is comparable with the value reported in literature.^46^ A cutoff of 40% of cell growth inhibition at 50 μM was set as for *L. infantum*, resulting in 45 hits with anti-*T. cruzi* activity (10% of the Ty-Box, Figure 5). Lastly, for the identification of the most promising hits with antitubercular activity against replicant (H37Rv) and nonreplicant (ss18b) strains of *Mtb*, a cutoff of 20% of bacterial growth inhibition at 20 μM was set. Only 10% of compounds (45 molecules) against the nonreplicant ss18b strains and 12% of compounds (57 molecules) against the replicant H37Rv strains showed bactericidal activity at the tested concentration (Figure 5).

### In Vitro Early ADME-Tox Related Studies

The entire Ty-Box library was assessed in parallel for potential early toxicity-related issues using HTS in vitro assays. These studies included five CYP isoforms (1A2, 2C9, 2C19, 2D6, and 3A4), for the evaluation of drug–drug interactions, *h*ERG liability, for the evaluation of a potential cardiotoxicity by inhibition of potassium voltage-gated channel, and mitochondrial toxicity and cytotoxicity in the A549 cell-line for preliminary evaluation of in vivo toxicity. The compounds were screened at 10 μM and the results reported as % inhibition (*h*ERG and CYPs), % toxicity (mitochondrial toxicity in A549 cell-line), and % viability in the A549 cell-line. The overall early in vitro ADME-Tox profile of the Ty-Box library is represented using a heat-map visualization (Figure 4) and the numerical data are reported in Table S1. Almost all compounds present a safe CYP inhibition profile with an average value of inhibitor activity around 15% at 10 μM, with few exceptions exceeding this trend. Interestingly, the entire library showed an overall negligible adverse effect against *h*ERG, mitochondrial toxicity, and cytotoxicity in the A549 cell-line. Pentamidine, benznidazole, amphotericin B, and miltefosine were used as reference controls and their early toxicity is in line with the literature data (Table S1).^30,36^

### Active and Not Toxic Hit Selection by Pareto Ranking and Validation in Secondary Assays

**Figure 6:**
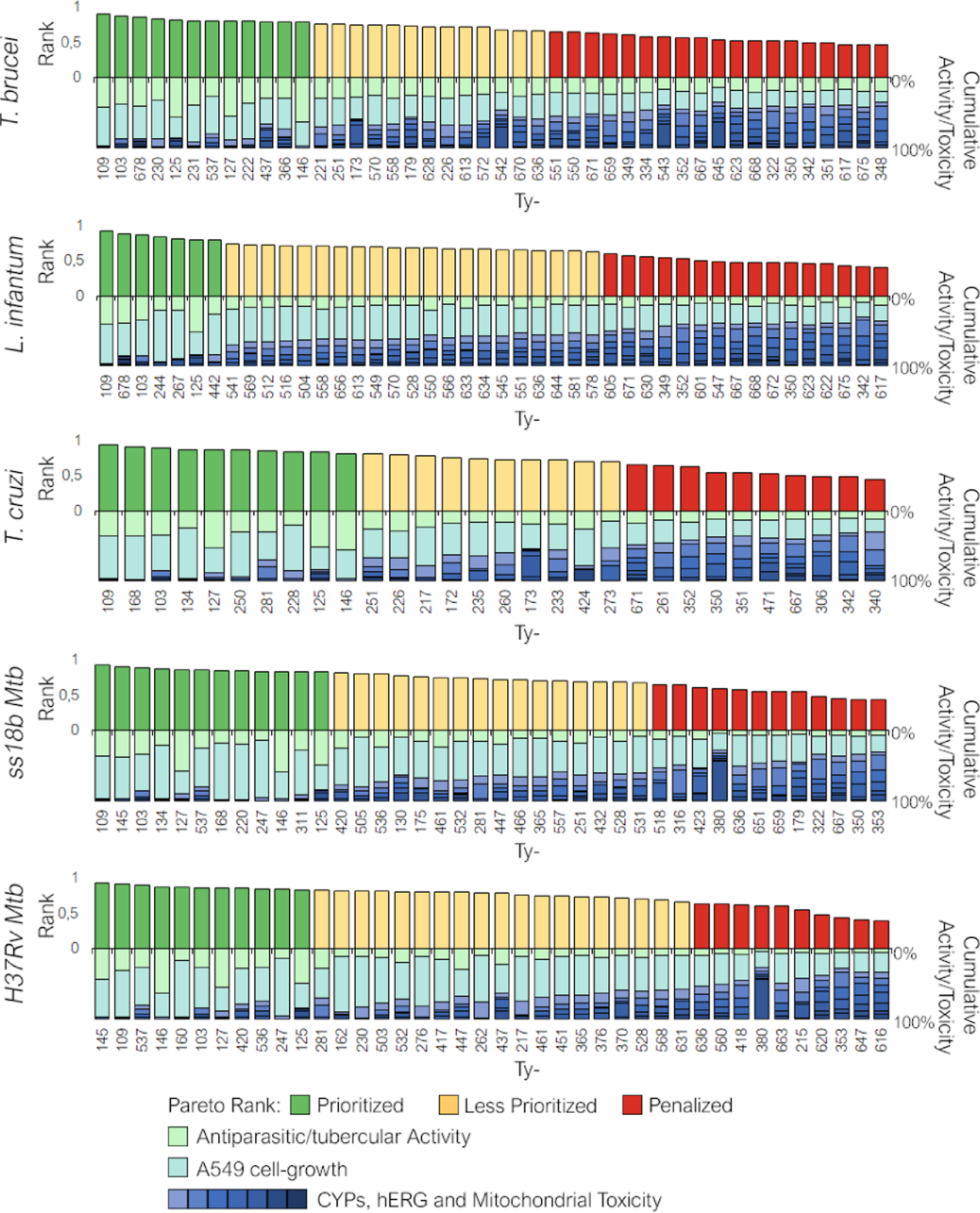
Prioritization of the primary hits active against at least one parasitic strain (according to the defined cutoff described in the main text) by Pareto ranking. The hits marked with a high score and reported with a green bar on the positive *y*-axis were prioritized and assessed in dose–response assays; conversely, the deprioritized or penalized hits (yellow and red bars, respectively) were rejected. In the negative *y*-axis is reported the cumulative activity and toxicity profile of the analyzed primary hits to highlight the weight of the antiparasitic activity (pale green bar), of the lack of A549 toxicity (aquamarine bar), and of the early toxicity (CYPs and *h*ERG inhibition and mitochondria toxicity, in gradient blue bars) on the Pareto rank.

Chemoinformatic techniques can assist the drug discovery process by analyzing the relationship between biological activity, toxicity, molecular properties, and chemical structure of the tested compounds to help prioritize selections and increase efficiency. Using the various chemoinformatic techniques, the bioactive hits resulting from primary screening reported in the previous paragraph, and collected in Table S1 in the Supporting Information, were ranked by a Pareto model. The best hits for activity against at least one parasitic strain and low/null toxicity (step B, Figure 2) were selected (compounds identified as “prioritized” in Figure 6) and validated in secondary screening (step C, Figure 2).

**Figure 7:**
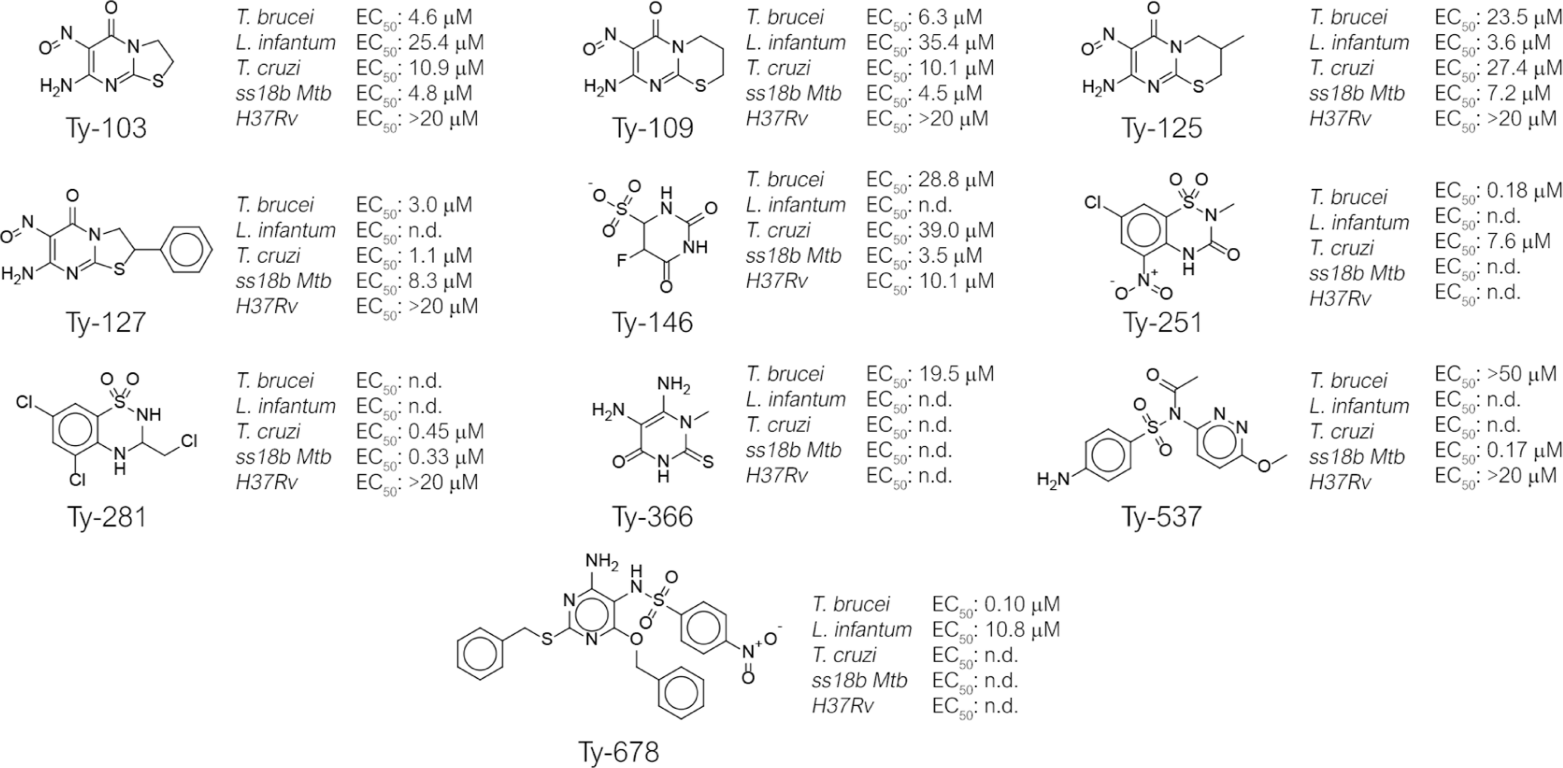
Chemical structures of the sole validated primary hits and the corresponding antiparasitic activity expressed as EC_50_. For compounds that did not agree with the defined cutoff against at least one parasitic strain, the dose–response was not performed (n.d., not determined). SD for all the assays is within ±10% of each value.

The Pareto ranking enabled the selection of compounds endowed with activity at least against one parasite and/or *Mtb* strain and with low potential early toxicity-related issues according to the cutoff criteria: percentage of cell growth inhibition >80% at 50 μM against *T. brucei*, >40% at 50 μM against *L. infantum* and *T. cruzi*, >20% at 20 μM against replicant and nonreplicant strains of *Mtb*, <50% at 10 μM for CYPs inhibition and mitochondrial toxicity, <20% for *h*ERG inhibition and >70% for A549 cell-growth (Figure 6). Twenty-seven primary hits were selected for the secondary assay, and dose–response studies were performed for the determination of EC_50_ values. The antiparasitic and/or antitubercular activity was confirmed for 10 out of 27 hits (Figure 7).

**Ty-103**, **Ty-109**, **Ty-125**, and **Ty-127** (Figure 7) were the most interesting compounds of the library since they exerted wide-antiparasitic activity against all three kinetoplastids and the two strains of *Mtb* with EC_50_ values in the low micromolar range (from 2.0 to 35.4 μM, Figure 7). These four primary hits share the dihydrothiazolpyrimidone scaffold (for **Ty-103** and **Ty-127**,Figure 3C, cluster 1) or its extended ring homologue dihydrothiazinpyrimidone scaffold (for **Ty-109** and **Ty-125**,Figure 3C, cluster 1). Although these hits possess a nitroso substituent on the pyrimidinone ring, in contrast with what was expected, they showed a safe metabolic and toxicological profile against the entire panel of targets considered in this study. The nitroaromatic moiety is a well-known motif recurring in several examples of antiparasitic agents.^47^ The presence of a nitro group is not a guarantee it will be toxic or substrate for nitro reductases, as the anti-TB drug delamanid (a nitroimidazole containing compound) is not a substrate for these enzymes.^48^ The expansion of the dihydrothiazinpyrimidone scaffold toward the tricyclic system of the dihydrothiazinopurinone (Figure 3C, cluster 2) overall led to a loss of antiparasitic and antitubercular activity. Another interesting chemical class resulting from the primary screening was based on the benzothiadiazine scaffold and its subclusters benzothiadiazinone (Figure 3C, cluster 3), benzothiadiazine, and dihydrobenzothiadiazine (Figure 3C, cluster 4). Twenty-five compounds out of 84 were active against at least one parasite strain in the primary screening and two of those (**Ty-251** and **Ty-281**, Figure 7) were prioritized by Pareto ranking and validated in the secondary screening.

**Ty-251** was the most promising of the two showing a dual antiparasitic activity against *T. brucei* (EC_50_ 0.18 μM) and *T. cruzi* (EC_50_ 7.6 μM). In contrast, moving from the benzothiadiazinone scaffold toward the dihydrobenzothiadiazine **Ty-281** (Figure 3C, cluster 5), the antiparasitic activity against *T. brucei* and *L. infantum* is completely lost, whereas it retained a submicromolar anti-Chagas activity with an EC_50_ of 0.45 μM, and antitubercular activity (ss18b *Mtb*) with an EC_50_ of 0.33 μM. The chemical class of the sulfonamides and the sub-clusters of the 2-amino-BS (Figure 3C, clusters 7 and 9), *N*-heteroaryl benzenesulfonamides (Figure 3C, clusters 8 and 11), *N*-pyrimidonylbenzen-BS (Figure 3C, cluster 10), and *N*-pyrimidinyl-BS (Figure 3C, cluster 13) were the most representative for the number of primary hits active at least against one parasite strain (76 active hits over 167 compounds). Among this heterogeneous class of compounds, only two of the prioritized primary hits were validated (**Ty-537**, and **Ty-678**, Figure 7). These two compounds showed anti-*T. brucei* activity with an EC_50_ ca. 50 and 0.1 μM, respectively. **Ty-537** (Figure 3C, cluster 7) showed in addition an interesting activity against replicant *Mtb* with an EC_50_ of 0.17 μM. Particularly interesting was compound **Ty-678** (Figure 3C, cluster 13) that, beside the nanomolar activity against *T. brucei* (EC_50_ of 0.10 μM), showed promising activity toward the amastigote stage of *L. infantum* (EC_50_ of 11 μM, Figure 7). Lastly, two primary hits belonging to the less representative chemical cluster of uracil were identified and validated. **Ty-146** (Figure 7) showed a broad antiparasitic and antitubercular activity with EC_50_ in the low micromolar range (EC_50_ of 28.8, 39.0, 3.5, and 10.1 μM against *T. brucei*, *T. cruzi,* and both replicant and nonreplicant *Mtb*, respectively). Interestingly, the analogue bioisoster thiouracil **Ty-366** retained solely the anti-*T. brucei* activity with an EC_50_ of 19.5 μM.

In summary, primary screening of the Ty-Box identified a pool of primary hits with diverse chemical scaffolds and promising antikinetoplastid and/or antitubercular activity. This represents a valuable starting point for initiating a chemoinformatic-guided hit-to-lead program aimed at the optimization of these primary hits toward the identification of a lead compound with a balanced activity and toxicity profile.

### Bayesian Models to Predict New Active and Nontoxic Hits

Bayesian machine learning modeling is a chemoinformatic approach useful for discriminating between user-defined active and inactive compounds present in a screening data set and therefore can be used to predict the likelihood of new hits not present in the training set (step D, Figure 2). Moreover, since the drug leads must not only be efficacious but also safe, the Bayesian model can be jointly adopted for predicting and discriminating potential toxic from safe compounds. Thus, Bayesian models can be constructed to correlate 2D chemical structural features of the compounds with activity and toxicity or lack thereof. We have therefore generated Bayesian models using the 456 molecules of the Ty-Box library as a training set, combining the bioactivity (against three different kinetoplastids and *Mtb*) and the enzymatic and cytotoxicity features. The screened molecules of the library were classified first into active/inactive and toxic/safe compounds according to the results achieved from each assay of the primary screening. The software was instructed using the cutoff values described above for discriminating active/inactive and toxic/safe primary hits. Compounds were set as active whether (i) antiparasitic activity >80% at 50 μM against *T. brucei*, (ii) antiparasitic activity >40% at 50 μM against amastigote *L. infantum* and *T. cruzi*, and (iii) activity >20% at 20 μM against both replicant and nonreplicant strains of *Mtb*. Analogously, compounds with % adverse activity in the early toxicity-related assays at 10 μM >50% against five CYP isoforms and mitochondrial toxicity, >20% against *h*ERG, and >30% A549 cytotoxicity were set as toxic. All the compounds that do not fulfill these criteria were considered inactive or safe compounds. Models were generated using a standard protocol in Discovery Studio (Biovia, San Diego, CA) with the following molecular descriptors: molecular function class fingerprints of maximum diameter 6 (FCFP_6), *A* log *P*, molecular weight, number of rotatable bonds, number of rings, number of aromatic rings, number of hydrogen bond acceptors, number of hydrogen bond donors, and molecular fractional polar surface area. The generated model was validated by receiver operating characteristic (ROC) plot based on the leave-one-out cross-validation. The ROC was calculated for each model and assumed as a measurement of the accuracy of the model to identify the true positive and the true negative. An ideal model should have an ROC of 1. The models generated for the prediction of *T. brucei*, *L. infantum,* and *T. cruzi* activity were particularly predictive with ROC curves between 0.7 and 0.8, whereas these ROC values were lower for the two *Mtb* strains, with an ROC between 0.64 and 0.68 for ss18b and H37Rv, respectively (Figure S1). Interestingly, the Bayesian machine learning models resulted in excellent predictions for the chemical features responsible for the inhibition of the five CYP isoforms and for the inhibition of *h*ERG, with ROC values ranging from 0.76 to 0.92. Lower ROC values were obtained for the models of mitochondrial toxicity and A549 cell-line toxicity (0.64 and 0.58, respectively) (Figure S2). Using the FCFP_6 descriptors, the fragments for Bayesian models were generated. The 20 highest- and lowest-scoring fragments accounting for activity/toxicity were also identified with the software. Given the clusters above, the top-scoring fragments accounting for antiparasitic activity were dominated by sulfonamide motifs (such as BZ, *N*-heteroarylbenzene-BS, *N*-pyrimidonyl-BS, and *N*-pyrimidinyl-BS) and bicyclic scaffolds (such as dihydrothiazinpyrimidone or dihydrothiazinopurinone). Conversely, the bottom-scoring fragments included the benzothiadiazine scaffold (and its variants), although few relevant exceptions to this trend were present especially against *T. cruzi*. Interestingly, although both sulfonamides and dihydrothiazine derivatives could account for some interference with the activity of the five CYP isoforms, they were reported to be less involved in human toxicity, especially against *h*ERG, mitochondrial toxicity, and cytotoxicity. We have also generated individual Assay Central machine learning models for these data sets using ECFP_6 descriptors alone. These results for 5-fold cross validation with Assay Central are summarized in Figure S3 and Table S2 and are very comparable to those produced with Discovery Studio and FCFP_6 descriptors. These models were also compared with a wide array of additional machine learning algorithms, namely, random forest, *k*-nearest neighbors, support vector classification, naïve Bayesian, AdaBoosted decision trees, and deep learning architecture as previously described.^49^ Radar plots for the various 5-fold cross validation metrics show broadly similar patterns (Figure S4), and when compared with the distance from the top normalized scores (Kruskal–Wallis), it shows that the random forest, support vector classification, and Assay Central Bayesian algorithm perform the best (Table S2). Similarly, the mean rank difference shows the same (Table S3). Different statistical tests on the rank normalized score such as Kruskal–Wallis (independent) did not show statistically significant differences (Table S4), whereas pairwise comparisons of the rank normalized score showed statistically significant differences for random forest, support vector classification, and our Assay Central Bayesian algorithm (Tables S5–S8 and Figure S5).

Given the large volume and complexity of the in vitro data generated by the diverse HTS assays performed on the compounds of the Ty-Box library, the chemoinformatic and machine learning tools allowed us to explore the biological and toxicological activities and to aid in the decision-making process toward the design and preparation of a second generation of compounds with improved activity/toxicity profile.

### Validation of Bayesian Machine Learning Models for Whole-Cell Activity, Design, and Selection of the Test Set of Compounds

**Figure 8:**
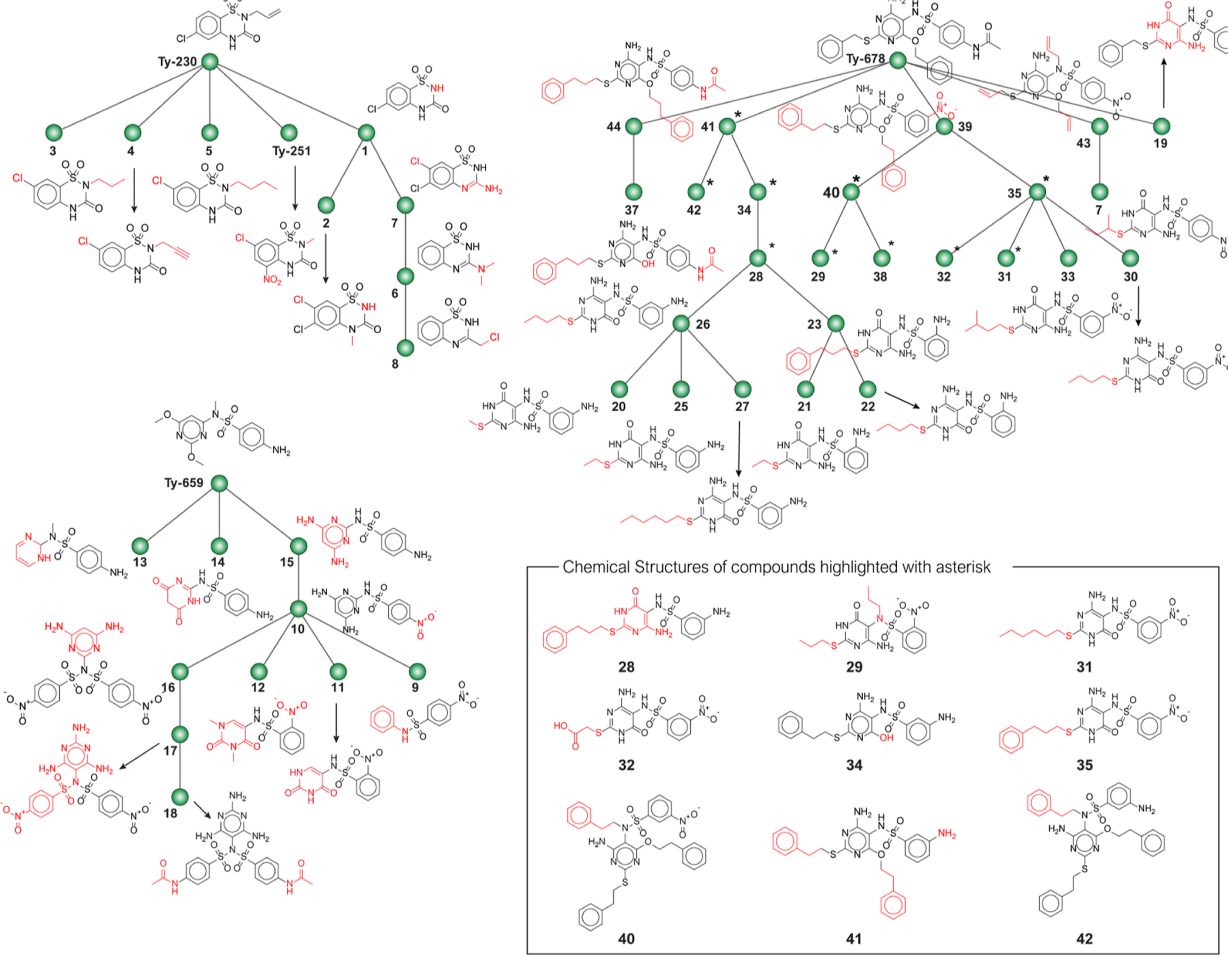
Structural similarity trees of the secondary hit compounds. The compounds were divided in three clusters according to the structural similarity and the derivation from the parent hits identified in the primary screening. The modification in the chemical structure of each hit, with the respect to the most similar analogues, is highlighted in red.

Given the potency and synthetic tractability of the validated hits and the identification of the chemical features responsible for activity and CYP inhibition, we initiated the medicinal chemistry elaboration focused on the optimization of the primary hits identified (while maintaining or improving the antikinetoplastids or antitubercular activity) and on the validation of the Bayesian models to establish early leads for further development. Thus, we mainly focused our attention on the chemical scaffolds of the benzothiadiazine (and its variants, clusters 3–5, Figure 3C) and *N*-pyrimidonyl-BS and *N*-pyrimidinyl-BS (clusters 10, 11, and 13, Figure 3C). **Ty-251** and **Ty-678** were assumed as reference compounds for each of these chemical classes and focused structure modification were introduced on the main scaffold. The virtual compounds were designed to be putatively active against at least one parasite strain and with null or reduced human toxicity. Putative decoy compounds were designed as well. In particular, the main scaffold of **Ty-251**, **Ty-659**, and **Ty-678** was decorated by introducing chemical modifications and substituents to map the chemical space around this and to draft a preliminary structure–activity relationship (SAR) necessary to further validate these primary hits identified to support the identification of a lead compound. The virtual library of compounds that was designed was filtered for predicted activity and toxicity through the previously elaborated Bayesian models and a resulting set of 47 secondary hits, **1**–**47**, were prioritized (Figure S6). We explored in particular the benzothiadiazine/one scaffolds variously decorated on the aromatic ring and on the nitrogen in position 2 as well as the reduction of the thiadiazinone ring to thiadiazine (Figure 8). Conversely, starting from the *N*-pyrimidinyl-BS scaffold two divergent structural modifications were examined. The chemical structure of **Ty-678** was modified by introducing a diverse alkyl or phenylalkyl chain at the sulfur or oxygen atoms of the 6-amino-2-mercaptopyrimidinol ring or by modifying the position of the amino or nitro group on the benzensolfonamidic moiety, whereas starting from **Ty-659**, we mainly focused on the bioisosteric replacement of the 2,4-dimethoxypyrimidine scaffold and on doubling the sulfonamide portion (Figure 8). Compounds **1**–**44** were thus synthesized, whereas compounds **45**–**47** were commercially available and purchased (Figure 8).

All the secondary hits were assessed for antiparasitic activity against *T. brucei*, *L. infantum,* and *T. cruzi* and for potential early toxicity-related issues, which also served as a test set for the prospective validation of the identified primary hits and assessment of the predictive capacity of our Bayesian models. From this point, we focused on the compounds active against the kinetoplastids of most interest to us.

### Description of Chemical Structures of the Test Set and Synthesis

**Scheme 1:**
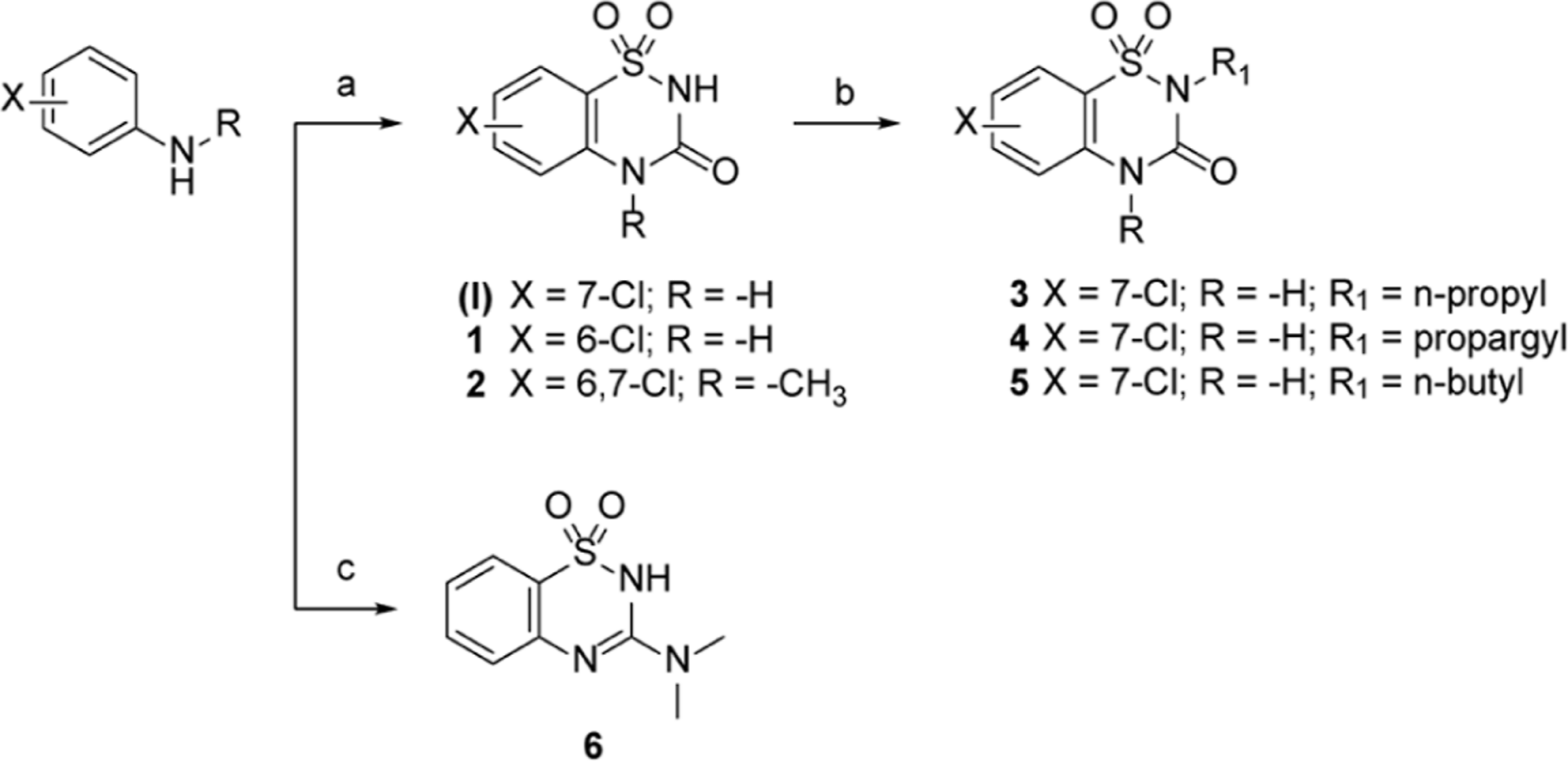
Reagents and conditions: (a) aniline (1 equiv), chlorosulfonyl isocyanate (1.2 equiv), nitromethane, 0 °C for 25 min and then AlCl_3_ (1.5 equiv), reflux, 30 min, 77% yield (for **I**), 62% yield (for **1**), 65% yield (for **2**); (b) alkylbromide (0.5 equiv), K_2_CO_3_ (1 equiv), DMF, r.t., 12 h, 85% yield (for **3**), 93% yield (for **4**), 72% yield (for **5**). (c) Aniline (1 equiv), *N*′-(chlorosulfonyl)-*N*,*N*-dimethylcarbamimidoyl chloride (1 equiv), DIPEA (2.2 equiv), dry DCM, r.t., N_2_, 1.5 h, 43% yield.

The synthesis of the benzothiadiazinones **1**–**5** is reported in Scheme 1. Compounds **1** and **2** and the intermediate **I** were prepared in good yield by the reaction of the appropriate aniline (6-chloroaniline for **1**, *N*-methyl-6,7-dichloroaniline for **2**, and 7-chloroaniline for **I**) with chlorosulfonyl isocyanate in nitromethane, followed by the addiction of anhydrous aluminum chloride. The intermediate **I** was further reacted with the appropriate alkyl bromide (bromopropane for **3**, propargyl bromide for **4** and 1-bromobutane for **5** in the standard Sn2 condition,^50^ using K_2_CO_3_ as base, in DMF at room temperature and overnight to afford the N^2^-substituted benzothiadiazinones **3**–**5**. Benzothiadiazines **6**–**8** were synthesized following different synthetic approaches according to the substituent to be introduced in position 3 on the benzothiadiazinic ring. **6** was directly obtained by reacting aniline with *N*′-(chlorosulfonyl)-*N*,*N*-dimethylcarbamimidoyl chloride and DIPEA in anhydrous DCM at room temperature for 1.5 h (Scheme 1).

**Scheme 2:**
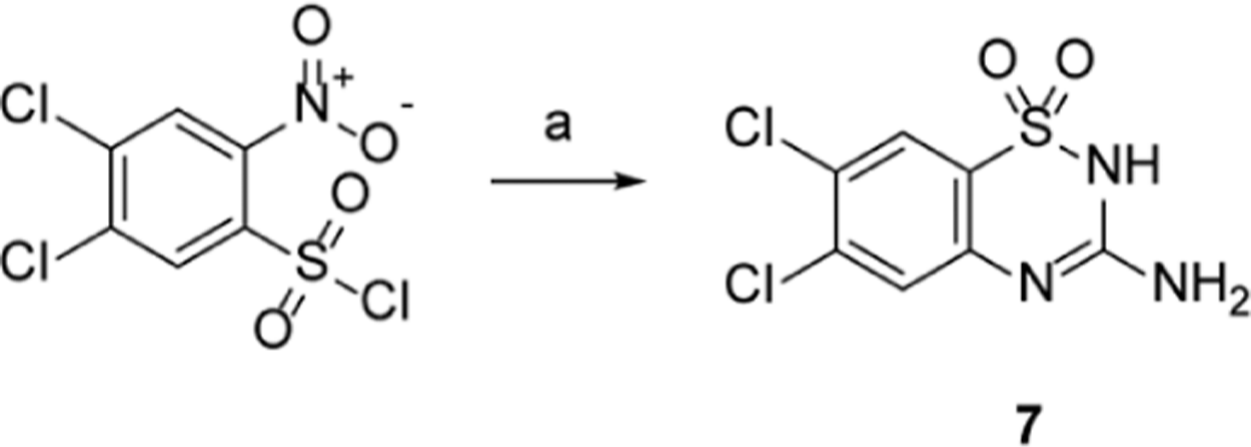
Reagents and conditions: (a) 5-aminotetrazole (1 equiv), 10% aq KOH, sodium dithionite (1 equiv) 40 °C for 1 h, then concd HCl (until acid pH), 60 °C, 5 h, 53% yield.

Conversely, **7** was prepared in one step following the procedure described by Peet et al. (Scheme 2).^68^ The commercially available 4,5-dichloro-2-nitrobenzenesulfonyl chloride was reacted first with 5-aminotetrazole in aqueous THF to provide the corresponding sulfonylcarbamidic azide (a ring opened version of the expected sulfonylaminotetrazole). The in situ reduction of the nitro group with alkaline sodium dithionite followed by the reaction in refluxing concentrated hydrochloric acid, provided the cyclization of the sulfonylcarbamidic azide to 3-aminobenzothiadiazine **7**.

**Scheme 3:**
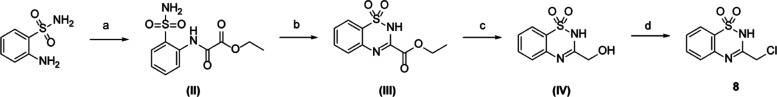
Reagents and conditions: (a) 2-aminobenzenesulfonamide (1 equiv), TEA (1 equiv), ethyl oxalyl chloride (1 equiv), dry THF, 0 °C to r.t, 5 h, 62% yield; (b) **II** (1 equiv), NaH 60% dispersion in mineral oil (1.05 equiv), dry EtOH, r.t., 2 h, 68% yield; (c) **III** (1 equiv), LiBH_4_ (2 equiv), dry THF, −78 °C, 5 min, 55% yield; and (d) **IV** (1 equiv), thionyl chloride, neat, 80 °C, 18 h, 90% yield.

Compound **8** was synthesized in a multistep approach starting from 2-aminobenzenesulfonamide and ethyl oxalyl chloride in anhydrous THF at room temperature for 5 h to afford ethyl 2-oxo-2-[(2-sulfamoylphenyl)amino]acetate (**II**) in 62% yield. By reaction with sodium hydride in anhydrous ethanol at room temperature for 2 h, **II** was cyclized into the corresponding benzothiadiazine **III**. The reduction of the ethyl ester with LiBH_4_ in anhydrous THF at −78 °C gave the corresponding alcohol (**IV**) which was converted into the resultant alkyl chloride **8** by the reaction in refluxing thionyl chloride **8** (Scheme 3).

**Scheme 4:**
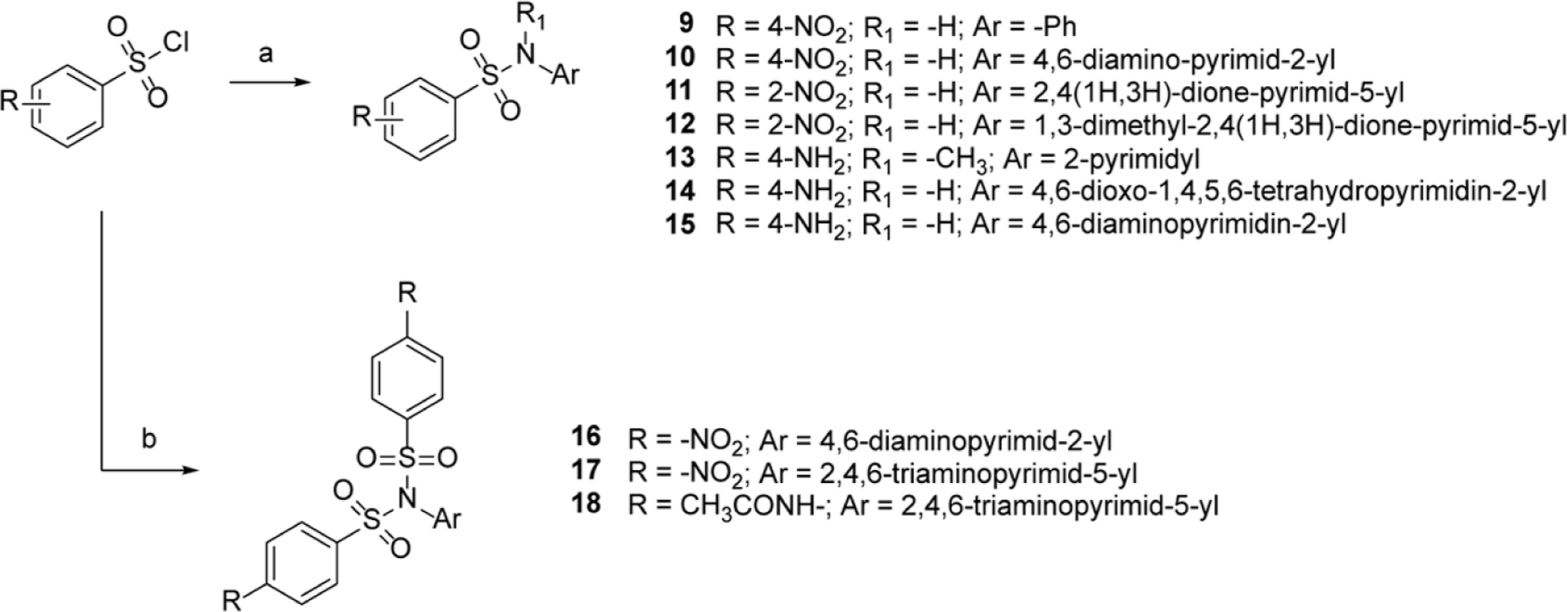
Reagents and conditions: (a) appropriate substituted benzensulfonyl chloride (1.1 equiv), aryl/heteroarylamine (1 equiv), pyridine, 0 °C to r.t., 2–3 h, 45–71% yield; (b) appropriate substituted benzensulfonyl chloride (2.5 equiv), aryl/heteroarylamine (1 equiv), pyridine, 0 °C to r.t., 6–12 h, 38–62% yield.

The aryl/heteroaryl-BS **9**–**15** were easily prepared in good yields by reaction of the substituted benzenesulfonyl chloride with the appropriate aryl/heteroaryl amine in dry pyridine at room temperature for 2–3 h (Scheme 4). The bis-aryl/heteroaryl-BS **16–18** were synthesized analogously to the monosubstituted benzenesulfonamide **9–15** but using 2.5 equiv benzenesulfonyl chloride and a longer reaction time (6–12 h) in order to promote the bis-substitution of the aryl/heteroarylamine (Scheme 4).

**Scheme 5:**
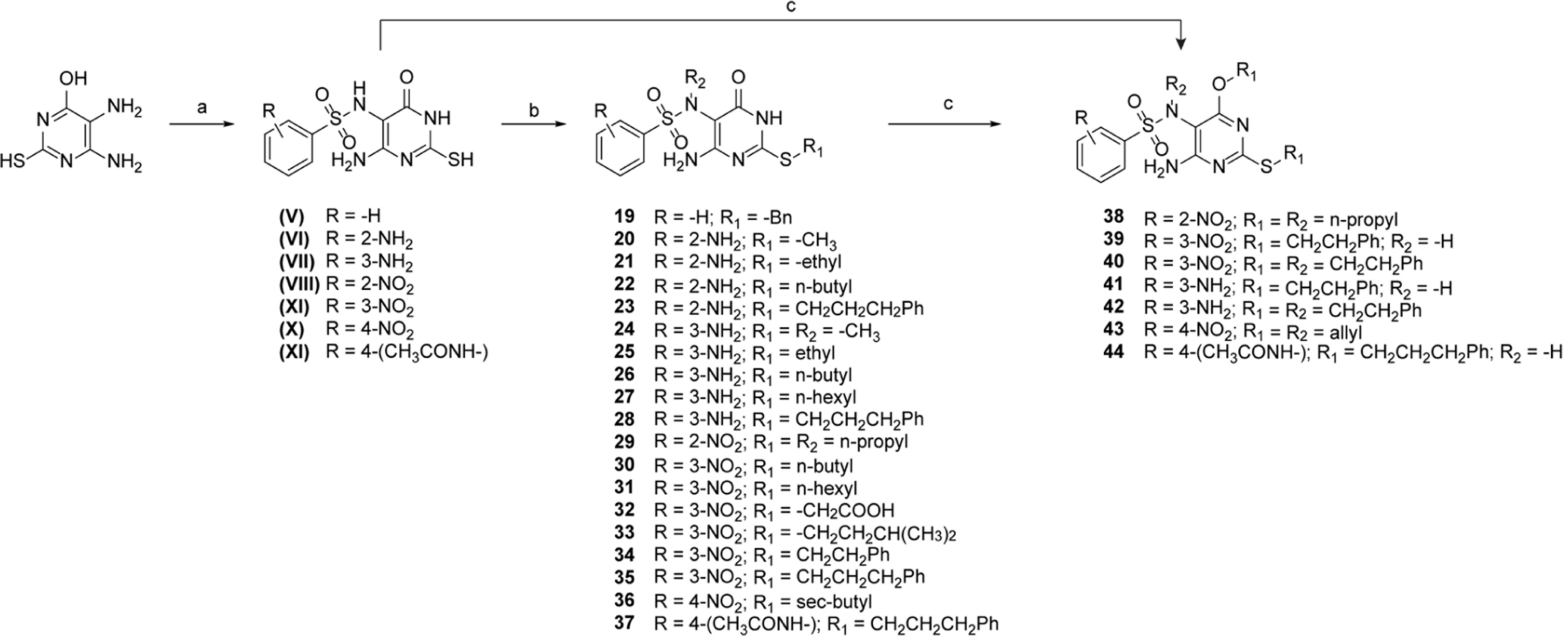
Reagents and conditions: (a) appropriate benzenesulfonyl chloride (1.2 equiv), 0.3 N NaOH, r.t. for 10 min then 80 °C, 1 h. (b) **V–XI** (1 equiv), alkylbromide (1.2 equiv), K_2_CO_3_ (1.1 equiv), DMA, 45 °C for 1 h then r.t.; and (c) excess of appropriate alkylbromide, K_2_CO_3_ (2.5 equiv), DMA, 80 °C, overnight.

Nevertheless, the synthesis of the substituted *N*-(4-amino-2-mercapto-6-oxo-1,6-dihydropyrimidin-5-yl)-BS (**V–XI**) required synthetic conditions divergent from those employed for the synthesis of the analogues **9–15**. A stronger base than pyridine and a higher temperature were necessary for the reaction to take place. Thus, 5,6-diamino-2-mercaptopyrimidin-4(3*H*)-one was reacted with the appropriate benzenesulfonyl chloride in aqueous 0.3 N NaOH at 80 °C for 1 h to give **V–XI** in high yield. Starting from intermediates **V–XI**, the final compounds **19–37** were synthesized by the S_N_2 reaction with the appropriate alkyl bromide. In order to maximize the yield of the reaction by selectively alkylating the thiol and avoiding the formation of the poly-substituted byproduct, the S_N_2 reaction was performed using 1.2 equiv alkyl bromide, a mild heating, and short reaction time (ca. 1 h). Conversely, for the synthesis of the bis- and tris-substituted *N*-(4-amino-2-mercapto-6-oxo-1,6-dihydropyrimidin-5-yl)-BS **38–44**, the above-described synthetic procedure was slightly modified, employing an excess of alkyl bromide (>2 equiv), higher temperature (80 °C rather than 45 °C), and longer reaction time (usually overnight). Compounds **38–40** and **44** were synthesized starting from the corresponding S-substituted *N*-(4-amino-2-mercapto-6-oxo-1,6-dihydropyrimidin-5-yl)-BS **29**, **34**, and **37**, respectively. In contrast, **41–43** were prepared with the same synthetic approach starting from intermediates **VII** and **X**. Bis- and tris-substituted *N*-(4-amino-2-mercapto-6-oxo-1,6-dihydropyrimidin-5-yl)-BS were prepared simultaneously and separated by column chromatography (Scheme 5). Lastly, **45–47** were commercially available and purchased.

### Biological Evaluation of the Test Set and Validation of the Bayesian Models

**Figure 9:**
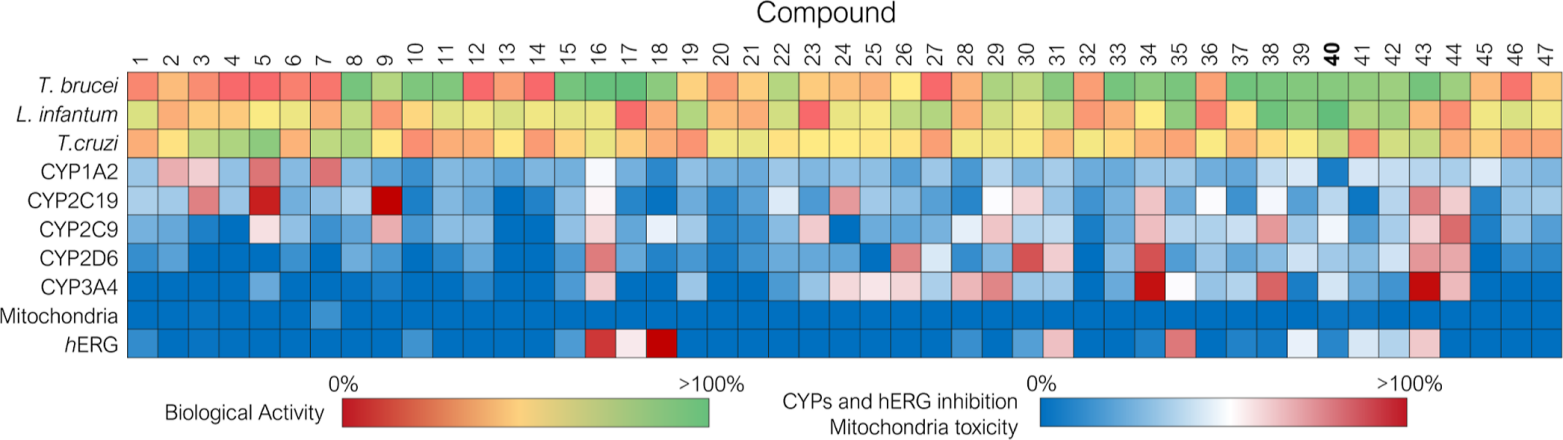
Heat map representation reporting the antiparasitic activity, drug–drug interactions, and early human toxicity for the secondary hits.

Regardless of the predicted activity by the Bayesian models against one or more parasite strains, the 47 secondary hits were screened first for the antiparasitic activity versus the three kinetoplastids. The results of the screening on the test set of compounds are reported in Table S9 and visually represented in Figure 9 as a heat map representation.

**Figure 10:**
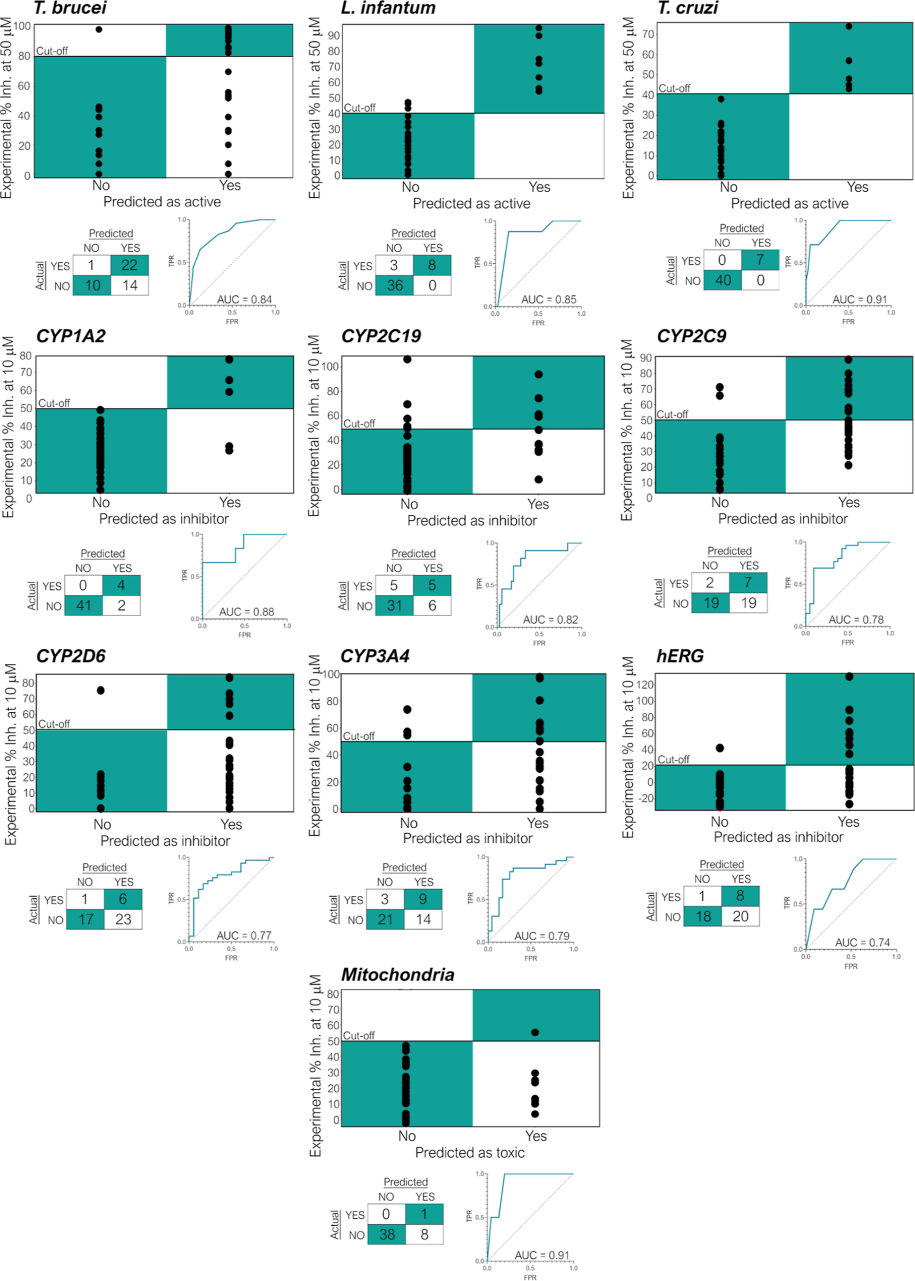
Statistical analysis for the validation of the Bayesian machine learning models by comparison of the predicted vs experimental data. For each target and off-target the correlation between the predicted state and the experimental activity/toxicity, the confusion matrix at the selected cutoff (antiparasitic activity >80% at 50 μM against *T. brucei*; antiparasitic activity >40% at 50 μM against amastigote *L. infantum* and *T. cruzi*; inhibitor activity >50% against CYP isoforms, >20% against *h*ERG and mitochondrial toxicity >50% at 10 μM) and the ROC curve for the evaluation of the diagnostic ability of Bayesian machine learning model to discriminate the true state of test set of compounds is reported.

The test compounds were divided in three clusters according to the structural similarity and the derivation from the parent hits identified in the primary screening. The compounds were tested at 50 μM and % cell growth inhibition is reported with a heat-map ranging from red (no cell growth inhibition) to green (100% of cell growth inhibition at the tested concentration). For the validation of the Bayesian machine learning model, the same cutoff values used in the primary screening were adopted for discriminating actives from inactives. Figure 10 shows the results of the study. In detail, 36 and 11 compounds were predicted to be active and inactive, respectively, against *T. brucei*. Among the 36 predicted active compounds, 22 showed inhibition >80% at the tested concentration, whereas 10 of 11 derivatives were inactive, as expected. The model for *T. brucei* therefore resulted in an accuracy of 68%. For *L. infantum,* a lower accuracy of 47% resulted for the test set against *L. infantum*. Although 34 test compounds were predicted to be potentially active against *L. infantum*, only 10 compounds showed inhibition >40% at the tested concentration. Conversely, this machine learning model seems to be more effective in predicting the inactive compounds against Leishmania since of the 13 compounds predicted as inactive, only one showed an antileishmania activity above the set cutoff. Interestingly, the most impressive results were achieved in the *T. cruzi* test set. While only a few test compounds showed a modest activity against *T. cruzi*, the Bayesian model was able to effectively discriminate between active and inactive compounds, resulting in an accuracy of 91%. Only six compounds were predicted to be active against *T. cruzi*, and 4 of them showed activity >40% at 50 μM, whereas among the 41 predicted inactive compounds, only 2 compounds showed a modest cell growth inhibitory activity. All the synthesized compounds were assessed for potential early toxicity-related issues and the results of the in vitro screening were compared with the potential liability predicted by the Bayesian models for the test set of compounds. Tables S10 and S11 shows the prediction by the elaborated Bayesian model versus experimental CYPs inhibition of the secondary hits and the prediction by the elaborated Bayesian model versus experimental mitochondria and *h*ERG toxicity of the secondary hits, respectively. Interestingly, the models resulted in an accuracy of 86% for *h*ERG, 87% for CYP1A2, 91% for CYP2C19, 70% for CYP3A4, and 77% for mitochondrial toxicity. Conversely, a fair accuracy was achieved against CYP2C9 and CYP2D6, although the models were able to discriminate more effectively the nontoxic compounds. However, to identify only the compounds with the most promising antiparasitic activity, more stringent selection criteria were applied. The secondary hits showing a *T. brucei* growth inhibition >80% at 50 μM were subjected to a secondary screen by performing a dose–response assay for the determination of the EC_50_. Of the 22 tested compounds responding to this criterion, 11 were confirmed with low micromolar anti-*T. brucei* activity with EC_50_ 2.4–12.7 μM (**8**, **16–18**, **38**, **40**, and **44**) (Figure 8). Conversely, compounds **9**, **11**, **22**, **29**, **33–35**, and **37** resulted in modest actives with EC_50_ > 50 μM, whereas three compounds, namely, **15**, **30**, and **31** were highlighted as false positives since their anti-*T. brucei* activity was not confirmed in secondary screening, resulting in being completely inactive. When tested in primary screening against amastigote *L. infantum* just compounds **38** and **40** yielded inhibition >40% at 50 μM. However, only compound **40** was confirmed as antileishmania agent in secondary screen, whereas **38** resulted in a false-positive in the primary screening since its activity was not confirmed in the dose–response assay. Lastly, none of the compounds showed any promising anti-*T. cruzi* activity in the primary screening and therefore they were not investigated further. In summary, the test set exhibited an overall antiparasitic profile with a higher number of compounds showing anti-*T. brucei* activity (Figure 9).

**Figure 11:**
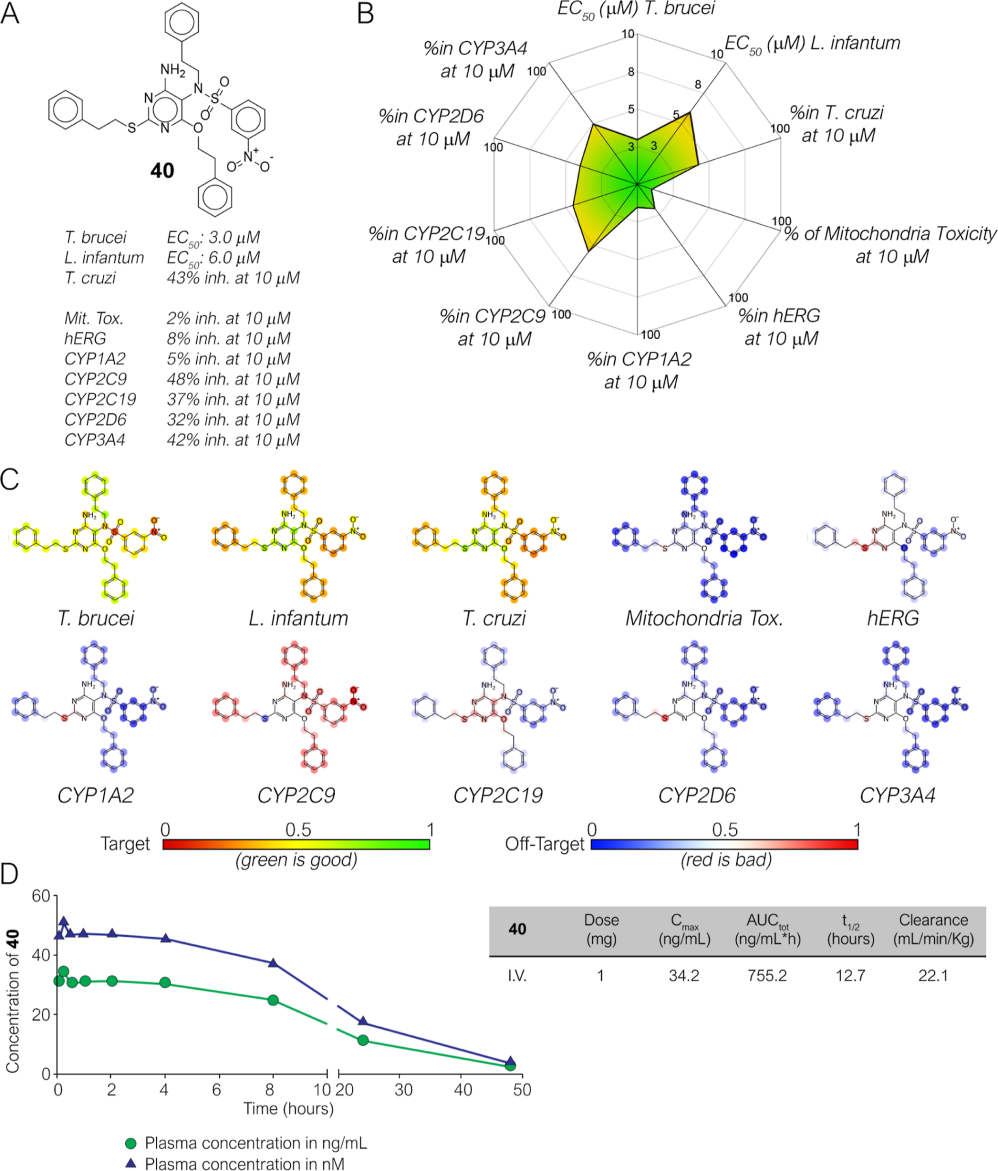
Biological activity summary of **40**. (A) Chemical structure of the secondary hit **40**; (B) summary of the antiparasitic activity and liability of **40**. (C) Atom coloring of **40** with Assay Central Models for targets and off-targets generated in this work. (D) Pharmacokinetic profile for compound **40** after IV administration at the dose of 1 mg/Kg in mice.

By analyzing the antiparasitic and toxicity profile of the 47 test compounds, compound **40** (Figure 11A) resulted as the most promising derivative. Indeed, it showed a confirmed dual antiparasitic activity against *T. brucei* and *L. infantum* with IC_50_ values of 3.0 and 6.0 μM, respectively. Moreover, with a 43% cell growth inhibitor activity at 10 μM against amastigote *T. cruzi* (IC_50_ of ca. 12 μM) it could represent a valuable starting point for the further design of a pan-antikinetoplastid compound (Figure 11B). While other compounds showed an interesting activity and a safe early toxicity profile, only compound **40** was confirmed in secondary assays (e.g., **8** was not confirmed in the same assays). Besides, **40** showed a % of inhibition toward *h*ERG, and all CYPs between 16 and 48% and a minimal mitochondrial toxicity (8% at 10 μM) (Figure 11B). The strongest inhibitory effect was observed only on CYP2C9. These results for antiparasitic activity and early toxicity are in accordance with the prediction obtained from machine learning models which successfully predicted for **40** required for biological activity and early toxicity (Figure 11C). The toxicity profile shows a few liabilities, but this is also likely linked to the presence of the nitro moiety. The nitro group should not always be regarded as a toxicophore, in particular when the drugs are used as antiparasitic agents.^47,51^ A few existing drugs contain the nitro group such as nifurtimox, benznidazole (for Chagas disease treatment), and fexinidazole that has been recently approved for HAT. However, if the compound proceeds further in the development program, mutagenicity assays will represent an important test to be performed. To evaluate the novelty of the identified lead, a similarity structure search of **40** was performed and provided only two substances with a structure similarity between 70 and 74%. The outcome of the research in different databases (Reaxys, SciFinder, and ChEMBL) clearly indicates that compound **40** represents a new and unexplored molecule. Based on the results achieved so far, **40** was elected as a promising hit with dual antiparasitic activity and low or null early toxicity, to be investigated in further in vivo studies. Before proceeding to in vivo studies, the preliminary pharmacokinetic profile of **40** was evaluated in healthy BALB/c mice using a SNAP-PK approach. **40** was administered I.V. at a dose of 1 mg/Kg. Interestingly, after a single dose administration compound **40** showed a long half-life (*t*_1/2_ 12.7 h), and it was not detected in blood after 48 h (Figure 11D). The compound reached the maximum plasma concentration of *C*_max_ 51.4 nM that is lower than its anti-*T. brucei* activity in vitro (EC_50_ 3 μM). A significantly higher dose can be used (e.g., 100 mg/Kg if tolerated) and different delivery formulation or route should be attempted for the progression of the compound to in vivo antiparasitic activity in infected mice. Compound **40** therefore represents a new pharmaceutical tool as an unexplored chemical scaffold for the design of improved anti-*T. brucei* compounds and it will subsequently be the subject of a lead optimization program to improve its properties.

## Conclusions

In summary, a workflow was applied to prioritize the hits resulting from a HTS phenotypic screening of the Ty-Box library of 456 diverse chemical compounds and in order to identify new lead compounds against kinetoplastidae and/or *Mtb*. We progressed stepwise with the compound selection and obtained different readouts (Figure S7). We explored the large amount of activity and toxicity data generated by HTS with several computational approaches that are unique in the realm of NID research and have been successfully implemented in the NID drug discovery pipeline. Over 20,000 data points were generated that required the use of cheminformatic and machine learning approaches to assist in lead identification. We have subsequently demonstrated that antiparasitic and antituberculosis drug discovery can exploit these Bayesian machine learning models to assist in selecting compounds for in vitro screening that may have a higher probability of activity against *T. brucei*, *L. infantum*, *T. cruzi,* and *Mtb*, while at the same time a lower probability of undesirable off-target effects due to CYP inhibition, *h*ERG inhibition, mitochondrial toxicity, or cytotoxicity. These new compounds could in turn lead to novel therapeutic treatments. The integrated in vitro and computational approaches also highlighted molecular substructures that contributed to the various activities and toxicities measured in vitro. Ultimately, the synthesis of similar and focused analogues solved some of the chemical or biological liabilities that would have prevented the molecules from proceeding further in the pipeline (Figure S7). Compound **40** was successfully identified as a new chemical entity, with low similarity to existing molecules, with a dual inhibitory activity against the parasitic enzymes and low toxicity. It is anticipated that the improved accessibility of such drug discovery data sets when combined with the machine learning tools like those described herein will facilitate the calculation of these activities more routinely in drug discovery. The outcomes of this research are publicly disclosed for the first time. We expect that this information will enable the scientific community to seed future lead discovery programs, apply and validate other computational approaches, and eventually we hope to aid in delivering innovative treatments for these important, but neglected diseases.

Finally, we have attempted a balanced use of different machine learning tools to support decision-making in drug discovery which led us to identify innovative, optimized, and developable compounds. Our efforts also used objective criteria and clearly identified chemical/biological liabilities and opportunities in a previously unexplored compound library in the field of NID.

## Experimental Section

### Synthetic Procedures

All reagents, solvents, and other chemicals were used as purchased without further purification unless otherwise specified. Air- or moisture-sensitive were performed under an argon atmosphere. Reactions were monitored by thin-layer chromatography on silica gel plates (60F-254, E. Merck) and visualized with UV light, cerium ammonium sulfate, and alkaline KMnO_4_ aqueous solution. Column liquid chromatography (LC) purifications were carried out using Merck silica gel 60 (230–400 mesh, ASTM). Flash chromatography purification was performed with an ISOLERA SP1-Biotage system. The structures of all isolated compounds were ensured by nuclear magnetic resonance (NMR) and mass spectrometry. ^1^H and ^13^C NMR (1D and 2D experiments) spectra were recorded on a DPX-400 Avance (Bruker) spectrometer at 400 MHz or on a DPX-600 Avance (Bruker) spectrometer at 600 MHz. Chemical shifts are expressed in ppm (δ) and calibrated on the residue signal of the solvent: CDCl_3_: δ 77.04, CD_3_OD: δ 49.8, DMSO-*d*_6_: δ 39.5. NMR data are reported as follows: chemical shift, multiplicity (s, singlet; d, doublet; t, triplet; q, quartet; qnt, quintet; sxt, sextet; m, multiplet; br, broad), coupling constants (Hz), and number of protons/carbons. The following solvent and reactive and chemical moieties were abbreviated: ethyl acetate (AcOEt), dimethyl sulfoxide (DMSO), dichloromethane (DCM), cyclohexane (CE), diethyl ether (Et_2_O), methanol (MeOH), ethanol (EtOH), tetrahydrofuran (THF), dimethylformamide (DMF), and triethylamine (TEA). Mass spectra were recorded on a 6520 Accurate-Mass Q-TOF LC/MS and 6310A Ion TrapLC-MS(n). The melting point was recorded on a Stuart, SMP3 (Barloworld Scientific Limited Stone, Staffordshire, UK) and was uncorrected. All compounds showed a purity ≫95% as evaluated from elemental analysis. ^1^H NMR and melting point data were reported for all the synthesized compounds.

#### Synthesis of 7-Chloro-2*H*-benzo[*e*][1,2,4]thiadiazin-3(4*H*)-one 1,1-Dioxide (**I**)

A solution of 4-chloroaniline (5 g, 39.2 mmol, 1 equiv) in nitromethane (15 mL) was added to a stirred, cooled solution (at −78 °C) of chlorosulfonyl isocyanate (6.65 g, 4.083 mL, 47.032 mmol, 1.2 equiv) in nitromethane (15 mL) during 10 min, and the reaction was further stirred for 25 min. The temperature of the mixture was allowed to increase to 0 °C and anhydrous AlCl_3_ (7.828 g, 58.708 mmol, 1.5 equiv) was added to give a clear solution. The reaction mixture was refluxed for 30 min, cooled, and poured in ice water (150 mL). The solid thus precipitated was filtered, washed with water, and decolorized with animal charcoal. It was recrystallized from ethanol to give a pale brown powder (7.020 g, 77% yield). ^1^H NMR (600 MHz, DMSO-*d*_6_): δ 7.26 (1H, d, *J* = 8.8 Hz), 7.69 (1H, dd, *J* = 8.8, 2.0 Hz), 7.82 (1H, d, *J* = 2.0 Hz), 11.39 (1H, s).

#### Synthesis of 6-Chloro-2*H*-benzo[*e*][1,2,4]thiadiazin-3(4*H*)-one 1,1-Dioxide (**1**)

A solution of 4-chloroaniline (1 g, 7.838 mmol, 1 equiv) in nitromethane (10 mL) was added to a stirred, ice cooled solution of chlorosulfonyl isocyanate (1.33 g, 0.816 mL, 9.406 mmol, 1.2 equiv) in nitromethane (10 mL) during 10 min, and the reaction was further stirred for 25 min. Anhydrous AlCl_3_ (1.565 g, 11.741 mmol, 1.5 equiv) was added to give a clear solution. The reaction mixture was refluxed for 30 min, cooled, and poured in ice water (100 mL). The solid thus precipitated was filtered, washed with water, and recrystallized from ethanol to give a pale orange powder (1.130 g, 62% yield). mp [302–304 °C]. ^1^H NMR (400 MHz, MeOD): δ 7.79 (d, *J* = 8.5 Hz, 1H), 7.31 (dd, *J* = 8.5, 1.4 Hz, 1H), 7.23 (d, *J* = 1.8 Hz, 1H). ^13^C NMR (101 MHz, MeOD): δ 152.34, 140.83, 137.97, 125.13, 124.94, 123.15, 117.78. Anal. Calcd for C_7_H_5_ClN_2_O_3_S: C, 36.14; H, 2.17; N, 12.04. Found: C, 35.98; H, 2.59; N, 11.99; HRMS (*m*/*z*) (ESI): calcd for C_7_H_7_ClN_2_O_3_S [M + H]^+^, 232.9782; found, 232.9769.

#### Synthesis of 6,7-Dichloro-4-methyl-2*H*-benzo[*e*][1,2,4]thiadiazin-3(4*H*)-one 1,1-Dioxide (**2**)

A solution of 3,4-dichloro-*N*-methylaniline (8.0 g, 50 mmol, 1 equiv) in nitromethane (25 mL) was added to a stirred, ice cooled solution of chlorosulfonyl isocyanate (8.5 g, 60 mmol, 1,2 equiv) in nitromethane (25 mL) during 5 min, and the reaction was further stirred for 25 min. Anhydrous AlCl_3_ (10 g, 75 mmol, 1.7 equiv) was added to give a clear solution. The reaction mixture was refluxed for 30 min, cooled and poured in ice water (150 mL). The thus precipitated was filtered, rinsed with water, and recrystallized from ethanol to give compound **2**. Pale yellow solid. Yield 65%. mp [318–320 °C]. ^1^H NMR (600 MHz, DMSO): δ 7.87 (s, 1H), 7.77 (s, 1H), 3.44 (s, 3H). ^13^C NMR (151 MHz, DMSO): δ 155.42, 138.67, 135.41, 126.79, 126.30, 124.24, 119.83, 35.35. Anal. Calcd for C_8_H_6_Cl_2_N_2_O_3_S: C, 34.18; H, 2.15; N, 9.97. Found: C, 34.30; H, 1.98; N, 10.00; HRMS (*m*/*z*) (ESI): calcd for C_8_H_5_Cl_2_N_2_O_3_S [M – H]^−^, 278.9403; found, 278.9426.

#### Synthesis of 7-Chloro-2-propyl-2*H*-benzo[*e*][1,2,4]thiadiazin-3(4*H*)-one 1,1-Dioxide (**3**)

K_2_CO_3_ (150 mg) was added to a solution of 7-chloro-3-oxo-3,4-dihydro-2*H*-1,2,4-benzothiadiazine 1,1-dioxide (**1**) (460 mg, 2 mmol, 1 equiv) in DMF (15 mL). After 30 min, 1-bromopropane (230 mg, 1 mmol, 0.5 equiv) was added and the solution was stirred overnight at r.t. After the reaction was completed, the mixture was filtered through a glass frit and washed with ethyl acetate. The organic layers were washed with brine, dried with anhydrous MgSO_4_, and evaporated in vacuo. The residue was purified by column chromatography to give the title compound. White solid. Yield 85%. mp [207–211 °C]. ^1^H NMR (600 MHz, DMSO-*d*_6_): δ 7.93 (d, *J* = 2.3 Hz, 1H), 7.77 (dd, *J* = 8.8, 2.4 Hz, 1H), 7.29 (d, *J* = 8.8 Hz, 1H), 3.80–3.69 (m, 2H), 1.65 (h, *J* = 7.4 Hz, 2H), 0.87 (t, *J* = 7.4 Hz, 3H). ^13^C NMR (151 MHz, DMSO): δ 149.90, 135.24, 134.36, 127.39, 123.63, 121.83, 119.87, 43.08, 22.72, 11.38. Anal. Calcd for C_10_H_11_ClN_2_O_3_S: C, 43.72; H, 4.04; N, 10.20. Found: C, 43.56; H, 4.39; N, 10.16; HRMS (*m*/*z*) (ESI): calcd for C_10_H_12_ClN_2_O_3_S [M + H]^+^, 275.0252; found, 275.0274.

#### Synthesis of 7-Chloro-2-(prop-2-yn-1-yl)-2*H*-benzo[*e*][1,2,4]thiadiazin-3(4*H*)-one 1,1-Dioxide (**4**)

K_2_CO_3_ (371 mg, 2.7 mmol, 2.5 equiv) was added to a solution of 7-chloro-3-oxo-3,4-dihydro-2*H*-1,2,4-benzothiadiazine 1,1-dioxide (**1**) (500 mg, 2 mmol, 1 equiv) in DMF (15 mL). After 30 min, 3-bromoprop-1-yne (128 mg, 1 mmol, 0.5 equiv) was added and the solution was stirred overnight at r.t. After the reaction was completed, the mixture was filtered through a glass frit and washed with ethyl acetate. The organic layers were washed with brine, dried with anhydrous MgSO_4_, and evaporated in vacuo. The residue was purified by column chromatography to give product **4**. Pale yellow solid. Yield 93%. mp [251–253 °C]. ^1^H NMR (600 MHz, DMSO-*d*_6_): δ 11.67 (s, 1H), 7.98 (d, *J* = 2.4 Hz, 1H), 7.80 (dd, *J* = 8.8, 2.4 Hz, 1H), 7.31 (d, *J* = 8.8 Hz, 1H), 4.54 (d, *J* = 2.5 Hz, 2H), 3.35 (t, *J* = 2.4 Hz, 1H). ^13^C NMR (151 MHz, DMSO): δ 149.23, 135.49, 133.95, 127.80, 123.99, 121.85, 119.95, 78.92, 75.49, 30.64. Anal. Calcd for C_10_H_7_ClN_2_O_3_S: C, 44.37; H, 2.61; N, 10.35. Found: C, 44.21; H, 2.87; N, 10.31; HRMS (*m*/*z*) (ESI): calcd for C_10_H_8_ClN_2_O_3_S [M + H]^+^, 270.9939; found, 271.0016.

#### Synthesis of 2-Butyl-7-chloro-2*H*-benzo[*e*][1,2,4]thiadiazin-3(4*H*)-one 1,1-Dioxide (**5**)

K_2_CO_3_ (445 mg, 3.22 mmol, 2.5 equiv) was added to a solution of 7-chloro-3-oxo-3,4-dihydro-2*H*-1,2,4-benzothiadiazine 1,1-dioxide (**I**) (300 mg, 1.29 mmol, 1 equiv) in DMF (15 mL). After 30 min, 1-bromobutane (88 mg, 0.64 mmol, 0.5 equiv) was added and the solution was stirred overnight at r.t. After the reaction was completed, the mixture was filtered through a glass filter and washed with ethyl acetate. The organic layers were washed with brine, dried with anhydrous MgSO_4_, and evaporated in vacuo. The residue was purified by column chromatography to give product **5**. White solid. Yield 72%. mp [231–234 °C]. ^1^H NMR (600 MHz, DMSO-*d*_6_): δ 7.93 (d, *J* = 2.4 Hz, 1H), 7.77 (dd, *J* = 8.8, 2.4 Hz, 1H), 7.29 (d, *J* = 8.8 Hz, 1H), 3.81–3.72 (m, 2H), 1.68–1.56 (m, 2H), 1.29 (h, *J* = 7.4 Hz, 2H), 0.88 (t, *J* = 7.4 Hz, 3H). ^13^C NMR (151 MHz, DMSO): δ 149.83, 135.26, 134.21, 127.47, 123.63, 121.83, 119.80, 41.30, 31.47, 19.74, 13.94. Anal. Calcd for C_11_H_13_ClN_2_O_3_S: C, 45.76; H, 4.54; N, 9.70. Found: C, 45.60; H, 4.87; N, 9.67; HRMS (*m*/*z*) (ESI): calcd for C_11_H_14_ClN_2_O_3_S [M + H]^+^, 289.0408; found, 289.0423.

#### Synthesis of 3-(Dimethylamino)-2*H*-benzo[*e*][1,2,4]thiadiazine 1,1-Dioxide (**6**)

A solution of aniline (930 mg, 10 mmol, 1 equiv) in anhydrous DCM (40 mL) was added to a solution of *N*′-(chlorosulfonyl)-*N*,*N*-dimethylcarbamimidoyl chloride (2.1 g, 10 mmol, freshly prepared, 1 equiv) in anhydrous DCM (50 mL), and the mixture was stirred at r.t. for 30 min. The obtained solution was vigorously stirred and treated by the addition of diisopropylethylamine (2.84 g, 22 mmol, 2.2 equiv) solution in anhydrous DCM (10 mL), and the mixture was then stirred at r.t. for 1.5 h. The solvent was removed in vacuo and the residue was treated with cold 5% HCl solution (100 mL). The precipitate was filtered off, washed with water (5 × 30 mL), and dried. Yellow solid. Yield 43%. mp [321 °C with dec]. ^1^H NMR (400 MHz, DMSO): δ 7.63 (dd, *J* = 7.8, 1.2 Hz, 1H), 7.53 (ddd, *J* = 8.6, 7.3, 1.5 Hz, 1H), 7.4–7.42 (m, 1H), 7.28–7.22 (m, 1H), 3.07 (s, 6H). ^13^C NMR (101 MHz, DMSO): δ 151.16, 136.01, 132.05, 124.01, 122.93, 122.42, 117.47, 37.47. Anal. Calcd for C_9_H_11_N_3_O_2_S: C, 47.99; H, 4.92; N, 18.65. Found: C, 48.20; H, 4.75; N, 18.70; HRMS (*m*/*z*) (ESI): calcd for C_9_H_10_N_3_O_2_S [M – H]^−^, 224.0499; found, 224.0513.

#### Synthesis of 3-Amino-6,7-dichloro-2*H*-benzo[*e*][1,2,4]thiadiazine 1,1-Dioxide (**7**)

To a solution of 5-aminotetrazole monohydrate (8.5 g, 0.100 mol, 1 equiv) in 25 mL of THF and 10 mL of water was added 4,5-dichloro-2-nitrobenzenesulfonyl chloride (15 g, 50 mmol, 0.5 equiv). The solution was diluted with water and then evaporated in vacuo, the residue was partitioned between DC and water and the organic layer was dried and concentrated. To a solution of the obtained intermediate in 25 mL of 10% aqueous potassium hydroxide was added in portion sodium dithionite (10 g, 57 mmol, 1.1 equiv) at 40 °C over 1 h. Thereafter, the solution was acidified with HCl and heated at 60 °C for 5 h. After cooling at room temperature, the resulting precipitate was collected, washed with water, and dried to give the compound **7**. Yellow solid. Yield 53%. mp [310–312 °C]. ^1^H NMR (400 MHz, DMSO-*d*_6_): δ 10.91 (br s, 1H), 7.88 (s, 1H), 7.45 (s, 1H), 7.28 (br s, 2H). ^13^C NMR (101 MHz, DMSO): δ 152.04, 135.57, 134.66, 125.56, 124.43, 122.45, 118.51. Anal. Calcd for C_7_H_5_Cl_2_N_3_O_2_S: C, 31.60; H, 1.89; N, 15.79. Found: C, 31.72; H, 1.74; N, 15.85; HRMS (*m*/*z*) (ESI): calcd for C_7_H_4_Cl_2_N_3_O_2_S [M – H]^−^, 263.9407; found, 263.9387.

#### Synthesis of Ethyl 2-Oxo-2-((2-sulfamoylphenyl)amino)acetate (**II**)

To a solution of 2-aminobenzenesulfonamide (1 g, 5.806 mmol, 1 equiv) in THF chilled in an ice–water bath triethylamine (0.81 mL, 5.806 mmol, 1 equiv) was added, followed by slow dropwise addition of ethyl chlorooxalate (0.649 mL, 5.806 mmol, 1 equiv) over 5–10 min. The mixture was allowed to slowly warm to 20 °C and reacted for 5 h. The precipitate was removed by filtration, and the concentrated filtrate was recrystallized from EtOAc to give 0.98 g (62% yield) of the title compound. ^1^H NMR (400 MHz, chloroform-*d*): δ 9.78 (s, 1H), 8.28 (dd, *J* = 7.5, 2.0 Hz, 1H), 8.07 (dd, *J* = 7.4, 2.1 Hz, 1H), 7.63–7.53 (m, 1H), 7.33–7.19 (m, 1H), 3.30 (q, *J* = 8.0 Hz, 2H), 0.94 (t, *J* = 8.0 Hz, 3H).

#### Synthesis of Ethyl 2*H*-1,2,4-Benzothiadiazine-3-carboxylate 1,1-Dioxide (**III**)

To a flask containing anhydrous ethanol (25 mL) was added NaH (60% suspension in mineral oil; 123 mg, 3.085 mmol, 1.05 equiv). The mixture was stirred for 15 min, and **II** (800 mg, 2.938 mmol, 1.00 equiv) was added in one portion. The mixture was stirred for 2 h, at which time TLC analysis showed complete consumption of starting material. Water (50 mL) was added, the pH was adjusted to 3–4 (4 N HCl), and the ethanol was removed on the rotary evaporator. The precipitate was collected by vacuum filtration, washed with water, and dried to constant weight to afford 0.507 g (68% yield) of the title compound. ^1^H NMR (400 MHz, chloroform-*d*): δ 7.80 (dd, *J* = 7.4, 1.7 Hz, 1H), 7.74–7.60 (m, 2H), 7.54–7.42 (m, 1H), 4.18 (q, *J* = 8.0 Hz, 2H), 1.29 (t, *J* = 8.0 Hz, 3H).

#### Synthesis of 3-(Hydroxymethyl)-2*H*-benzo[*e*][1,2,4]thiadiazine 1,1-Dioxide (**IV**)

To a solution of the **III** (400 mg, 1.884 mmol, 1 equiv) in THF (15 mL) at −78 °C was added dropwise LiBH_4_ (82 mg, 3.769 mmol, 2 equiv). The reaction mixture was stirred at −78 °C for 5 min, after which time it was quenched with sat aq NH_4_Cl. The mixture was filtered and the filtrate was extracted with EtOAc (3 × 30 mL). The combined organics were dried and concentrated to provide 220 mg (55% yield) of the titled compound as a yellow solid. ^1^H NMR (400 MHz, chloroform-*d*): δ 8.01 (dd, *J* = 7.5, 2.1 Hz, 1H), 7.83–7.67 (m, 2H), 7.48 (td, *J* = 7.4, 2.1 Hz, 1H), 4.47 (s, 2H).

#### Synthesis of 3-(Chloromethyl)-2*H*-benzo[*e*][1,2,4]thiadiazine 1,1-Dioxide (**8**)

**IV** (0.5 g, 2.60 mmol) was dissolved in thionyl chloride (10 mL) and the reaction mixture was stirred overnight at 60 °C. Thionyl chloride was evaporated and the solid residue triturated over dry toluene, ACN, and filtered to afford the title product as a dark yellow solid (0.623 g, 90% yield). mp [233 °C with dec]. ^1^H NMR (600 MHz, DMSO): δ 7.70 (dd, *J* = 8.0, 1.2 Hz, 1H), 7.59–7.55 (m, 1H), 7.38–7.32 (m, 1H), 7.26 (dd, *J* = 8.3, 0.5 Hz, 1H), 4.37 (s, 2H). ^13^C NMR (151 MHz, DMSO): δ 155.24, 135.29, 133.89, 127.41, 124.03, 121.63, 118.24, 43.69. Anal. Calcd for C_8_H_7_ClN_2_O_2_S: C, 41.66; H, 3.06; Cl, 15.37; N, 12.14. Found: C, 41.84; H, 3.00; N, 12.10; HRMS (*m*/*z*) (ESI): calcd for C_8_H_6_ClN_2_O_2_S [M – H]^−^, 228.9844; found, 228.9836.

### General Procedure for the Synthesis of the Sulfonamides (**9–15**, **V–XI**)

A solution of the appropriate aromatic amine (1 equiv) in NaOH 0.3N (15 mL) was stirred for 10 min at r.t. Subsequently, the respective benzenesulfonyl chloride derivative (1.2 equiv) was added dropwise at 40 °C and the pH of reaction was adjusting using NaOH 2N for returning at starting pH. At a stable pH, the reaction was heated at 80 °C for 1 h. Then, the reaction was cooled at r.t. and the precipitate obtained was filtered, washing with water, and dried. The desiderate compound was crystallized in ethyl ether to obtain the pure compound.

#### 4-Nitro-*N*-phenylbenzenesulfonamide (**9**)

To a solution of aniline (8.1 mmol, 1 equiv) in pyridine (40.5 mL, 0.2 M) was added 4-nitrobenzenesulfonyl chloride (8.9 mmol, 1.1 equiv) at 0 °C. After being stirred at 25 °C for 2–3 h, pyridine was removed by rotary evaporator and the reaction mixture was poured into water. The product was extracted with CH_2_Cl_2_ (three times), dried over MgSO_4_, and concentrated in vacuo. The residue was purified by column chromatography on silica gel to give the corresponding product **9**. Yield 47%. mp [169–172 °C]. ^1^H NMR (400 MHz, methanol-*d*_4_): δ 8.39–8.25 (m, 2H), 8.01–7.92 (m, 2H), 7.25 (tt, *J* = 7.5, 2.1 Hz, 2H), 7.17–7.03 (m, 3H). ^13^C NMR (101 MHz, MeOD): δ 151.57, 146.73, 138.36, 130.34, 129.63, 126.36, 125.21, 122.80. Anal. Calcd for C_12_H_10_N_2_O_4_S: C, 51.79; H, 3.62; N, 10.07. Found: C, 51.61; H, 3.97; N, 10.03; HRMS (*m*/*z*) (ESI): calcd for C_12_H_11_N_2_O_4_S [M + H]^+^, 279.0434; found, 279.0457.

#### *N*-(4,6-Diaminopyrimidin-2-yl)-4-nitrobenzenesulfonamide (**10**)

To a solution of pyrimidine-2,4,6-triamine (8.1 mmol, 1 equiv) in pyridine (40.5 mL, 0.2 M) was added 4-nitrobenzenesulfonyl chloride (8.9 mmol, 1.1 equiv) at 0 °C. After being stirred at 25 °C for 2–3 h, pyridine was removed by rotary evaporator and the reaction mixture was poured into water. The product was extracted with CH_2_Cl_2_ (three times), dried over MgSO_4_, and concentrated in vacuo. The residue was purified by column chromatography on silica gel to give the corresponding product **10**. White solid. Yield 87%. mp [262–263 °C]. ^1^H NMR (600 MHz, DMSO-*d*_6_): δ 10.79 (br s, 1H), 8.20 (d, *J* = 8.7 Hz, 2H), 7.84 (d, *J* = 8.8 Hz, 2H), 7.16 (br s, 2H), 7.02 (br s, 2H), 5.04 (s, 1H). ^13^C NMR (151 MHz, DMSO): δ 154.64, 154.44, 147.75, 140.97, 127.38, 123.81, 73.30. Anal. Calcd for C_10_H_10_N_6_O_4_S: C, 38.71; H, 3.25; N, 27.09. Found: C, 38.58; H, 3.56; N, 27.10; HRMS (*m*/*z*) (ESI): calcd for C_10_H_11_N_6_O_4_S [M + H]^+^, 311.0557; found, 311.0542.

#### *N*-(2,4-Dioxo-1,2,3,4-tetrahydropyrimidin-5-yl)-2-nitrobenzenesulfonamide (**11**)

2-nitrobenzenesulfonyl chloride (0.23 g, 1.18 mmol) was added to a solution of 5-amino uracil (0.15 g, 1.18 mmol) in pyridine (5 mL) at room temperature. The reaction was stirred for 2.5 h, was concentrated in vacuo to give a solid residue. The product was purified by HPLC on a C-18 column eluting with an acetonitrile: water: TFA gradient to give the title compound. Yellow solid. Yield 63%. mp [278–279 °C]. ^1^H NMR (600 MHz, DMSO-*d*_6_): δ 11.23 (s, 1H), 11.01 (br s, 1H), 9.74 (br s, 1H), 8.10–8.04 (m, 1H), 7.91–7.86 (m, 1H), 7.85–7.80 (m, 2H), 7.41 (s, 1H). ^13^C NMR (151 MHz, DMSO): δ 161.87, 150.97, 148.01, 141.04, 134.69, 133.15, 132.67, 130.80, 124.27, 109.73. Anal. Calcd for C_10_H_8_N_4_O_6_S: C, 38.47; H, 2.58; N, 17.94. Found: C, 38.59; H, 2.27; N, 18.00; HRMS (*m*/*z*) (ESI): calcd for C_10_H_7_N_4_O_6_S [M – H]^−^, 311.0092; found, 311.0102.

#### *N*-(1,3-Dimethyl-2,4-dioxo-1,2,3,4-tetrahydropyrimidin-5-yl)-2-nitrobenzenesulfonamide (**12**)

To a solution of 5-amino-1,3-dimethylpyrimidine-2,4(1*H*,3*H*)-dione (8.1 mmol, 1 equiv) in pyridine (40.5 mL, 0.2 M) was added 2-nitrobenzenesulfonyl chloride (8.9 mmol, 1.1 equiv) at 0 °C. After being stirred at 25 °C for 2–3 h, pyridine was removed by a rotary evaporator, and the reaction mixture was poured into water. The product was extracted with CH_2_Cl_2_ (three times), dried over MgSO_4_, and concentrated in vacuo. The residue was purified by column chromatography on silica gel to give the corresponding product **12**. Yellow solid. Yield 69%. mp [236–238 °C]. ^1^H NMR (400 MHz, DMSO): δ 8.11 (dd, *J* = 7.8, 1.3 Hz, 1H), 8.04 (d, *J* = 4.0 Hz, 1H), 7.95–7.82 (m, 3H), 3.22 (s, 3H), 2.54 (dt, *J* = 3.6, 1.8 Hz, 3H). ^13^C NMR (101 MHz, DMSO): δ 160.73, 150.53, 148.20, 147.72, 134.63, 131.96, 130.58, 130.21, 123.78, 112.57, 37.56, 35.59. Anal. Calcd for C_12_H_12_N_4_O_6_S: C, 42.35; H, 3.55; N, 16.46. Found: C, 42.48; H, 3.27; N, 16.51; HRMS (*m*/*z*) (ESI): calcd for C_12_H_12_N_4_O_6_S [M – H]^−^, 339.0405; found, 339,0423.

#### 4-Amino-*N*-methyl-*N*-(pyrimidin-2-yl)benzenesulfonamide (**13**)

To a solution of *N*-methylpyrimidin-2-amine (8.1 mmol, 1 equiv) in pyridine (40.5 mL, 0.2 M) 4-aminobenzenesulfonyl chloride (8.9 mmol, 1.1 equiv) was added at 0 °C. After being stirred at 25 °C for 2–3 h, pyridine was removed by rotary evaporator and the reaction mixture was poured into water. The product was extracted with CH_2_Cl_2_ (three times), dried over MgSO_4_, and concentrated in vacuo. The residue was purified by column chromatography on silica gel to give the titled compound. White solid. Yield 54%. mp [>325 °C]. ^1^H NMR (400 MHz, DMSO-*d*_6_): δ 8.58 (dd, *J* = 4.3, 2.4 Hz, 1H), 8.39 (dd, *J* = 6.5, 2.4 Hz, 1H), 7.60–7.49 (m, 2H), 6.73 (dd, *J* = 6.4, 4.3 Hz, 1H), 6.56–6.45 (m, 2H), 5.64 (s, 2H), 3.55 (s, 3H). ^13^C NMR (101 MHz, DMSO): δ 163.40, 154.21, 151.31, 150.91, 129.64, 128.76, 111.90, 107.20, 41.22. Anal. Calcd for C_11_H_13_N_4_O_2_S: C, 49.80; H, 4.94; N, 21.12. Found: C, 49.68; H, 4.77; N, 21.09; HRMS (*m*/*z*) (ESI): calcd for C_11_H_13_N_4_O_2_S [M + H]^+^, 265.0754; found, 265.0737.

#### 4-Amino-*N*-(4,6-dioxo-1,4,5,6-tetrahydropyrimidin-2-yl)benzenesulfonamide (**14**)

White solid. Yield 72%. mp [199–201 °C]. ^1^H NMR (400 MHz, methanol-*d*_4_): δ 7.78–7.70 (m, 2H), 6.68 (s, 1H), 6.66–6.60 (m, 2H). ^13^C NMR (101 MHz, MeOD): δ 169.45, 158.15, 154.44, 131.68, 127.13, 114.93, 113.65, 23.44. Anal. Calcd for C_10_H_10_N_4_O_4_S: C, 42.55; H, 3.57; N, 19.85. Found: C, 42.40; H, 3.67; N, 19.78; HRMS (*m*/*z*) (ESI): calcd for C_10_H_11_N_4_O_4_S [M + H]^+^, 283.0496; found, 283.0513.

#### 4-Amino-*N*-(4,6-diaminopyrimidin-2-yl)benzenesulfonamide (**15**)

White solid. Yield 46%. mp [266–268 °C]. ^1^H NMR (400 MHz, methanol-*d*_4_): δ 7.67–7.59 (m, 2H), 6.62–6.55 (m, 2H), 5.26 (s, 1H). ^13^C NMR (100 MHz, methanol-*d*_4_): δ 163.81, 163.65, 156.27, 153.02, 129.83, 126.22, 112.35, 77.55. Anal. Calcd for C_10_H_12_N_6_O_2_S: C, 42.85; H, 4.32; N, 29.98. Found: C, 42.80; H, 4.42; N, 30.05; HRMS (*m*/*z*) (ESI): calcd for C_10_H_13_N_6_O_2_S [M + H]^+^, 281.0815; found, 281.0820.

#### *N*-(4-Amino-2-mercapto-6-oxo-1,6-dihydropyrimidin-5-yl)benzenesulfonamide (**V**)

White solid. Yield 88%. ^1^H NMR (400 MHz, chloroform-*d*): δ 7.84–7.73 (m, 2H), 7.69–7.42 (m, 3H).

#### 2-Amino-*N*-(4-amino-2-mercapto-6-oxo-1,6-dihydropyrimidin-5-yl)benzenesulfonamide (**VI**)

Yellow solid. Yield 68%. ^1^H NMR (400 MHz, chloroform-*d*): δ 7.67 (dd, *J* = 7.5, 2.0 Hz, 1H), 7.26 (td, *J* = 7.5, 2.0 Hz, 1H), 6.97–6.71 (m, 2H).

#### 3-Amino-*N*-(4-amino-2-(hexylthio)-6-oxo-1,6-dihydropyrimidin-5-yl)benzenesulfonamide (**VII**)

Yellow solid. Yield 75%. ^1^H NMR (400 MHz, chloroform-*d*): δ 7.27–7.15 (m, 2H), 7.12 (t, *J* = 1.8 Hz, 1H), 6.90–6.70 (m, 1H).

#### *N*-(4-Amino-2-mercapto-6-oxo-1,6-dihydropyrimidin-5-yl)-3-nitrobenzenesulfonamide (**IX**)

Orange solid. Yield 88%. ^1^H NMR (400 MHz, DMSO-*d*_6_): δ 11.72 (d, *J* = 1.8 Hz, 1H), 11.66 (d, *J* = 2.0 Hz, 1H), 9.24 (s, 1H), 8.55 (t, *J* = 2.0 Hz, 1H), 8.46–8.40 (m, 1H), 8.16 (dt, *J* = 7.8, 1.3 Hz, 1H), 7.79 (t, *J* = 8.0 Hz, 1H), 6.47 (s, 2H).

#### *N*-(4-(*N*-(6-Amino-2-mercapto-4-oxo-4,5-dihydropyrimidin-5-yl)sulfamoyl)phenyl)acetamide (**XI**)

Following general procedure A. White solid. Yield 74%. ^1^H NMR (400 MHz, chloroform-*d*): δ 7.71–7.47 (m, 4H), 5.63 (s, 1H), 2.10 (s, 3H).

### General Procedure for the Synthesis of the Bissulfonamides (**16–18**)

The same procedure described for the synthesis of the sulfonamides **9–15**, **V–XI**, was adopted, with the sole exception of using 2.5 equiv of the appropriate arylsulfonyl chloride and longer reaction time (from 6 to 12 h).

#### *N*-(4,6-Diaminopyrimidin-2-yl)-4-nitro-*N*-((4-nitrophenyl)sulfonyl)benzenesulfonamide (**16**)

Pale yellow solid. Yield 41%. mp [259–260 °C]. ^1^H NMR (400 MHz, methanol-*d*_4_): δ 8.34–8.27 (m, 2H), 8.04–7.96 (m, 2H). ^13^C NMR (100 MHz, methanol-*d*_4_): δ 163.46, 162.61, 162.12, 148.78, 140.99, 128.41, 124.35, 97.85. Anal. Calcd for C_16_H_13_N_7_O_8_S_2_: C, 38.79; H, 2.64; N, 19.79. Found: C, 38.82; H, 2.57; N, 19.75; HRMS (*m*/*z*) (ESI): calcd for C_16_H_14_N_7_O_8_S_2_^+^ [M + H]^+^, 496.0340; found, 496.0342.

#### 4-Nitro-*N*-((4-nitrophenyl)sulfonyl)-*N*-(2,4,6-triaminopyrimidin-5-yl)benzenesulfonamide (**17**)

Pale yellow solid. Yield 35%. mp [255–257 °C]. ^1^H NMR (400 MHz, DMSO-*d*_6_): δ 8.36–8.29 (m, 4H), 8.02–7.95 (m, 4H), 6.60 (br s, 4H), 5.29 (s, 1H). ^13^C NMR (100 MHz, methanol-*d*_4_): δ 165.38, 165.12, 153.22, 148.78, 138.98, 128.31, 124.35, 77.58. Anal. Calcd for C_16_H_14_N_8_O_8_S_2_: C, 37.65; H, 2.75; N, 21.95. Found: C, 37.60; H, 2.82; N, 21.92; HRMS (*m*/*z*) (ESI): calcd for C_16_H_14_N_8_O_8_S_2_^+^ [M + H]^+^, 511.0449; found, 511.0451.

#### *N*,*N*′-(((Hydrosulfonyl(2,4,6-triaminopyrimidin-5-yl)amino)sulfonyl)bis(4,1-phenylene))diacetamide (**18**)

Yellow solid. Yield 29%. mp [260–262 °C]. 1H NMR (400 MHz, methanol-*d*_4_): δ 7.82–7.81 (m, 4H), 7.73–7.71 (m, 4H), 2.16 (s, 6H). ^13^C NMR (100 MHz, methanol-*d*_4_): δ 169.06, 163.30, 162.87, 162.12, 142.62, 134.69, 128.62, 119.77, 97.85, 24.09. Anal. Calcd for C_20_H_22_N_8_O_6_S_2_: C, 44.94; H, 4.14; N, 20.96. Found: C, 45.05; H, 4.10; N, 20.95; HRMS (*m*/*z*) (ESI): calcd for C_20_H_23_N_8_O_6_S_2_^+^ [M + H]^+^, 535.1176; found, 535.1175.

### General Procedure for the Synthesis of S-Substituted-*N*-(5-Pyrimidinyl)benzenesulfonamide Derivatives (**19–37**)

To a suspension of **V**–**XI** (1 equiv) and K_2_CO_3_ (1.1 equiv) in DMA (3 mL) was slowly added the respective bromo derivate (1.2 equiv) and the reaction was heated at 45 °C for 1 h. After standing for a few hour at r.t, the mixture was decomposed with ice and acidified with glacial acid to pH 5. The pure compound was obtained as precipitate that was filtered and washed with water.

#### *N*-(4-Amino-2-(benzylthio)-6-oxo-1,6-dihydropyrimidin-5-yl)benzenesulfonamide (**19**)

Light yellow solid. Yield 59%. mp [248 °C with dec]. ^1^H NMR (400 MHz, DMSO-*d*_6_): δ 11.80 (br s, 1H), 8.74 (br s, 1H), 7.80–7.74 (m, 2H), 7.60–7.52 (m, 1H), 7.50–7.39 (m, 4H), 7.35–7.28 (m, 2H), 7.28–7.22 (m, 1H), 6.46 (br s, 2H), 4.33 (s, 2H). ^13^C NMR (101 MHz, DMSO): δ 160.68, 140.90, 137.49, 132.15, 129.16, 128.37, 128.33, 127.22, 127.13, 92.49, 33.45. Anal. Calcd for C_17_H_16_N_4_O_3_S_2_: C, 52.56; H, 4.15; N, 14.42. Found: C, 52.70; H, 3.98; N, 14.46; HRMS (*m*/*z*) (ESI): calcd for C_17_H_15_N_4_O_3_S_2_ [M – H]^−^, 387.0591; found, 387.0574.

#### 3-Amino-*N*-(4-amino-2-(methylthio)-6-oxo-1,6-dihydropyrimidin-5-yl)benzenesulfonamide (**20**)

White solid. Yield 36%. mp [246–248 °C]. ^1^H NMR (400 MHz, DMSO-*d*_6_): δ 11.84 (s, 1H), 8.45 (s, 1H), 7.09 (t, *J* = 7.9 Hz, 1H), 6.97 (t, *J* = 2.0 Hz, 1H), 6.89 (ddd, *J* = 7.8, 1.9, 1.0 Hz, 1H), 6.68 (ddd, *J* = 8.1, 2.3, 1.0 Hz, 1H), 6.20 (s, 2H), 5.43 (s, 2H), 2.43 (s, 3H). ^13^C NMR (101 MHz, DMSO): δ 148.70, 141.40, 128.84, 117.14, 113.98, 111.77, 92.93, 40.13, 39.92, 39.71, 39.50, 39.29, 39.08, 38.88, 12.69. Anal. Calcd for C_11_H_13_N_5_O_3_S_2_: C, 40.36; H, 4.00; N, 21.39. Found: C, 40.30; H, 4.10; N, 21.45; HRMS (*m*/*z*) (ESI): calcd for C_11_H_14_N_5_O_3_S_2_ [M + H]^+^, 328.0533; found, 328.0535.

#### 2-Amino-*N*-(4-amino-2-(ethylthio)-6-oxo-1,6-dihydropyrimidin-5-yl)benzenesulfonamide (**21**)

White solid. Yield 68%. mp [251–253 °C]. ^1^H NMR (400 MHz, methanol-*d*_4_): δ 7.57 (dd, *J* = 8.1, 1.6 Hz, 1H), 7.26 (ddd, *J* = 8.5, 7.1, 1.6 Hz, 1H), 6.80 (dd, *J* = 8.3, 1.2 Hz, 1H), 6.63 (ddd, *J* = 8.2, 7.2, 1.2 Hz, 1H), 3.13 (q, *J* = 7.3 Hz, 2H), 1.35 (t, *J* = 7.3 Hz, 3H). ^13^C NMR (100 MHz, methanol-*d*_4_): δ 161.21, 160.34, 149.07, 141.45, 131.32, 129.42, 121.50, 120.30, 118.00, 99.60, 23.81, 14.37. Anal. Calcd for C_12_H_15_N_5_O_3_S_2_: C, 42.22; H, 4.43; N, 20.51. Found: C, 42.20; H, 4.45; N, 20.45; HRMS (*m*/*z*) (ESI): calcd for C_12_H_16_N_5_O_3_S_2_ [M + H]^+^, 342.0689; found, 342.0691.

#### 2-Amino-*N*-(4-amino-2-(butylthio)-6-oxo-1,6-dihydropyrimidin-5-yl)benzenesulfonamide (**22**)

White solid. Yield 76%. mp [239 °C with dec]. ^1^H NMR (400 MHz, DMSO-*d*_6_): δ 11.86 (br s, 1H), 8.49 (br s, 1H), 7.45 (dd, *J* = 8.0, 1.6 Hz, 1H), 7.21 (ddd, *J* = 8.5, 7.0, 1.6 Hz, 1H), 6.75 (dd, *J* = 8.3, 1.1 Hz, 1H), 6.51 (ddd, *J* = 8.1, 7.1, 1.2 Hz, 1H), 6.16 (br s, 4H), 3.06 (t, *J* = 7.3 Hz, 2H), 1.69–1.50 (m, 2H), 1.46–1.29 (m, 2H), 0.89 (t, *J* = 7.3 Hz, 3H). ^13^C NMR (101 MHz, DMSO): δ 160.62, 147.02, 133.53, 129.37, 120.50, 116.77, 114.37, 92.06, 30.81, 29.29, 21.27, 13.45. Anal. Calcd for C_14_H_19_N_5_O_3_S_2_: C, 45.51; H, 5.18; N, 18.96. Found: C, 45.59; H, 5.36; N, 18.90; HRMS (*m*/*z*) (ESI): calcd for C_14_H_20_N_5_O_3_S_2_ [M + H]^+^, 370.1002; found, 370.1035.

#### 2-Amino-*N*-(4-amino-6-oxo-2-((3-phenylpropyl)thio)-1,6-dihydropyrimidin-5-yl)benzenesulfonamide (**23**)

White solid. Yield 48%. mp [269–271 °C]. ^1^H NMR (400 MHz, methanol-*d*_4_): δ 8.00 (s, 1H), 7.56 (dd, *J* = 8.0, 1.6 Hz, 1H), 7.30 (s, 1H), 7.33–7.14 (m, 6H), 6.80 (dd, *J* = 8.3, 1.1 Hz, 1H), 6.62 (ddd, *J* = 8.2, 7.1, 1.2 Hz, 1H), 3.13 (t, *J* = 7.2 Hz, 2H), 3.01 (s, 4H), 2.88 (d, *J* = 0.8 Hz, 5H), 2.74 (t, *J* = 7.6 Hz, 2H), 2.06–1.94 (m, 2H). ^13^C NMR (101 MHz, MeOD): δ 164.88, 148.40, 142.62, 135.22, 131.05, 129.57, 129.47, 127.04, 122.12, 118.52, 116.96, 49.65, 49.44, 49.23, 49.01, 48.80, 48.59, 48.37, 36.95, 35.62, 32.24, 31.66, 30.73. Anal. Calcd for C_19_H_21_N_5_O_3_S_2_: C, 52.88; H, 4.91; N, 16.23. Found: C, 52.90; H, 4.90; N, 16.25; HRMS (*m*/*z*) (ESI): calcd for C_19_H_22_N_5_O_3_S_2_ [M + H]^+^, 432.1159; found, 432.1160.

#### 3-Amino-*N*-(4-amino-2-(methylthio)-6-oxo-1,6-dihydropyrimidin-5-yl)-*N*-methylbenzenesulfonamide (**24**)

White solid. Yield 87%. mp [261–264 °C]. ^1^H NMR (400 MHz, DMSO-*d*_6_): δ 8.63 (s, 1H), 7.26 (t, *J* = 8.0 Hz, 1H), 7.12 (t, *J* = 2.2 Hz, 1H), 7.03 (d, *J* = 7.6 Hz, 1H), 6.89 (dd, *J* = 8.3, 2.6 Hz, 1H), 6.25 (s, 2H), 3.17 (s, 3H), 2.93 (d, *J* = 2.6 Hz, 6H). ^13^C NMR (101 MHz, DMSO): δ 160.13, 158.99, 158.72, 149.97, 141.73, 128.90, 115.72, 114.04, 109.97, 92.52, 40.13, 39.92, 39.87, 39.71, 39.50, 39.29, 39.08, 38.88, 29.46, 14.25. Anal. Calcd for C_12_H_15_N_5_O_3_S_2_: C, 42.22; H, 4.43; N, 20.51. Found: C, 42.20; H, 4.40; N, 20.57; HRMS (*m*/*z*) (ESI): calcd for C_12_H_16_N_5_O_3_S_2_ [M + H]^+^, 342.4115; found, 342.4110.

#### 3-Amino-*N*-(4-amino-2-(ethylthio)-6-oxo-1,6-dihydropyrimidin-5-yl)benzenesulfonamide (**25**)

White solid. Yield 76%. mp [240–242 °C]. ^1^H NMR (400 MHz, DMSO-*d*_6_): δ 11.78 (br s, 1H), 8.45 (br s, 1H), 7.09 (t, *J* = 7.9 Hz, 1H), 6.97 (t, *J* = 2.0 Hz, 1H), 6.91–6.88 (m, 1H), 6.68 (ddd, *J* = 8.1, 2.3, 1.0 Hz, 1H), 6.18 (br s, 2H), 5.42 (s, 2H), 3.04 (q, *J* = 7.3 Hz, 2H), 1.26 (t, *J* = 7.3 Hz, 3H). ^13^C NMR (101 MHz, DMSO): δ 162.87, 148.69, 141.42, 128.83, 117.13, 113.97, 111.75, 92.98, 24.07, 14.56. Anal. Calcd for C_12_H_15_N_5_O_3_S_2_: C, 42.22; H, 4.43; N, 20.51. Found: C, 42.09; H, 4.51; N, 20.45; HRMS (*m*/*z*) (ESI): calcd for C_12_H_16_N_5_O_3_S_2_ [M + H]^+^, 342.0689; found, 342.0654.

#### 3-Amino-*N*-(4-amino-2-(butylthio)-6-oxo-1,6-dihydropyrimidin-5-yl)benzenesulfonamide (**26**)

White solid. Yield 69%. mp [224–227 °C]. ^1^H NMR (400 MHz, DMSO-*d*_6_): δ 11.90 (s, 1H), 7.73 (s, 1H), 7.60 (d, *J* = 7.4 Hz, 1H), 7.46–7.36 (m, 2H), 6.80 (d, *J* = 7.4 Hz, 1H), 6.34 (d, *J* = 7.0 Hz, 1H), 6.22 (d, *J* = 7.1 Hz, 1H), 5.28 (s, 2H), 3.16 (t, *J* = 7.1 Hz, 2H), 1.64 (p, *J* = 7.0 Hz, 2H), 1.43 (h, *J* = 7.6 Hz, 2H), 0.91 (t, *J* = 8.0 Hz, 3H). ^13^C NMR (100 MHz, DMSO-*d*_6_): δ 160.89, 159.72, 148.96, 146.04, 136.01, 129.61, 121.63, 118.52, 112.43, 99.47, 30.52, 29.26, 21.73, 13.36. Anal. Calcd for C_14_H_19_N_5_O_3_S_2_: C, 45.51; H, 5.18; N, 18.96. Found: C, 45.50; H, 5.10; N, 19.02; HRMS (*m*/*z*) (ESI): calcd for C_14_H_20_N_5_O_3_S_2_ [M + H]^+^, 370.1002; found, 370.1005.41.

#### 3-Amino-*N*-(4-amino-2-(hexylthio)-6-oxo-1,6-dihydropyrimidin-5-yl)benzenesulfonamide (**27**)

Yellow solid. Yield 56%. mp [233 °C with dec]. ^1^H NMR (400 MHz, DMSO-*d*_6_): δ 11.79 (br s, 1H), 8.46 (br s, 1H), 7.08 (t, *J* = 7.8 Hz, 1H), 6.98 (s, 1H), 6.89 (d, *J* = 7.6 Hz, 1H), 6.68 (dd, *J* = 8.0, 2.2 Hz, 1H), 6.18 (br s, 2H), 5.42 (s, 2H), 3.05 (t, *J* = 7.2 Hz, 2H), 1.59 (p, *J* = 7.2 Hz, 2H), 1.34 (q, *J* = 6.9, 6.2 Hz, 2H), 1.27 (qd, *J* = 7.6, 5.3, 3.6 Hz, 4H), 0.86 (t, *J* = 6.6 Hz, 3H). ^13^C NMR (101 MHz, DMSO): δ 160.34, 148.69, 141.42, 128.81, 117.14, 113.98, 111.76, 92.97, 30.70, 29.60, 28.70, 27.79, 21.98, 13.85. Anal. Calcd for C_16_H_23_N_5_O_3_S_2_: C, 48.34; H, 5.83; N, 17.62. Found: C, 48.22; H, 6.00; N, 17.57; HRMS (*m*/*z*) (ESI): calcd for C_16_H_24_N_5_O_3_S_2_ [M + H]^+^, 398.1315; found, 398.1336.

#### 3-Amino-*N*-(4-amino-6-oxo-2-((3-phenylpropyl)thio)-1,6-dihydropyrimidin-5-yl)benzenesulfonamide (**28**)

Light orange solid. Yield 76%. mp [218–219 °C]. ^1^H NMR (400 MHz, DMSO-*d*_6_): δ 11.82 (br s, 1H), 8.46 (br s, 1H), 7.32–7.25 (m, 2H), 7.24–7.14 (m, 3H), 7.08 (t, *J* = 7.8 Hz, 1H), 6.98 (t, *J* = 2.0 Hz, 1H), 6.89 (dt, *J* = 7.8, 1.2 Hz, 1H), 6.68 (ddd, *J* = 8.0, 2.3, 1.0 Hz, 1H), 6.16 (br s, 2H), 5.42 (s, 2H), 3.06 (t, *J* = 7.2 Hz, 2H), 2.67 (dd, *J* = 8.8, 6.7 Hz, 2H), 1.91 (tt, *J* = 7.8, 6.2 Hz, 2H). ^13^C NMR (101 MHz, DMSO): δ 160.37, 148.69, 141.42, 141.12, 128.82, 128.31, 128.28, 125.85, 117.13, 113.98, 111.76, 93.01, 34.10, 30.57, 29.26. Anal. Calcd for C_19_H_21_N_5_O_3_S_2_: C, 52.88; H, 4.91; N, 16.23. Found: C, 52.76; H, 5.74; N, 16.19; HRMS (*m*/*z*) (ESI): calcd for C_19_H_21_N_5_O_3_S_2_ [M + H]^+^, 432.1159; found, 432.1142.

#### *N*-(4-Amino-6-oxo-2-(propylthio)-1,6-dihydropyrimidin-5-yl)-2-nitro-*N*-propylbenzenesulfonamide (**29**)

Yellow solid. Yield 63%. mp [241–243 °C]. ^1^H NMR (400 MHz, DMSO-*d*_6_): δ 11.95 (s, 1H), 8.34–8.27 (m, 1H), 7.90–7.83 (m, 1H), 7.77–7.67 (m, 2H), 7.40 (s, 2H), 3.64 (t, *J* = 7.1 Hz, 2H), 3.03 (t, *J* = 7.1 Hz, 2H), 1.67 (qd, *J* = 7.8, 5.4 Hz, 4H), 0.95 (dt, *J* = 10.7, 8.0 Hz, 6H). ^13^C NMR (100 MHz, DMSO-*d*_6_): δ 159.96, 158.08, 151.31, 148.60, 134.12, 134.02, 130.69, 130.55, 125.94, 117.42, 49.55, 31.81, 23.86, 21.79, 14.02, 12.28. Anal. Calcd for C_16_H_21_N_5_O_5_S_2_: C, 44.95; H, 4.95; N, 16.38. Found: C, 44.95; H, 5.02; N, 16.40; HRMS (*m*/*z*) (ESI): calcd for C_16_H_22_N_5_O_5_S [M + H]^+^, 428.1057; found, 428.1060.

#### *N*-(4-Amino-2-(butylthio)-6-oxo-1,6-dihydropyrimidin-5-yl)-3-nitrobenzenesulfonamide (**30**)

Light yellow solid. Yield 71%. mp [240–242 °C]. ^1^H NMR (400 MHz, DMSO-*d*_6_): δ 11.78 (br s, 1H), 9.16 (br s, 1H), 8.56 (t, *J* = 2.0 Hz, 1H), 8.42 (ddd, *J* = 8.3, 2.3, 1.0 Hz, 1H), 8.15 (dt, *J* = 7.9, 1.2 Hz, 1H), 7.78 (t, *J* = 8.0 Hz, 1H), 6.55 (br s, 2H), 3.06 (t, *J* = 7.2 Hz, 2H), 1.58 (tt, *J* = 8.0, 6.5 Hz, 2H), 1.37 (dq, *J* = 14.5, 7.3 Hz, 2H), 0.89 (t, *J* = 7.3 Hz, 3H). ^13^C NMR (101 MHz, DMSO): δ 161.09, 147.23, 142.90, 133.29, 130.23, 126.66, 122.35, 91.52, 30.81, 29.31, 21.26, 13.44. Anal. Calcd for C_14_H_17_N_5_O_5_S_2_: C, 42.10; H, 4.29; N, 17.53. Found: C, 42.20; H, 4.33; N, 17.58; HRMS (*m*/*z*) (ESI): calcd for C_14_H_16_N_5_O_5_S_2_ [M – H]^−^, 398.0598; found, 398.0573.

#### *N*-(4-Amino-2-(hexylthio)-6-oxo-1,6-dihydropyrimidin-5-yl)-3-nitrobenzenesulfonamide (**31**)

Yellow solid. Yield 69%. mp [204–205 °C]. ^1^H NMR (400 MHz, methanol-*d*_4_): δ 8.68 (t, *J* = 2.0 Hz, 1H), 8.43 (ddd, *J* = 8.3, 2.3, 1.0 Hz, 1H), 8.20 (dt, *J* = 7.8, 1.2 Hz, 1H), 7.74 (t, *J* = 8.1 Hz, 1H), 3.16 (t, *J* = 7.3 Hz, 2H), 1.80–1.62 (m, 2H), 1.45 (ddt, *J* = 12.3, 8.9, 6.5 Hz, 2H), 1.39–1.30 (m, 4H), 0.97–0.89 (m, 3H). ^13^C NMR (101 MHz, MeOD): δ 163.33, 161.99, 161.68, 149.42, 143.62, 134.37, 131.08, 128.03, 123.92, 93.47, 32.49, 31.45, 30.32, 29.45, 23.62, 14.35. Anal. Calcd for C_16_H_21_N_5_O_5_S_2_: C, 44.95; H, 4.95; N, 16.38. Found: C, 45.02; H, 4.87; N, 16.42; HRMS (*m*/*z*) (ESI): calcd for C_16_H_21_N_5_O_5_S_2_ [M – H]^−^, 426.0911; found, 426.0903.

#### 2-((4-Amino-5-((3-nitrophenyl)sulfonamido)-6-oxo-1,6-dihydropyrimidin-2-yl)thio)acetic Acid (**32**)

Light yellow solid. Yield 76%. mp [247–251 °C]. ^1^H NMR (600 MHz, DMSO-*d*_6_): δ 12.32 (br s, 1H), 9.21 (s, 1H), 8.57 (t, *J* = 2.0 Hz, 1H), 8.43 (ddd, *J* = 8.3, 2.3, 1.0 Hz, 1H), 8.16 (ddd, *J* = 7.8, 1.7, 1.1 Hz, 1H), 7.79 (t, *J* = 8.0 Hz, 1H), 6.54 (br s, 2H), 3.93 (s, 2H). ^13^C NMR (151 MHz, DMSO): δ 170.02, 161.53, 147.77, 143.43, 133.78, 130.75, 127.19, 122.87, 92.17, 32.94. Anal. Calcd for C_12_H_11_N_5_O_7_S_2_: C, 36.00; H, 2.52; N, 17.49. Found: C, 36.11; H, 2.59; N, 17.43; HRMS (*m*/*z*) (ESI): calcd for C_14_H_20_N_5_O_3_S_2_ [M – H]^−^, 400.0027; found, 400.0012.

#### *N*-(4-Amino-2-(isopentylthio)-6-oxo-1,6-dihydropyrimidin-5-yl)-3-nitrobenzenesulfonamide (**33**)

Light yellow solid. Yield 72%. mp [258 °C with dec]. ^1^H NMR (400 MHz, DMSO-*d*_6_): δ 11.77 (br s, 1H), 9.16 (br s, 1H), 8.55 (t, *J* = 1.9 Hz, 1H), 8.52–8.40 (m, 1H), 8.15 (dd, *J* = 8.0, 1.4 Hz, 1H), 7.78 (td, *J* = 8.1, 1.4 Hz, 1H), 6.53 (br s, 2H), 3.19–2.94 (m, 2H), 1.64 (dq, *J* = 13.3, 6.6 Hz, 1H), 1.56–1.44 (m, 2H), 0.90 (d, *J* = 1.4 Hz, 3H), 0.88 (d, *J* = 1.4 Hz, 3H). ^13^C NMR (101 MHz, DMSO): δ 161.09, 147.23, 142.91, 133.28, 130.23, 126.66, 122.35, 91.55, 37.60, 27.79, 26.89, 22.06. Anal. Calcd for C_15_H_19_N_5_O_5_S_2_: C, 43.57; H, 4.63; N, 16.94. Found: C, 43.68; H, 4.48; N, 16.98; HRMS (*m*/*z*) (ESI): calcd for C_15_H_18_N_5_O_5_S_2_ [M – H]^−^, 412.0755; found, 412.0781.

#### *N*-(4-Amino-6-oxo-2-(phenethylthio)-1,6-dihydropyrimidin-5-yl)-3-nitrobenzenesulfonamide (**34**)

Light yellow solid. Yield 53%. mp [245–246 °C]. ^1^H NMR (400 MHz, DMSO-*d*_6_): δ 10.50 (s, 1H), 9.40 (s, 1H), 8.45 (s, 1H), 8.36 (d, *J* = 7.5 Hz, 1H), 8.00 (d, *J* = 7.4 Hz, 1H), 7.72 (t, *J* = 7.5 Hz, 1H), 7.12 (hept, *J* = 7.1 Hz, 5H), 6.80 (s, 2H), 3.20 (t, *J* = 7.1 Hz, 2H), 2.87 (t, *J* = 7.1 Hz, 2H). ^13^C NMR (100 MHz, DMSO-*d*_6_): δ 165.73, 162.54, 160.77, 146.93, 139.52, 138.49, 130.60, 130.53, 128.50, 127.92, 127.57, 126.04, 122.50, 107.01, 33.40, 32.90. Anal. Calcd for C_18_H_17_N_5_O_5_S_2_: C, 48.31; H, 3.83; N, 15.65. Found: C, 48.31; H, 3.83; N, 15.65; HRMS (*m*/*z*) (ESI): calcd for C_18_H_16_N_5_O_5_S_2_ [M – H]^−^, 446.0598; found, 446.0608.

#### *N*-(4-Amino-6-oxo-2-((3-phenylpropyl)thio)-1,6-dihydropyrimidin-5-yl)-3-nitrobenzenesulfonamide (**35**)

Light yellow solid. Yield 79%. mp [325 °C with dec]. ^1^H NMR (400 MHz, DMSO-*d*_6_): δ 11.81 (br s, 1H), 9.17 (br s, 1H), 8.56 (t, *J* = 2.0 Hz, 1H), 8.41 (ddd, *J* = 8.3, 2.4, 1.0 Hz, 1H), 8.15 (dt, *J* = 7.8, 1.3 Hz, 1H), 7.78 (t, *J* = 8.0 Hz, 1H), 7.33–7.24 (m, 2H), 7.24–7.13 (m, 3H), 6.54 (br s, 2H), 3.06 (t, *J* = 7.2 Hz, 2H), 2.67 (dd, *J* = 8.8, 6.7 Hz, 2H), 1.91 (tt, *J* = 7.8, 6.2 Hz, 2H). ^13^C NMR (101 MHz, DMSO): δ 161.09, 147.24, 142.92, 141.11, 133.29, 130.22, 128.31, 128.28, 126.66, 125.85, 122.36, 91.57, 34.07, 30.56, 29.29. Anal. Calcd for C_19_H_19_N_5_O_5_S_2_: C, 49.45; H, 4.15; N, 15.18. Found: C, 49.56; H, 4,12; N, 15.21; HRMS (*m*/*z*) (ESI): calcd for C_19_H_18_N_5_O_5_S_2_ [M – H]^−^, 460.0755; found, 460.0769.

#### *N*-(4-Amino-2-(*sec*-butylthio)-6-oxo-1,6-dihydropyrimidin-5-yl)-4-nitrobenzenesulfonamide (**36**)

Light yellow solid. Yield 48%. mp [241 °C with dec]. ^1^H NMR (600 MHz, DMSO-*d*_6_): δ 11.76 (s, 1H), 9.13 (s, 1H), 8.31 (d, *J* = 8.8 Hz, 2H), 8.01 (d, *J* = 8.9 Hz, 2H), 6.49 (s, 3H), 1.63 (pd, *J* = 7.1, 3.9 Hz, 2H), 1.31 (d, *J* = 6.9 Hz, 3H), 0.94 (t, *J* = 7.4 Hz, 4H). ^13^C NMR (151 MHz, DMSO): δ 161.59, 159.28, 149.78, 129.31, 124.12, 42.24, 40.53, 40.41, 40.39, 40.27, 40.13, 39.99, 39.85, 39.71, 39.57, 29.28, 20.97, 11.67. Anal. Calcd for C_14_H_17_N_5_O_5_S_2_: C, 42.10; H, 4.29; N, 17.53. Found: C, 42.15; H, 4.35; N, 17.48; HRMS (*m*/*z*) (ESI): calcd for C_14_H_17_N_5_O_5_S_2_ [M + H]^+^, 400.0744; found, 400.0745.

#### *N*-(4-(*N*-(6-Amino-4-oxo-2-((3-phenylpropyl)thio)-4,5-dihydropyrimidin-5-yl)sulfamoyl)phenyl)acetamide (**37**)

Light yellow solid. Yield 51%. mp [234 °C with dec]. ^1^H NMR (400 MHz, chloroform-*d*): δ 7.86–7.78 (m, 1H), 7.72–7.65 (m, 1H), 7.30–7.14 (m, 3H), 3.22 (t, *J* = 7.1 Hz, 1H), 2.68 (tt, *J* = 7.1, 1.0 Hz, 1H), 2.15 (s, 2H), 2.04 (p, *J* = 7.0 Hz, 1H). ^13^C NMR (100 MHz, chloroform-*d*): δ 169.06, 165.01, 162.67, 160.89, 142.16, 140.89, 134.70, 128.70, 128.44, 128.40, 126.15, 119.80, 107.13, 34.50, 31.15, 29.85, 24.10. Anal. Calcd for C_21_H_23_N_5_O_4_S_2_: C, 53.26; H, 4.90; N, 14.79. Found: C, 53.20; H, 4.74; N, 14.75. HRMS (*m*/*z*) (ESI): calcd for C_21_H_23_N_5_O_4_S_2_ [M + H]^+^, 474.1264; found, 474.1264.

### General Procedure for the Synthesis of Poly-Substituted-*N*-(5-Pyrimidinyl)benzenesulfonamide Derivatives (**38–45**)

The same procedure described for the synthesis of the *S*-substituted *N*-(5-pyrimidinyl)benzenesulfonamide derivatives (**19–37**) was adopted using an excess of the appropriate alkyl bromide, higher temperature (80 °C), and longer reaction time (usually overnight).

#### *N*-(4-Amino-6-propoxy-2-(propylthio)pyrimidin-5-yl)-2-nitro-*N*-propylbenzenesulfonamide (**38**)

Light yellow solid. Yield 87%. mp [259 °C with dec]. ^1^H NMR (600 MHz, DMSO-*d*_6_): δ 7.93–7.79 (m, 4H), 6.78 (s, 2H), 3.98 (ddd, *J* = 10.4, 7.8, 6.5 Hz, 1H), 3.72 (ddt, *J* = 13.2, 8.4, 6.5 Hz, 1H), 3.42–3.33 (m, 4H), 3.02–2.91 (m, 2H), 1.67 (dq, *J* = 23.4, 7.3 Hz, 3H), 1.61–1.46 (m, 1H), 1.42–1.30 (m, 1H), 0.96 (t, *J* = 7.3 Hz, 4H), 0.85 (t, *J* = 7.4 Hz, 4H), 0.66 (t, *J* = 7.4 Hz, 3H). ^13^C NMR (100 MHz, DMSO-*d*_6_): δ 169.31, 162.86, 162.77, 148.06, 133.45, 133.29, 131.55, 129.64, 125.16, 110.87, 69.34, 51.64, 33.22, 22.96, 22.49, 21.42, 13.13, 11.49, 10.49. Anal. Calcd for C_19_H_27_N_5_O_5_S_2_: C, 48.60; H, 5.80; N, 14.91. Found: C, 48.65; H, 5.82; N, 17.95. HRMS (*m*/*z*) (ESI): calcd for C_19_H_28_N_5_O_5_S_2_ [M + H]^+^, 470.1526; found, 470.1525.

#### *N*-(4-Amino-6-phenethoxy-2-(phenethylthio)pyrimidin-5-yl)-3-nitrobenzenesulfonamide (**39**)

Light yellow solid. Yield 56%. mp [299 °C with dec]. ^1^H NMR (600 MHz, DMSO-*d*_6_): δ 9.55 (s, 1H), 8.56–8.50 (m, 2H), 8.18–8.12 (m, 1H), 7.90 (t, *J* = 7.9 Hz, 1H), 7.25–7.14 (m, 8H), 7.07–7.01 (m, 2H), 6.77 (br s, 2H), 3.90 (t, *J* = 7.9 Hz, 2H), 3.18–3.13 (m, 2H), 2.91–2.81 (m, 2H), 2.35 (t, *J* = 7.9 Hz, 2H). ^13^C NMR (151 MHz, DMSO): δ = 167.89, 164.20, 163.61, 148.18, 143.46, 140.74, 137.45, 133.43, 131.51, 129.13, 128.91, 128.89, 128.75, 127.52, 126.91, 126.63, 122.12, 93.09, 66.81, 36.07, 34.72, 32.06. Anal. Calcd for C_26_H_25_N_5_O_5_S_2_: C, 56.61; H, 4.57; N, 12.70. Found: C, 56.70; H, 4.48; N, 12.72; HRMS (*m*/*z*) (ESI): calcd for C_26_H_24_N_5_O_5_S_2_ [M – H]^−^, 550.1224; found, 550.1247.

#### *N*-(4-Amino-6-phenethoxy-2-(phenethylthio)pyrimidin-5-yl)-3-nitro-*N*-phenethylbenzenesulfonamide (**40**)

Light yellow solid. Yield 61%. mp [280–283 °C]. ^1^H NMR (600 MHz, DMSO-*d*_6_): δ 8.57 (t, *J* = 1.5 Hz, 1H), 8.47 (dt, *J* = 7.5, 1.5 Hz, 1H), 8.05 (dt, *J* = 7.5, 1.5 Hz, 1H), 7.87 (t, *J* = 7.5 Hz, 1H), 7.31–7.15 (m, 15H), 7.05 (m, 2H) 4.42 (dt, *J* = 13.3, 7.1 Hz, 1H), 4.36–4.28 (m, 2H), 4.13 (dt, *J* = 13.4, 7.1 Hz, 1H), 3.34 (t, *J* = 7.1 Hz, 2H), 3.08–2.94 (m, 7H). ^13^C NMR (151 MHz, DMSO-*d*_6_): δ 169.28, 164.26, 163.22, 146.61, 139.72, 138.76, 138.10, 137.81, 131.02, 130.94, 128.95, 128.65, 128.63, 128.50, 128.35, 128.09, 126.83, 126.30, 126.27, 126.18, 122.62, 111.65, 67.76, 53.59, 35.70, 34.30, 33.43, 33.04. Anal. Calcd for C_34_H_33_N_5_O_5_S_2_: C, 62.27; H, 5.07; N, 10.68. Found: C, 62.18; H, 5.01; N, 10.64; HRMS (*m*/*z*) (ESI): calcd C_34_H_34_N_5_O_5_S_2_ [M + H]^+^, 656.1996; found, 656.2004.

#### 3-(*N*-(4-Amino-6-phenethoxy-2-(phenethylthio)pyrimidin-5-yl)sulfamoyl)benzenaminium Chloride (**41**)

White solid. Yield 59%. mp [298–300 °C]. ^1^H NMR (300 MHz, DMSO-*d*_6_): δ 9.62 (s, 1H), 7.58 (d, *J* = 7.5 Hz, 1H), 7.48–7.35 (m, 2H), 7.24 (q, *J* = 10.5, 8.9 Hz, 10H), 7.07 (d, *J* = 8.4 Hz, 1H), 6.94 (d, *J* = 8.4 Hz, 1H), 6.85 (d, *J* = 7.4 Hz, 1H), 5.44 (br s, 2H), 4.30 (t, *J* = 7.1 Hz, 2H), 3.34 (t, *J* = 7.2 Hz, 2H), 3.02 (dt, *J* = 13.9, 7.1 Hz, 4H). ^13^C NMR (75 MHz, DMSO-*d*_6_): δ 167.81, 163.64, 162.93, 148.31, 140.83, 139.98, 137.42, 131.13, 130.27, 130.14, 129.93, 129.31, 127.61, 127.51, 122.91, 119.84, 113.94, 107.35, 69.08, 37.02, 34.76, 34.38. Anal. Calcd for C_26_H_28_ClN_5_O_3_S_2_: C, 55.95; H, 5.06; N, 12.55. Found: C, 55.90; H, 5.10; N, 12.64. HRMS (*m*/*z*) (ESI): calcd C_26_H_28_N_5_O_3_S_2_ [M + H]^+^, 522.1628; found, 522.1625.

#### 3-(*N*-(4-Amino-6-phenethoxy-2-(phenethylthio)pyrimidin-5-yl)-*N*-phenethylsulfamoyl)benzenaminium Chloride (**42**)

Light yellow solid. Yield 59%. mp [279–282 °C]. ^1^H NMR (300 MHz, DMSO-*d*_6_): δ 7.51 (d, *J* = 4.6 Hz, 2H), 7.44 (s, 1H), 7.35–6.98 (m, 18H), 6.89 (t, *J* = 4.7 Hz, 1H), 5.20 (s, 2H), 4.42 (dt, *J* = 33.3, 7.0 Hz, 3H), 4.17 (dt, *J* = 13.7, 7.1 Hz, 1H), 3.38 (t, *J* = 7.1 Hz, 2H), 3.14–2.97 (m, 6H). ^13^C NMR (75 MHz, DMSO-*d*_6_): δ 168.91, 163.89, 162.85, 146.85, 139.39, 138.36, 137.44, 135.93, 129.79, 128.58, 128.28, 128.27, 128.13, 127.98, 127.68, 125.94, 125.90, 125.86, 119.59, 118.12, 112.70, 111.28, 67.40, 53.22, 35.34, 33.82, 33.06, 32.68. Anal. Calcd for C_34_H_36_ClN_5_O_3_S_2_: C, 61.66; H, 5.48; N, 10.58. Found: C, 61.60; H, 5.50; N, 10.55. HRMS (*m*/*z*) (ESI): calcd C_34_H_36_N_5_O_3_S_2_ [M + H]^+^, 626.2254; found, 626.2250.

#### *N*-Allyl-*N*-(4-(allyloxy)-2-(allylthio)-6-aminopyrimidin-5-yl)-4-nitrobenzenesulfonamide (**43**)

Light yellow solid. Yield 92%. mp [267–268 °C]. ^1^H NMR (400 MHz, DMSO-*d*_6_): δ 8.32–8.25 (m, 2H), 8.01–7.94 (m, 2H), 7.12 (d, *J* = 7.7 Hz, 1H), 6.98 (d, *J* = 7.7 Hz, 1H), 6.09–5.86 (m, 3H), 5.34 (dt, *J* = 13.4, 1.1 Hz, 2H), 5.24 (dp, *J* = 13.4, 1.0 Hz, 2H), 5.20–5.13 (m, 2H), 4.85–4.77 (m, 4H), 3.85 (dt, *J* = 6.1, 1.0 Hz, 2H). ^13^C NMR (100 MHz, DMSO-*d*_6_): δ 168.18, 163.07, 162.88, 148.83, 142.53, 134.11, 132.43, 131.80, 128.47, 124.32, 118.17, 117.75, 117.36, 111.81, 68.74, 49.84, 33.79. Anal. Calcd for C_19_H_21_N_5_O_5_S_2_: C, 49.23; H, 4.57; N, 15.11. Found: C, 49.30; H, 4.52; N, 15.05. HRMS (*m*/*z*) (ESI): calcd C_19_H_22_N_5_O_5_S_2_ [M + H]^+^, 464.1057; found, 464.1055.

#### *N*-(4-(*N*-(4-Amino-6-(3-phenylpropoxy)-2-((3-phenylpropyl)thio)pyrimidin-5-yl)sulfamoyl)phenyl)acetamide (**44**)

White solid. Yield 37%. mp [264 °C with dec]. ^1^H NMR (600 MHz, DMSO-*d*_6_): δ 10.29 (s, 1H), 8.94 (br s, 1H), 7.72 (d, *J* = 8.9 Hz, 2H), 7.64 (d, *J* = 8.8 Hz, 2H), 7.25 (q, *J* = 7.4 Hz, 5H), 7.19–7.13 (m, 5H), 6.53 (br s, 2H), 3.67 (t, *J* = 6.6 Hz, 2H), 2.92 (t, *J* = 7.3 Hz, 2H), 2.65–2.59 (m, 2H), 2.48–2.40 (m, 2H), 2.06 (s, 3H), 1.93–1.79 (m, 2H), 1.54–1.43 (m, 2H). ^13^C NMR (151 MHz, DMSO): δ 169.36, 167.30, 164.34, 163.38, 143.39, 141.73, 134.97, 128.76, 128.71, 128.66, 128.47, 126.26, 126.19, 118.61, 93.92, 65.41, 34.75, 31.54, 31.46, 30.17, 30.01, 24.59. Anal. Calcd for C_30_H_33_N_5_O_4_S_2_: C, 60.89; H, 5.62; N, 11.84. Found: C, 61.00; H, 5.77; N, 11.86; HRMS (*m*/*z*) (ESI): calcd for C_30_H_33_N_5_O_4_S_2_ [M – H]^−^, 590.1901; found, 590.1885.

### Biological Evaluation

#### General Procedures for HTS Experiments

All chemical reagents, cell culture media, and standard inhibitors were of the highest quality and included penicillin G (P-11–010, PAA Laboratories GmbH, Austria), MitoTracker Red CMXRos (Thermo, Waltham, MA), SYBR Green (Invitrogen, Waltham, MA), Trichostatin A (Sigma-Aldrich, St. Louis, MO), SU6656 (A15518-10, Calbiochem, Burlington, MA), E-4031 (BML-KC158-0005, Enzo Life Sciences, Inc., Farmingdale, NY), valinomycin (V0627, Sigma-Aldrich), paclitaxel (T7191, Sigma-Aldrich), methotrexate (ALX-440-045-M100, Enzo Life Sciences, Inc.), cytochrome *c* (C2037, Sigma-Aldrich), dihydrobiopterin (H2B; 37,272, Sigma-Aldrich), pyrimethamine (46,706, Sigma-Aldrich), alpha-naphthoflavone (N5757-1G, Sigma-Aldrich), sulfaphenazole (S0758-1G, Sigma-Aldrich), troglitazone (T2573-5MG, Sigma-Aldrich) quinidine (Q3625-5G, Sigma-Aldrich), ketoconazole (K1003, Sigma-Aldrich), amphotericin B (A9528, Sigma-Aldrich), and pentamidine isethionate salt (Sigma-Aldrich). Benznidazole was a kind gift of Nortec Química (Duque de Caxias—RJ, Brazil). Compounds were dissolved to yield stock solutions in 100% v/v DMSO (Carl Roth GmbH & Co. KG, Karlsruhe, Germany) and stored at −20 °C. Assay kits used in the ADME-Tox assay panel included cytotoxicity CellTiter-Glo (CTG) reagent (Promega Corp., Madison, WI); hERG assay (Predictor hERG, Thermo); CYP P450 1A2, 2C9, 2C19, 2D6, and 3A4 assays (P450-Glo, Promega Corp.); CYP P450 preparations as Supersomes (Corning Inc., Corning, NY); Aurora B ADP-Glo Kinase Enzyme System (Promega Corp.); and HDAC (HDAC-Glo Class I/II Kits, Promega Corp.). Media Culture and Fetal Bovine Serum for *T. brucei*, *T. cruzi,* and *L. infantum* screening assays were purchased from Gibco-ThermoScientific. The *T. brucei* phenotypic screen was performed using a Janus MDT (PerkinElmer), equipped with a 384-head, WellMate (Thermo), and MixMate plate mixer (Eppendorf AG). Assay measurements were taken using the EnVision Multilabel 2103 Reader (PerkinElmer) or Infinite M1000 PRO plate reader (Tecan Group Ltd.). Images from the mitochondrial toxicity assay were taken using an Opera automated microscope (PerkinElmer). The CYP450 assay was performed using the Tecan Fluent liquid-handling automation platform (Tecan Group Ltd.).

#### In Vitro Evaluation of Activity against *T. brucei*

The efficacy of compounds against *T. brucei* bloodstream forms was evaluated using a modified resazurin-based assay previously described in literature.^52^ Mid log bloodstream forms were added to an equal volume of serial dilutions of compounds in a supplemented complete HMI-9 medium at a final cell density of 5 × 10^3^/mL. Following incubation for 72 h at 37 °C 5% CO_2_, 20 μL of a 0.5 μM resazurin solution was added and plates were incubated for a further 4 h under the same conditions. Fluorescence was measured at 540 and 620 nm excitation and emission wavelength, respectively, using a Synergy 2 Multi-Mode Reader (Biotek). This assay was successfully miniaturized into a 384-well microtiter plate and met the criteria for suitability in a screening campaign. The parameters investigated included concentrations of cells, assay media composition, incubation time and temperature, Z′, DMSO tolerance, and reproducibility of the potencies of the reference compound pentamidine (3.17 ± 0.69 nM). The *T. brucei* phenotypic screen was performed using a Janus MDT (PerkinElmer), equipped with a 384-head, WellMate (Thermo) and MixMate plate mixer (Eppendorf AG). Assay measurements were taken using the EnVision Multilabel 2103 Reader (PerkinElmer) or Infinite M1000 PRO plate reader (Tecan Group Ltd.). Images from the mitochondrial toxicity assay were taken using an Opera automated microscope (PerkinElmer). The CYP450 assay was performed using the Tecan Fluent liquid-handling automation platform (Tecan Group Ltd.).

#### In Vitro Evaluation of Activity against *L. Infantum* Intramacrophage Amastigotes

Antiparasitic activity against *L. infantum* intracellular amastigotes at 10 μM was determined according to literature.^53^ Briefly, THP-1 cells were differentiated to macrophages, infected with *L. infantum* promastigotes and, 24 h after infection, were treated with compounds, incubated for another 72 h, and then submitted to high content analysis for determination of antiparasitic activity. The Operetta high-content automated imaging system (PerkinElmer) was used to acquire images and the Harmony Software (PerkinElmer) was optimized quantifying host cells number, infection ratio and number of parasites per infected cell. The ratio between infected cells and total number of cells is then calculated and defined as the infection ratio (IR). The *Z*′-factor was determined for each plate based on the IR for control wells and used as quality control criteria for plate approval. The IR was normalized to positive (amphotericin B-treated infected cells) and negative (vehicle-treated infected cells) and was used to determine the reduction of infection as the antiparasitic activity.

#### In Vitro Culture of *T. cruzi*

The drug assay method consists of infecting the osteosarcoma-derived human U2OS cell-line with tissue-derived trypomastigote forms of *T. cruzi* for 24 h prior to the addition of the compounds to 384-well plates, as previously described.^46^ Infected cultures were exposed to compounds for 72 h. Plates were processed for high content analysis as described above for the *Leishmania* assay.

#### *M. tuberculosis*

As described in ref (37).

#### *h*ERG Assay

This assay made use of the Predictor *h*ERG fluorescence polarization assay (Thermo). The assay uses a membrane fraction containing *h*ERG channel (Predictor *h*ERG Membrane) and a high-affinity red fluorescent *h*ERG channel ligand, or “tracer” (Predictor *h*ERG Tracer Red), whose displacement by test compounds can be determined in a homogeneous, fluorescence polarization (FP)-based format.^54^

#### Cytochrome P450 1A2, 2C9, 2C19, 2D6, and 3A4 Assays

These assays made use of the P450-Glo assay platform (Promega). Each CYP450 assay made use of microsomal preparations of cytochromes from baculovirus infected insect cells. Action of the CYP450 enzymes upon each substrate ultimately resulted in the generation of light and a decrease in this was indicative of inhibition of the enzymes.^54^

#### Cytotoxicity Assay against the A549 Cell-Line

The assays were performed using the Cell Titer-Glo assay (Promega). The assay detects cellular ATP content with the amount of ATP being directly proportional to the number of cells present. The A549 cells were obtained from DSMZ (German Collection of Microorganisms and Cell Cultures, Braunschweig, Germany) and WI38cells were obtained from ATCC (ATCC CCL-75) and were grown in DMEM with FCS (10% v/v), streptomycin (100 μg/mL), and penicillin G (100 U/mL).^54^

#### Assessment of Mitochondrial Toxicity

This assay made use of MitoTracker Red chloromethyl-X-rosamine (CMXRos) uptake and high content imaging to monitor compound-mediated mitochondrial toxicity in the 786-O (renal carcinoma) cell line. Cells were maintained using a RPMI-1640 medium containing 2 mM glutamine, FCS (10% v/v), streptomycin (100 μg/mL), and penicillin G (100 U/mL).^54^

#### Biological Data Analysis

The screening data were analyzed using ActivityBase (IDBS, Guildford, UK), and outlier elimination in the control wells was performed using the 3-sigma method. Unless stated, dose–response experiments were performed in the 11-point format with the IC_50_ (or EC_50_) value, Hill slope, minimum signal, and maximum signal for each dose–response curve obtained using a four-parameter logistic fit in the XE module of ActivityBase (IDBS).

### Statistical Analysis

#### Bayesian Machine Learning

We generated and validated Laplacian-corrected naive Bayesian classifier models using Discovery Studio version 4.1 (Biovia, San Diego, CA). Values of the *A* log *P*; molecular weight; number of rotatable bonds, rings, aromatic rings, hydrogen bond acceptors, and hydrogen bond donors; molecular fractional polar surface area; and FCFP_6 were used as the molecular descriptors. Compounds were set as active when (i) % antiparasitic activity >80% at 50 μM against *T. brucei*, (ii) antiparasitic activity >40% at 50 μM against amastigote *L. infantum* and *T. cruzi*, and (iii) >20% activity at 20 μM against both replicant and nonreplicant strains of *Mtb*. Similarly, compounds with % adverse activity (>50% against the five CYP isoforms at 50 μM, >20% against hERG at 10 μM, >50% against mitochondrial toxicity at 10 μM, and >30% cytotoxicity against the A549 cell-line) were set as toxic. Computational models were validated using leave-one-out (LOO) cross validation, in which each sample was left out one at a time. A model was built using the remaining samples, and that model was used to predict the left-out sample. Each model was internally validated, receiver operator characteristic curve (ROC) plots were generated, and the cross-validated ROC’s “area under the curve” was calculated. Then, 5-fold cross validation (i.e., leave out 20% of the data set, and repeat five times) was also performed.

Assay Central, the proprietary Assay Central software, has been described elsewhere.^11,21,23,55−61^ It uses automated workflows to detect problematic molecules for proper integration into machine learning methods, which can then be rapidly corrected using automation standardization and human recuration. This software also outputs a high-quality data set and a Bayesian machine learning model that may be used to predict potential bioactivity of additional compounds. These models are generated with ECFP6 descriptors produced from the CDK library^54^ that have been used for structure–activity relationships.^62^ Each Bayesian machine learning model also includes metrics to evaluate internal 5-fold cross-validation performance,^21^ including Receiver Operator Characteristic (ROC), Recall, Precision, F1 Score, Cohen’s Kappa,^63,64^ and Matthews Correlation Coefficient.^65^ The prediction scores generated with Assay Central model^62,66^ as a probability-like score determined by the ratio of fingerprints (e.g., ECFP_6) in active and inactive molecules, with a value of 0.5 or greater designating a chemical as active at the modeled target.

#### Comparison of Machine Learning Algorithms

The data sets were used for the comparison of additional machine learning algorithms, namely, random forest, *k*-nearest neighbors, support vector classification, naïve Bayesian, AdaBoosted decision trees, and deep learning architecture.^49^ These alternative machine learning methods use ECFP6 as the molecular descriptors. 5-fold cross-validation metrics were compared across all algorithms with a rank normalized score.^61,67^ These scores can be evaluated pairwise (i.e., method per training set) or independently (i.e., generally method comparison). A further measure is a “difference from the top” (ΔRNS) metric which subtracts the rank normalized score for each algorithm from the highest rank normalized score for a specific training data set. This method maintains the pairwise results from each training set score by algorithm and allows a direct performance comparison of any two machine learning algorithms and yet maintains the information from the other six algorithms.

## Data Availability

SMILES string for Ty-Box components and secondary hits are accessible on fairdom-hub at the following link: https://fairdomhub.org/data_files/4067?version=1. Authors are available for contacts to make all information accessible upon requests. Molecular Formula Strings data are accessible at https://fairdomhub.org/data_files/6749.

## Supporting Information Available

Entire panel of data for activity and toxicity profile of the 456-training set of Ty-Box compounds; toxicity profiles for the reference drugs pentamidine, benznidazole, amphotericin B, and miltefosine; antiparasitic and antitubercular activity of the selected primary hits in secondary dose–response studies; Assay Central model statistics; predicted antiparasitic activity and early toxicity-related issue according to the elaborated Bayesian model and experimental values for the test compounds;·sdf file of Ty-Box compounds and secondary hits; ^1^H and ^13^C NMR spectra of the secondary hit; and raw data for predicted physicochemical properties for Ty-Box compounds, activity and toxicity profile of the 456-training set of Ty-Box compounds,) predicted antiparasitic activity according to the elaborated Bayesian model and experimental antiparasitic activity, and predicted according to the elaborated Bayesian model versus experimental early toxicity profile of the secondary hits (PDF)
Molecular formula strings (CSV)

## Abbreviations

AcOEt: ethyl acetate
ADME-Tox: absorption, distribution, metabolism, elimination, toxicity
BALB/c mice: laboratory-bred strain of the house mouse
CE: cyclohexane
ChEMBL: database of compounds
CTG: CellTiter-Glo
CYP: cytochrome P450 enzymes
DCM: dichloromethane
DIPEA: *N*,*N*-diisopropylethylamine
DMF: dimethylformamide
DMSO: dimethyl sulfoxide
EC_50_: half maximal effective concentration
ECFP-6: extended-connectivity fingerprints of maximum diameter
Et_2_O: diethyl ether
EtOH: ethanol
FCFP_6: molecular function class fingerprints of maximum diameter 6
H37Rv: replicant strains of *Mtb*
HAT: human african trypanosomiasis
HBA: number of H-bond acceptors
HBD: number of H-bond donors
HDAC: (HDAC-Glo Class I/II Kits, Promega Corp.)
*h*ERG: human ether-à-go-go-related gene
HRMS: high-resolution mass spectrometry
HTS: high-throughput screening
IC_50_: half-maximal inhibitory concentration
LC: column liquid chromatography
LC/MS: liquid chromatography tandem mass spectrometer
*Lm*: *Leishmania major*
mp: melting point
MeOH: methanol
MM4TB: more medicines for tuberculosis
*Mtb*: *Mycobacterium tuberculosis*
MTS: medium-throughput screening
NIDs: neglected infectious diseases
nM: nanomolar
NMR: nuclear magnetic resonance
NMTrypI: new medicine for trypanosomatidic infection
PTR1: pteridine reductase 1
Q-TOF: quadrupole time-of-flight mass spectrometer
RO5: lipinski’s “rule of five”
ROC: receiver operating characteristic
SAR: structure–activity relationship
Sn2: nucleofilic substitution
ss18b: nonreplicant strains of *Mtb*
*t*-SNE: 3D and 2D *t*-stochastic neighbor embedding
*Tb*: *Trypanosoma brucei*
TB: tuberculosis
*Tc*: *Trypanosoma cruzi*
TEA: triethylamine
THF: tetrahydrofuran
TLC: thin-layer chromatography
U2OS: human osteosarcoma cell-line
UV light: ultraviolet light
WHO: World Health Organization
μM: micromolar
