# Supplementary material for: High-Throughput Phenotypic Screening and Machine Learning Methods Enabled the Selection of Broad-Spectrum Low-Toxicity Antitrypanosomatidic Agents
Source: J Med Chem. 2023 Nov 3;66(22):15230–55. doi: 10.1021/acs.jmedchem.3c01322 (PMC10683024; doi:10.1021/acs.jmedchem.3c01322)
Supplement: Supplementary file 1 — jm3c01322_si_001.pdf [file jm3c01322_si_001.pdf]

Supporting Information

High-throughput phenotypic screening and machine learning methods enabled the selection of broad spectrum low-toxicity anti-trypanosomatidic agents.

Pasquale Linciano[1†‡], Antonio Quotadamo[1†], Rosaria Luciani[1], Matteo Santucci[1], Kimberley M. Zorn[2], Daniel H. Foil[2], Thomas R. Lane[2], Anabela Cordeiro da Silva[3,4], Nuno Santarem[3,4], Carolina B. Moraes[5,¥], Lucio Freitas-Junior[5], Ulrike Wittig[6], Wolfgang Mueller[6], Michele Tonelli[7], Stefania Ferrari[1], Alberto Venturelli[1,8], Sheraz Gul[9,10], Maria Kuzikov[9,10], Bernhard Ellinger[9,10], Jeanette Reinshagen[9,10,] Sean Ekins[2*], Maria Paola Costi[1*].*

[1]Department of Life Sciences, University of Modena and Reggio Emilia, via Campi 103, 41126, Modena (MO), Italy.

[2]Collaborations Pharmaceuticals, Inc., 840 Main Campus Drive, Lab 3510, Raleigh, NC 27606, USA.

[3] Institute for Molecular and Cell Biology, 4150-180 Porto, Portugal.

[4] Instituto de Investigaçao e Inovaçao em Saúde, Universidade do Porto and Institute for Molecular and Cell Biology, 4150-180 Porto, Portugal.

[5] Brazilian Biosciences National Laboratory (LNBio), Brazilian Center for Research in Energy and Materials (CNPEM), 13083-970, Campinas, SP, Brazil.

[6]Scientific Databases and Visualization Group and Molecular and Cellular Modelling Group, Heidelberg Institute for Theoretical Studies (HITS), D-69118 Heidelberg, Germany.

[7] Department of Pharmacy, University of Genoa, viale Benedetto XV n.3, 16132 Genoa, Italy.

[8]TYDOCK PHARMA S.r.l., Strada Gherbella 294/b, Modena, 41126, Italy.

[9]Fraunhofer Translational Medicine and Pharmacology, Schnackenburgallee 114, D-22525 Hamburg, Germany.

[10]Fraunhofer Cluster of Excellence Immune-Mediated Diseases CIMD, Schnackenburgallee 114, D-22525 Hamburg, Germany.

†These authors contributed equally to this work: P.L. and A.Q.

‡Present affiliation of P.L.: Department of Drug Sciences, University of Pavia, viale Taramelli 12, 27100, Pavia, Italy.

¥Present affiliation of C.B.M.: Departamento de Ciências Farmacêuticas, Universidade Federal de São Paulo, Diadema, Brazil.

Corresponding authors
*P.L. pasquale.linciano@unipv.it
*S.E. collaborationspharma@gmail.com
*M.P.C. mariapaola.costi@unimore.it

This PDF file includes:

Figures S1 to S7 pag S3 to S9

Tables S1 to S11 pag. S22 to S46

Other Supplementary Information for this manuscript include the following:

Data S1 pag. S47

References pag.S53

Proton and Carbon NMR spectra of compounds from Ty-01 to Ty-44 pag.S55 – S142

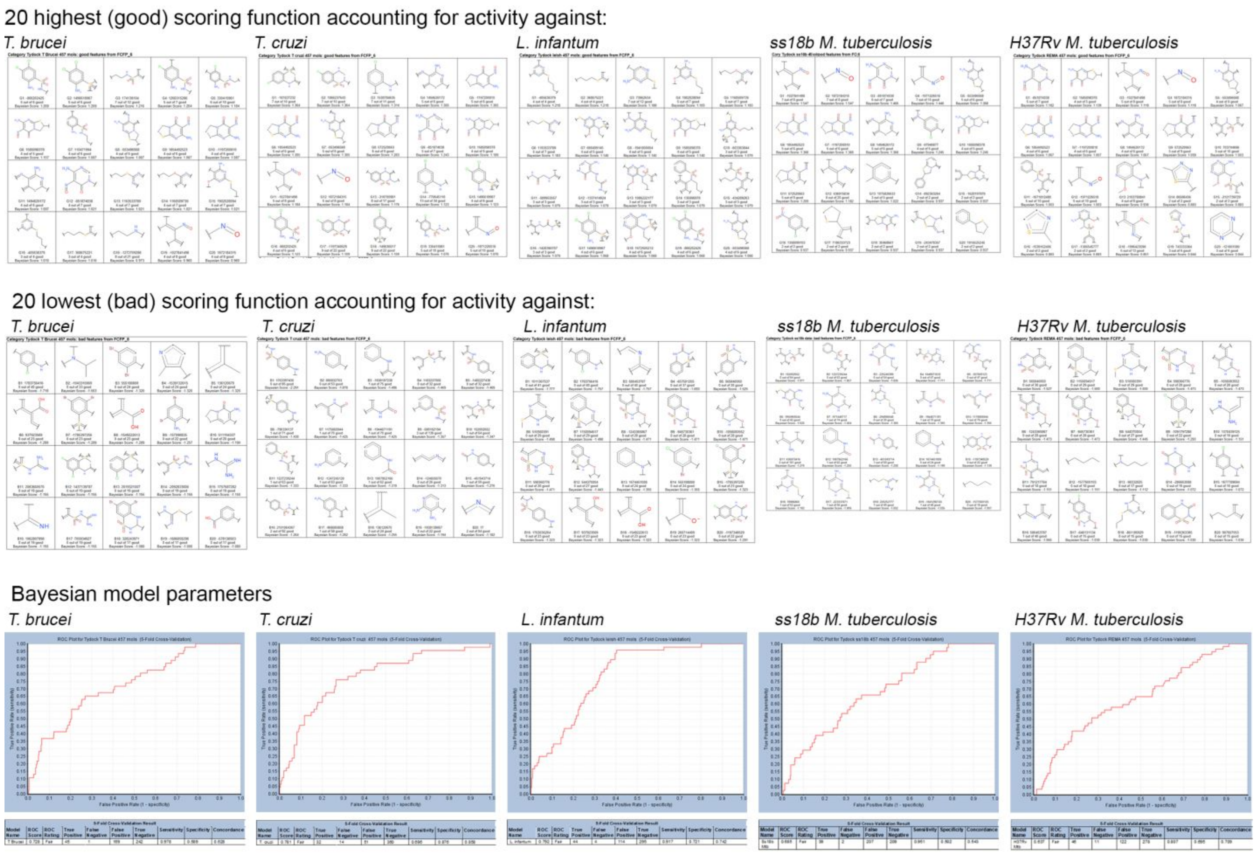

Fig. S1. 5-fold cross validation and good and bad fingerprints for activity Bayesian models generated with Discovery Studio using FCFP_6 descriptors and additional descriptors described in the methods.

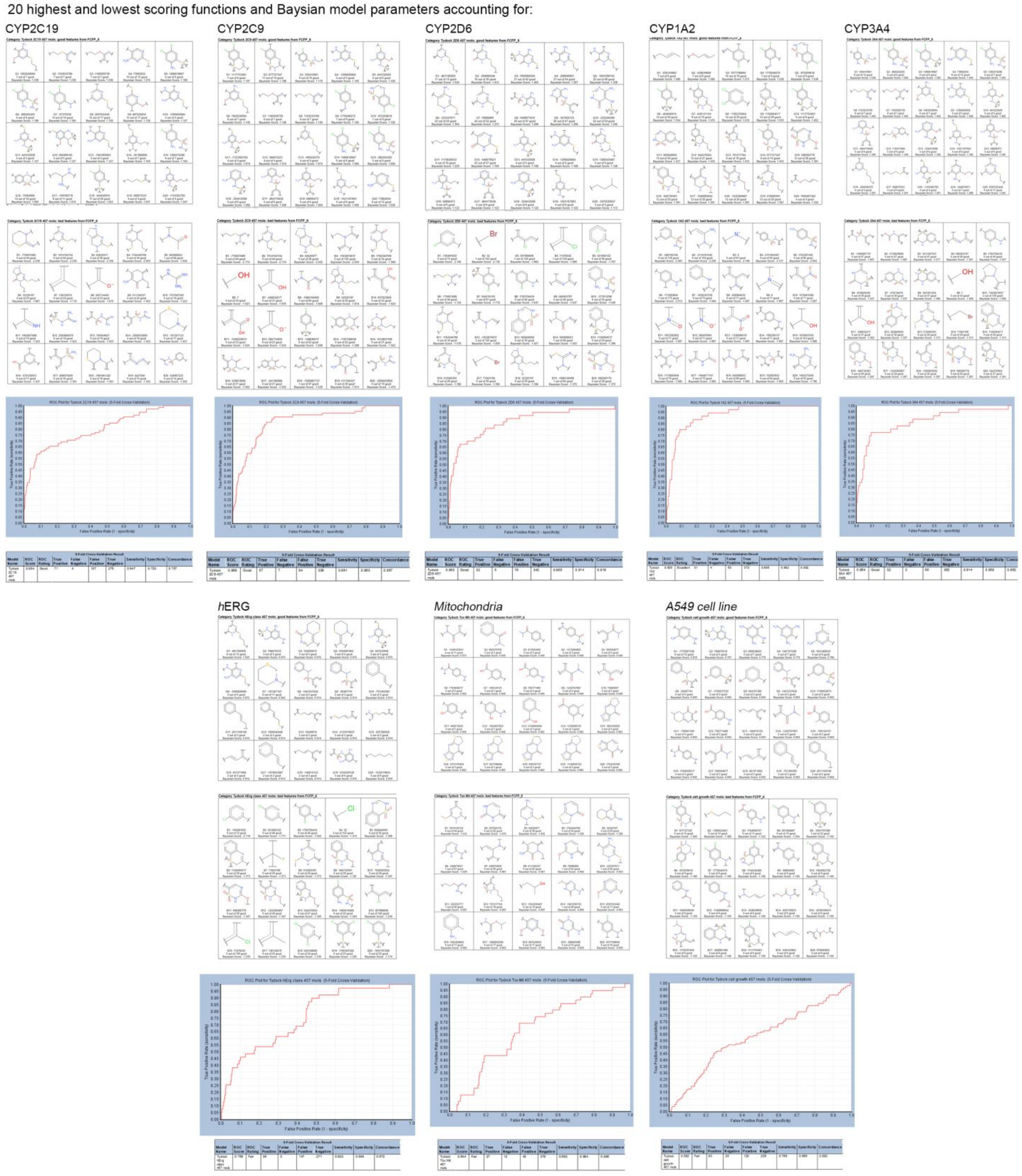

Fig. S2. 5-fold cross validation and good and bad fingerprints for toxicity Bayesian models generated with Discovery Studio using FCFP_6 descriptors and additional descriptors described in the methods.

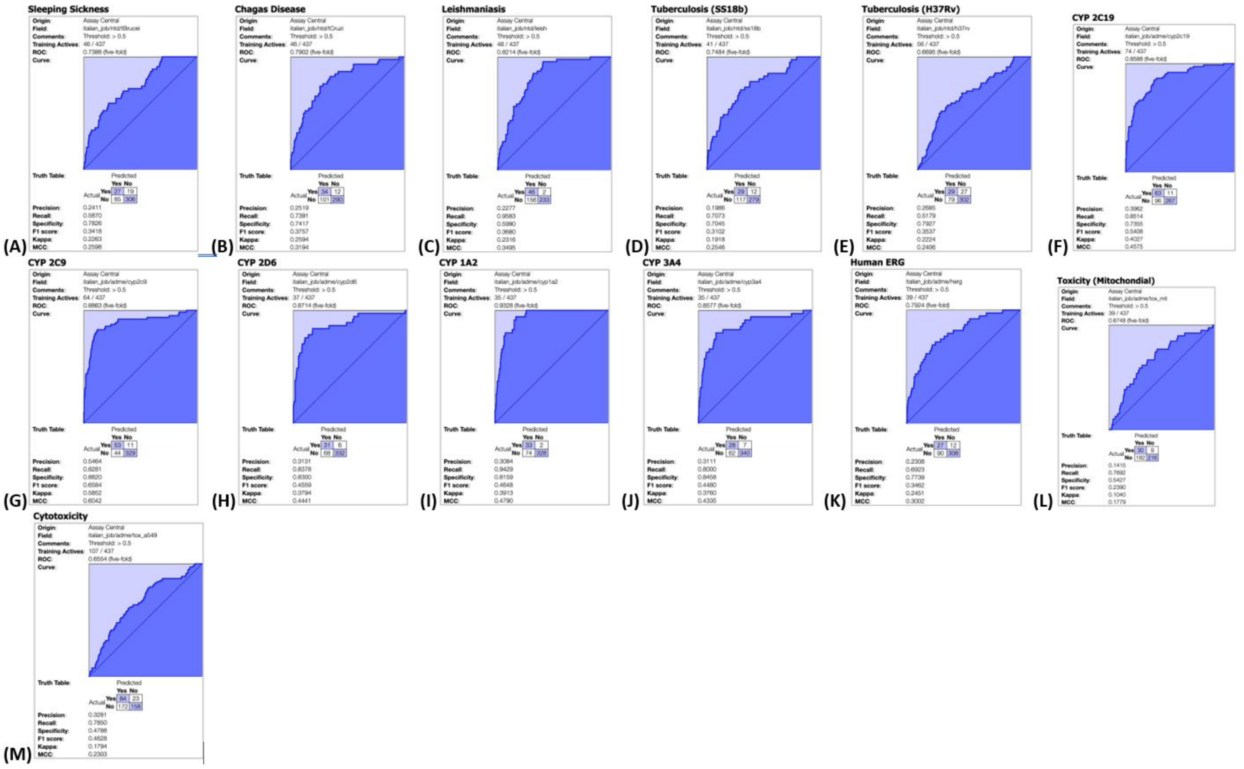

Fig. S3. ROC plots for 5-fold cross validation in Assay Central® using Bayesian models with ECFP6 descriptors only. (A) *T. brucei*. (B) *T. Cruz*. (C) *L. infantum*. (D) SS18b *Mtb*. (E) H37Rv *Mtb*. (F) CYP2C19. (G) CYP2C9. (H) CYP2D6. (I) CYP1A2. (J) CYP3A4. (K) *h*ERG. (L) mitochondrial toxicity. (M) A549 cell line.

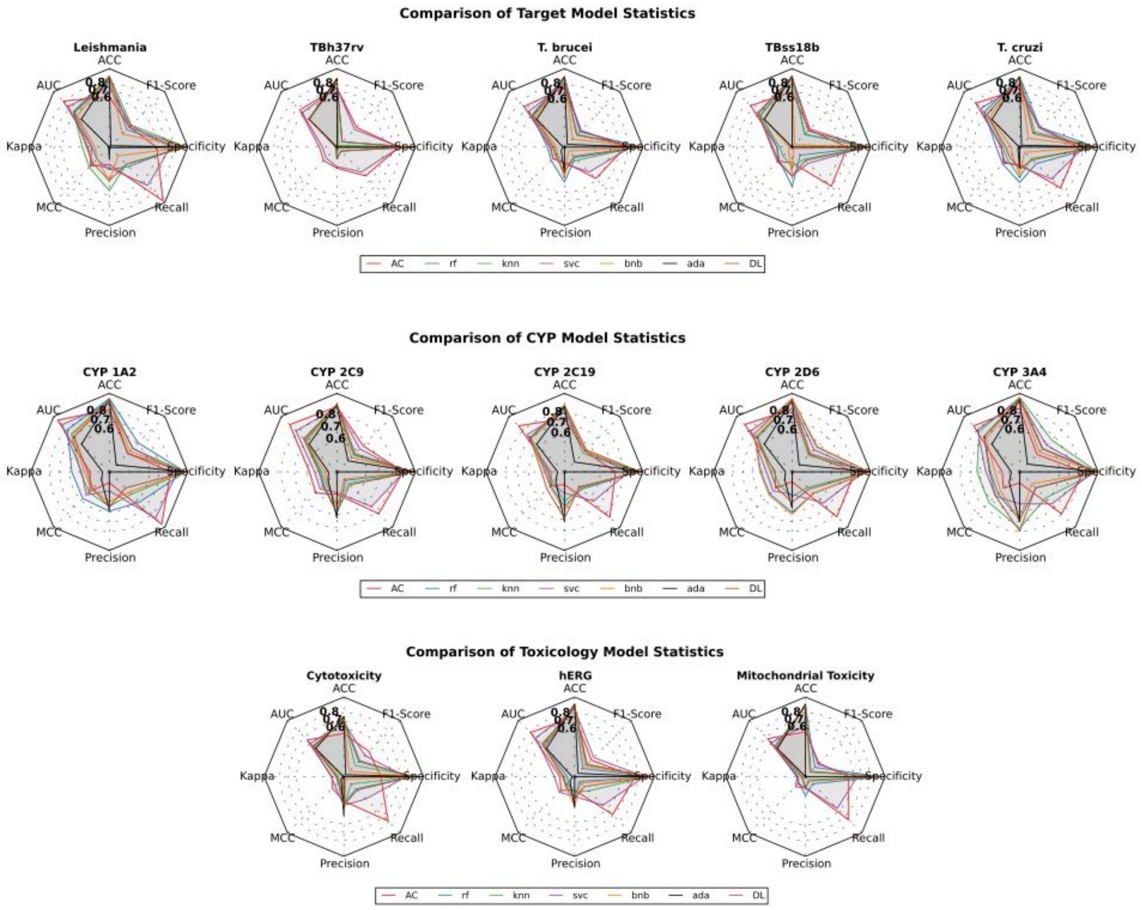

Fig. S4. Machine learning algorithm comparisons across multiple metrics for the training datasets. The radius of a given point reflects the value of the metric at each corner of the radar plot. Abbreviations: ROC = receiver operator characteristic, Kappa = Cohen's Kappa, MCC = Matthews Correlation Coefficient, AC = Assay Central® (Bayesian), rf = random forest, knn = k-Nearest Neighbors, svc = support vector classification, bnb = naïve Bayesian, ada = AdaBoosted decision trees, DL = deep learning architecture.

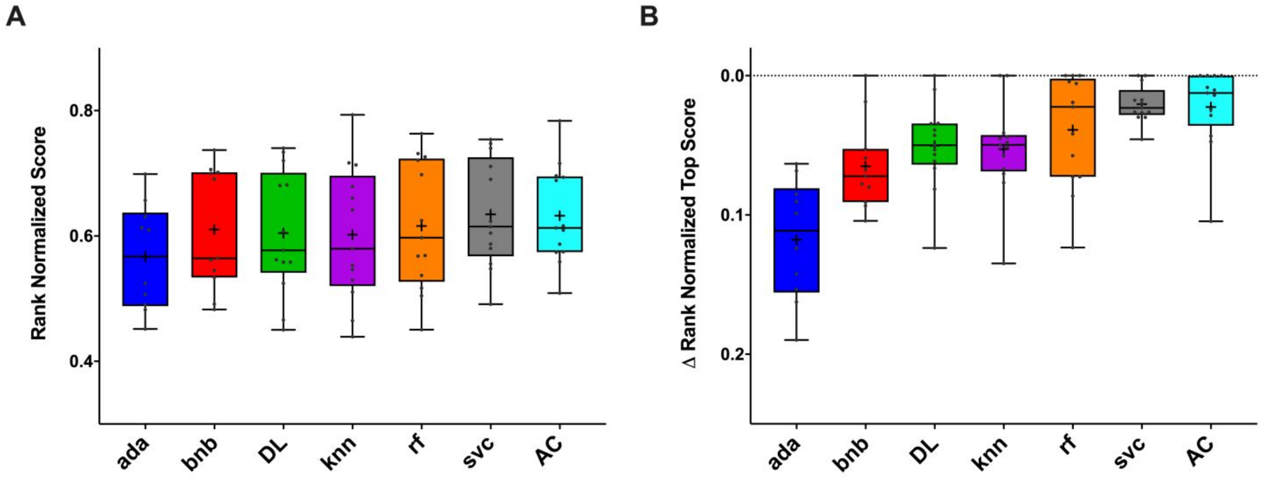

Fig. S5. Machine learning algorithm comparisons across multiple five-fold cross-validation metrics for all the machine learning methods. A) Rank normalized scores (RNS) and B) ΔRNS. Box and whisker plots show individual points for those values that fall outside of the 5-95 percentile. Abbreviations: AC = Assay Central® (Bayesian), rf = random forest, knn = k-Nearest Neighbors, svc = support vector classification, bnb = naïve Bayesian, ada = AdaBoosted decision trees, DL = deep learning architecture.

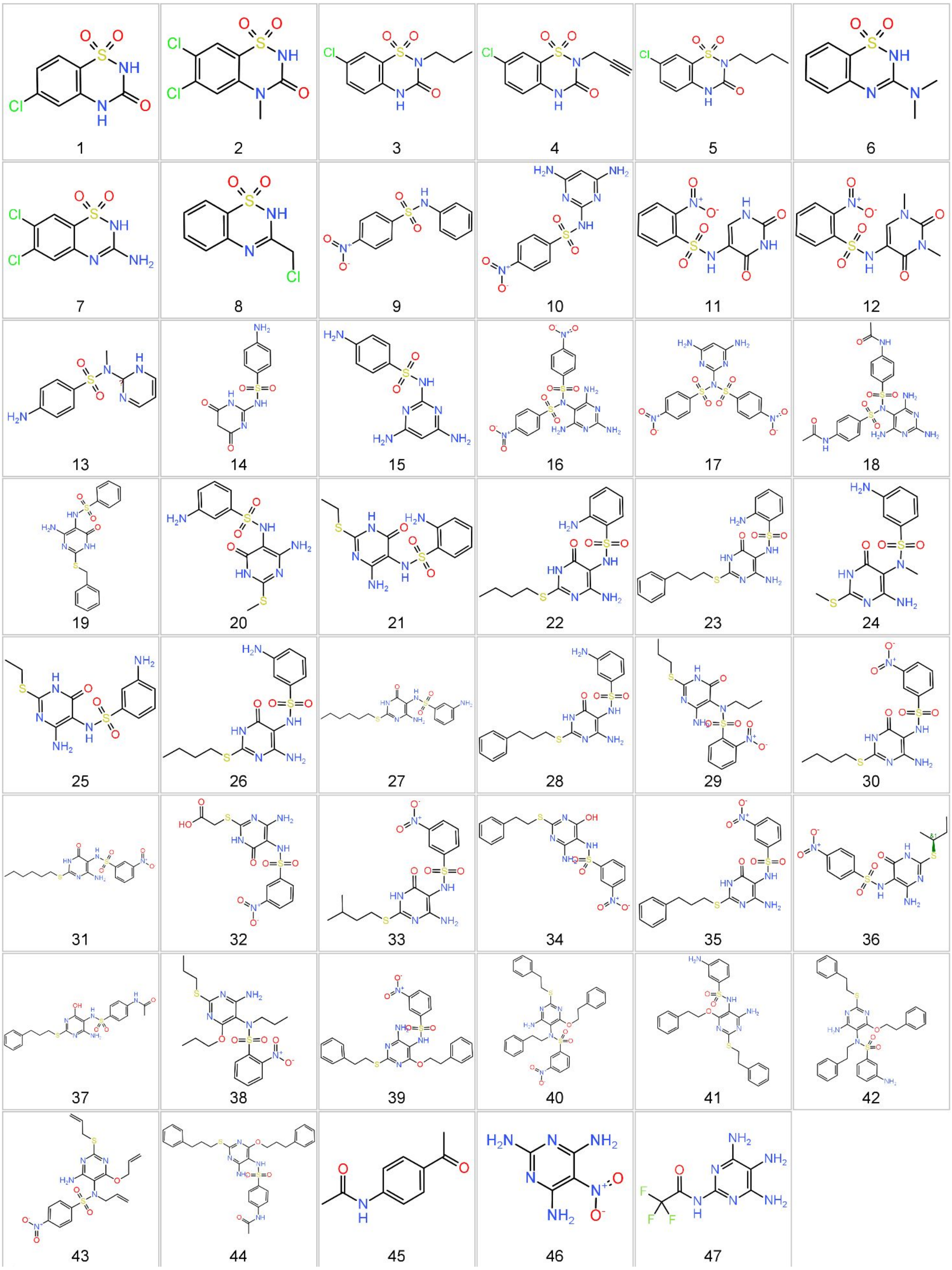

Fig. S6. Chemical structure of the test set of synthesized compounds.

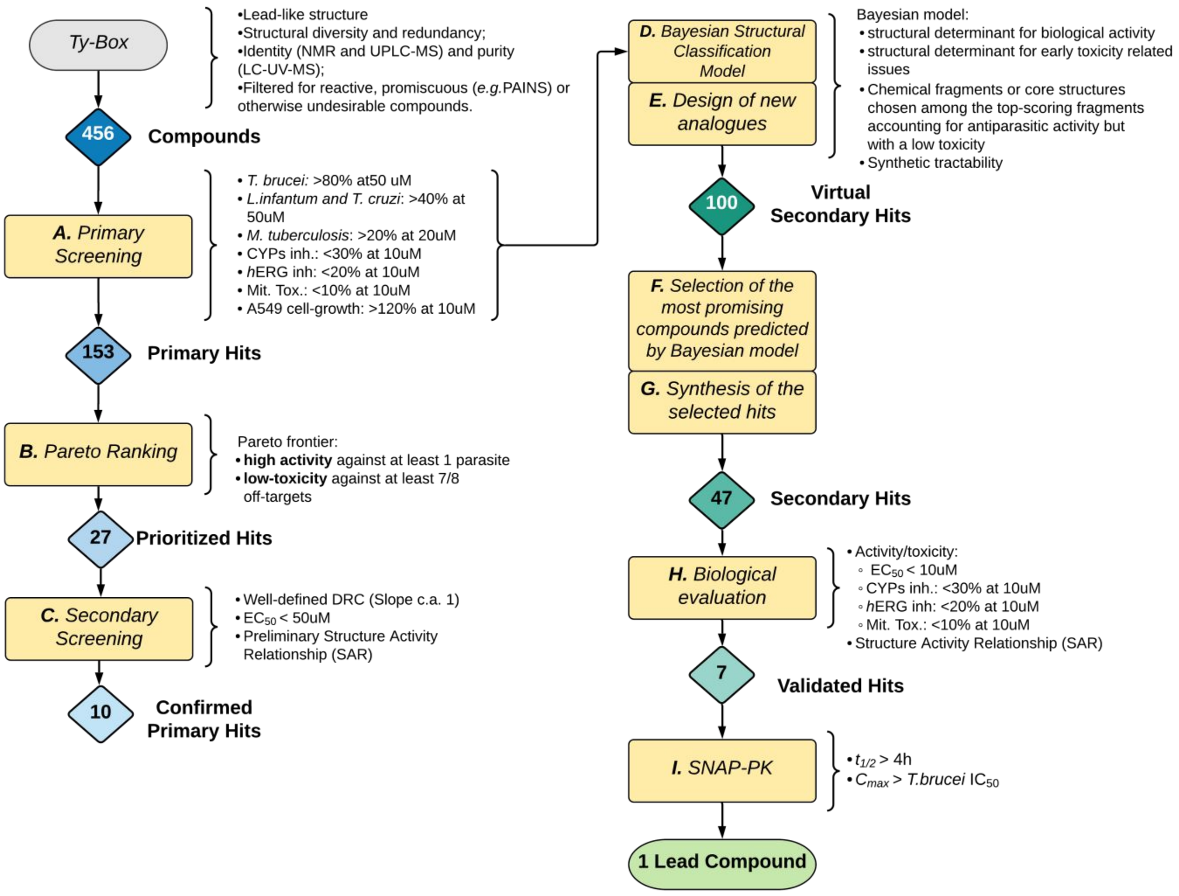

Figure S7. Screening cascade from primary screening to identification of a lead compound. For each step in the pipeline according to **Fig. 1**, the cut-off values and/or criteria adopted for selecting the molecule to progress in the following studies, and the numbers of the resulting compounds are reported.

Molecule	*T. brucei*	*L. major*	*T. cruzi*	*ss18*	*h37rv*	*h*ERG	CYP1A2	CYP2C9	CYP2C19	CYP2D6	CYP3A4	A549	Mitochondrial toxicity
**Ty-101**	0.41	26.6	-6.53	10.15	-3.35	1.95	1.7	-0.92	-35.5	-48.76	-11.88	137.56	-5.75
**Ty-102**	-0.8	1.38	-1.79	1.34	-9.04	11.17	4.17	-4.74	-36.14	-34.37	-9.15	128.79	-2.32
**Ty-103**	102.85	87.77	89.54	87.02	62.49	9.98	-0.13	-1.01	20.19	-9.07	-14.49	132.28	5.29
**Ty-104**	1.41	33.2	10.81	-3.95	-7.8	9.6	4.94	-4.3	-55.22	-30.92	-7.48	142.38	6.28
**Ty-105**	-0.4	6.64	2.35	-13.94	-0.93	-9	6.8	6.83	-22.69	-9.38	9.37	120.83	-8.88
**Ty-106**	-1	1.82	0.58	-11.4	18.68	4.19	-1.1	0.34	-37.56	-31.63	-4.69	138	-5.14
**Ty-107**	-1.81	33.66	14.2	-23.68	-4.99	2.45	2.58	2.55	-16.85	-24.23	-14.07	153.66	-8.2
**Ty-108**	-0.4	37.5	0.76	-8.63	-8.73	8.24	0.95	0.08	-27.97	-25.31	-8.03	149.48	-0.11
**Ty-109**	102.17	93.89	81.77	83.56	64.35	3.49	1.91	-1.54	-38.08	-18.61	-19.43	136.43	0.2
**Ty-110**	0.61	-9.29	34.27	-3.84	16.49	9.12	4.16	-2	-30.4	-22.2	-22.73	147	-9.38
**Ty-111**	-1	-12.95	-10.96	-8.95	9.04	-32.11	2.34	-1.37	-47.22	-23.07	-14.03	146.86	10.66
**Ty-112**	-1	-54.2	-24.77	-1.26	11.68	13.67	-1.49	-1.09	-24.87	-16.73	-3.33	156.01	-1.08
**Ty-114**	-0.6	2.56	-25.64	9.63	-0.03	-1.36	-1.55	-13.08	-24.25	-38.76	-19.36	114.85	-1.37
**Ty-115**	0.41	26.68	-10.51	15.15	2.71	-0.56	-5.45	-9.32	-35.32	-39.75	-0.85	127.07	-2.92
**Ty-116**	-0.8	21.5	-14.85	9.02	-0.2	1.66	10.17	-6.51	-26.22	-32.78	-16.52	132.55	-6.87
**Ty-117**	0.2	31.21	-6.48	3.63	1.18	-10.94	3.19	-11.55	-37.87	-35.2	-1.14	130.42	-0.35
**Ty-118**	0.41	33.84	-4.99	0.2	-8.4	-9.44	9.75	-3.35	-28.61	-46.79	-14.67	133.19	-5.26
**Ty-119**	1.42	-20.34	-13.73	11.03	12.58	-26.38	1.69	-5.03	-36.36	-16.97	-21.44	147.28	4.09
**Ty-120**	-1.61	-69.54	-45.52	-3.8	-8.81	21.13	7.49	40.86	-7.11	-8.56	37.09	158.84	13.24
**Ty-121**	0.81	6.93	19.49	-9.61	6.76	27.01	17.27	6.71	24	-6.77	19.98	162.12	3.59
**Ty-122**	-0.2	21.25	-15.04	-17.86	15.23	-2.43	2.94	-6.91	-38.83	-41.51	-12.46	133.6	-6.11
**Ty-123**	-2.81	-48.6	1.29	-9.81	16.07	4.43	9.5	-2.37	-16.67	-14.6	7.8	113.51	1.26
**Ty-124**	-1.4	17.7	-30.26	-1.61	6.91	-7.44	5.2	-5.05	-49.48	-32.53	-11.08	141.72	6.67
**Ty-125**	106.46	88.21	91.29	82.39	82.06	12.6	3.27	4.84	-24.72	-8.62	-1	57.41	5.38
**Ty-126**	-1.4	-7.63	-9.66	-10.3	1.44	14.52	8.4	3.21	-27.5	-23.05	26.62	126.99	-5.28
**Ty-127**	98.34	4.31	90.88	104.86	96.13	-3.72	12.94	4.28	-7.06	-25.17	-2.35	59.84	1.22
**Ty-128**	-1.2	-110.63	0.12	0.26	6.79	0.93	-6.28	1.1	-3.28	-20.23	-8.6	107.7	-2.41
**Ty-129**	2.42	-4.22	-9.28	15.64	19.66	-7.24	10.19	7.67	14.26	-5.86	30.74	135.52	-4.43

Molecule	*T. brucei*	*L. major*	*T. cruzi*	*ss18*	*h37rv*	*h*ERG	CYP1A2	CYP2C9	CYP2C19	CYP2D6	CYP3A4	A549	Mitochondrial toxicity
**Ty-130**	69.81	14.03	-30.13	26.29	-6.24	30.2	6.17	23.11	25.23	-3.9	21.17	158.97	-6.97
**Ty-131**	-0.4	0.75	-0.1	10.02	20.82	-3.47	9.02	-1.39	-35.53	-17.44	3.13	140.8	-4.98
**Ty-132**	2.32	22.49	0.12	4.48	-1.98	1.22	14.47	13.17	7.89	-11.37	54.41	140.46	-1.18
**Ty-134**	16.52	-2.53	48.98	38.7	-26.3	-9.57	0.31	2.63	-28.03	-12.18	-6.63	143.19	2.26
**Ty-135**	1.42	11.39	-22.9	7.6	-13.58	5.41	2.89	-4.31	-18.95	-22.25	-11.76	151.48	1.02
**Ty-136**	-1.2	-5.29	-2.56	7.6	-13.58	-4.07	12.1	-0.82	-34.65	-20.83	-3.44	155.28	-10.47
**Ty-137**	1.01	27.91	-14.49	-18.05	5.65	-7.63	10.09	2.73	-37.49	-27.01	-9.95	164.5	0.25
**Ty-138**	-0.2	14.22	-5.16	-15.51	2.12	-8.72	2.97	-2.24	-31.42	-23.71	-14.78	141.7	-5.91
**Ty-139**	2.42	25.75	-8.21	-9.51	-1.79	-6.05	10.13	-0.32	-36.01	-28.16	-17.34	149.16	-4.71
**Ty-140**	0.01	15.73	-5.95	-7.51	13.98	-5.78	4.95	-6.45	-34.93	-18.38	-18.98	149.47	5.41
**Ty-141**	-0.2	-35.13	-16.78	-4.54	4.57	-3.12	3.69	-0.01	-26.52	-14.09	-13.71	155.43	-1.03
**Ty-142**	-0.4	-63.03	-29.54	-8.75	20.97	-6.07	-11.22	-4.22	-16.42	-18.38	-22.68	106.05	6.51
**Ty-144**	-1.2	-35.86	15.67	-0.82	12.61	65.88	-3.5	-9.25	-11.48	-20.74	-20.5	110.07	-9.39
**Ty-145**	-0.8	-22	5.03	72.68	89.17	5.17	-8.35	-8.84	-29.94	-33.1	-4.76	111.65	-7.34
**Ty-146**	86.83	-2.11	64.18	72.68	89.17	-9.68	3	-2.89	-29.28	-17.54	-5.05	47.46	-8.3
**Ty-147**	-0.2	24.95	-6.12	0.36	-0.76	5.54	-7.35	-7.13	-21.05	-12.91	-11.85	117.89	-4.21
**Ty-149**	0.61	6.7	-5.75	-4.62	6.83	4.04	3.6	-4.7	-33.33	-34.9	-9.05	129.38	-1.72
**Ty-150**	-1.41	24.43	9.27	-4.62	6.83	2.4	-0.96	-0.59	-57.64	-38.43	1.16	118.13	6.78
**Ty-151**	1.62	28.85	-11.34	0.36	-0.76	4.03	3.72	-6.6	-46.84	-33.79	-18.06	128.26	-2.69
**Ty-152**	-2.21	21.01	-16.84	-5.38	-24.31	-1.43	5.64	-4.39	-49.49	-41.62	-1.67	133.36	-6.88
**Ty-153**	41.64	6.05	-47.82	2.48	2.61	-5.97	11.16	1.51	-36.41	-19.6	6.28	142.74	-1.94
**Ty-154**	-0.8	30.76	-18.74	-12.39	-6.4	1.87	4.83	2.12	-42.07	-34.21	-13.76	138	-9.63
**Ty-155**	0.21	20.22	-23.69	-16	0.69	4.76	2.33	1.04	-55.62	-23.23	-11.82	129.86	-5.42
**Ty-156**	-0.6	20.1	-9.97	-16.01	-7.92	17.13	6.66	4.27	-34.45	-36.51	-17	143.73	6.93
**Ty-157**	-0.2	24.58	8.26	-15.95	2.33	3.27	-3.71	-0.51	-49.54	-38.17	-9.47	163.23	-5.37
**Ty-158**	1.21	-4.02	-28.43	-4.61	14.33	-13.84	7	0.14	2.79	-16.48	48.46	156.83	-10.17
**Ty-159**	0.41	26.52	36.59	-5.21	7.35	24.26	28.07	7.81	22.21	-17.59	29.53	151.12	1.81
**Ty-160**	0.01	0.31	-4.95	0.39	31.1	-1.28	-3.11	0.57	-43.73	-26.71	-10.65	149.95	3.06

Molecule	*T. brucei*	*L. major*	*T. cruzi*	*ss18*	*h37rv*	*h*ERG	CYP1A2	CYP2C9	CYP2C19	CYP2D6	CYP3A4	A549	Mitochondrial toxicity
**Ty-161**	0.01	-0.28	-10.51	-1.62	0.47	-11.35	27.18	19.91	65.73	22.32	95.33	154.13	-3.39
**Ty-162**	1.01	-7.48	-3.53	-0.49	22.66	1.49	5.89	1.52	-34.57	-22.55	-16.43	154.41	10.69
**Ty-163**	0.01	-7.01	21.43	6.34	11.92	2.97	-3.81	-5.01	-27.76	-21.61	-23.23	167.9	-4.02
**Ty-164**	-1.4	-51.37	-29.24	18.33	8.63	2.99	1.74	-1.58	-20.35	-11.38	-13.51	157.73	-0.9
**Ty-165**	2.02	-45.21	3.68	7.67	-3.11	8.35	-11.42	-5.69	-14.64	-28.1	-25.42	112.05	-0.01
**Ty-166**	1.22	-0.77	18.46	-5.22	16.06	2.17	-3.55	-5.77	-23.69	-25.68	2.6	114.56	-6.42
**Ty-167**	1.62	19.8	-1.54	-1.48	-6.57	3.45	2.61	-3.46	-28.35	-11.83	-13.22	119.55	0.6
**Ty-168**	-0.8	-74.13	73.41	27.58	-11.93	1.99	0.77	-8.62	-49.03	-54.69	-11.65	126.65	-6.98
**Ty-169**	0.41	24.71	8.06	2.75	-8.02	25.32	13.63	31.23	-6.13	-27.17	-12.65	132.5	2.92
**Ty-170**	0.01	-2.03	-28.67	-9.32	-1.83	-14.02	59.31	51.09	76.18	2.52	75.08	115.03	-6.71
**Ty-171**	-0.4	19.37	-13.22	12.31	-11.46	-4.04	8.91	2.61	-40.32	-37.03	-13.64	126.45	-4.63
**Ty-172**	-0.4	13.1	49.75	5.2	-17.07	4.27	31.32	24.24	40.42	-3.95	-17.93	126.86	-1.67
**Ty-173**	105.52	20.62	66.93	-10.61	-3.07	18.08	1.17	24.83	-7.81	102.94	6.55	122.57	-11.15
**Ty-174**	54.49	-14.54	2.46	-2.09	14.34	-12.33	-6.81	-2.96	-20.54	-16.51	6.76	107.23	-5.64
**Ty-175**	64.29	14.95	25.57	39.67	-2.3	27.78	38.36	16.23	-12.57	-13.19	1.36	135.33	-5.19
**Ty-176**	69.81	-13.26	3.83	5.04	8.26	-2.28	33.43	3.6	-23.43	-12.52	5.11	84	-4.61
**Ty-177**	47.32	-28.84	10.38	15.83	-21.87	21.66	61.77	38.13	89.93	18.98	81.87	146.31	-1.62
**Ty-179**	121.81	12.22	-0.69	23.21	15.5	21.03	14.02	50.93	33.73	47.84	41.99	151.1	5.07
**Ty-180**	0.41	26.25	2.4	17.13	15.21	0.32	5.12	3.4	-37.6	-28.71	-8.16	146.22	-6.31
**Ty-181**	-0.2	5.57	16.46	-8.96	-22.14	-1.83	48.17	66.15	65.14	-2.24	-13.5	154.19	-2.5
**Ty-201**	48.32	20.06	23.48	-8.05	2.06	-8.88	13.87	58.97	10.46	96.05	14.98	157.51	-6.16
**Ty-203**	-1.2	-24.35	-9.26	6.83	3.39	-3.01	1.55	-7.5	-18.77	-24.29	-33.97	104.1	-2.22
**Ty-204**	1.62	8.63	36.01	7.94	6.99	3.27	-0.93	-3.76	8.33	-20.07	17.57	157.88	-9.35
**Ty-206**	-0.6	36.62	-51.54	3.24	4.41	-5.78	9.19	0.58	-18.06	-25.87	-0.5	147.58	8.86
**Ty-209**	0.21	19.92	-22.76	11.29	20.46	13.34	7.41	0.94	-15.66	-29.25	7.81	127.14	-2.79
**Ty-210**	0.21	-30.63	-2.88	7.72	3.93	6.95	21.98	10.87	20.57	-42.31	-24.19	107.25	-4.13
**Ty-212**	-0.4	35.13	-30.09	6.41	18.49	-4.03	43.57	3.41	42.92	38.61	20.7	154.93	6.62
**Ty-215**	-0.8	-26.17	-28.79	8.4	23.81	-37.09	80.15	-1.64	62.17	79.46	13.72	156.35	6.8

Molecule	*T. brucei*	*L. major*	*T. cruzi*	*ss18*	*h37rv*	*h*ERG	CYP1A2	CYP2C9	CYP2C19	CYP2D6	CYP3A4	A549	Mitochondrial toxicity
**Ty-216**	-0.8	-3.9	14.8	7.66	18.48	2.63	66.31	6.36	35.89	-12.26	-0.22	120.68	-5.6
**Ty-217**	1.21	-2.41	51.13	14.3	22.08	-4.54	37.07	1.58	-14.99	-27.17	-4.38	120.85	8.02
**Ty-218**	0.81	14.3	9.51	-8.52	-0.73	-16.21	5.37	4.35	-23.94	-28.56	-10.76	145.17	6.43
**Ty-219**	60.65	-24.93	-15.4	14.06	2.05	2.06	5.04	-14.29	-11.86	-31.27	-1.01	112.89	-3.38
**Ty-220**	39.16	34.18	-7.32	29.19	-23.81	1.04	-1.51	-10.4	-30.14	-29.34	-3.74	119.96	1.37
**Ty-221**	91.38	39.54	39.22	6.8	-3.97	-6.86	33.81	8.26	50.29	-33.75	-18.03	131.94	1.65
**Ty-222**	84.84	23.49	17.54	-11.23	7.22	-1.79	19.17	7.25	-27.44	-32.77	-9.26	121.22	3.8
**Ty-223**	-0.2	14.79	-3.37	-9.73	-5.26	-9.99	18.18	-1.51	-34.8	-22.81	1.11	126.18	-9.75
**Ty-224**	40.73	-7.78	25.98	-4.03	6.03	-4.76	17.42	-3.78	-28.31	-20.07	-15.22	159.85	1.22
**Ty-225**	5.05	15.49	12	-8.79	19.35	-5.53	26.38	-0.22	-32.21	-44.49	-18.37	143.24	1.3
**Ty-226**	82.08	-57.49	77.28	-5.14	14.78	8.48	44.37	1.94	32.14	-2.18	-23.03	104.8	-4.83
**Ty-227**	8.21	12.18	17.22	-3.54	1.74	-11.16	43.96	19.39	-26.67	-22.91	-2.79	118.76	-4.08
**Ty-228**	0.21	9.14	44.93	-3.84	5.01	-13.62	24.35	0.23	5.22	-29.9	-8.53	142.21	-2.66
**Ty-229**	0.41	16.22	-4.05	2.5	-2.62	-5.03	32.41	7.58	-26.34	-23.9	-1.8	152.87	-0.8
**Ty-230**	87.91	38.95	33.19	2.26	23.64	-5.41	31.93	0.62	-10.18	-21.75	1.2	146.31	-7.93
**Ty-231**	83.24	15.68	21.62	3.05	10.83	0.56	10.84	0.64	-22.9	-25.04	1.91	111.57	3.41
**Ty-232**	23.4	8.03	-1.56	5.78	3.87	-9.03	15.05	8.79	-26.3	-28.86	-5.12	110.26	-0.96
**Ty-233**	-0.6	-34.87	81.52	14.57	6.96	1.74	53.09	35.87	74.54	-0.99	-5.87	155.82	16.5
**Ty-234**	-1	11.24	25.01	2.97	-14.28	-4.81	55.28	33.11	8.24	-15.31	-7.43	138.79	3.77
**Ty-235**	69.46	24.64	40.59	0.03	-1.16	-2.37	46.61	36.44	-19.47	-28.94	8.15	123.35	-8.45
**Ty-236**	63	31.68	39.22	4.1	-11.62	-15.13	39.1	28.44	-20.28	-32.74	3.32	114.79	-4.15
**Ty-237**	0.41	14.78	17.43	14.35	7.88	-9.91	48.7	0.57	-31.95	-32.99	-13.5	150.51	-0.99
**Ty-238**	43.9	33.49	19.79	-17.53	-4.14	0.91	5.33	4.53	-31.93	-18.74	4.01	117.6	-0.13
**Ty-239**	1.62	22.93	18.38	-9.44	15.73	7.04	5.69	-0.47	-30.19	-29.72	-0.51	113.41	-6.57
**Ty-240**	-0.4	24.77	-1.66	-7.52	6.55	-0.78	0.48	-4.11	-35.69	-39.47	-8.39	125.37	-4.15
**Ty-241**	0.21	30.16	-10.38	-7.88	0.93	-7.44	0.38	-15.27	-34.26	-38.77	-0.01	122.81	-4.39
**Ty-242**	-0.2	8.55	-29.52	-6.28	-7.39	-35.87	-6.08	-48.86	-35.1	-34.71	-16.92	160.96	-4.35
**Ty-243**	-0.2	13.59	-0.03	-7.17	16.17	14.72	2.24	-13.48	-39.34	-19.79	-24.36	148.6	11.13

Molecule	*T. brucei*	*L. major*	*T. cruzi*	*ss18*	*h37rv*	*h*ERG	CYP1A2	CYP2C9	CYP2C19	CYP2D6	CYP3A4	A549	Mitochondrial toxicity
**Ty-244**	-0.8	44.25	-0.69	-1.12	12.75	7.85	4.65	-44.76	-35.74	-36.63	-10.11	155.34	-5.8
**Ty-245**	1.21	23.71	-6.21	-1.44	8.96	-3.73	8.14	-352.75	-44.8	-36.14	-6.41	146.6	-0.23
**Ty-246**	1.21	28.38	-49.85	2.33	12.84	-9.56	1.9	-14.22	-41.73	-42.65	-6.4	145.91	-7.88
**Ty-247**	-0.19	19.75	-0.15	22.21	21.82	-4	6.78	-8.6	-42.84	-30.85	-3.42	132.84	-3.53
**Ty-248**	-1.6	-12.83	-4.31	11.81	4.56	-0.63	-4.55	-50.19	-28.36	-52.55	-20.55	119.04	-0.01
**Ty-249**	0.81	-6.09	-12.41	18.31	9.44	3.19	4.57	-452.94	-40.86	-36.9	-2.93	131.38	-6.6
**Ty-250**	24.67	32.84	56.29	11.11	-11.47	0.53	6.36	-338.08	-20.74	-35.49	3.62	118.85	-4.28
**Ty-251**	106.71	-98.44	91.49	59.22	-65.74	1.24	49.31	22	25.05	6.6	0.09	145.95	12.54
**Ty-252**	0.61	-1.72	-9.76	-4.08	-26.34	-4.21	-0.15	-10.79	-54.25	-32.68	-12.25	115.57	0.23
**Ty-253**	1.21	27.4	-12.83	4.8	6.25	-8.45	5.61	-2.07	-31.9	-35.14	-5.22	135.83	-7.53
**Ty-254**	-0.39	31.65	-8.44	-11.41	12.8	-2.59	10.86	-68.45	-37.29	-30.95	-10.42	164.04	5.73
**Ty-255**	0.41	-99.12	-4.45	-7.1	10.57	3.42	-5.86	-33.74	-13.63	-27.27	-18.04	114.05	-3.62
**Ty-256**	-0.2	-65.31	-25.86	-6.68	-3.67	-7.3	2.15	-1.18	-21.04	-34.66	-26.07	163	4.58
**Ty-257**	1.01	15.25	2.2	-8.22	16.02	-2.02	3.5	-19.97	-35.19	-37.05	2.26	137.67	-7.77
**Ty-258**	-0.4	23.4	-5.85	-4.21	0.21	-6.12	-4.65	-5.58	-26.1	-46.47	-12.56	127.96	-7.96
**Ty-259**	9.09	19.89	23.95	9.09	10.83	-1.12	64.14	26.39	86.98	-13.18	0.67	124.37	1.01
**Ty-260**	46.61	30.15	53.3	-3.5	1.88	-3.02	59.3	21.19	46.05	-7.05	-30.07	140.11	3.67
**Ty-261**	0.21	-18.89	50.12	3.31	3.96	-1.13	58.66	46.92	80.03	21.13	-9.66	122.35	-0.25
**Ty-262**	-1	14.22	23.56	0.14	23	-9.41	53.4	-215.69	-12.86	-23.61	-17.24	164.37	3.97
**Ty-263**	-1.2	-54.07	-13.2	12.75	4.41	-11.8	41.23	-233.95	25.87	-16.97	-3.47	165.99	-0.39
**Ty-264**	0.81	9.05	33.22	9.33	-0.8	-4.29	52.65	-354.25	76.18	-3.91	-13.64	137.95	-2.81
**Ty-265**	23.28	-39.68	34.31	10.84	6.39	4.58	60.06	-151.55	73.97	-24.38	-17.72	119.64	8.55
**Ty-266**	-2.01	8.6	24.76	8.06	3.79	-4.32	24.9	-82.45	43.21	-23.58	-11.3	158.34	-2.66
**Ty-267**	-1	45.37	-6.1	13.47	1.57	17.57	3.58	-3.35	-45.3	-26.42	-18.59	148.67	-0.61
**Ty-268**	20.03	-8.77	-17.94	9.95	-7.2	4.05	6.87	-6.37	-36.4	-19.05	-6.47	137.41	-15.45
**Ty-269**	-0.19	1.15	3.02	-0.01	-15.8	-4.69	7.23	-2.25	-32.12	-24.14	-24.51	162.74	-8.18
**Ty-270**	-1.4	9.18	-22.76	-9.94	12.16	-7.12	10.3	-2.99	-34.46	-26.99	-13.46	141.96	-4.85
**Ty-271**	-1.61	-7.86	-15.01	-10.48	-8.72	7.65	50.53	2.04	-35.72	-40.29	-9.95	130.22	0.83

Molecule	*T. brucei*	*L. major*	*T. cruzi*	*ss18*	*h37rv*	*h*ERG	CYP1A2	CYP2C9	CYP2C19	CYP2D6	CYP3A4	A549	Mitochondrial toxicity
**Ty-272**	0.21	15.92	11.19	-5.23	3.35	7.09	71.61	24.41	-14.82	-29.4	3.74	126.39	-10.24
**Ty-273**	0	35.8	58.34	-12.89	-20.92	-38.85	71.23	77.78	27.02	-16.45	-15.49	143.08	-0.15
**Ty-274**	-0.2	29.94	-12.68	-12.33	1.08	-4.92	3.77	-10.07	-40.28	-25.39	-7.7	106.21	-2.1
**Ty-275**	0.81	15.79	-15.69	-11.4	-10.98	1.35	-2.74	-10.67	-26.5	-41.91	-10.02	113.24	10.22
**Ty-276**	0.21	0.4	-0.45	3.33	23.89	-2.24	31.98	-14.26	-8.28	-20.98	-11.11	156.06	6.4
**Ty-278**	75.12	-18.63	0.21	-5	18.51	-3.02	19.08	-41.83	-7.99	-1.03	47.53	108.6	2.36
**Ty-279**	27.89	30.01	12.89	-2.93	5.64	-1.43	29.73	-348.07	-8.8	-36	-7.74	136.19	-4
**Ty-280**	63.21	16.49	9.12	16.1	-6.54	-2.81	46.14	-382.95	-5.14	-28.71	-9.17	125.25	4.56
**Ty-281**	30.44	37.18	91.82	39.03	85.68	-1.7	29.86	-218.16	52.28	-11.12	7.05	127.16	-3.12
**Ty-301**	16.14	-40.12	34.03	15.89	16.94	13.43	54.68	-34.58	32.76	14.91	33.54	131.11	-22.18
**Ty-302**	-11.85	-27.21	12.29	-0.63	1.7	10.12	35.42	-290.46	49.58	1.6	17.4	108.6	-15.58
**Ty-303**	-13.28	-32.18	10.52	-4.36	15.99	9.21	12.3	-21.82	18.2	8.3	-4.48	124.88	-16.88
**Ty-304**	-15.68	-44.24	13.15	0.49	14.46	7.72	-1.71	-16.97	4.71	9.14	-0.93	115.24	-17.89
**Ty-305**	-4.71	-78.89	23.55	-3.21	-2.39	8.13	41.91	17.45	97.53	7.64	28.95	117.79	31.56
**Ty-306**	19.67	-41.04	42.87	-3.73	-15.4	0.81	34.61	101.13	55.69	-13.35	19.74	78.87	21.95
**Ty-307**	28.76	-37.84	17.15	-7.49	2.78	-10.19	48.51	-38.47	89.41	26.93	24.36	102.68	-9.06
**Ty-308**	22	5.32	24.51	-4.86	-2.43	-5.98	69.52	-652.27	49.79	10.84	22.1	99.75	-20.04
**Ty-309**	19.2	-8.6	20.5	-10.58	-13.9	1.72	48.22	-339.41	30.56	23.18	31.11	100.79	-17.33
**Ty-310**	25.65	-23.95	9.65	-4.3	17.71	-1.42	39.12	-40.54	81.66	38.56	9.97	77.46	-21.26
**Ty-311**	-5.24	-23.6	11.03	47.24	10	-3.66	9.82	-10.16	0.57	1.42	2.31	107.88	-10.33
**Ty-312**	20.23	6.57	27	-3.75	9.77	0.01	7.4	-13.3	-10.56	-7.35	3.35	111.85	-23.52
**Ty-313**	9.62	15.06	17.74	2.92	7.93	-7.17	7.34	4.3	10.46	1.91	-2.87	118	-18.94
**Ty-314**	25.52	5.33	16.1	2.3	6.65	4.78	39.33	32.12	11.03	1.44	10.1	116.45	-22.01
**Ty-315**	16.84	16.51	1.77	7.74	-11.86	4.91	33.95	18.02	6.68	5.51	-8.68	123.65	-18.54
**Ty-316**	14.58	14.81	15.17	23.43	-3.37	3.01	21.39	1.66	98.03	11	13.83	119.07	-27.78
**Ty-317**	3.13	26.29	15.68	10.25	-2.93	3.5	32.11	26.53	18.32	23.52	-2.9	125.02	-16.41
**Ty-319**	8.63	20.65	16.9	6.4	-2.63	8.98	49.04	55.63	58.03	22.63	14.13	125.12	-26.1
**Ty-320**	22.81	6.84	7.8	16.91	11.54	10.35	12.19	27.89	108.58	11.05	33.84	125.08	-14.63

Molecule	*T. brucei*	*L. major*	*T. cruzi*	*ss18*	*h37rv*	*h*ERG	CYP1A2	CYP2C9	CYP2C19	CYP2D6	CYP3A4	A549	Mitochondrial toxicity
**Ty-321**	21.75	-19.04	17.55	-1.38	-17.31	5.34	49.63	51.37	65.19	16.96	15.13	126.91	-15.61
**Ty-322**	83.59	-24.08	15.75	34.49	-4.47	17.84	31.38	49.66	110.32	20.96	32.64	100.06	4.75
**Ty-323**	11.66	-72.65	17.88	-6.83	-26.03	2.23	41.99	95.74	73.93	17.16	24.12	85.35	11.24
**Ty-324**	20.25	-31.74	50.88	1.22	12.76	-2.99	89.88	102.06	61.65	100.78	99.67	96.1	-17.01
**Ty-325**	18.34	-16.03	17.19	-1.54	14.05	-11.41	24.05	19.16	14.64	14.17	19.62	111.64	-14.49
**Ty-326**	26.86	-10.08	18.38	0.46	-0.52	-11.91	57.57	9.23	72.67	21.3	31.17	102.92	-13.74
**Ty-327**	34.16	-49.61	-1.81	-2.2	13.88	1.97	63.71	9.97	46.18	29.05	21.23	108.11	-11.91
**Ty-328**	47.77	-14.54	12.02	-10.75	-7.32	-9.56	61.26	66.01	31.93	15.99	26.75	130.57	-10.04
**Ty-330**	51.36	-5.21	16.39	7.27	-5.42	-10.09	71.99	63.92	23.06	14.6	33.26	112.64	-23.49
**Ty-331**	30.23	-14.45	3.2	20.31	0.84	-5.04	63.65	60.1	19.58	10.15	11.1	125.8	-12.44
**Ty-332**	32.41	7.29	-1.04	1.43	21.59	1.87	45.44	10.66	86.87	14.98	12.09	123.31	-16
**Ty-333**	17.74	-27.44	11.03	19.11	-11.59	18.07	41.91	43.19	93.58	20.16	33.84	125.87	-20.06
**Ty-334**	97.61	-13.32	7.87	0.6	-36.9	-1.19	65.16	78.6	63.41	16.27	25.18	120.76	-23.59
**Ty-335**	39.85	-32.59	15.27	5.5	5.16	4.88	58.59	74.5	30.08	7.7	34.36	118.64	-12.58
**Ty-336**	35.31	-14.54	6.51	13.12	-20.48	13.72	23.66	22.89	103.27	9.43	32.72	108.33	-15.61
**Ty-337**	19.41	3.38	25.4	19.06	5.31	33.36	20.16	32.68	102.77	7.9	21.02	115	-16.4
**Ty-338**	23.57	-41.44	10.16	-5.69	-19.05	4.36	35.96	100.86	77.08	9.85	23.31	126.01	-19.4
**Ty-339**	8.03	-48.33	18.57	-12.36	2.64	1.4	51.4	13.11	98.73	19.64	2.8	123.48	4.34
**Ty-340**	19.32	-55.95	40.46	-13.59	-0.22	-1.73	86.84	20.1	77.05	7.87	15.11	65.76	29.81
**Ty-341**	36.13	-20.88	4.62	-14.42	-3.95	1.85	80.89	34.53	78.62	14.49	14	80.2	-9.62
**Ty-342**	99.86	40.38	48.82	9.51	14.24	5.19	23.73	91.42	106.83	34.26	65.66	102.03	-12.17
**Ty-343**	36.97	-2.62	6.65	-13.49	-10.74	-3.94	53.94	28.28	107.48	21.05	19.21	93.41	-17.76
**Ty-344**	36.67	1.8	22.5	-0.6	-5.15	-14.98	22.61	19.31	-10.4	12.94	12.99	90.39	-10.9
**Ty-346**	31.15	29.96	16.36	5.11	2.51	-5.33	20.18	54.03	26.11	15.34	38.29	114.61	-13.08
**Ty-347**	31.3	23.8	31.65	18.13	4.01	10.31	22.69	53.39	11.56	19.85	49.14	120.82	-15.89
**Ty-348**	96.91	43.44	48.07	16.47	5.8	21.95	29.46	86.01	97.85	16.41	81.18	80.73	-17.56
**Ty-349**	93.28	46.15	37.27	20.97	14.5	2.39	58.72	13.63	58.98	18.5	57.54	113.76	-14.35
**Ty-350**	98.02	61.45	59.48	39.88	-10.61	7.79	45.86	84.58	71.62	18.36	72.94	116.65	-12.46

Molecule	*T. brucei*	*L. major*	*T. cruzi*	*ss18*	*h37rv*	*h*ERG	CYP1A2	CYP2C9	CYP2C19	CYP2D6	CYP3A4	A549	Mitochondrial toxicity
**Ty-351**	98.04	32.06	62.39	12.93	10.19	0.63	72.5	71.54	86.61	20.12	62.67	115.96	-12.35
**Ty-352**	97.45	56.67	72.05	3.41	-9.13	-4.36	27.05	59.85	87.22	16.47	77.57	123.52	-23.63
**Ty-353**	28.75	-5.14	14.62	36.82	22.61	42.09	23.76	66.15	103.52	25.23	80.98	114.66	-7.09
**Ty-354**	29.07	9.58	0.72	-20.06	-10.76	-3.18	14.98	7.98	21.12	4.26	-10.97	121.33	-21.85
**Ty-355**	22.98	-12.99	7.85	0.46	-0.52	-5.5	27.04	-2.14	44.66	13.5	-0.35	120.07	-17.31
**Ty-356**	19.25	-33.91	17.45	-10.75	-7.32	-3.13	18.62	9.29	17.65	14.02	1.97	120.9	8.59
**Ty-357**	4.89	-51.03	32.34	1.43	21.59	-16.21	4.05	-15.59	53.23	0.18	-11.71	89.34	12.64
**Ty-358**	5.02	-8.87	10.32	14.84	-4.21	-13.47	26.14	5.56	4.43	1.07	7.94	105.3	-9.74
**Ty-359**	14.45	-15.2	8.61	-4.28	-6.3	-8.76	22.59	9.74	6.94	6.83	8.04	85.07	-12.31
**Ty-360**	21.06	-42.82	-1.99	-7.94	13.66	-9.36	49.25	40.58	23.44	21.1	43.72	110.78	-18.17
**Ty-361**	20.52	11.91	-0.24	-3.13	3.51	-4.58	45.43	47.26	17.05	15.06	45.87	74.53	-9.87
**Ty-362**	5.8	26.56	-1.84	6.21	18.42	-5.16	23.88	13.3	10.35	15.19	8.4	88.31	-14.54
**Ty-363**	32.95	-1.27	-5.35	18.94	6.76	-6.17	22.13	13.05	8.83	8.06	15.37	74.83	-16.03
**Ty-364**	22.37	4.87	2.46	18.44	1.35	-2.84	22.34	10.81	4.38	0.67	12.23	112.57	-14.9
**Ty-365**	16.44	32.73	-13.41	23.94	22.26	-8.51	26.68	13.75	15.45	15.27	2.38	120.05	-18.22
**Ty-366**	96.58	15.19	-5.06	-1.12	11.41	1.99	38.03	18.78	7.18	15.93	7.03	141.59	-6.96
**Ty-367**	28.51	10.14	2.76	20.78	-17.19	-8.12	20.39	-179.44	33.91	8.89	19.3	119.87	-16.33
**Ty-368**	12.67	9.99	-6.64	15.24	-4.44	-4.41	21.92	15.21	14.34	3.65	7.3	148.57	-18.75
**Ty-369**	22.91	23.7	2.43	7.71	1.25	-2.86	74.59	-3.73	26.41	13.08	3.64	117.46	-11.98
**Ty-370**	14.76	24.12	-4.71	-8.1	25.63	5.05	27.22	10.9	11.34	53.02	-14.35	133.84	-15.39
**Ty-371**	-4.92	-3.29	7.24	-3.36	14.07	-4.51	9.8	3.19	1.96	6.73	-16.24	118.49	-13.26
**Ty-372**	-2.14	-60.57	11.78	-4.93	1.39	-5.81	7.8	3.27	-0.37	4.68	-7.34	128.2	-2.28
**Ty-373**	29.02	-55.07	21.63	-7.6	-7.07	-13.86	6.27	2.55	1.84	-6.62	-14.75	91.97	33.41
**Ty-374**	28.11	-37.46	11.73	-11.05	-1.03	-8.45	17.82	18.53	10.31	-1.49	-1.72	94.61	-11.6
**Ty-375**	43.75	-6.23	8.45	-0.67	19.08	-9.82	23.27	9.39	14.03	18.7	13.89	119.79	-9.03
**Ty-376**	52.42	18.15	-12.97	-8.55	24.82	-11.81	26.58	12.84	12.69	17.59	4.11	106.43	-15.65
**Ty-377**	44.39	11.03	-6.78	-0.09	-0.9	-17.88	26.75	13.6	17.66	12.89	0.64	113.7	-17.5
**Ty-378**	57.21	1.69	-10.63	21.48	15.14	-4.51	25.29	15.16	17.38	18.07	-9.24	98.52	-11.21

Molecule	*T. brucei*	*L. major*	*T. cruzi*	*ss18*	*h37rv*	*h*ERG	CYP1A2	CYP2C9	CYP2C19	CYP2D6	CYP3A4	A549	Mitochondrial toxicity
**Ty-379**	44.21	12.65	-5.74	18.96	19.12	1.76	45.05	20.67	103.33	7.47	20.7	118.2	-15.67
**Ty-380**	-8.79	9.35	12.55	25.04	22.21	311.97	27.35	18.61	22.1	9.77	16.29	128.11	-2.37
**Ty-381**	55.9	6.37	-0.86	16.91	6.05	4.73	34.9	48.27	24.62	15.87	-4.92	110.76	-11.41
**Ty-401**	50.72	27.45	-2.49	0.14	-4.02	-3.34	63.87	29.86	17.89	18.83	7.31	153.5	-13.71
**Ty-402**	50.62	-3.03	-4.98	-9.51	-0.56	-1.92	66.58	72.68	45.66	19.54	10.33	128.24	-16.98
**Ty-403**	60.76	4.65	2.79	-0.73	4.94	5.09	30.74	41.91	22.42	28.18	54.75	112.16	-15.34
**Ty-404**	50.31	16.99	3.13	-0.16	6	-5.42	18.77	20.14	14.94	58.13	7.25	136.8	-17.82
**Ty-405**	70.81	15	1.45	-9.48	0.06	-1.77	14.73	3.68	-0.13	7.15	-8.8	130.93	-12.12
**Ty-406**	45.41	-8	2.77	-9.99	0.63	-7.3	9.85	-1.58	2.57	8.43	-13.01	139.31	-11.55
**Ty-407**	59.4	-104.51	15.34	-1.98	3.09	2.18	35.07	6.46	57.84	9.47	-3.54	127.45	1.14
**Ty-408**	26.33	-71.65	24.33	-17.87	-0.34	-17.39	10.26	2.4	-2.01	13.23	-8.92	61.31	6.18
**Ty-409**	30.36	-93.51	30.16	-8.52	0.72	-10.55	48.42	27.13	14.86	17.97	37.75	103.95	-13.6
**Ty-410**	22.06	-12.17	8.78	0.26	5.85	-7.71	32.44	26.59	17.95	29.39	21.89	113.43	-11.38
**Ty-411**	46.13	-10.48	-6.41	-16	18.5	-6.05	29.08	20.14	15.32	24.11	-4.38	117.26	-11.31
**Ty-414**	35.31	8.74	-9.41	4.23	21.52	-14.69	29.36	19.42	13.24	6.23	-8.99	97.26	-14.56
**Ty-415**	30.21	-9.85	-7.06	11.04	19.41	-8.71	29.5	6.33	41.09	14.34	30.97	122.83	-16.55
**Ty-416**	42.53	21.11	-8.77	13.2	21.19	-2.93	28.3	14.43	11.27	19.07	15.25	139.3	-15.47
**Ty-417**	52.27	-7.86	-17.91	5.58	36	-2.55	24.86	12.44	8.26	8.84	-19.94	136.47	-14.42
**Ty-418**	30.56	0.2	-0.5	-9.61	25.38	-1.52	23.28	29.55	57.46	23.54	45.88	124.39	-21.18
**Ty-419**	41.25	10.9	-3.14	-1.25	-11.28	-13.23	24.61	14.56	15.86	9.65	20.32	130.4	-13.48
**Ty-420**	32.57	10.42	-0.8	66.19	78.01	-7.38	19.71	23.77	6.76	8.06	-13.42	135.52	-9.44
**Ty-421**	35.22	-2.09	0.24	3.63	3.44	-8.95	17.87	5.08	-0.21	1.35	-13.54	113.52	-11.8
**Ty-422**	18.23	22.52	8.1	-2.74	-2.67	-3.18	10.52	-80.07	87.4	17.48	19.07	109.72	-17.6
**Ty-423**	27.97	-9.52	6.54	43.41	14.4	-1.93	6.96	85.72	16.24	6.84	14.97	145.53	34.18
**Ty-424**	8.52	-32.03	41.06	-10.03	-0.78	-18.63	7.45	-49.82	3.16	-0.56	-18.76	84.58	24.25
**Ty-425**	19.92	-14.89	-7.97	12.02	-17.73	-15.86	19.25	60.77	10.8	17.21	-21.7	71.49	-13.83
**Ty-426**	74.03	-12.62	-2.88	2.59	-12.65	-18.31	22.7	-262.06	23.02	7.58	8.25	83.49	-14.13
**Ty-427**	31.13	-0.29	2.63	-8.22	9.71	-20.31	25.73	13.66	36.72	15.11	23.11	76.4	-11.99

Molecule	*T. brucei*	*L. major*	*T. cruzi*	*ss18*	*h37rv*	*h*ERG	CYP1A2	CYP2C9	CYP2C19	CYP2D6	CYP3A4	A549	Mitochondrial toxicity
**Ty-428**	29.58	8.96	-3.58	-16.52	5.87	-5.16	27.49	50.97	83.83	75.31	36.25	109.31	-25.07
**Ty-429**	23.27	32	0.84	-5.77	5.13	-8.86	28	13.34	14.83	3.42	-9.67	113.27	-15.74
**Ty-430**	39.75	13.34	-5.58	-10.64	-0.37	-0.87	29.21	9.3	9.55	6.25	-15.4	115.34	-10.86
**Ty-431**	22.75	14.68	-7.06	-3.5	3.02	-13.71	28.13	-4.98	52.58	8.43	-7.34	125.66	-10.6
**Ty-432**	44.68	22.01	1.71	22.84	-4.86	6.59	28.7	29.32	11.27	14.05	-7.42	116.28	-11.17
**Ty-433**	21	-13.64	-8.28	20.9	20	-5.22	24.02	44.2	15.54	12.85	-11.69	127.54	-12.26
**Ty-434**	31.37	-3.14	-6.37	-9.92	-15.51	5.1	26.57	11.81	15.95	12.74	21.23	110.75	-22.28
**Ty-435**	9.51	31.74	3.22	6.97	-21.88	-0.7	25.48	49.17	21.92	14.79	17.97	121.89	-19.01
**Ty-436**	33.37	4.86	8	-10.96	-18.78	3.32	26.6	102.94	32.87	22.63	4.58	136.39	-13.66
**Ty-437**	100.15	10.99	1.51	-2.87	68.79	33.76	17.59	40.15	10.09	12.5	-5.31	130.04	-15.52
**Ty-438**	12.35	8.91	4.1	-2.25	-10.78	1.45	7.78	100.71	22.6	5.74	28.35	122.98	-8.2
**Ty-439**	21.67	-31.97	0.75	-16.43	-47.85	-1.42	3.66	91.3	6.97	4.85	-12.14	144.45	10.41
**Ty-440**	30.54	-24.86	32.06	-24.67	-7.55	-17.69	5.07	92.44	3.02	-3.13	-4.39	89.44	8.99
**Ty-442**	30.36	49.56	5.52	-20.02	1.62	-15.07	18.22	7.73	3.54	0.28	-8.92	113.34	-13.45
**Ty-443**	22.73	29.63	0.58	-16.09	13.24	-6.75	22.3	11.79	12.33	21.58	-1.91	102.41	-13.72
**Ty-444**	30.06	27.74	0.46	-20.98	8.4	-23.46	29.61	30.1	29.43	15.11	56.85	86.09	-16.79
**Ty-446**	27.97	24.99	-0.73	-2.5	-0.54	-18.2	26.37	10.38	10.63	2.93	-21.2	113.82	-17.8
**Ty-447**	34.61	17.21	-4.02	48.81	82.06	-14.37	30.92	25.84	12.35	5.65	15.41	112.15	-9.24
**Ty-449**	2.23	-3.87	-8.86	-8.13	3.36	-14.03	29.86	12.87	5.13	9.71	4.96	123.8	-18.14
**Ty-450**	35.88	15.19	-5.38	-2.28	13.92	-13.07	30.51	11.79	10.94	13.52	1.59	124.84	-18.92
**Ty-451**	44.64	18.4	-11.49	8	24.68	-7	30.5	16.54	18.03	14.66	-8.77	130.75	-2.84
**Ty-452**	17.81	19.98	1.39	4.58	18.65	-7.14	29.59	14.72	11.92	4.61	-5.68	121.93	-19.23
**Ty-453**	37.75	7.33	4.07	-18.4	7.62	-17.21	26.29	11.48	9.48	5.98	-9.22	120.43	-16.81
**Ty-454**	29.06	28.98	-7.68	-13.23	1.9	-14.97	20.23	22.32	5.49	4.46	-27.1	148.42	-16
**Ty-455**	30.06	22.83	-3.16	-4.67	-12.02	-8.84	18.29	6.19	3.55	0.02	-15.38	150.18	-19.51
**Ty-456**	18.01	30	6.09	-3.48	-0.1	-7.13	12.27	1.42	2.36	3.47	-1.16	120.5	-12.26
**Ty-457**	18.98	-44.26	6.77	-2.82	6.72	57.76	17.52	-0.7	18.09	18.25	-2.03	140.53	14.98
**Ty-458**	2.43	-14.86	10.47	-22.12	-9.19	-13.67	7.36	-10.76	-7.1	-7.42	-25.51	105.04	17.55

Molecule	*T. brucei*	*L. major*	*T. cruzi*	*ss18*	*h37rv*	*h*ERG	CYP1A2	CYP2C9	CYP2C19	CYP2D6	CYP3A4	A549	Mitochondrial toxicity
**Ty-459**	3.04	29.86	3.77	-11.8	-12.12	-11.37	19.14	4.85	6.67	3.57	-29.44	114.05	-12.11
**Ty-460**	21.44	21.96	0.57	-23.04	-7.2	-2.4	25.08	7.09	13.5	13.35	-2.18	113.44	-9.95
**Ty-461**	45.3	5.6	-8.37	46.58	27.49	-11.27	25.43	11.25	9.53	7.98	-1.87	97.13	-15
**Ty-462**	52.89	37.78	-9.97	-5.34	-4.04	12.49	26.45	14.01	14.74	4.87	1.4	100.97	-15.82
**Ty-464**	16.53	24.73	-4.3	7.38	5.15	-6.6	27.69	8.11	7.14	4.53	-17.84	111.25	-9.89
**Ty-465**	39.87	24.92	-6.77	-21.94	0.59	-14.97	26.01	10.21	5.42	6.01	-16.6	120.98	-14.43
**Ty-466**	37.98	13.17	-8.59	23.2	-16.35	-2.76	27.27	15.98	15.16	11.9	-11.95	115.1	-14.04
**Ty-467**	46.06	37.65	-16.18	-4.95	6.9	-9.03	28.22	13.64	9.14	1.45	-15.23	125.36	-11.84
**Ty-468**	27.59	-3.67	-9.55	-6.21	13.5	-10.79	28.5	13.97	11.4	5.24	-3.08	133.68	-18.88
**Ty-469**	27.21	27.36	-4.95	3.41	6.71	-88.21	23.85	10.1	6.24	7.96	-6.31	139.91	-12.58
**Ty-470**	29.85	23.27	-4.37	-5.37	6.08	-11.49	20.03	7.6	4.66	8.02	-7.06	144.95	-15.04
**Ty-471**	64.93	3.73	64.59	17.67	-2.73	63.73	36.27	31.82	76.96	61.22	25.22	123.71	-5.99
**Ty-472**	-10.61	18.15	3.81	0.17	-4.16	-11.13	37.66	-0.3	-0.68	-1.17	-16.51	127.65	-10.02
**Ty-473**	6.13	-41	16.21	-5.68	0.56	-11.15	6.41	-0.61	-1.02	6.54	-4.23	146.19	12.95
**Ty-474**	25.74	-51.63	34.76	-6.81	2.9	-0.35	9.08	-4.95	-3.75	-3.54	-29.8	104.25	5.91
**Ty-475**	27.58	0.91	11.24	-10.18	-10.43	-3.25	18.81	22.64	11.15	5.51	13.49	114.06	26.6
**Ty-476**	42.69	-9.28	2.27	-13.58	-40.14	-2.99	28.45	13.09	14.71	8.92	12.58	84.23	28.38
**Ty-477**	41.16	-15.37	-7.41	-18.71	-33.23	-7.35	44.28	12.23	13.85	5.25	-2.61	101.33	30.69
**Ty-478**	38.09	3.66	-8.52	-30.33	2.5	-5.33	28.17	12.01	6.66	4.83	0.54	95.4	-16.31
**Ty-479**	45.58	9.44	-12.52	-12.3	-0.53	-3.46	27.83	14.48	10.09	9.03	1.15	116.53	-15.24
**Ty-480**	35.88	30.87	-10.17	-16.59	2.59	1.96	28.98	12.08	12.67	5.97	-18.11	126.95	-9.45
**Ty-481**	40.44	6.1	2.07	-8.37	18.47	11.59	43.58	33.64	37.57	96.32	-3.19	101.84	-14.6
**Ty-501**	13.19	7.68	-19.41	-11.01	5.15	-24.54	28.06	16.74	23.24	8.47	-8.01	166.12	-10.12
**Ty-502**	-2.12	27.25	6.45	-2.04	15.39	-17.36	43.19	24.67	34.1	19.23	-0.71	127.15	-9.37
**Ty-503**	29.03	19.67	12.46	9.66	29.84	-35.78	25.99	4.84	18.82	7.01	-25.39	164.68	-12.53
**Ty-504**	17.44	45.35	14.67	8.18	4.55	-18.73	36.07	23.71	30.43	21.78	1.02	146.31	-26.05
**Ty-505**	36.97	27.95	22.99	21.64	0.74	-30.09	15.24	9.94	18.16	4.66	-7.12	167.55	-37.08
**Ty-506**	23.09	-33.81	17.46	-4.96	5.47	-3.12	16.87	11.7	50.98	14.08	29.43	150.48	-14.35

Molecule	*T. brucei*	*L. major*	*T. cruzi*	*ss18*	*h37rv*	*h*ERG	CYP1A2	CYP2C9	CYP2C19	CYP2D6	CYP3A4	A549	Mitochondrial toxicity
**Ty-507**	36.56	18.99	8.65	-12.15	-7.78	-23.55	19	8.12	23.07	4.05	14.9	110.55	-39.41
**Ty-508**	-6.4	36.8	9.18	-1.9	3.97	-0.92	23.87	10.61	26.99	3.69	9.69	112.43	-3.11
**Ty-509**	49.43	25.1	1.56	-12.47	20.11	3.38	28.14	15.43	28.23	7.87	2.36	126.62	-1.19
**Ty-510**	31.11	27.48	-1.72	-19.52	0.84	-4.44	30.33	22.32	28.26	12.29	4.28	112.64	12.67
**Ty-511**	41.68	22.57	-7.28	-17.83	-4.66	-11.08	29.93	22.76	34.01	9.36	-0.35	142.38	10.71
**Ty-512**	18.72	47.7	-9.74	-12.15	-7.78	-10.46	30.8	22.26	29.03	11.97	-14.54	147.96	7.23
**Ty-513**	14	23.34	5.21	-12.14	-0.66	-2.78	30.4	18.37	33.13	15.47	-6.31	140.43	18.77
**Ty-514**	2.61	29.94	-9.87	-8.49	12.09	-0.32	29.85	19.87	35.13	20.14	6.48	146.94	5.25
**Ty-515**	6.71	35.49	-11.49	-12.84	4.7	86.85	24.81	23	28.9	12.17	-4.53	163.13	1.84
**Ty-516**	-5.94	43.76	-9.69	-10.56	12.25	-16.57	27.13	22.65	34.64	13.52	-13.6	154.94	9.68
**Ty-517**	59.91	22.83	-1.77	-9.37	21.59	-8.7	45.62	22.34	46.88	26.32	6.78	149.86	-8.37
**Ty-518**	-0.33	23.68	15.37	50.69	19.66	-22.91	27.53	93.28	43.28	11.45	18.6	169.48	-5.28
**Ty-519**	31.97	19.92	1.06	8.39	13.63	-37	25.5	21.22	24.65	13.34	-1.67	150.22	-38.8
**Ty-520**	4.35	35.04	12.62	12.39	9.83	-39.01	19.01	14.07	16.81	4.97	-1.5	153.39	-8.67
**Ty-521**	53.2	23.39	18.28	9.54	4.55	-28.89	16.85	11.99	20.11	7.52	-8.2	169.16	-48.27
**Ty-522**	34.36	-31.36	8.67	-3.77	-0.45	-26.29	11.44	-11.77	2.38	-0.25	-11.58	131	-9.25
**Ty-523**	37.88	11.82	5.3	1.32	-4.44	-23.88	17.68	1.95	12.8	-4.36	15.35	117.92	-14.29
**Ty-524**	10.55	16.88	12.58	4.34	-7.97	-26.3	22.94	5.79	21.64	-3.58	14.41	128.59	-9.72
**Ty-525**	55.57	29.33	0.09	5.78	-7.01	-14.27	30.97	39.88	28.12	10.78	17.36	131.82	10.43
**Ty-526**	33.99	36.89	0.56	-18.04	20.33	-23.44	29.77	18.14	29.02	8.52	-1.51	135.03	20.85
**Ty-527**	28.57	31.4	-3.39	5.06	10.59	16.85	29.47	24.48	34.18	15.67	5.34	141.56	19.13
**Ty-528**	11.26	42.54	-1.83	48.43	40.3	-29.69	29.04	29.69	32.48	17.53	1.9	152.06	12.8
**Ty-529**	31.92	35.88	-10.76	-11.07	-7.34	-23.23	30.9	36.96	26.08	13.04	28.15	140.74	9.51
**Ty-531**	51.66	31.71	-18.19	24.48	-2.77	-24.46	28.43	41.04	29.33	14.38	-4.09	157.68	7.15
**Ty-532**	38.84	32.07	-29.15	44.7	62.6	-33.7	30.31	3.62	24.03	16	2.38	167.2	10.14
**Ty-533**	29.54	-14.35	-7.76	1.53	-2.55	-27.3	31.12	89.24	34.38	14.07	16.71	170.64	0.32
**Ty-535**	38.85	-2.37	-2.01	10.86	2.25	-27.12	32.37	29.53	24.89	18.31	-9.17	166.81	10.66
**Ty-536**	36.14	9.48	10.3	73.22	83.59	-7.24	25.24	22.08	21.47	14.08	-4.8	154.99	-34.82

Molecule	*T. brucei*	*L. major*	*T. cruzi*	*ss18*	*h37rv*	*h*ERG	CYP1A2	CYP2C9	CYP2C19	CYP2D6	CYP3A4	A549	Mitochondrial toxicity
**Ty-537**	80.89	32.97	7.73	76.5	84.5	-31.89	19.62	-32.89	20.94	8.23	12.46	169.25	-13.94
**Ty-538**	50.49	35.53	5.12	-4.3	-11.1	-3.98	23.85	73.18	14.87	14.9	12.62	175.23	-35.01
**Ty-539**	45.61	-16.83	18.22	-0.75	-2.95	-55.03	9.32	-0.26	4.25	-2.12	2.47	161.21	-10.08
**Ty-540**	33.45	29.29	4.45	10.49	3.53	-33.77	19.9	11.45	19.19	7.83	17.95	127.83	-21.3
**Ty-541**	29.69	43.29	16.85	-12.79	-3.52	-13.76	23.17	7.77	22.31	-3.65	13.6	119.58	-16.94
**Ty-542**	96.55	39.71	1.44	-9.82	20.01	141.45	29.67	19.33	28.74	16.37	17.6	124.31	-4.31
**Ty-543**	92.58	18.44	-1.46	-12.43	2.5	167.36	31.02	27.15	41.67	27.01	23.92	122.14	14.26
**Ty-544**	75.9	27.82	-28.04	-14.01	11.64	-2.27	30.31	24.54	31.42	12.86	21.97	137.91	22.31
**Ty-545**	48.68	42.21	-12.04	-7.48	4.22	-14.99	32.59	25.62	32.29	22.04	8.3	130.24	8.73
**Ty-547**	-11.1	40.1	3.06	-10.09	-4.69	-14	31.35	66.89	43.34	22.04	68.87	117.9	14.39
**Ty-548**	17.76	34.25	-10.91	-8.6	-11.34	-26.74	32.44	24.84	30.27	14.65	7.42	133.23	11.45
**Ty-549**	36.39	41.4	-12.97	-7.57	6.07	-3.64	32.57	23.89	26.22	10.8	0.93	147.3	11.91
**Ty-550**	86.47	77.65	1.57	-7.08	11.23	-21.21	32.92	39.28	44.54	33.32	7.8	133.99	10.02
**Ty-551**	96.86	75.28	0	-1.01	11.81	-26.86	37.29	32.53	55.42	44.17	22.06	155.7	10.52
**Ty-552**	-7.19	32.09	-4.14	13.02	20.34	-45.93	29.24	19.77	17.57	13.65	-8.21	152.68	2.89
**Ty-553**	76.71	36.29	-11.42	17.25	12.35	-38.64	28.05	18.36	19.7	15.23	14.61	147.34	-5.63
**Ty-554**	52.48	12.01	-11.11	3.62	4.32	-25.47	19.82	17.46	17.63	7.83	2.68	164.86	8.13
**Ty-555**	42.52	24.9	-0.7	3.76	11.23	-51.36	16.29	13.5	17.31	5.87	3.29	146.58	-4.13
**Ty-556**	41.87	-12.16	6.51	15.37	4.45	-42.23	13.89	44.57	15.38	8.26	20.04	140.68	-15.79
**Ty-557**	51.51	26.17	11.12	40.93	-19.61	-30.46	26.9	15.28	25.46	4.59	36.74	119.29	-5.84
**Ty-558**	97.56	56.86	20.24	-14.57	8.92	-18.26	28.38	19.92	30.63	5.26	23.4	125.62	3.19
**Ty-559**	54.89	35.54	15.43	-12.57	11.39	-5.98	28.49	19.07	30.78	9.29	52.49	117.9	10.94
**Ty-560**	30.4	29.8	-8.26	-17.13	23.54	-13.96	33.56	26.96	31.94	14.41	35.8	135.56	10.48
**Ty-561**	37.12	10.24	-7.98	-14.92	10	-14.97	31.96	26.41	32.97	11.64	19.59	137.78	10.28
**Ty-562**	1.97	38.16	-3.36	-11.32	6.91	-38.21	35.1	30.16	34.08	13.3	7.12	143	13.69
**Ty-563**	8.57	19.07	-2.39	-6.79	4.32	-20.47	47.26	41.57	35.28	21.5	16.11	148.2	11.03
**Ty-564**	1.15	33.99	-0.44	-5.74	19.6	-23.24	35.56	26.14	30.24	14.26	15.75	145.96	5.85
**Ty-565**	15.4	39.3	-6.96	-6.14	5.25	-39.16	36.06	26.25	27.41	8.49	9.66	127.96	4

Molecule	*T. brucei*	*L. major*	*T. cruzi*	*ss18*	*h37rv*	*h*ERG	CYP1A2	CYP2C9	CYP2C19	CYP2D6	CYP3A4	A549	Mitochondrial toxicity
**Ty-566**	9.08	44.6	-11.68	0.66	12.01	-37.15	36.68	22.81	32.06	19.42	19.1	168.68	11.16
**Ty-567**	7.68	-3.16	-13.86	12.39	15.45	-44.14	33.75	25.75	33.55	18.52	18.8	147.29	4.95
**Ty-568**	46.01	30.14	-4.78	18.84	27.73	-34.99	31.44	21.11	28.59	12	4.47	134.66	11.02
**Ty-569**	22.56	52.5	-1.13	10.46	-4.07	-51.85	28.89	30.46	24.53	10.24	4.91	162.56	12.57
**Ty-570**	97.03	41.56	-1.92	0.78	1.05	-51.29	27	28.29	26.76	9.63	26.28	157.28	7.03
**Ty-571**	13.67	32.72	8.23	5.64	-4.45	-34.02	20.25	26.33	20.24	21.93	9.64	158.18	4.49
**Ty-572**	89.93	-24.16	12.08	-0.28	8.98	-53.85	18.01	19.82	26.6	57.26	22.51	144.89	-4.26
**Ty-574**	7.21	23.22	5.33	-2.22	-0.02	18.64	22.65	29.58	37.48	44.78	28.6	124.97	-12.87
**Ty-575**	41.52	16.69	2.82	-21.76	7.68	5.48	27.68	39.92	43.48	48.54	26.88	138.21	6.1
**Ty-576**	74.47	31.03	-13.31	-16.06	-0.87	1.91	30.65	28.48	45.26	55.8	18.42	139.54	13.47
**Ty-577**	59.69	14.37	-5.34	-10.16	9.71	-3.45	33.28	30.27	45.58	19.89	25.89	144.13	3.94
**Ty-578**	63.3	46.79	-7.95	-9.62	1.68	24.59	38.29	26.05	36.71	15.25	8.3	144.97	2.21
**Ty-579**	3.39	28.17	1.16	-17.01	16.1	9.53	39.23	30.16	31.83	10.7	20.2	138.82	8.01
**Ty-580**	-8.51	33.76	-8.96	-12.75	5.32	12.99	38.9	29.03	34.04	14.48	23.99	150.09	8.6
**Ty-581**	16.88	47.4	-6.89	-6.08	8.42	-13.79	39.15	33.6	30.84	23.52	27.79	139.24	4.48
**Ty-601**	29.32	44.48	-29.08	1.26	13.45	17.91	35.64	58.73	40.17	46.72	35.39	130.45	7.64
**Ty-602**	5.68	24.17	-4.75	9.29	8.33	-2.38	31.76	27.04	31.73	6.75	-3.74	153.08	5.53
**Ty-603**	-2.25	37.75	-17.58	19.68	-12.94	-52.1	29.7	32.9	39.92	73.41	-5.83	142.61	6.61
**Ty-604**	65.44	19.49	3.6	-6.84	-18.39	39.85	31.11	56.16	57.95	90.41	54.92	136.4	16.32
**Ty-605**	12.41	44.62	4.25	-13.08	-0.37	0.7	26.28	33.65	38.27	55.58	29.49	151.31	11.51
**Ty-606**	28.44	0.74	-2.37	-11.38	-5.57	-15.73	19.85	22.86	41.01	29.9	14.78	152.25	8.35
**Ty-607**	78.18	-2.08	6.28	-11.56	-11.71	-1.12	16.35	24.28	31.53	46.6	9.59	134.99	0.86
**Ty-608**	55.58	0	8.94	-13.06	-8.95	-25.11	12.65	1.92	9.55	14.33	-21.36	103.03	-1.95
**Ty-609**	76.97	30.66	-0.74	-13.55	-13.44	5.57	20.1	15.68	41.44	95.95	-4.3	117.83	3.96
**Ty-610**	61.28	13.76	-20.53	-11.46	-1.31	32.94	27.39	63.78	38.55	80.7	18.38	115.99	18.1
**Ty-611**	14.55	-14.67	-13.28	-2.81	-4.11	-8.68	30.41	53.03	38.17	65.97	12.35	132.14	24.31
**Ty-612**	78.53	9.99	-33.31	-1.06	6.46	23.04	35.09	66.72	51.67	89.82	68.37	121.41	25.35
**Ty-613**	81.64	43.21	-29.3	-8.22	3.5	-3.49	35.64	32.35	28.07	9.39	-35.67	139.79	3.46

Molecule	*T. brucei*	*L. major*	*T. cruzi*	*ss18*	*h37rv*	*h*ERG	CYP1A2	CYP2C9	CYP2C19	CYP2D6	CYP3A4	A549	Mitochondrial toxicity
**Ty-614**	97.9	57.16	1.34	18.47	17.92	33.4	27.38	95.46	66.39	85.9	73.28	132.9	26.92
**Ty-615**	10.07	39.58	-9.83	16.56	18.37	11.26	36.05	46.69	34.62	90.21	37.92	114.67	4.43
**Ty-616**	66.95	38.41	-23.9	12.72	27.37	20.09	33.8	71.69	49.25	57.41	46.95	135.42	30.66
**Ty-617**	93.47	68.44	-18.1	2.84	11.5	25.41	32.1	50.01	61.43	92.37	57.33	124.81	17.53
**Ty-618**	35.18	23.39	-9.59	8.7	16.19	30.9	30.34	56.51	56.78	100.82	70.4	146.41	17.16
**Ty-620**	64.76	18.7	7.37	12.8	26.91	24.1	31.07	67.28	41.6	58.18	24.43	141.5	21.92
**Ty-621**	24.34	-84.51	-18.2	7.42	-8.6	-15.97	27.22	44.62	31.45	42.61	-10.53	146.09	9.15
**Ty-622**	28.48	41.76	-3.57	1.7	3.92	0.48	26.57	59.2	46.46	52.42	79.55	137.41	18.5
**Ty-623**	93.18	75.77	-4.02	4.52	-11.55	33.59	26.61	61.49	31.74	58.41	61.42	131.21	17.22
**Ty-624**	52.34	8.13	10.29	-0.78	0.69	-15.52	15.04	30.62	28.69	51.4	52.88	133.97	22.65
**Ty-625**	74.77	12.35	10.5	-6.76	-0.72	-7.67	17.81	32.39	49.37	46.64	25.7	117.9	-3.16
**Ty-626**	42.33	30.42	1.68	-10.33	18.23	-19.53	27.55	22.07	44.01	46.75	18.86	123.73	11.88
**Ty-627**	77.13	39.49	-8.58	-12.93	10.78	-3.97	49.32	-429.89	49.2	10.03	8.02	139.74	19
**Ty-628**	100.28	14.12	2.41	-7.42	4.94	-1.25	36.96	1.55	47.49	22.88	11.79	136.36	11.77
**Ty-630**	22.56	44.16	-18.2	-11.05	-1.18	28.67	37.91	33.51	40.32	12.84	-10.17	130.65	18.98
**Ty-631**	17.42	18.97	-9.4	-8.03	24.94	-3.13	39.63	32.25	6.82	21.49	-0.68	130.48	15.09
**Ty-632**	40.74	29.45	-5.46	-1.17	12.44	-4.6	43.17	-109.9	48.93	31.75	7.5	139.99	12.17
**Ty-633**	24.39	63.76	-6.13	4.34	1.37	-16.08	35.51	37.23	39.8	25.78	4.1	148.38	13.18
**Ty-634**	17.39	49.31	-17.91	6.33	11.74	-17.23	35.73	41.57	40.47	25.66	-8.73	151.08	8.46
**Ty-635**	17.53	5.1	-1.88	19.27	2.51	-5.54	33.21	30.05	35.32	14.51	-25.31	144.53	5.31
**Ty-636**	88.21	54.9	6.72	22.08	33.68	-26.93	37.02	34.25	39	26.75	-22.81	139.55	15.73
**Ty-637**	67.1	17.48	-3.47	-12.48	-9.85	-44.15	42.22	-353.04	38.13	16.87	-12.17	133.74	9.24
**Ty-638**	29.35	14.5	-7.79	6.37	17.01	-25.74	23.77	16.8	20.02	15.85	-8.83	139.81	8.62
**Ty-639**	37.03	29.06	-4.12	4.34	15.04	-43.3	20.17	16.08	20.41	10.16	-4.93	154.53	3.23
**Ty-640**	35.66	25.32	-3.29	-4.11	13.71	-41.7	18.9	15.12	17.38	-1.16	7.67	144.58	1.34
**Ty-641**	0.69	30.97	4.29	-9.29	-8.33	-28.41	28.04	18.79	18.59	1.93	1.61	138.57	6.59
**Ty-642**	32.93	16.51	-8.05	-3.3	15.15	-53.43	48.25	60.78	38.97	44.89	24.04	105.17	13.94
**Ty-643**	40.17	29.54	-12.71	-9.9	20.21	4.03	33.1	19.92	25.82	6.12	2.18	142.98	10.9

Molecule	*T. brucei*	*L. major*	*T. cruzi*	*ss18*	*h37rv*	*h*ERG	CYP1A2	CYP2C9	CYP2C19	CYP2D6	CYP3A4	A549	Mitochondrial toxicity
**Ty-644**	20.04	40.36	-7.2	-5.38	-13.28	-14.08	34.87	30.92	37.89	28.56	-0.72	146.15	11.56
**Ty-645**	96.64	28.98	-9.75	-0.75	7.45	244.69	44.37	42.3	63.87	42.56	2.28	147.04	10.08
**Ty-646**	4.08	34.05	-4.49	-15.43	13.31	-20.14	34.28	26.43	31.26	23.75	-9.21	153.91	22.32
**Ty-647**	-17.53	22.83	-3.97	-8.13	29.87	35.42	37.22	68.96	40.84	54.67	88.4	146.89	19.22
**Ty-648**	20.15	37.46	-11.76	-6.25	0.28	-11.3	35.27	33.18	41.51	22.55	18.61	153.8	8.87
**Ty-650**	41.17	-31.57	-6.35	18.92	5.28	44.82	37.55	79.02	55.01	76.04	97.64	149.57	15.53
**Ty-651**	28.26	-23.92	-14.55	23.78	21.09	-9.51	33.67	17.2	95.76	26.62	15.51	149.83	12.21
**Ty-652**	2.86	18.44	3.21	13.47	8.64	-12.54	28.27	-13.84	14.57	10.61	-18.33	161.08	13.51
**Ty-654**	49.68	11.99	4.37	2.22	-11.46	-26.49	19.53	20.02	16.41	21.41	-18.03	167.58	-0.76
**Ty-655**	-10.55	-19.35	32.54	11.47	-6.21	-40.53	14.38	61.95	32.4	39.31	52.74	125.06	-1.04
**Ty-656**	8.27	40.59	13.74	0.17	1.8	-33.81	22.67	23.25	26.16	8.07	15.11	117.06	-9.32
**Ty-657**	37.47	34.04	0.42	-14.47	12.75	-41.75	25.29	24.41	25.55	0.88	0.11	128.15	8.31
**Ty-658**	27.41	31.18	-16.16	-10.75	6.1	-31.35	28.5	28.16	34.66	8.47	2.83	122.78	8.04
**Ty-659**	90.8	15.13	-10.96	22.51	-2.4	-6.16	31.96	-285.52	78.43	7.33	40.23	116.5	19.39
**Ty-660**	24.03	22.57	-17.35	-4.85	20.72	1.41	31.63	23.5	31.34	4.78	-1.05	119.07	8.84
**Ty-661**	-8.11	34.03	-3.52	-9.55	-1.7	7.6	32.48	33	31.6	10.24	-10.12	121.95	16.36
**Ty-662**	47.42	31.43	-14.95	-9.62	1.68	-13.95	32.66	26.26	28.43	27.81	2.38	125.32	6.99
**Ty-663**	21.06	17.84	-7.63	-6.44	26.03	-12.69	38.99	36.95	65.67	29.24	-1.36	148.47	12.16
**Ty-664**	-3.43	35.99	-6.92	-6.23	5.26	-8.6	36.53	29.62	43.67	16.98	-16.6	132.28	20.23
**Ty-665**	37.1	31.38	-13.55	0.92	6.02	0.21	37.91	43.94	61.09	55.89	-11.39	144.69	6.88
**Ty-666**	39.36	32.22	0.37	15.75	20.21	15.83	30.59	32.43	62.35	33.66	5.26	142.68	20.67
**Ty-667**	98.87	48.22	44.83	24.03	21.43	12.04	28.23	24.79	71.78	29.21	42.49	115.78	28.31
**Ty-668**	97.45	63.72	-4.52	14.16	14.27	9.85	24.8	51.34	52.59	35.38	58.7	99.76	23.29
**Ty-669**	14.49	7.73	-7.62	16.1	4.56	-15.78	28.09	53.84	64.67	29.24	-4.76	156.56	8.6
**Ty-670**	99.45	13.44	28	3.13	-5.35	-25.24	19.14	51.43	31.44	26.46	35.14	113.97	6.18
**Ty-671**	97.33	67.02	78.73	9.97	-12.94	25.26	24.66	49.45	51.24	26.76	31.06	133.38	8.86
**Ty-672**	46.97	40.67	-10.53	-14.11	-4.8	4.76	30.3	51.13	54.13	15.84	56.78	121.51	28.78
**Ty-673**	63.72	34.11	-9.55	-10.81	7.93	-10.28	38.43	62.09	87.44	52.27	38.23	133.98	15.83

Molecule	*T. brucei*	*L. major*	*T. cruzi*	*ss18*	*h37rv*	*h*ERG	CYP1A2	CYP2C9	CYP2C19	CYP2D6	CYP3A4	A549	Mitochondrial toxicity
**Ty-674**	43.44	19.95	-23.44	-19.6	17.37	24.07	32.97	35.86	42.22	38.71	9.4	133.57	17.39
**Ty-675**	101.78	78.2	-24.15	4.33	10.41	11.23	38.12	59.04	52.81	44.31	62.44	118.93	37.36
**Ty-676**	100.51	59.15	-6.52	4.69	5.49	39.01	37.84	64.92	64.16	76.09	62.6	127.21	29.38
**Ty-677**	24.05	34.51	-24.26	-1.57	12.72	-30.61	38.12	57.41	50.37	86.59	72.32	126.12	19.33
**Ty-678**	106.35	100.23	-9.8	16.49	15.68	3.78	3.90	6.61	5.72	6.64	6.37	122.81	4.86
**Ty-679**	44.74	60.93	-2.44	0.06	15.3	42.55	37.68	56.59	65.83	82.92	68.39	130.64	31.05
**Ty-680**	47.9	-24.41	17.75	-4.67	16.98	30.46	34.05	56.54	43.4	64.52	26.59	134.03	25.06
**PEN**						18.7	>100	>100	>100	24.8	>100	>100	>100
**BZD**						>100	>100	>100	>100	77.8	98.2	>100	>100
**AmB**						>100	>100	>100	96	>100	>100	11.25	11.25
**MIL**						>100	>100	>100	>100	>100	>100	>100	>100

SD for all the assays are within ± 10% of each value. The assays were performed in duplicate.
PEN: Pentamidine; BZD: Beznidazole; AmB: Amphotericin B; MIL: Miltefosine.

Table S1. Data related to early in vitro activity and ADME-Tox profile for all compounds of the Ty-Box library.

Model	Target	Organism	Number of Actives	Number of Compounds	ROC	F1	Kappa	MCC	Domain of Compatibility
**CYP1A2**	CYP1A2	Human	35	437	0.9328	0.4648	0.3913	0.479	0.161
**CYP2C19**	CYP2C19	Human	74	437	0.8588	0.5408	0.4027	0.4575	0.161
**CYP2C9**	CYP2C9	Human	64	437	0.8863	0.6584	0.5852	0.6042	0.161
**CYP2D6**	CYP2D6	Human	37	437	0.8714	0.4559	0.3794	0.4441	0.161
**CYP3A4**	CYP3A4	Human	35	437	0.8577	0.448	0.376	0.4335	0.161
**Chagas Disease**	Whole cell	*Trypanosoma cruzi*	46	437	0.7902	0.3757	0.2594	0.3194	0.161
**Cytotoxicity**	Whole cell	Human lung carcinoma (A549)	107	437	0.6554	0.4628	0.1794	0.2303	0.161
**Human ERG**	Ether-a-go-go Related Gene (Kv11.1)	Human	39	437	0.7924	0.3462	0.2451	0.3002	0.161
**Leishmaniasis**	Whole cell	Leishmaniasis	48	437	0.8214	0.368	0.2316	0.3495	0.161
**Sleeping Sickness**	Whole cell	*Trypanosoma brucei*	46	437	0.7388	0.3418	0.2263	0.2598	0.161
**Mitochondrial toxicity**	Whole cell		39	437	0.6748	0.239	0.104	0.1779	0.161
**Tuberculosis (H37Rv)**	Whole cell	*Mycobacterium tuberculosis* H37Rv	56	437	0.6695	0.3537	0.2224	0.2406	0.161
**Tuberculosis (SS18b)**	Whole cell	*Mycobacterium tuberculosis* SS18b	41	437	0.7484	0.3102	0.1918	0.2546	0.161

Table S2. Assay Central® model statistics. Abbreviations: ROC = receiver operator characteristic, Kappa = Cohen’s Kappa, F1 = F1-Score, MCC = Matthews Correlation Coefficient.

Winning Algorithm	ada	bnb	DL	knn	rf	svc	AC
**ada**	N/A						
**bnb**	18.99 (ns)	N/A					
**DL**	29.98 (ns)	10.99 (ns)	N/A	1.154 (ns)			
**knn**	28.82 (ns)	9.832 (ns)	9 (ns)	N/A			
**rf**	38.98**	19.99 (ns)		10.15 (ns)	N/A		
**svc**	50.21****	31.22*	20.23 (ns)	21.38 (ns)	11.23 (ns)	N/A	
**AC**	50.28****	31.29*	20.31 (ns)	21.46 (ns)	11.31 (ns)	0.077 (ns)	N/A

Table S3. Distance from the top normalized score (Kruskal-Wallis test, Dunn's multiple comparisons test, Mean rank difference). Abbreviations: AC = Assay Central® (Bayesian), rf = random forest, knn = k-Nearest Neighbors, svc = support vector classification, bnb = naïve Bayesian, ada = AdaBoosted decision trees, DL = deep learning architecture.

Algorithm	ada	bnb	DL	knn	rf	svc	AC
**ada**	N/A	>0.9999	0.0892	0.1255	0.0042**	<0.0001****	<0.0001****
**bnb**	>0.9999	N/A	>0.9999	>0.9999	>0.9999	0.047*	0.0458*
**DL**	0.0892	>0.9999	N/A	>0.9999	>0.9999	0.8091	0.7937
**knn**	0.1255	>0.9999	>0.9999	N/A	>0.9999	0.6033	0.5913
**rf**	0.0042**	>0.9999	>0.9999	>0.9999	N/A	>0.9999	>0.9999
**svc**	<0.0001****	0.047*	0.8091	0.6033	>0.9999	N/A	>0.9999
**AC**	<0.0001****	0.0458*	0.7937	0.5913	>0.9999	>0.9999	N/A

Table S4. Distance from the top normalized score (Kruskal-Wallis test, Dunn's multiple comparisons test, Adjusted P Value). Abbreviations: AC = Assay Central® (Bayesian), rf = random forest, knn = k-Nearest Neighbors, svc = support vector classification, bnb = naïve Bayesian, ada = AdaBoosted decision trees, DL = deep learning architecture.

	Ada	Bnb	DL	Knn	Rf	Svc	AC
**Ada**	N/A	>0.9999	>0.9999	>0.9999	>0.9999	>0.9999	>0.9999
**Bnb**	>0.9999	N/A	>0.9999	>0.9999	>0.9999	>0.9999	>0.9999
**DL**	>0.9999	>0.9999	N/A	>0.9999	>0.9999	>0.9999	>0.9999
**Knn**	>0.9999	>0.9999	>0.9999	N/A	>0.9999	>0.9999	>0.9999
**Rf**	>0.9999	>0.9999	>0.9999	>0.9999	N/A	>0.9999	>0.9999
**Svc**	>0.9999	>0.9999	>0.9999	>0.9999	>0.9999	N/A	>0.9999
**AC**	>0.9999	>0.9999	>0.9999	>0.9999	>0.9999	>0.9999	N/A

Table S5. Rank normalized score (Kruskal-Wallis test (Independent), Dunn's multiple comparisons test, Adjusted P Value).

Winning Algorithm	ada	bnb	DL	knn	rf	svc	AC
**ada**	N/A						
**bnb**	10.65 (ns)	N/A	0.853 (ns)	2.315 (ns)			
**DL**	9.792 (ns)		N/A	1.462 (ns)			
**knn**	8.331 (ns)			N/A			
**rf**	13.48 (ns)	2.839 (ns)	3.692 (ns)	5.154 (ns)	N/A		1.462 (ns)
**svc**	18.79 (ns)	8.147 (ns)	9 (ns)	10.46 (ns)	5.308 (ns)	N/A	
**AC**	17.33 (ns)	6.685 (ns)	7.538 (ns)	9 (ns)	3.846 (ns)		N/A

Table S6. Rank normalized score (Kruskal-Wallis test (Independent), Dunn's multiple comparisons test, Mean rank difference). Abbreviations: AC = Assay Central® (Bayesian), rf = random forest, knn = k-Nearest Neighbors, svc = support vector classification, bnb = naïve Bayesian, ada = AdaBoosted decision trees, DL = deep learning architecture.

	ada	bnb	DL	knn	rf	svc	AC
**ada**	N/A	0.2726	0.0194*	0.0788	0.0011**	<0.0001****	0.0003***
**bnb**	0.2726	N/A	>0.9999	>0.9999	>0.9999	0.6243	>0.9999
**DL**	0.0194*	>0.9999	N/A	>0.9999	>0.9999	>0.9999	>0.9999
**knn**	0.0788	>0.9999	>0.9999	N/A	>0.9999	>0.9999	>0.9999
**rf**	0.0011**	>0.9999	>0.9999	>0.9999	N/A	>0.9999	>0.9999
**svc**	<0.0001****	0.6243	>0.9999	>0.9999	>0.9999	N/A	>0.9999
**AC**	0.0003***	>0.9999	>0.9999	>0.9999	>0.9999	>0.9999	N/A

Table S7. Rank normalized score (Friedman test (Pairwise comparison), Dunn's multiple comparison test, Adjusted P Value). Abbreviations: AC = Assay Central® (Bayesian), rf = random forest, knn = k-Nearest Neighbors, svc = support vector classification, bnb = naïve Bayesian, ada = AdaBoosted decision trees, DL = deep learning architecture.

Winning Algorithm	ada	bnb	DL	knn	rf	svc	AC
**ada**	N/A						
**bnb**	24 (ns)	N/A					
**DL**	32*	8 (ns)	N/A	4 (ns)			
**knn**	28 (ns)	4 (ns)		N/A			
**rf**	39**	15 (ns)	7 (ns)	11 (ns)	N/A		
**svc**	45****	21 (ns)	13 (ns)	17 (ns)	6 (ns)	N/A	3 (ns)
**AC**	42***	18 (ns)	10 (ns)	14 (ns)	3 (ns)		N/A

Table S8. Rank normalized score (Friedman test (Pairwise comparison), Dunn's multiple comparison test, mean rank difference). Abbreviations: AC = Assay Central® (Bayesian), rf = random forest, knn = k-Nearest Neighbors, svc = support vector classification, bnb = naïve Bayesian, ada = AdaBoosted decision trees, DL = deep learning architecture.

Secondary Hits	*T. brucei*			*L. infantum*			*T. cruzi*		
	Predicted as active	% inhbition at 50 µM	EC_{50} (µM)	Predicted as active	% inhbition at 50 µM	EC_{50} (µM)	Predicted as active	% inhbition at 50 µM	EC_{50} (µM)
**1**	NO	16.4	N/D	NO	34	N/D	YES	0	N/D
**2**	NO	44.81	N/D	NO	0	N/D	YES	12.1	N/D
**3**	YES	19.61	N/D	NO	6.86	N/D	YES	47.9	N/D
**4**	YES	0	N/D	NO	6.69	N/D	YES	56.7	N/D
**5**	YES	0	N/D	NO	16.99	N/D	YES	74.2	N/D
**6**	NO	12.56	N/D	NO	23.99	N/D	NO	1.37	N/D
**7**	NO	7.19	N/D	NO	0	N/D	YES	48.27	N/D
**8**	NO	98.96	3.0	NO	46.21	N/D	NO	57.46	N/D
**9**	YES	85.66	>50	YES	-4.66	N/D	NO	13.3	N/D
**10**	YES	97.4	>50	YES	9.61	N/D	NO	-7	N/D
**11**	YES	96.76	>50	YES	30.73	N/D	NO	0	N/D
**12**	YES	0	N/D	YES	23.38	N/D	NO	0	N/D
**13**	NO	29.83	N/D	NO	34.18	N/D	NO	13	N/D
**14**	NO	0	N/D	YES	22.41	N/D	NO	-4.2	N/D
**15**	YES	99.21	N.I.	YES	27.83	N/D	NO	8.58	N/D
**16**	YES	102.13	5.2	YES	24.23	N/D	NO	25.52	N/D
**17**	YES	102.97	2.4	YES	-15	N/D	NO	7.14	N/D
**18**	YES	94.41	7.12	YES	0	N/D	NO	0	N/D
**19**	YES	56.39	N/D	YES	53.98	N/D	NO	-6	N/D
**20**	NO	26.96	N/D	YES	3.06	N/D	NO	22.01	N/D
**21**	YES	54.24	N/D	YES	0	N/D	NO	25.98	N/D
**22**	YES	85.71	>50	YES	37.89	N/D	NO	12.19	N/D
**23**	YES	52.98	N/D	YES	-16	N/D	NO	14.05	N/D
**24**	NO	46.30	N/D	NO	25.54	N/D	NO	14.21	N/D
**25**	NO	39.32	N/D	YES	19.16	N/D	NO	13.17	N/D
**26**	YES	69.85	N/D	YES	47.62	N/D	NO	12.49	N/D
**27**	YES	0	N/D	YES	54.13	N/D	NO	-3.28	N/D

**28**	YES	39.24	N/D	YES	0	N/D	NO	20.89	N/D
**29**	YES	87.56	>50	YES	38.32	N/D	NO	21.85	N/D
**30**	YES	83.63	N.I.	YES	21.61	N/D	NO	17.88	N/D
**31**	YES	95.09	N.I.	YES	43.01	N/D	NO	3.76	N/D
**32**	YES	28.88	N/D	YES	-4.78	N/D	NO	20	N/D
**33**	YES	99.12	>50	YES	1	N/D	NO	10.27	N/D
**34**	YES	94.57	>50	NO	15.394	N/D	NO	1.38	N/D
**35**	YES	98.24	>50	YES	72.68	N/D	NO	-2	N/D
**36**	YES	30.48	N/D	YES	-10	N/D	NO	17.2	N/D
**37**	YES	100	>50	NO	11.908	N/D	NO	1.73	N/D
**38**	YES	98.42	5.5	YES	90.04	>50	NO	10.45	N/D
**39**	YES	96.24	6.8	YES	75.36	N/D	NO	17.44	N/D
**40**	YES	95.36	3.0	YES	95.758	6.0	YES	43.32	N/D
**41**	YES	93.19	12.7	YES	56.209	N/D	NO	-7.28	N/D
**42**	YES	90.82	7.1	YES	63.029	N/D	NO	38.19	N/D
**43**	YES	99.3	4.1	YES	2.7	N/D	NO	45.2	N/D
**44**	YES	90.6	5.3	YES	-7.23	N/D	NO	0	N/D
**45**	NO	43.86	N/D	YES	21.51	N/D	NO	7.8	N/D
**46**	YES	6.77	N/D	YES	34	N/D	NO	-2.2	N/D
**47**	YES	52.74	N/D	NO	22.01	N/D	NO	-2	N/D

SD for all the assays are within ± 10% of each value.
N/D. Not Determined

Table S9. Predicted antiparasitic activity according to the elaborated Bayesian model and experimental antiparasitic activity (% inhibition at 50 μM and EC_{50} in μM) against *T. brucei*, *L. infantum* and *T. cruzi* of the synthesized test hit compounds.

Secondary Hits	CYP1A2		CYP2C19		CYP2C9		CYP2D6		CYP3A4	
	**Pred. as active**	**% Inh at 10µM**	**Pred. as active**	**% Inh at 10µM**	**Pred. as active**	**% Inh at 10µM**	**Pred. as active**	**% Inh at 10µM**	**Pred. as active**	**% Inh at 10µM**
**1**	NO	30.77	NO	33.93	NO	23.40	NO	10.83	NO	-2.99
**2**	YES	65.65	YES	32.27	YES	20.25	NO	17.76	NO	-5.28
**3**	YES	59.17	YES	74.44	NO	5.76	NO	-16.55	NO	-3.40
**4**	YES	29.10	YES	30.57	NO	-4.45	NO	-7.65	NO	-7.15
**5**	YES	76.97	YES	93.99	NO	55.69	NO	-10.08	NO	20.67
**6**	YES	26.75	NO	22.73	NO	28.87	NO	11.80	NO	-7.23
**7**	YES	77.23	NO	28.18	NO	12.13	NO	-14.44	NO	-12.52
**8**	NO	27.51	NO	34.44	NO	18.90	NO	21.83	NO	1.01
**9**	NO	18.62	NO	106.41	YES	65.53	NO	14.04	NO	4.91
**10**	NO	11.68	NO	5.72	NO	14.23	NO	-4.25	NO	-20.28
**11**	NO	26.13	NO	25.91	NO	27.91	NO	8.31	NO	-0.08
**12**	NO	23.93	NO	20.44	NO	27.45	NO	20.04	NO	8.08
**13**	NO	18.41	NO	-1.37	NO	-27.03	NO	-11.03	NO	-0.32
**14**	NO	25.18	NO	6.78	NO	-3.77	YES	-16.45	NO	0.07
**15**	NO	22.93	NO	28.03	NO	28.82	NO	14.91	NO	15.31
**16**	NO	49.17	NO	51.50	YES	57.08	NO	75.53	YES	59.55
**17**	NO	23.03	YES	7.91	YES	19.79	YES	20.43	YES	-4.82
**18**	NO	8.85	NO	1.20	YES	46.32	YES	7.07	YES	-15.18
**19**	NO	28.62	NO	21.67	YES	32.45	YES	14.48	NO	31.06
**20**	NO	22.95	NO	8.13	NO	7.66	YES	4.26	NO	-3.82
**21**	NO	21.06	NO	17.77	YES	11.28	YES	12.57	YES	-9.08
**22**	NO	31.32	NO	43.68	YES	27.34	YES	21.11	YES	15.01
**23**	NO	25.42	NO	15.33	YES	59.75	YES	20.21	YES	30.00
**24**	NO	28.65	NO	69.53	NO	-52.39	YES	18.94	NO	57.13
**25**	NO	28.33	NO	31.97	NO	21.56	YES	0.24	NO	54.62
**26**	NO	17.35	NO	27.26	YES	19.36	YES	73.56	YES	57.69

Secondary Hits	CYP1A2		CYP2C19		CYP2C9		CYP2D6		CYP3A4	
	Pred. as active	% Inh at 10µM	Pred. as active	% Inh at 10µM	Pred. as active	% Inh at 10µM	Pred. as active	% Inh at 10µM	Pred. as active	% Inh at 10µM
**27**	NO	31.23	NO	17.32	YES	23.74	YES	43.25	YES	34.03
**28**	NO	14.84	NO	7.94	YES	45.50	YES	10.71	YES	63.75
**29**	NO	35.84	NO	50.17	NO	61.07	YES	25.75	NO	73.58
**30**	NO	25.73	NO	57.73	YES	35.48	YES	83.66	YES	30.85
**31**	NO	33.05	NO	30.36	YES	38.06	YES	59.35	YES	31.58
**32**	NO	21.47	NO	12.80	NO	5.13	YES	-4.73	NO	-12.71
**33**	NO	21.20	NO	23.77	YES	22.79	YES	27.60	YES	21.01
**34**	NO	30.53	YES	61.20	YES	62.31	YES	83.85	YES	96.79
**35**	NO	31.52	NO	22.00	YES	36.42	YES	16.17	YES	50.22
**36**	NO	22.34	NO	49.92	YES	34.66	YES	31.01	YES	29.87
**37**	NO	19.93	NO	12.48	YES	39.93	YES	19.71	YES	35.49
**38**	NO	38.49	YES	48.56	YES	69.83	YES	27.23	YES	80.33
**39**	NO	43.07	NO	17.44	YES	31.22	YES	40.70	YES	5.28
**40**	NO	5.00	YES	37.00	YES	48.00	YES	32.00	YES	42.00
**41**	NO	42.10	NO	2.51	YES	17.25	YES	27.55	YES	21.10
**42**	NO	39.26	YES	36.33	YES	33.50	YES	41.28	YES	13.05
**43**	NO	36.42	YES	74.41	YES	58.59	YES	70.07	YES	97.65
**44**	NO	33.59	YES	59.44	YES	78.70	YES	66.81	YES	63.14
**45**	NO	43.50	NO	7.66	NO	6.02	NO	-15.30	NO	-11.88
**46**	NO	29.87	NO	30.84	NO	28.95	NO	11.23	NO	-16.24
**47**	NO	25.29	NO	32.51	NO	16.71	NO	8.64	NO	0.34

SD for all the assays are within ± 10% of each value.

Table S10. Prediction by the elaborated Bayesian model vs. experimental CYPs inhibition of the secondary hits.

Secondary Hits	Mitochondria		*h*ERG	
	Pred. as toxic	% Tox. at 10µM	Pred. as toxic	% Inh at 10µM
**1**	NO	23.59	NO	10.26
**2**	NO	11.6	NO	-23.22
**3**	NO	1.13	NO	1.68
**4**	NO	-6.02	NO	0.43
**5**	NO	-0.4	NO	-3.70
**6**	NO	15.84	NO	-29.41
**7**	NO	11.8	NO	-25.10
**8**	NO	12.22	NO	-14.75
**9**	YES	3.61	YES	-10.10
**10**	YES	11.79	YES	12.61
**11**	NO	17.73	NO	-5.66
**12**	NO	23.76	NO	-3.15
**13**	YES	29.31	NO	-13.97
**14**	NO	25.39	NO	-3.83
**15**	NO	13.12	YES	15.41
**16**	YES	23.31	YES	89.27
**17**	YES	10	YES	53.81
**18**	YES	13.29	YES	130.30
**19**	NO	35.8	YES	-1.94
**20**	NO	10.22	YES	-39.55
**21**	NO	10.45	YES	-26.14
**22**	NO	33.58	YES	-0.20
**23**	NO	14.86	YES	-4.86
**24**	NO	20.81	NO	-25.49
**25**	NO	19.7	YES	-39.98
**26**	NO	12.91	YES	-10.86
**27**	NO	27.2	YES	-14.53
**28**	NO	10.53	YES	11.36
**29**	NO	44.24	YES	-4.26
**30**	NO	22.73	YES	16.79
**31**	NO	43.22	YES	61.92
**32**	NO	10.47	YES	-10.56
**33**	NO	26.92	YES	-4.63
**34**	NO	50.7	YES	6.08
**35**	NO	37.53	YES	76.24
**36**	NO	2.20	YES	-45.23
**37**	NO	25.37	NO	6.69
**38**	NO	46.74	NO	3.86

Secondary Hits	Mitochondria		*h*ERG	
	Pred. as toxic	% Tox. at 10µM	Pred. as toxic	% Inh at 10µM
**39**	NO	14.81	YES	46.35
**40**	NO	2.01	NO	8.00
**41**	NO	2.04	NO	42.66
**42**	NO	46.43	YES	35.46
**43**	NO	35.03	YES	60.30
**44**	NO	38.23	YES	-13.56
**45**	YES	24.95	NO	-11.69
**46**	YES	9.99	YES	-12.10
**47**	NO	3.95	NO	-0.27

SD for all the assays are within ± 10% of each value.

Table S11. Prediction by the elaborated Bayesian model vs. experimental mitochondria and *h*ERG toxicity of the secondary hits.

References

1. C. B. Moraes, G. Witt, M. Kuzikov, B. Ellinger, T. Calogeropoulou, K. C. Prousis, S. Mangani, F. Di Pisa, G. Landi, L. Dello Iacono, C. Pozzi, L. H. Freitas-Junior, B. dos Santos Pascoalino, C. P. Bertolacini, B. Behrens, O. Keminer, J. Leu, M. Wolf, J. Reinshagen, A. Cordeiro-da-Silva, N. Santarem, A. Venturelli, S. Wrigley, D. Karunakaran, B. Kebede, I. Pöhner, W. Müller, J. Panecka-Hofman, R. C. Wade, M. Fenske, J. Clos, J. M. Alunda, M. J. Corral, E. Uliassi, M. L. Bolognesi, P. Linciano, A. Quotadamo, S. Ferrari, M. Santucci, C. Borsari, M. P. Costi, S. Gul, Accelerating Drug Discovery Efforts for Trypanosomatidic Infections Using an Integrated Transnational Academic Drug Discovery Platform. *SLAS Discov. Adv. Life Sci. R&D*. **24**, 346–361 (2019).
2. Alcântara, L. M. *et al.* A Multi-Species Phenotypic Screening Assay for Leishmaniasis Drug Discovery Shows That Active Compounds Display a High Degree of Species-Specificity. *Molecules* **25**, 2551 (2020).
3. Moraes, C. B. *et al.* Nitroheterocyclic compounds are more efficacious than CYP51 inhibitors against Trypanosoma cruzi: implications for Chagas disease drug discovery and development. *Sci Rep-uk* **4**, 4703 (2014).
4. J. Neres, R. C. Hartkoorn, L. R. Chiarelli, R. Gadupudi, M. R. Pasca, G. Mori, A. Venturelli, S. Savina, V. Makarov, G. S. Kolly, E. Molteni, C. Binda, N. Dhar, S. Ferrari, P. Brodin, V. Delorme, V. Landry, A. L. de Jesus Lopes Ribeiro, D. Farina, P. Saxena, F. Pojer, A. Carta, R. Luciani, A. Porta, G. Zanoni, E. De Rossi, M. P. Costi, G. Riccardi, S. T. Cole, 2-Carboxyquinoxalines Kill Mycobacterium tuberculosis through Noncovalent Inhibition of DprE1. *ACS Chem. Biol.* **10**, 705–714 (2015).
5. E. L. Willighagen, J. W. Mayfield, J. Alvarsson, A. Berg, L. Carlsson, N. Jeliazkova, S. Kuhn, T. Pluskal, M. Rojas-Chertó, O. Spjuth, G. Torrance, C. T. Evelo, R. Guha, C. Steinbeck, The Chemistry Development Kit (CDK) v2.0: atom typing, depiction, molecular formulas, and substructure searching. *J. Cheminform.* **9**, 33 (2017).
6. S. Ekins, A. C. Puhl, K. M. Zorn, T. R. Lane, D. P. Russo, J. J. Klein, A. J. Hickey, A. M. Clark, Exploiting machine learning for end-to-end drug discovery and development. *Nat. Mater.* **18**, 435–441 (2019).
7. H. W. Hernandez, M. Soeung, K. M. Zorn, N. Ashoura, M. Mottin, C. H. Andrade, C. R. Caffrey, J. L. de Siqueira-Neto, S. Ekins, High Throughput and Computational Repurposing for Neglected Diseases. *Pharm. Res.* **36**, 27 (2019).
8. T. Lane, D. P. Russo, K. M. Zorn, A. M. Clark, A. Korotcov, V. Tkachenko, R. C. Reynolds, A. L. Perryman, J. S. Freundlich, S. Ekins, Comparing and Validating Machine Learning Models for Mycobacterium tuberculosis Drug Discovery. *Mol. Pharm.* **15**, 4346–4360 (2018).
9. M. Anantpadma, T. Lane, K. M. Zorn, M. A. Lingerfelt, A. M. Clark, J. S. Freundlich, R. A. Davey, P. B. Madrid, S. Ekins, Ebola Virus Bayesian Machine Learning Models Enable New in Vitro Leads. *ACS Omega*. **4**, 2353–2361 (2019).
10. A. G. Dalecki, K. M. Zorn, A. M. Clark, S. Ekins, W. T. Narmore, N. Tower, L. Rasmussen, R. Bostwick, O. Kutsch, F. Wolschendorf, High-throughput screening and Bayesian machine learning for copper-dependent inhibitors of Staphylococcus aureus. *Metallomics*. **11**, 696–706 (2019).
11. S. Ekins, J. Gerlach, K. M. Zorn, B. M. Antonio, Z. Lin, A. Gerlach, Repurposing Approved Drugs as Inhibitors of Kv7.1 and Nav1.8 to Treat Pitt Hopkins Syndrome. *Pharm. Res.* **36**, 137 (2019).
12. S. Ekins, M. Mottin, P. R. P. S. Ramos, B. K. P. Sousa, B. J. Neves, D. H. Foil, K. M. Zorn, R. C. Braga, M. Coffee, C. Southan, A. C. Puhl, C. H. Andrade, Déjà vu: Stimulating open drug discovery for SARS-CoV-2. *Drug Discov. Today*. **25**, 928–941 (2020).
13. P. J. Sandoval, K. M. Zorn, A. M. Clark, S. Ekins, S. H. Wright, Assessment of Substrate-

Dependent Ligand Interactions at the Organic Cation Transporter OCT2 Using Six Model Substrates. *Mol. Pharmacol.* **94**, 1057–1068 (2018).

14. P.F. Wang, A. Neiner, T. R. Lane, K. M. Zorn, S. Ekins, E. D. Kharasch, Halogen Substitution Influences Ketamine Metabolism by Cytochrome P450 2B6: In Vitro and Computational Approaches. *Mol. Pharm.* **16**, 898–906 (2019).
15. K. M. Zorn, T. R. Lane, D. P. Russo, A. M. Clark, V. Makarov, S. Ekins, Multiple Machine Learning Comparisons of HIV Cell-based and Reverse Transcriptase Data Sets. *Mol. Pharm.* **16**, 1620–1632 (2019).
16. J. Carletta, Assessing Agreement on Classification Tasks: The Kappa Statistic. *Comput. Linguist.* **22**, 249–254 (1996).
17. J. Cohen, A Coefficient of Agreement for Nominal Scales. *Educ. Psychol. Meas.* **20**, 37–46 (1960).
18. B. W. Matthews, Comparison of the predicted and observed secondary structure of T4 phage lysozyme. *Biochim. Biophys. Acta - Protein Struct.* **405**, 442–451 (1975).
19. A. M. Clark, K. Dole, A. Coulon-Spektor, A. McNutt, G. Grass, J. S. Freundlich, R. C. Reynolds, S. Ekins, Open Source Bayesian Models. 1. Application to ADME/Tox and Drug Discovery Datasets. *J. Chem. Inf. Model.* **55**, 1231–1245 (2015).
20. D. P. Russo, K. M. Zorn, A. M. Clark, H. Zhu, S. Ekins, Comparing Multiple Machine Learning Algorithms and Metrics for Estrogen Receptor Binding Prediction. *Mol. Pharm.* **15**, 4361–4370 (2018).
21. A. Korotcov, V. Tkachenko, D. P. Russo, S. Ekins, Comparison of Deep Learning With Multiple Machine Learning Methods and Metrics Using Diverse Drug Discovery Data Sets. *Mol. Pharm.* **14**, 4462–4475 (2017).

Proton and Carbon NMR spectra of compounds from Ty-01 to Ty-44

Ty-01 in MeOD

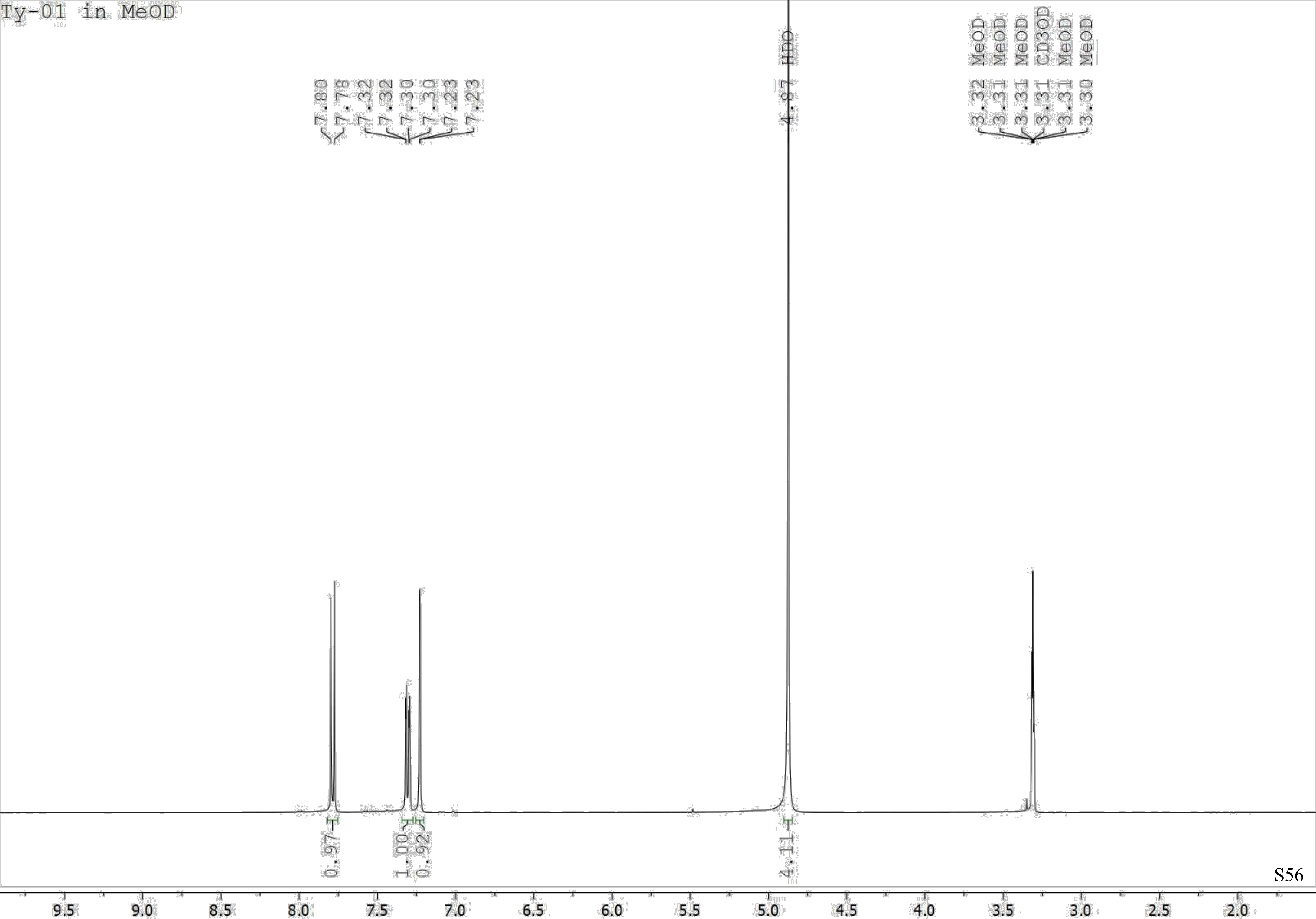
7.80
7.78
7.32
7.32
7.30
7.30
7.23
7.23
4.87 HDO
3.32 MeOD
3.31 MeOD
3.31 MeOD
3.31 CD3OD
3.31 MeOD
3.30 MeOD
0.97
1.00
0.92
4.11
9.5
9.0
8.5
8.0
7.5
7.0
6.5
6.0
5.5
5.0
4.5
4.0
3.5
3.0
2.5
2.0

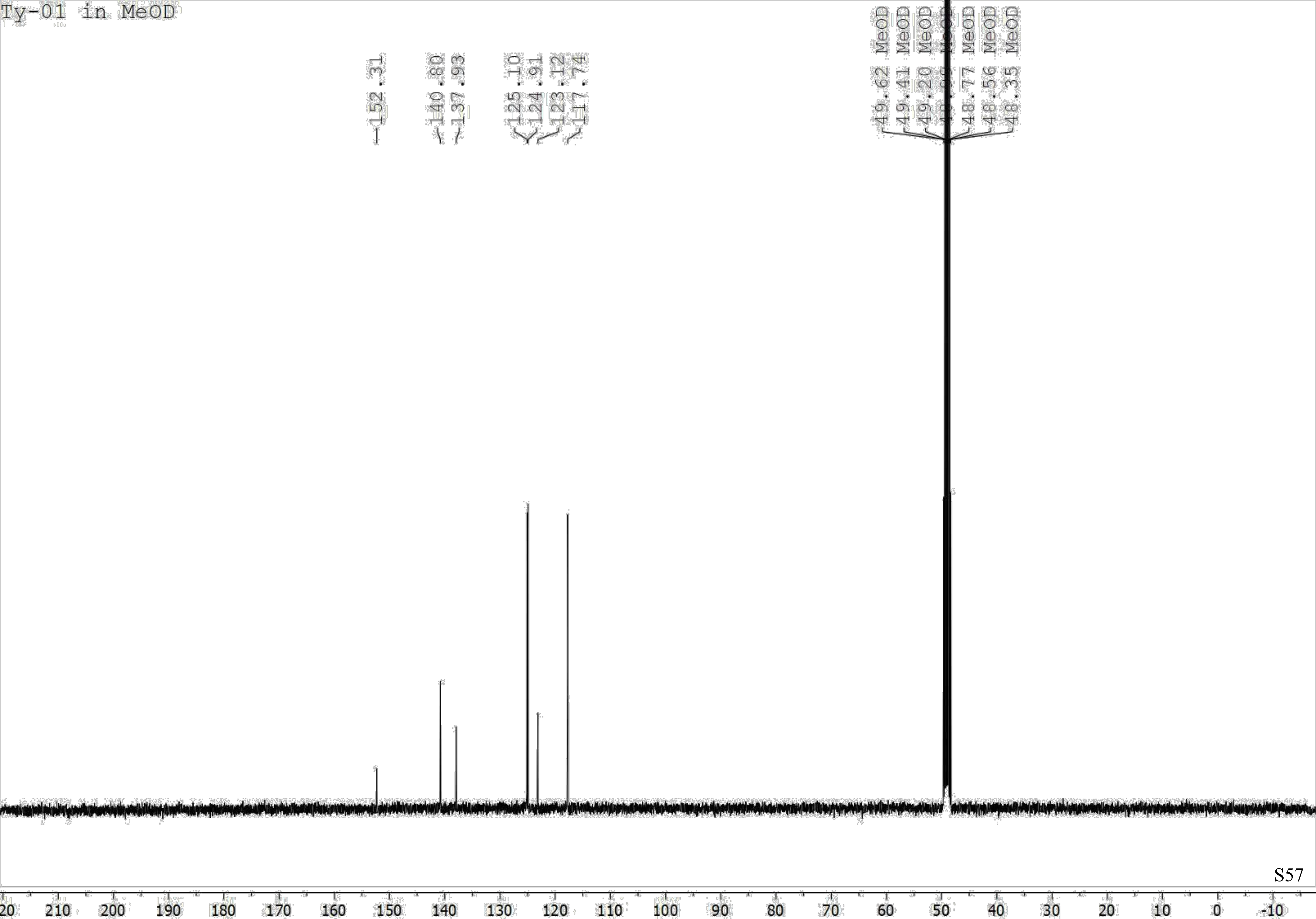
Ty-01 in MeOD
152.31
140.80
137.93
125.10
124.91
123.12
117.74
49.62 MeOD
49.41 MeOD
49.20 MeOD
48.77 MeOD
48.56 MeOD
48.35 MeOD
220 210 200 190 180 170 160 150 140 130 120 110 100 90 80 70 60 50 40 30 20 10 0 -10

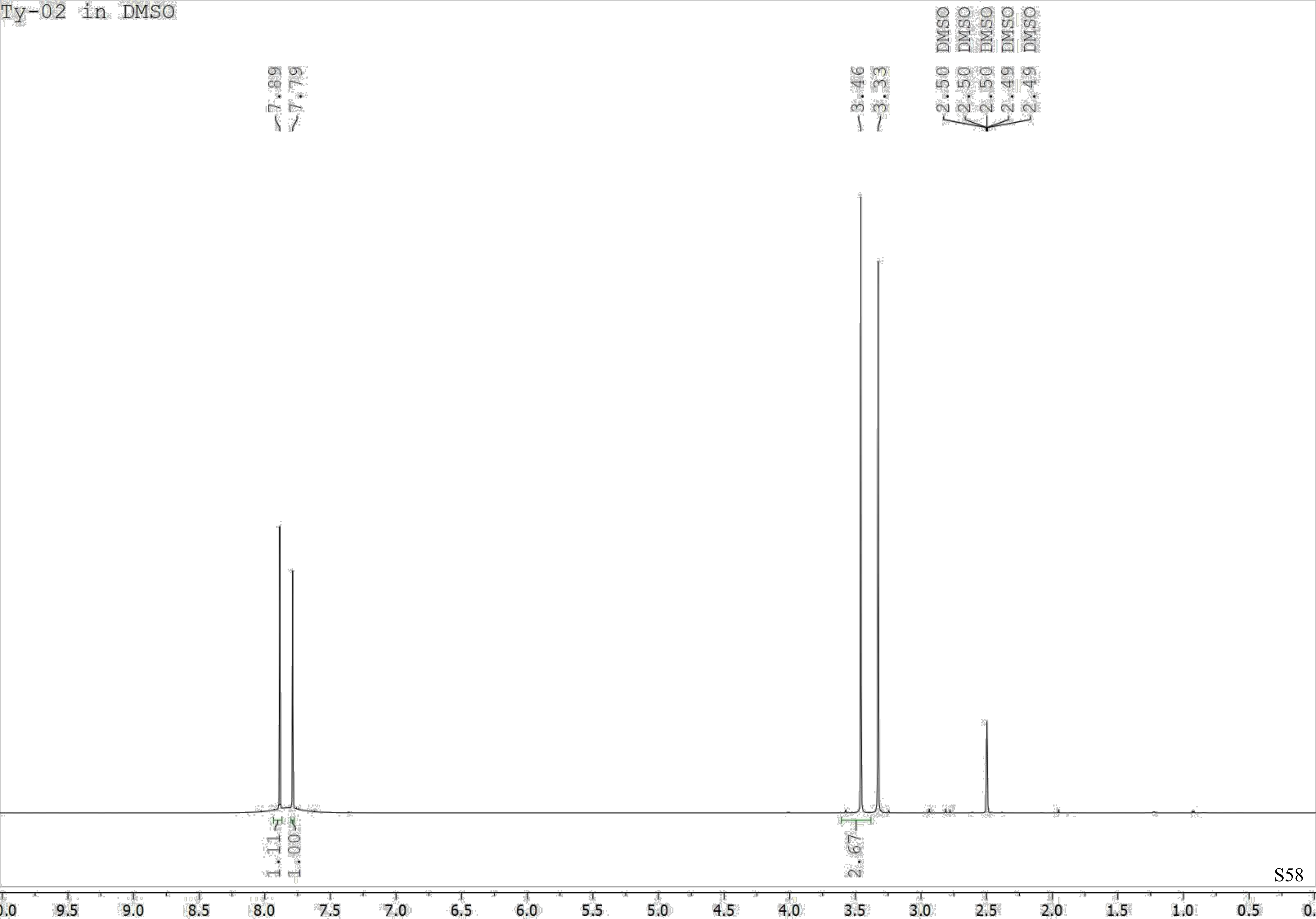

Ty-02 in DMSO
7.89
7.79
3.46
3.33
2.50 DMSO
2.50 DMSO
2.50 DMSO
2.49 DMSO
2.49 DMSO
1.11
1.00
2.67
10.0
9.5
9.0
8.5
8.0
7.5
7.0
6.5
6.0
5.5
5.0
4.5
4.0
3.5
3.0
2.5
2.0
1.5
1.0
0.5
0.0

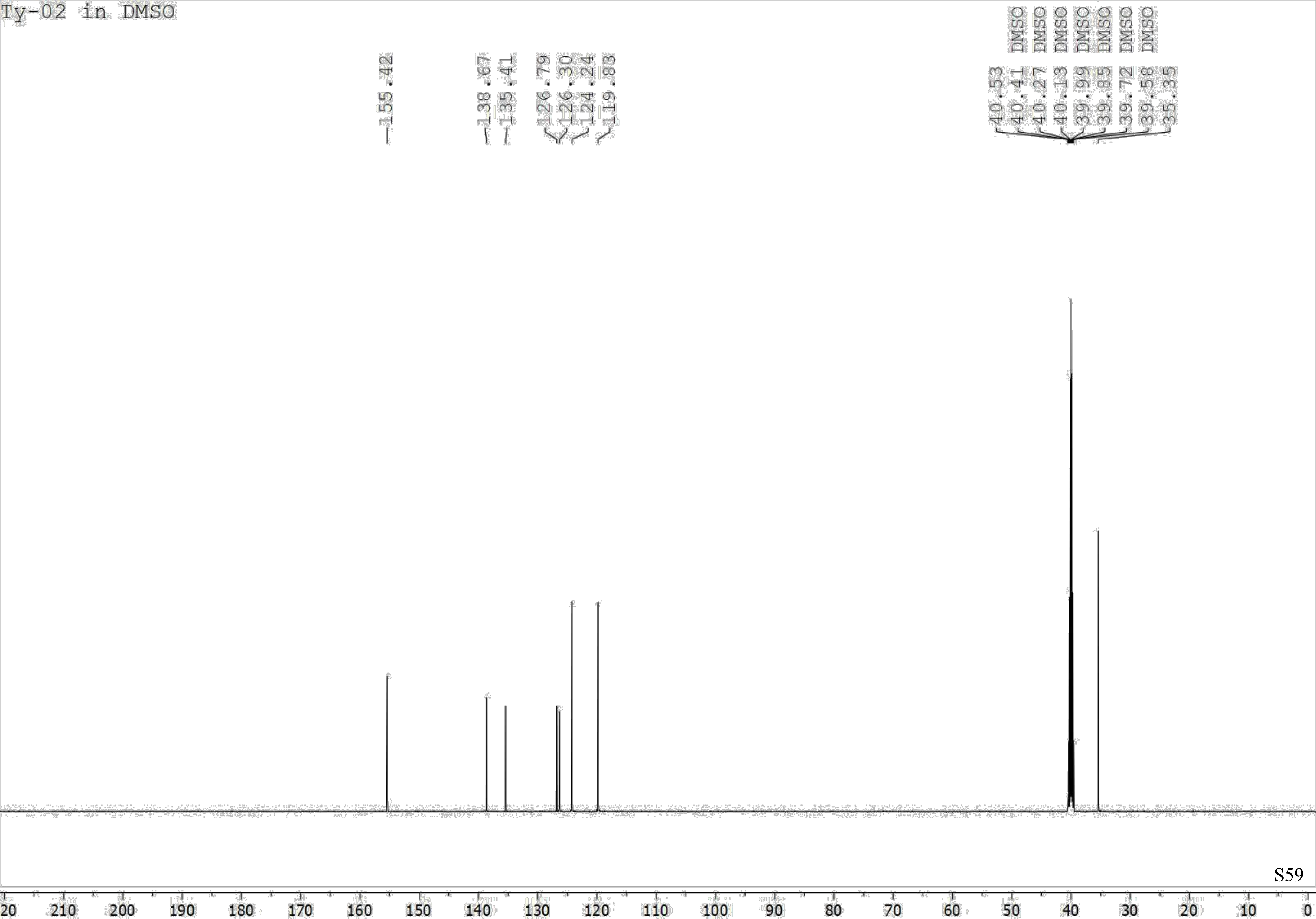
Ty-02 in DMSO
155.42
138.67
135.41
126.79
126.30
124.24
119.83
DMSO
DMSO
DMSO
DMSO
DMSO
DMSO
DMSO
40.53
40.41
40.27
40.13
39.99
39.85
39.72
39.58
35.35
220
210
200
190
180
170
160
150
140
130
120
110
100
90
80
70
60
50
40
30
20
10
0

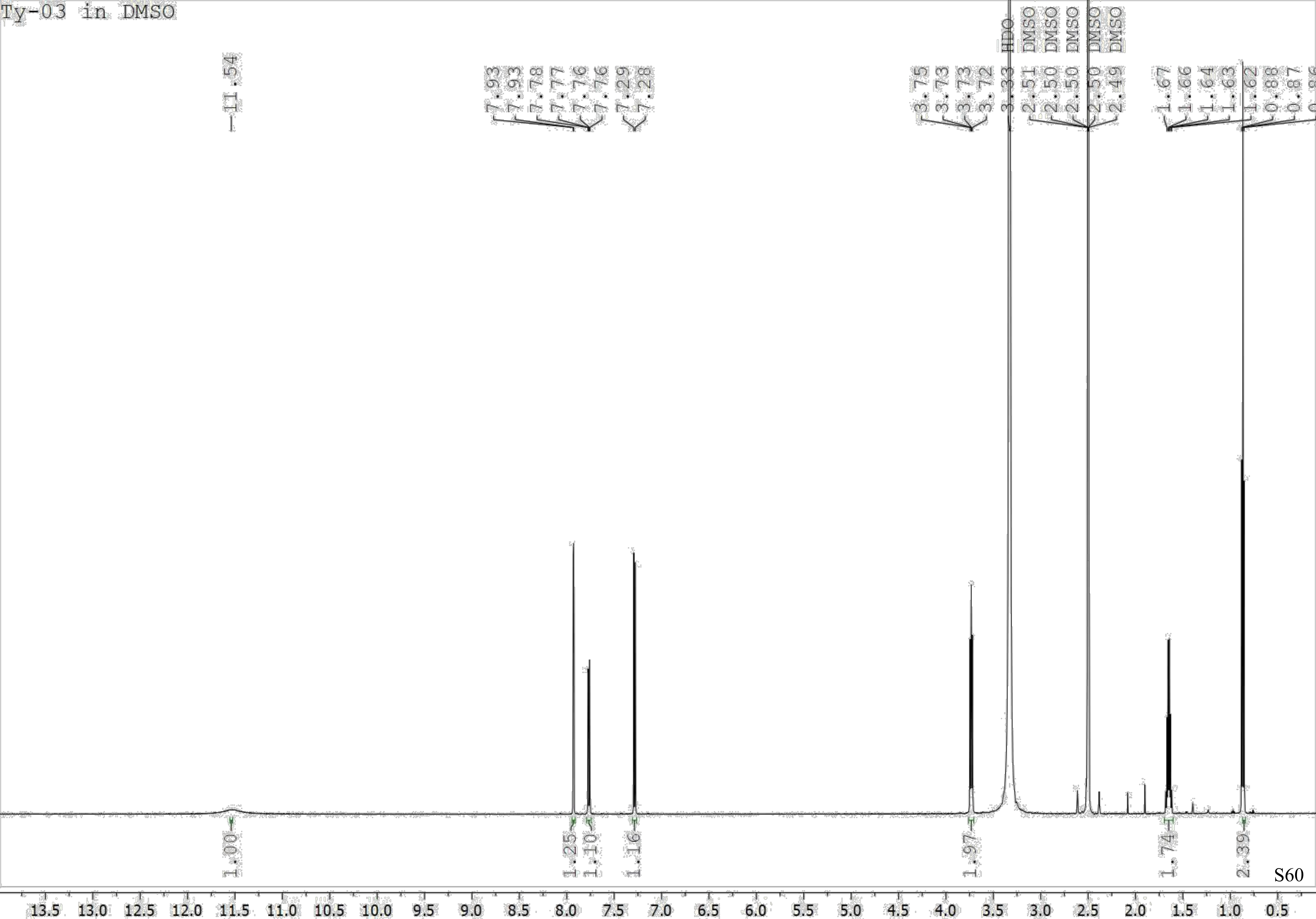
Ty-03 in DMSO
11.54
7.93
7.93
7.78
7.77
7.76
7.76
7.29
7.28
3.75
3.73
3.73
3.72
3.33 HDO
2.51 DMSO
2.50 DMSO
2.50 DMSO
2.50 DMSO
2.49 DMSO
1.67
1.66
1.64
1.63
1.62
0.88
0.87
1.00
1.25
1.10
1.16
1.97
1.74
2.39
13.5 13.0 12.5 12.0 11.5 11.0 10.5 10.0 9.5 9.0 8.5 8.0 7.5 7.0 6.5 6.0 5.5 5.0 4.5 4.0 3.5 3.0 2.5 2.0 1.5 1.0 0.5

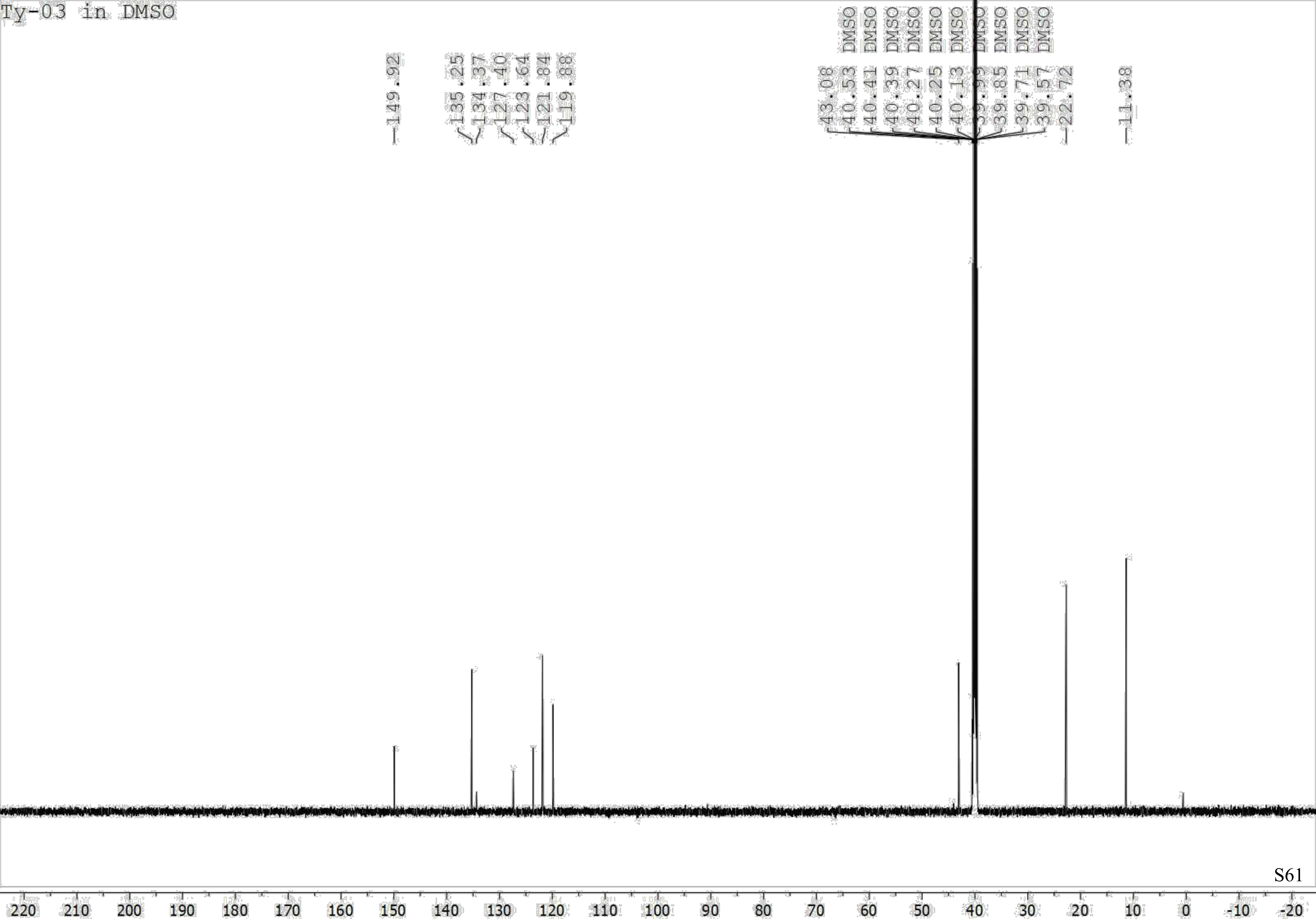
Ty-03 in DMSO
149.92
135.25
134.37
127.40
123.64
121.84
119.88
43.08
40.53
40.41
40.39
40.27
40.25
40.13
39.99
39.85
39.71
39.57
22.72
11.38
DMSO
220 210 200 190 180 170 160 150 140 130 120 110 100 90 80 70 60 50 40 30 20 10 0 -10 -20

Ty-04 in DMSO

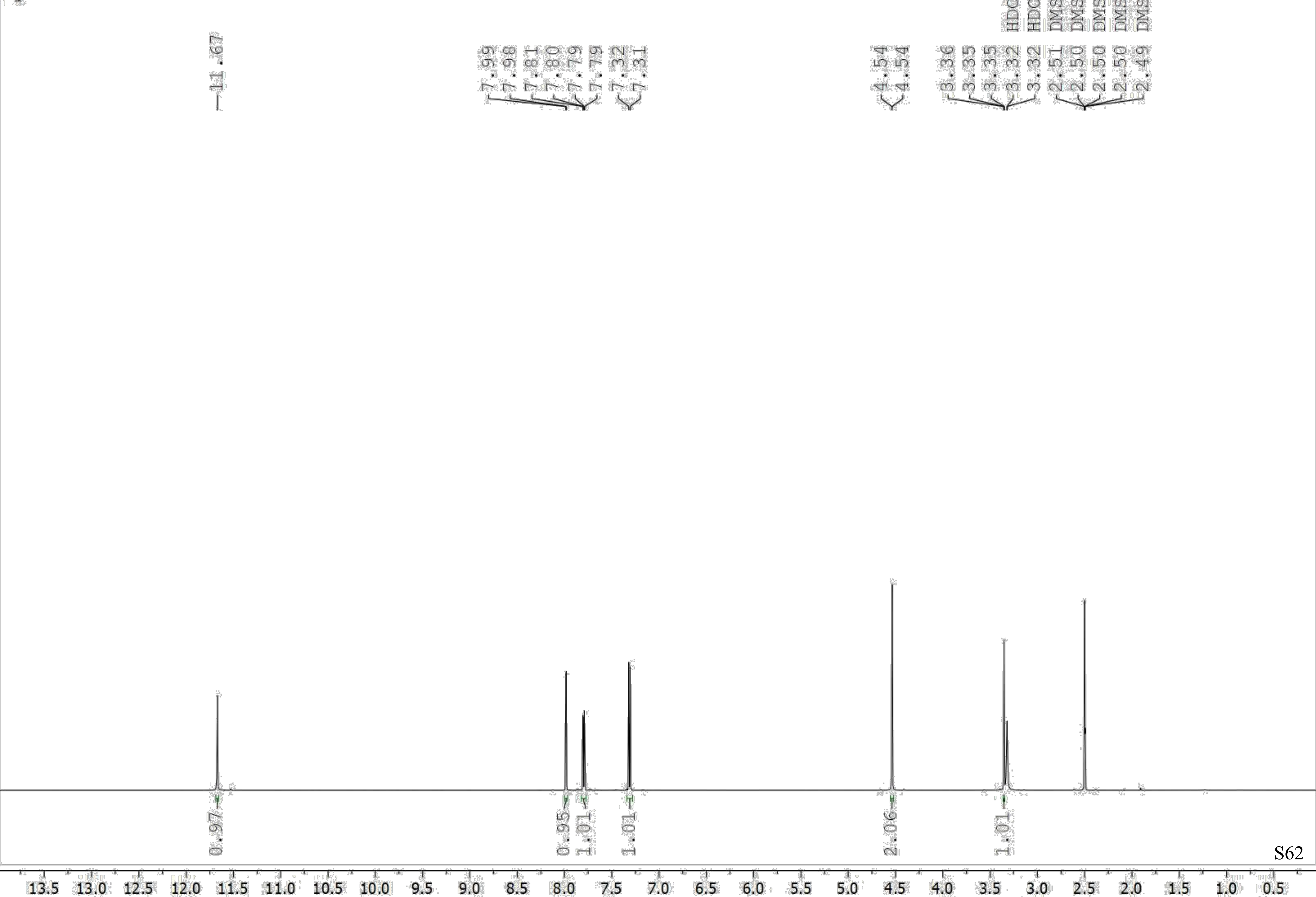
11.67
7.99
7.98
7.81
7.80
7.79
7.79
7.32
7.31
4.54
4.54
3.36
3.35
3.35
3.32
3.32
2.51
2.50
2.50
2.50
2.49
HDO
HDO
DMSO
DMSO
DMSO
DMSO
DMSO
0.97
0.95
1.01
1.01
2.06
1.01
13.5
13.0
12.5
12.0
11.5
11.0
10.5
10.0
9.5
9.0
8.5
8.0
7.5
7.0
6.5
6.0
5.5
5.0
4.5
4.0
3.5
3.0
2.5
2.0
1.5
1.0
0.5

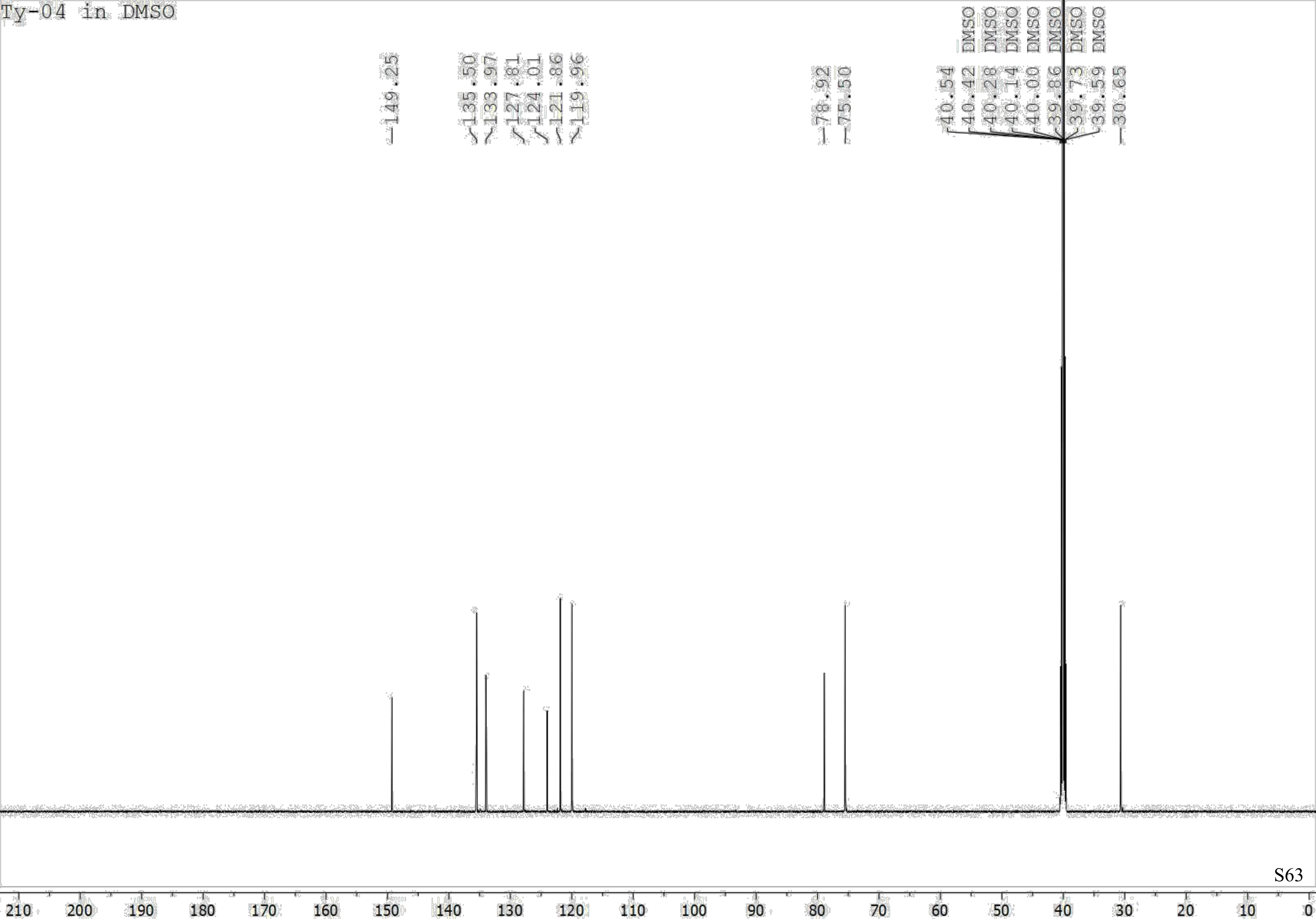
Ty-04 in DMSO
149.25
135.50
133.97
127.81
124.01
121.86
119.96
78.92
75.50
40.54
40.42
40.28
40.14
40.00
39.86
39.73
39.59
30.65
DMSO
DMSO
DMSO
DMSO
DMSO
DMSO
DMSO
210
200
190
180
170
160
150
140
130
120
110
100
90
80
70
60
50
40
30
20
10
0

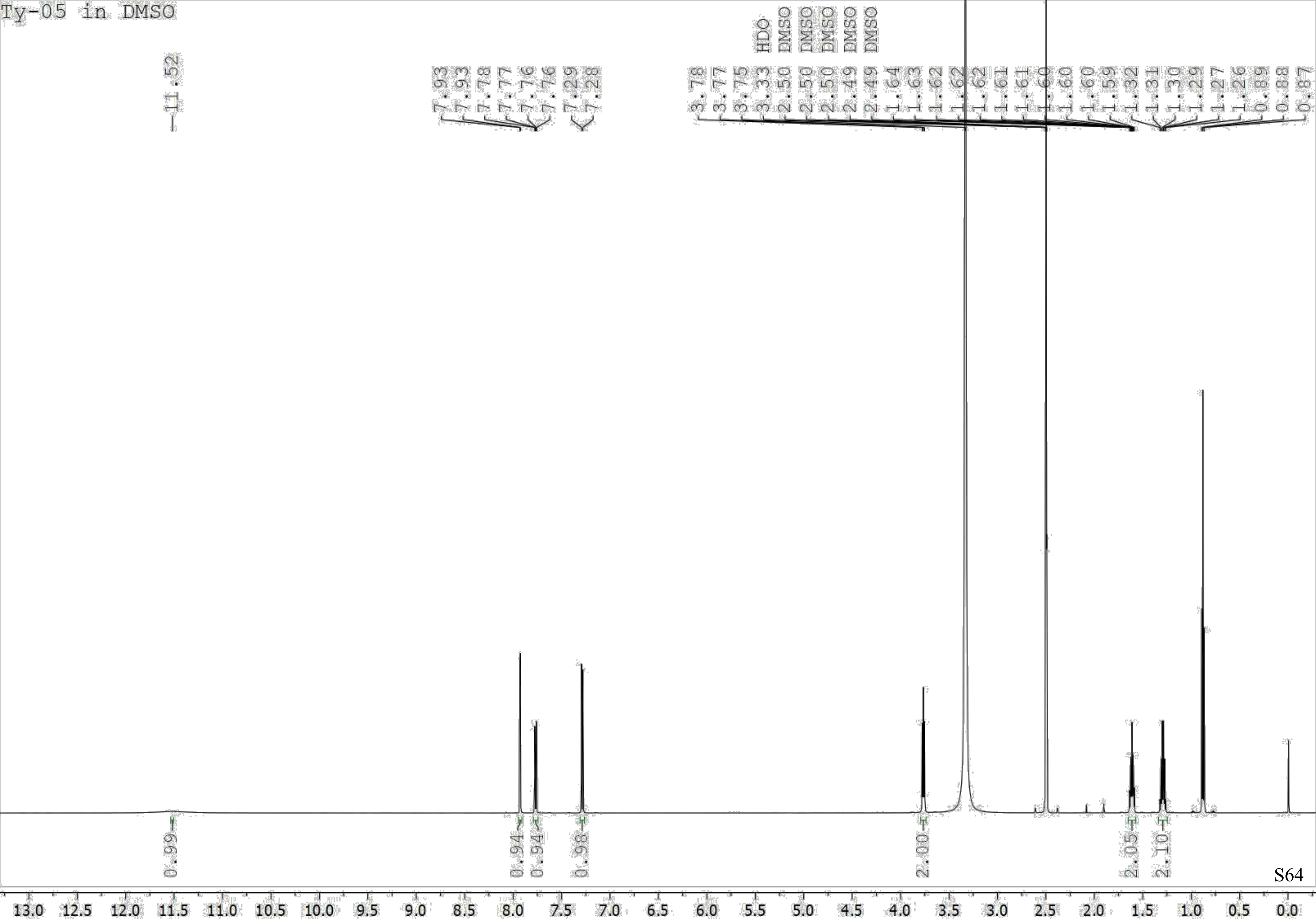
Ty-05 in DMSO
11.52
7.93
7.93
7.78
7.77
7.76
7.76
7.29
7.28
HDO
DMSO
DMSO
DMSO
DMSO
DMSO
3.78
3.77
3.75
3.33
2.50
2.50
2.50
2.49
2.49
1.64
1.63
1.62
1.62
1.62
1.61
1.61
1.60
1.60
1.60
1.59
1.32
1.31
1.30
1.29
1.27
1.26
0.89
0.88
0.87
0.99
0.94
0.94
0.98
2.00
2.05
2.10
13.0
12.5
12.0
11.5
11.0
10.5
10.0
9.5
9.0
8.5
8.0
7.5
7.0
6.5
6.0
5.5
5.0
4.5
4.0
3.5
3.0
2.5
2.0
1.5
1.0
0.5
0.0

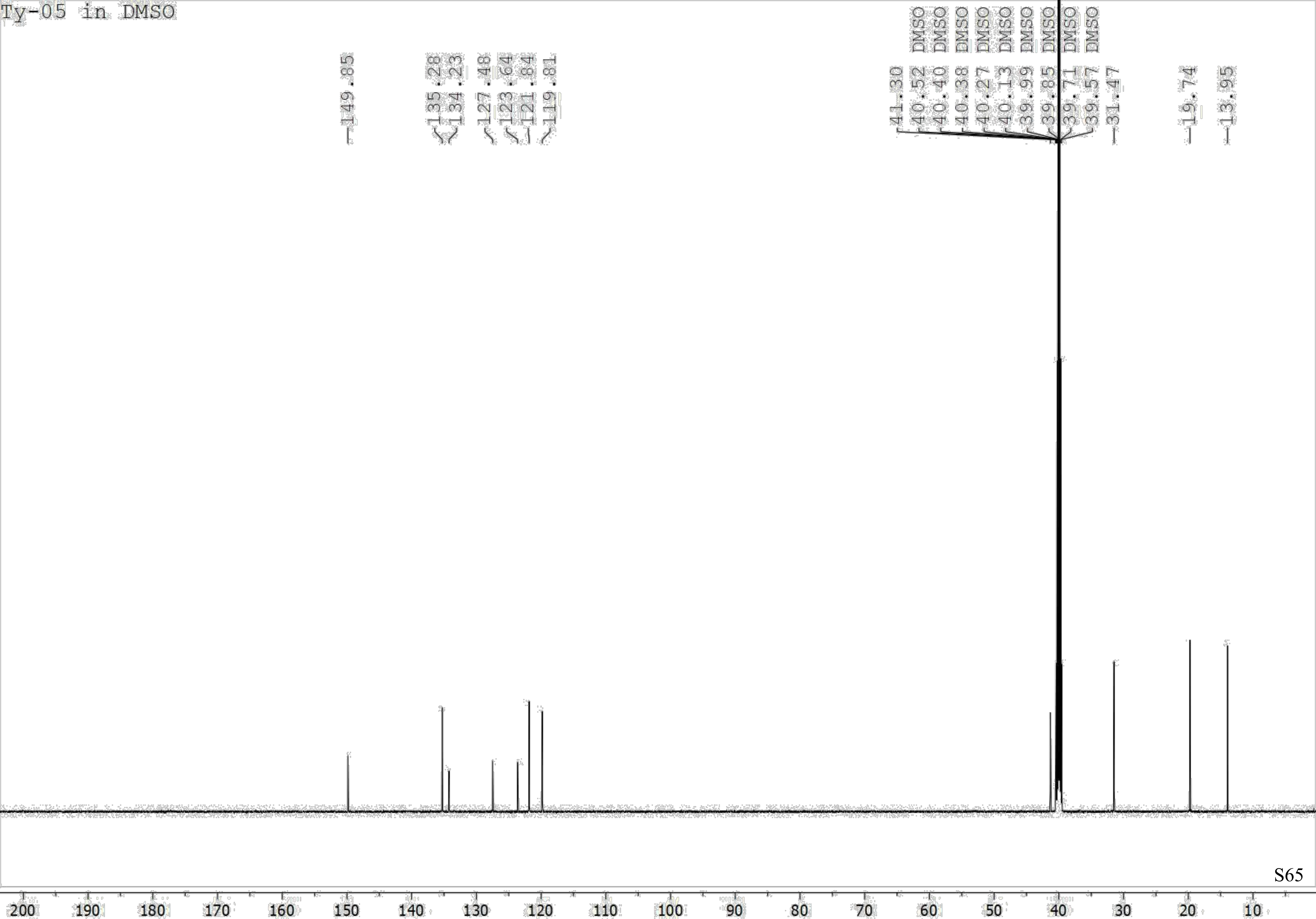
Ty-05 in DMSO
149.85
135.28
134.23
127.48
123.64
121.84
119.81
41.30
40.52
40.40
40.38
40.27
40.13
39.99
39.85
39.71
39.57
31.47
19.74
13.95
DMSO
DMSO
DMSO
DMSO
DMSO
DMSO
DMSO
DMSO
DMSO
200
190
180
170
160
150
140
130
120
110
100
90
80
70
60
50
40
30
20
10

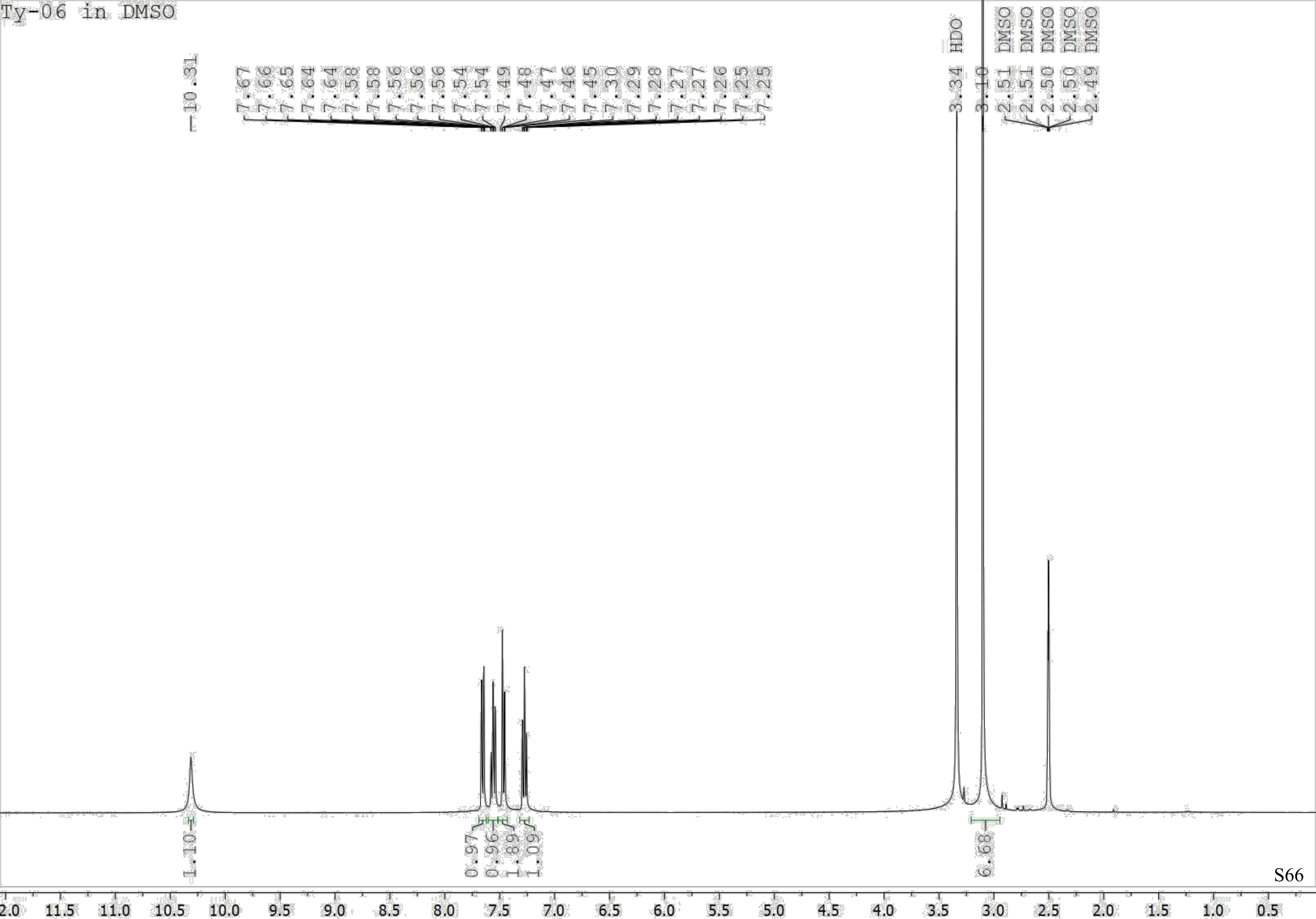
Ty-06 in DMSO
10.31
7.67 7.66 7.65 7.64 7.64 7.58 7.58 7.56 7.56 7.56 7.54 7.54 7.49 7.48 7.47 7.46 7.45 7.30 7.29 7.28 7.27 7.27 7.26 7.25 7.25
3.34 HDO
3.10
2.51 DMSO
2.51 DMSO
2.50 DMSO
2.50 DMSO
2.49 DMSO
1.10
0.97
0.96
1.89
1.09
6.68
2.0 11.5 11.0 10.5 10.0 9.5 9.0 8.5 8.0 7.5 7.0 6.5 6.0 5.5 5.0 4.5 4.0 3.5 3.0 2.5 2.0 1.5 1.0 0.5

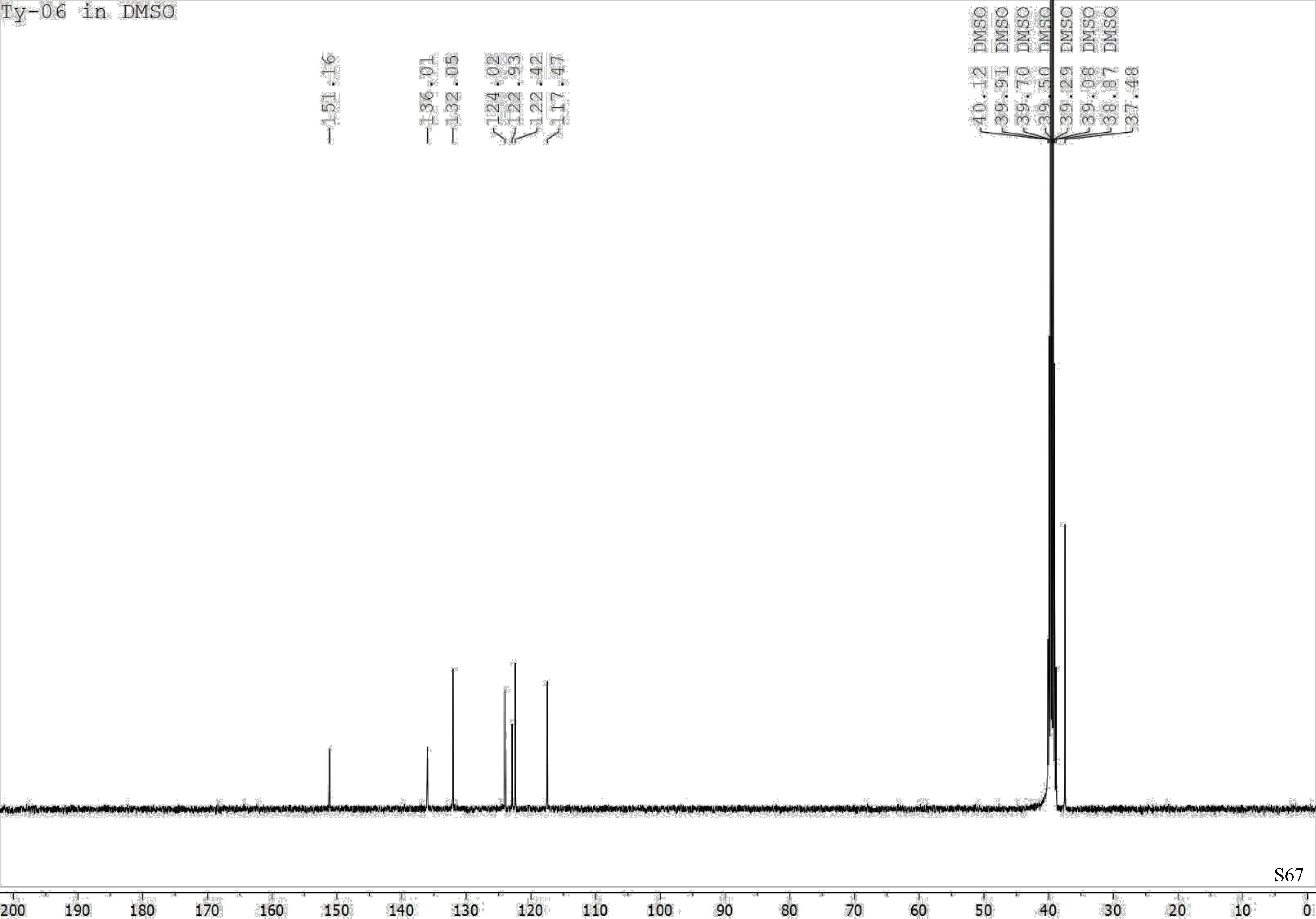
Ty-06 in DMSO
151.16
136.01
132.05
124.02
122.93
122.42
117.47
40.12 DMSO
39.91 DMSO
39.70 DMSO
39.50 DMSO
39.29 DMSO
39.08 DMSO
38.87 DMSO
37.48
200
190
180
170
160
150
140
130
120
110
100
90
80
70
60
50
40
30
20
10
0

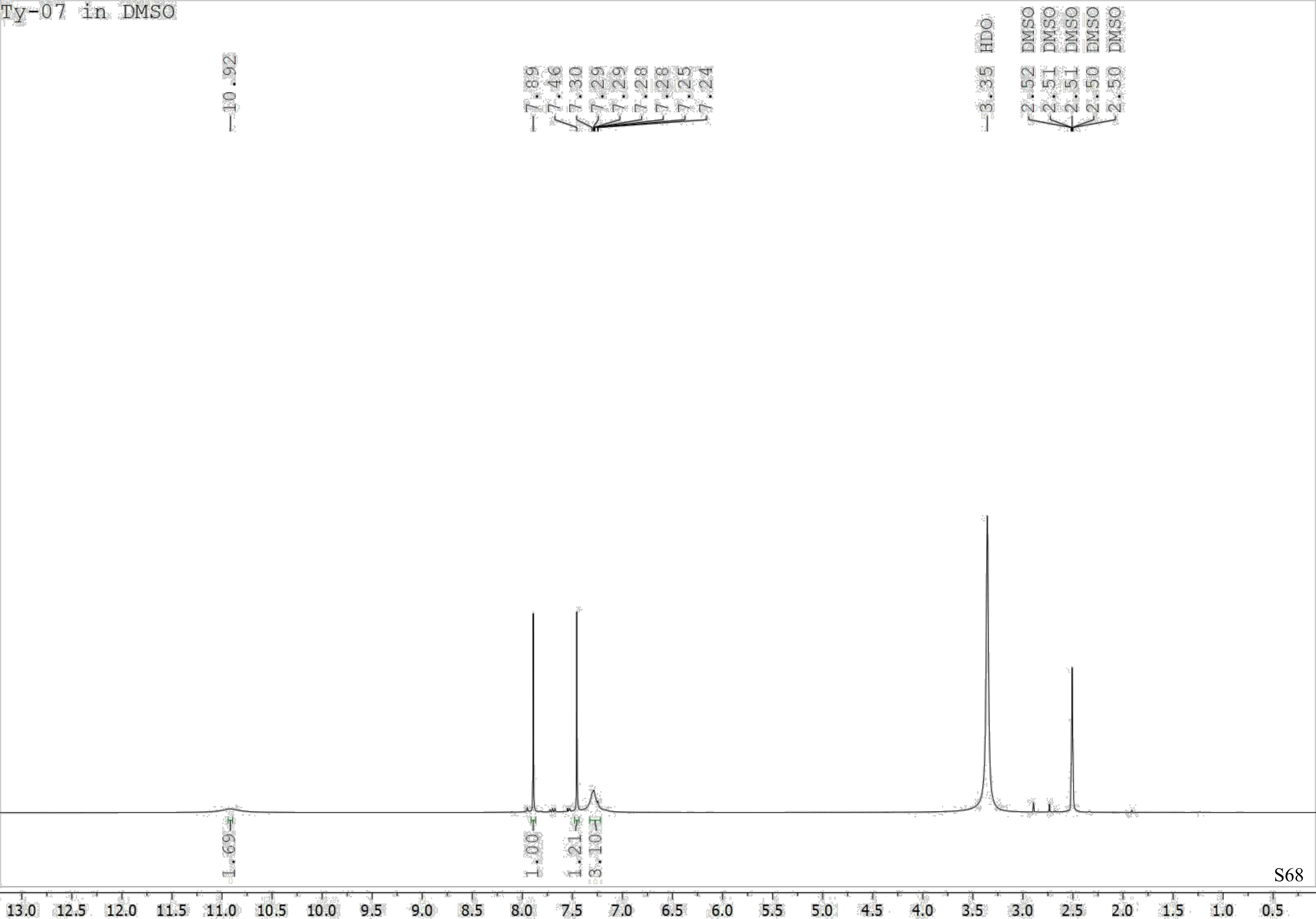
Ty-07 in DMSO
10.92
7.89
7.46
7.30
7.29
7.29
7.28
7.28
7.25
7.24
3.35 HDO
2.52 DMSO
2.51 DMSO
2.51 DMSO
2.50 DMSO
2.50 DMSO
1.69
1.00
1.21
3.10
13.0 12.5 12.0 11.5 11.0 10.5 10.0 9.5 9.0 8.5 8.0 7.5 7.0 6.5 6.0 5.5 5.0 4.5 4.0 3.5 3.0 2.5 2.0 1.5 1.0 0.5

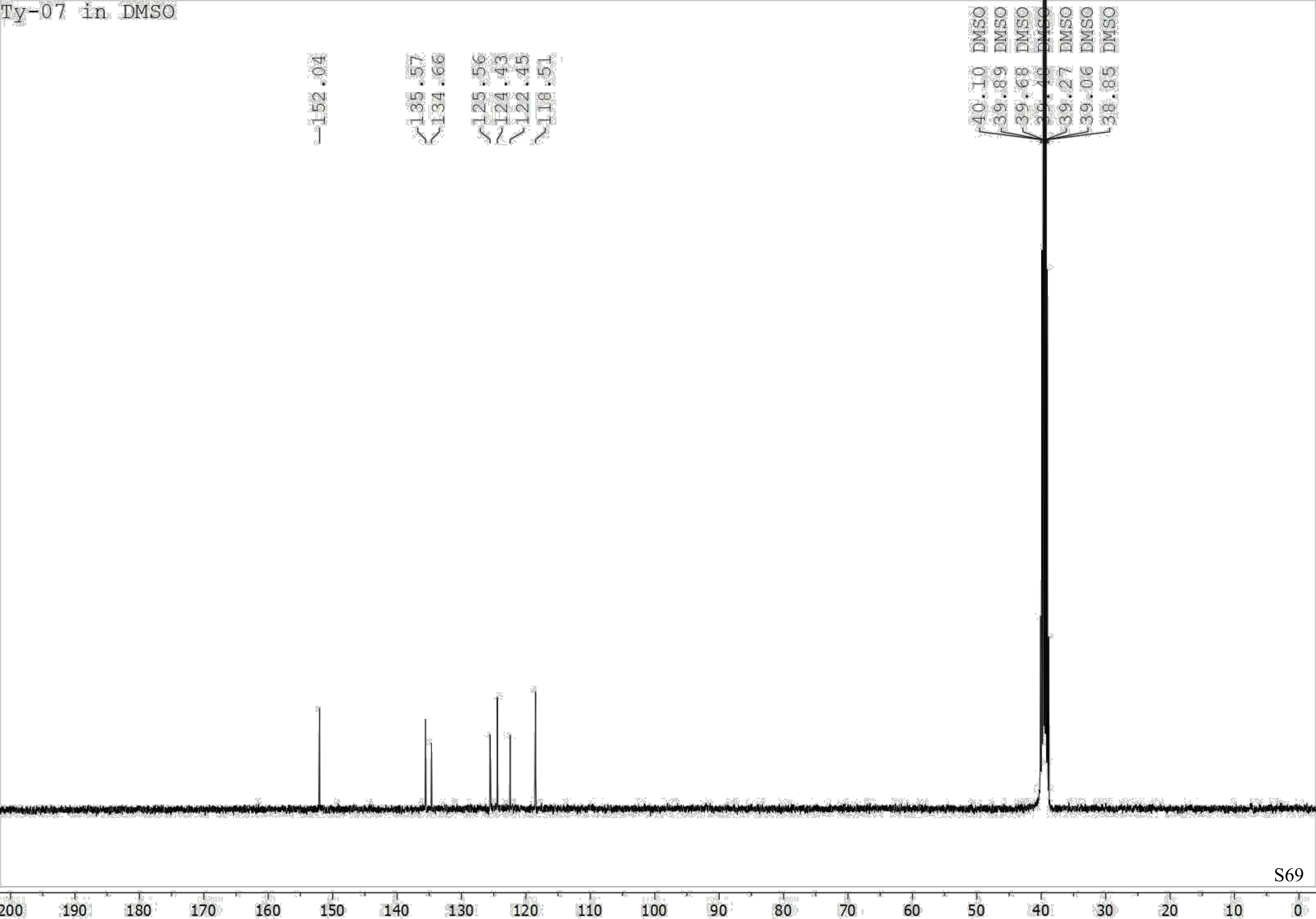
Ty-07 in DMSO
152.04
135.57
134.66
125.56
124.43
122.45
118.51
40.10 DMSO
39.89 DMSO
39.68 DMSO
39.48 DMSO
39.27 DMSO
39.06 DMSO
38.85 DMSO
200
190
180
170
160
150
140
130
120
110
100
90
80
70
60
50
40
30
20
10
0

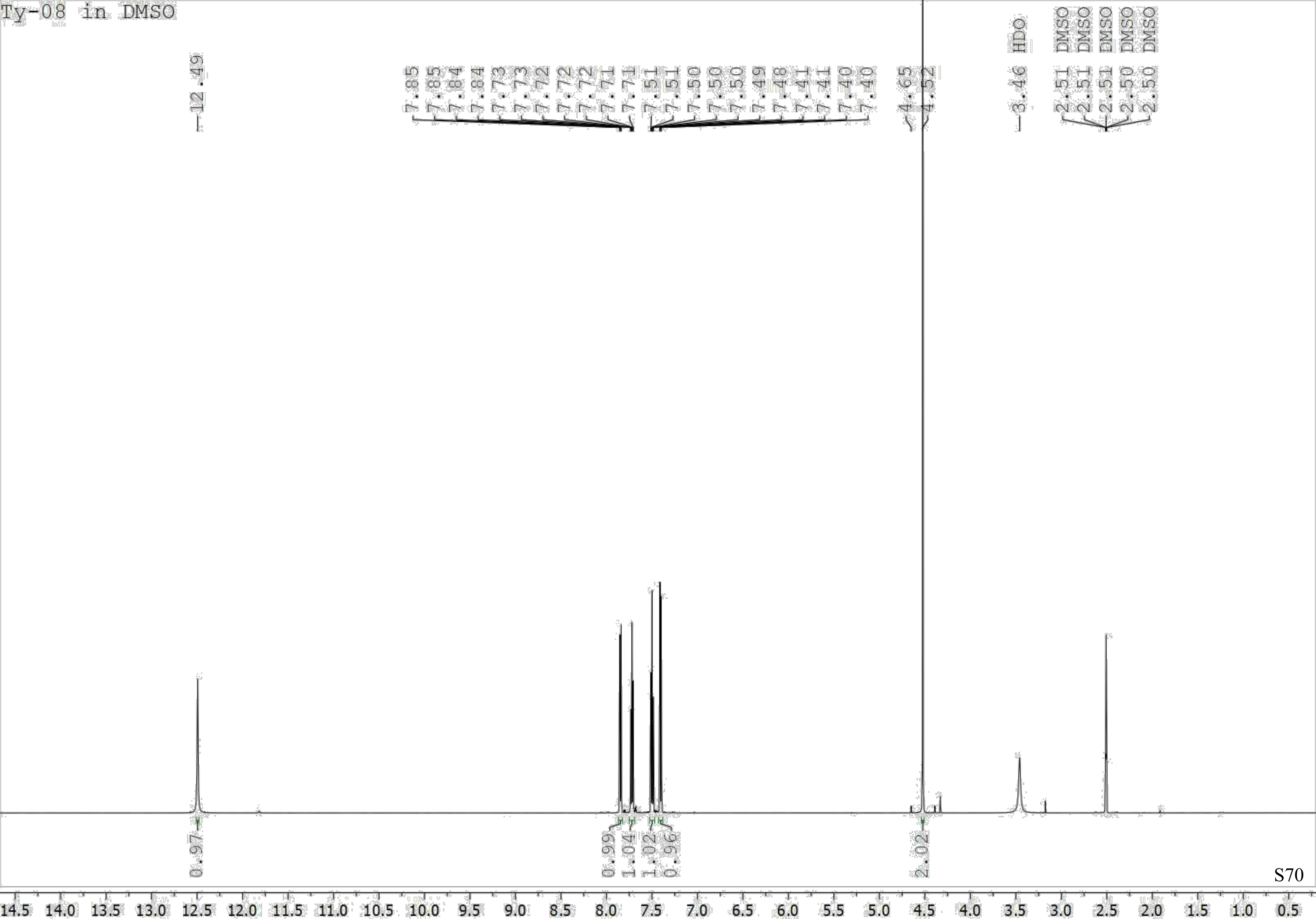
Ty-08 in DMSO
12.49
7.85 7.85 7.84 7.84 7.73 7.73 7.72 7.72 7.72 7.71 7.71 7.51 7.51 7.50 7.50 7.50 7.49 7.48 7.41 7.41 7.40 7.40
4.65 4.52
3.46 HDO
2.51 DMSO 2.51 DMSO 2.51 DMSO 2.50 DMSO 2.50 DMSO
0.97
0.99 1.04 1.02 0.96
2.02
14.5 14.0 13.5 13.0 12.5 12.0 11.5 11.0 10.5 10.0 9.5 9.0 8.5 8.0 7.5 7.0 6.5 6.0 5.5 5.0 4.5 4.0 3.5 3.0 2.5 2.0 1.5 1.0 0.5

Ty-08 in DMSO

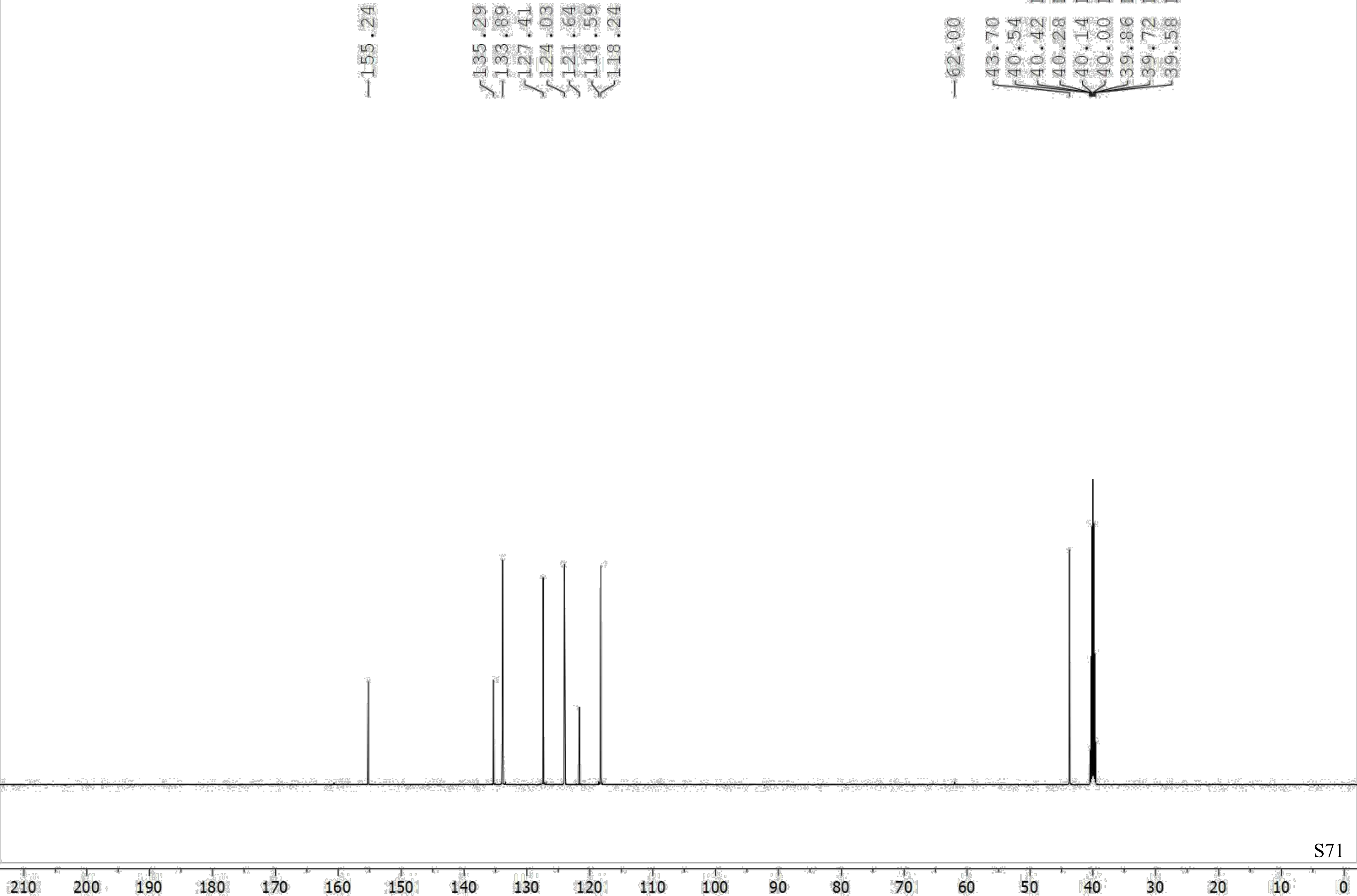
155.24
135.29
133.89
127.41
124.03
121.64
118.59
118.24
62.00
43.70
40.54 DMSO
40.42 DMSO
40.28 DMSO
40.14 DMSO
40.00 DMSO
39.86 DMSO
39.72 DMSO
39.58
210 200 190 180 170 160 150 140 130 120 110 100 90 80 70 60 50 40 30 20 10 0

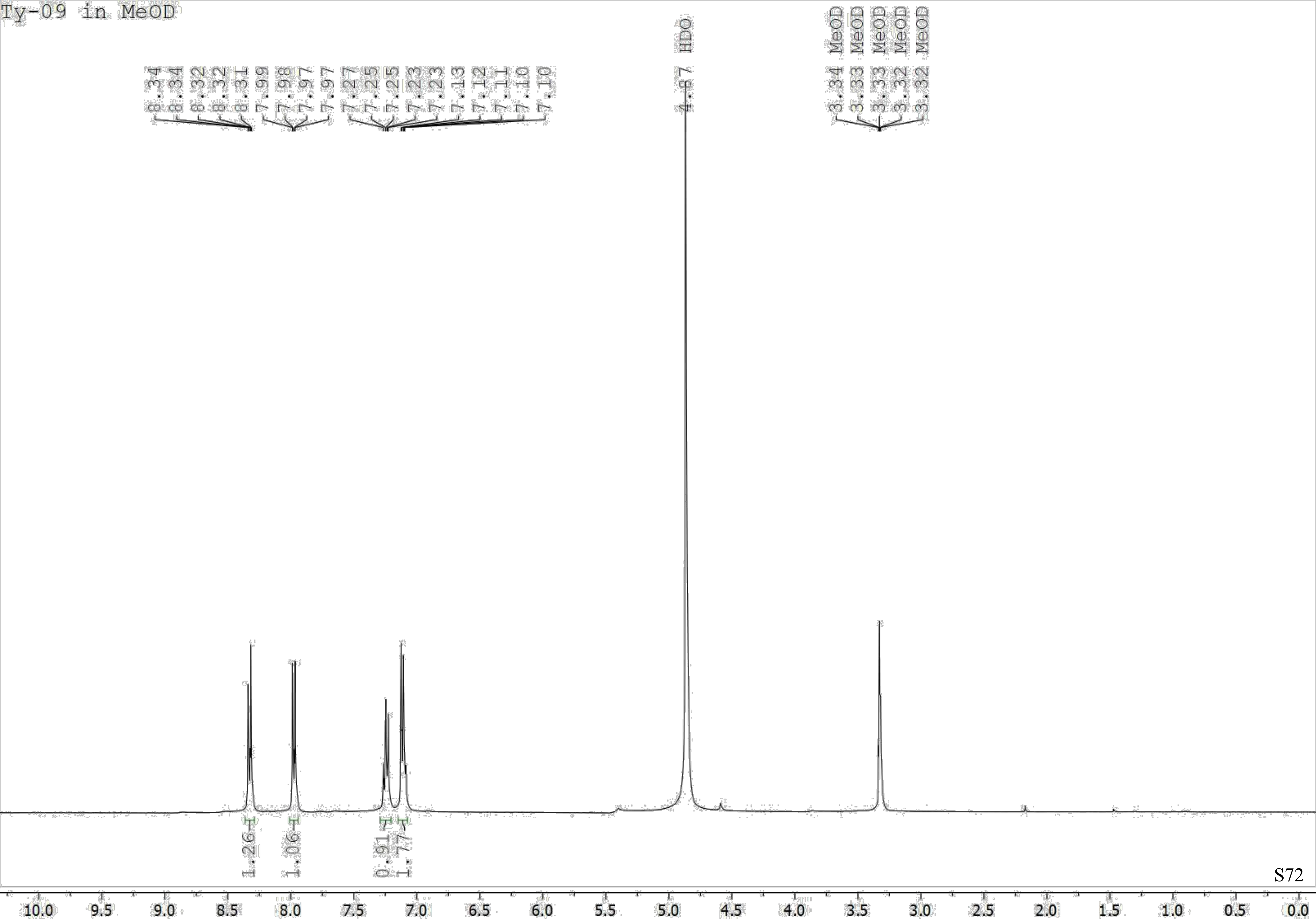
Ty-09 in MeOD
8.34
8.34
8.32
8.32
8.31
7.99
7.98
7.97
7.97
7.27
7.25
7.25
7.23
7.23
7.13
7.12
7.11
7.10
7.10
4.87 HDO
3.34 MeOD
3.33 MeOD
3.33 MeOD
3.32 MeOD
3.32 MeOD
1.26
1.06
0.91
1.77
10.0
9.5
9.0
8.5
8.0
7.5
7.0
6.5
6.0
5.5
5.0
4.5
4.0
3.5
3.0
2.5
2.0
1.5
1.0
0.5
0.0

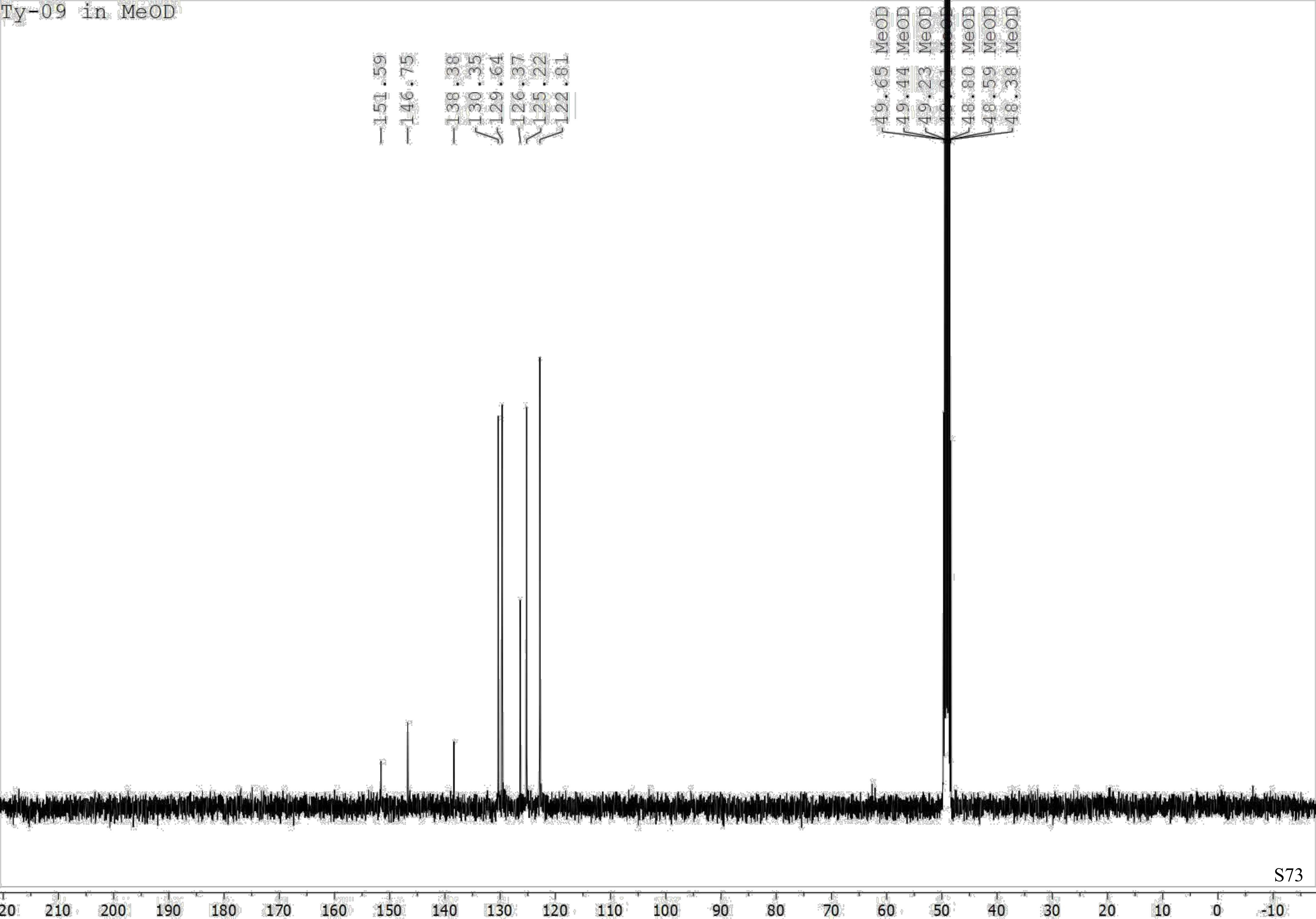
Ty-09 in MeOD
151.59
146.75
138.38
130.35
129.64
126.37
125.22
122.81
49.65 MeOD
49.44 MeOD
49.23 MeOD
48.80 MeOD
48.59 MeOD
48.38 MeOD
210
200
190
180
170
160
150
140
130
120
110
100
90
80
70
60
50
40
30
20
10
0
-10

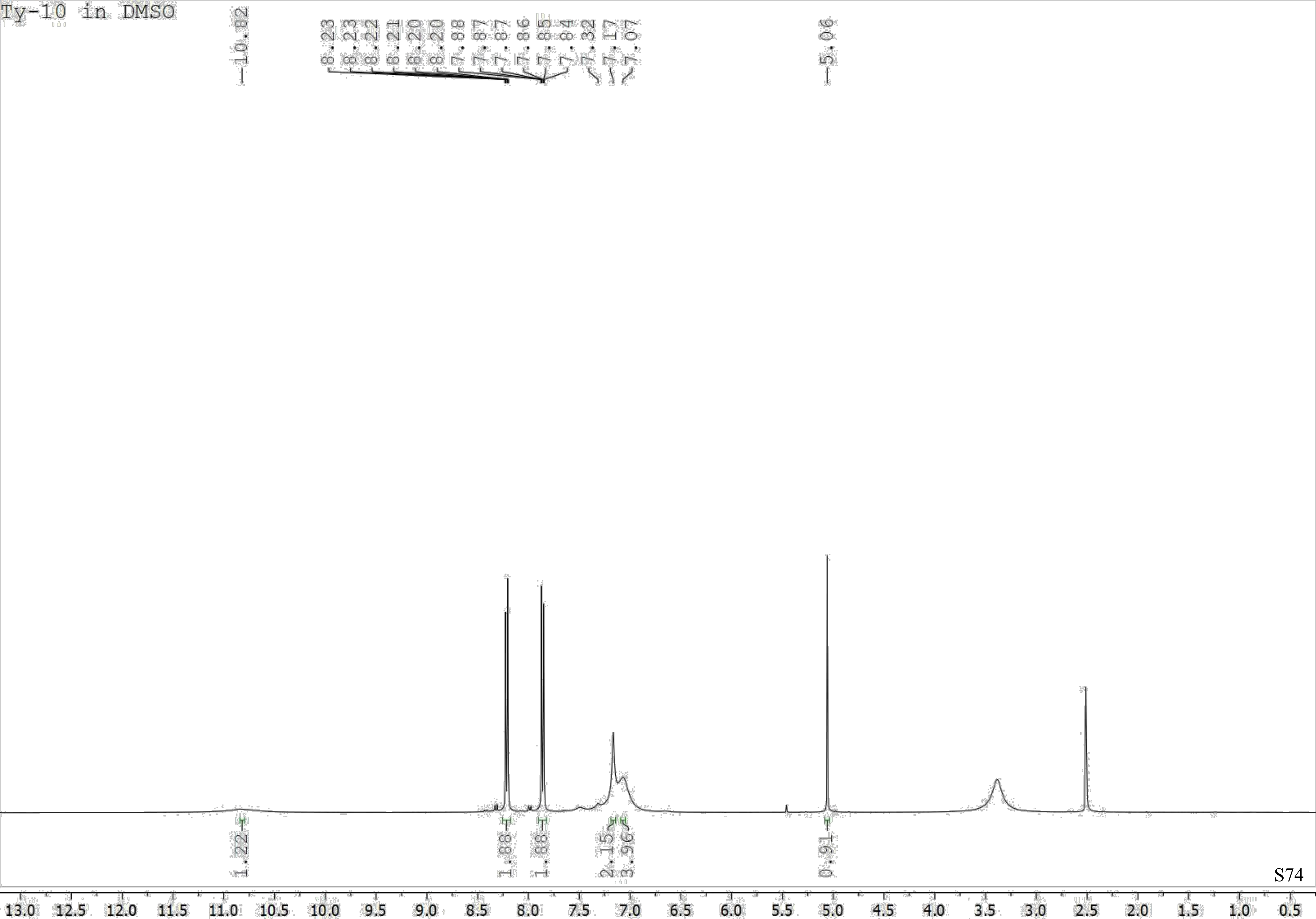
Ty-10 in DMSO
10.82
8.23
8.23
8.22
8.21
8.20
8.20
7.88
7.87
7.87
7.86
7.85
7.84
7.32
7.17
7.07
5.06
1.22
1.88
1.88
2.15
3.96
0.91
13.0 12.5 12.0 11.5 11.0 10.5 10.0 9.5 9.0 8.5 8.0 7.5 7.0 6.5 6.0 5.5 5.0 4.5 4.0 3.5 3.0 2.5 2.0 1.5 1.0 0.5

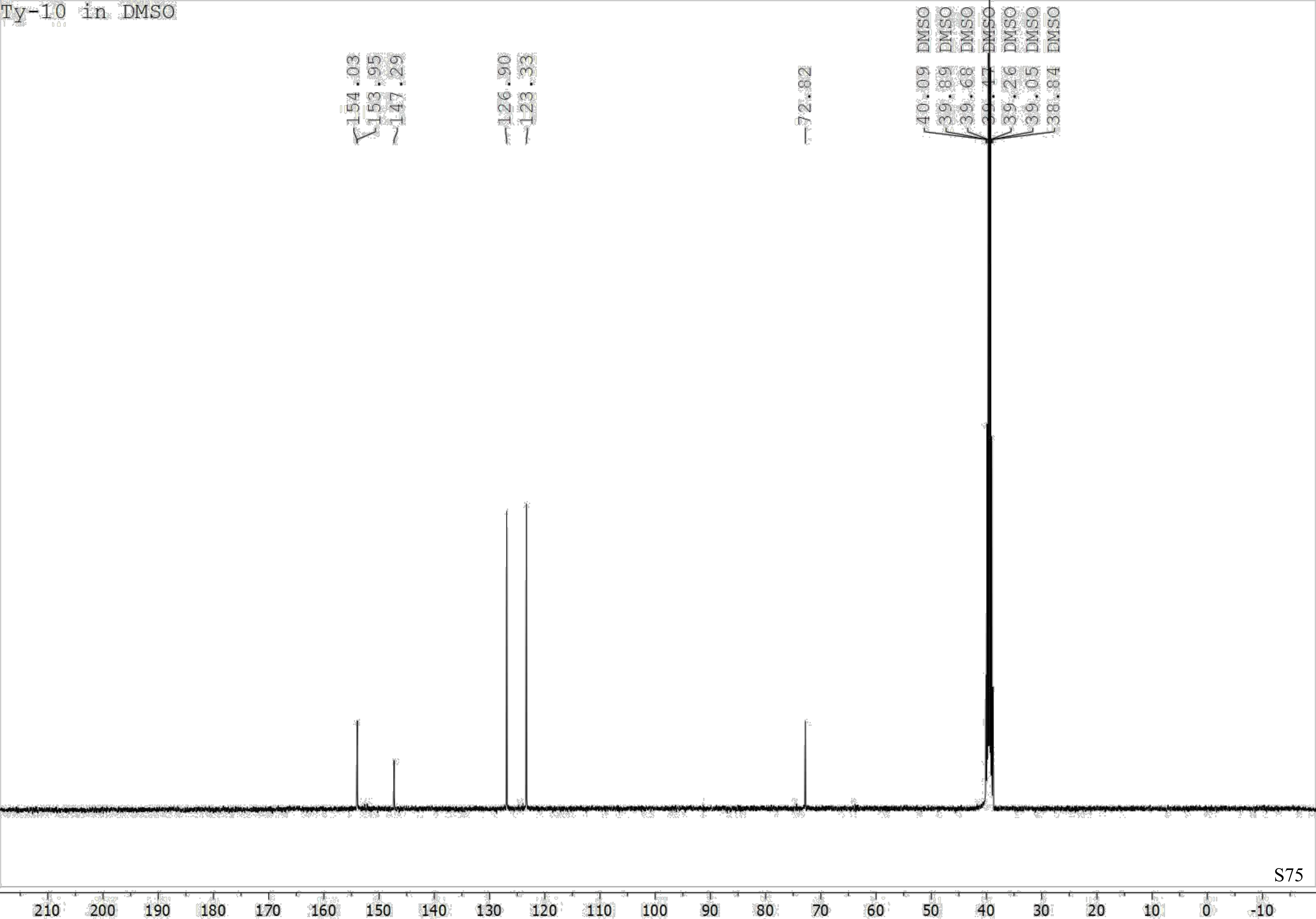
Ty-10 in DMSO
154.03
153.95
147.29
126.90
123.33
72.82
40.09 DMSO
39.89 DMSO
39.68 DMSO
39.47 DMSO
39.26 DMSO
39.05 DMSO
38.84 DMSO
210 200 190 180 170 160 150 140 130 120 110 100 90 80 70 60 50 40 30 20 10 0 -10

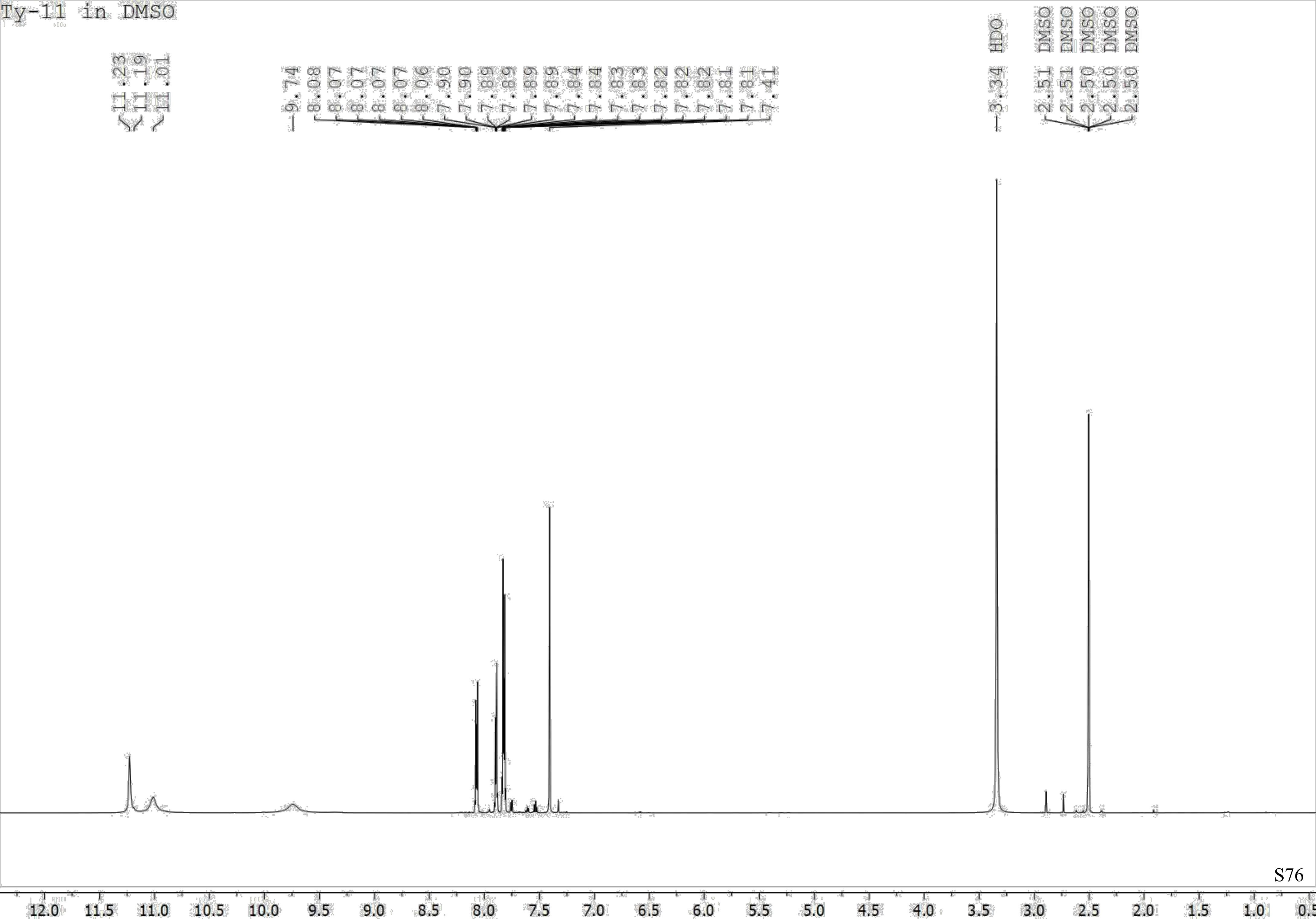
Ty-11 in DMSO
11.23
11.19
11.01
9.74
8.08
8.07
8.07
8.07
8.07
8.06
7.90
7.90
7.89
7.89
7.89
7.89
7.84
7.84
7.83
7.83
7.82
7.82
7.82
7.81
7.81
7.41
3.34 HDO
2.51 DMSO
2.51 DMSO
2.50 DMSO
2.50 DMSO
2.50 DMSO
12.0
11.5
11.0
10.5
10.0
9.5
9.0
8.5
8.0
7.5
7.0
6.5
6.0
5.5
5.0
4.5
4.0
3.5
3.0
2.5
2.0
1.5
1.0
0.5

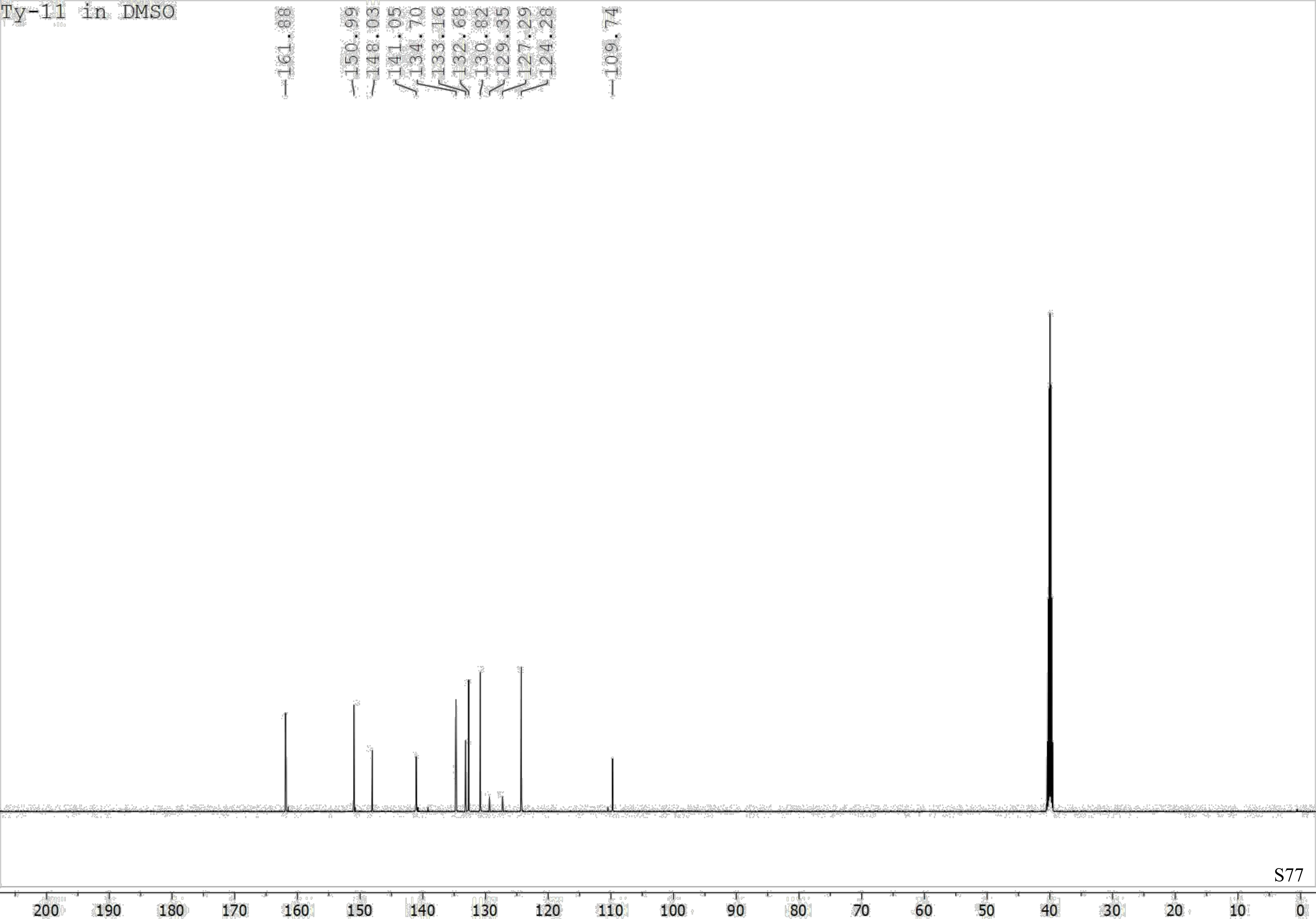
Ty-11 in DMSO
161.88
150.99
148.03
141.05
134.70
133.16
132.68
130.82
129.35
127.29
124.28
109.74
200 190 180 170 160 150 140 130 120 110 100 90 80 70 60 50 40 30 20 10 0

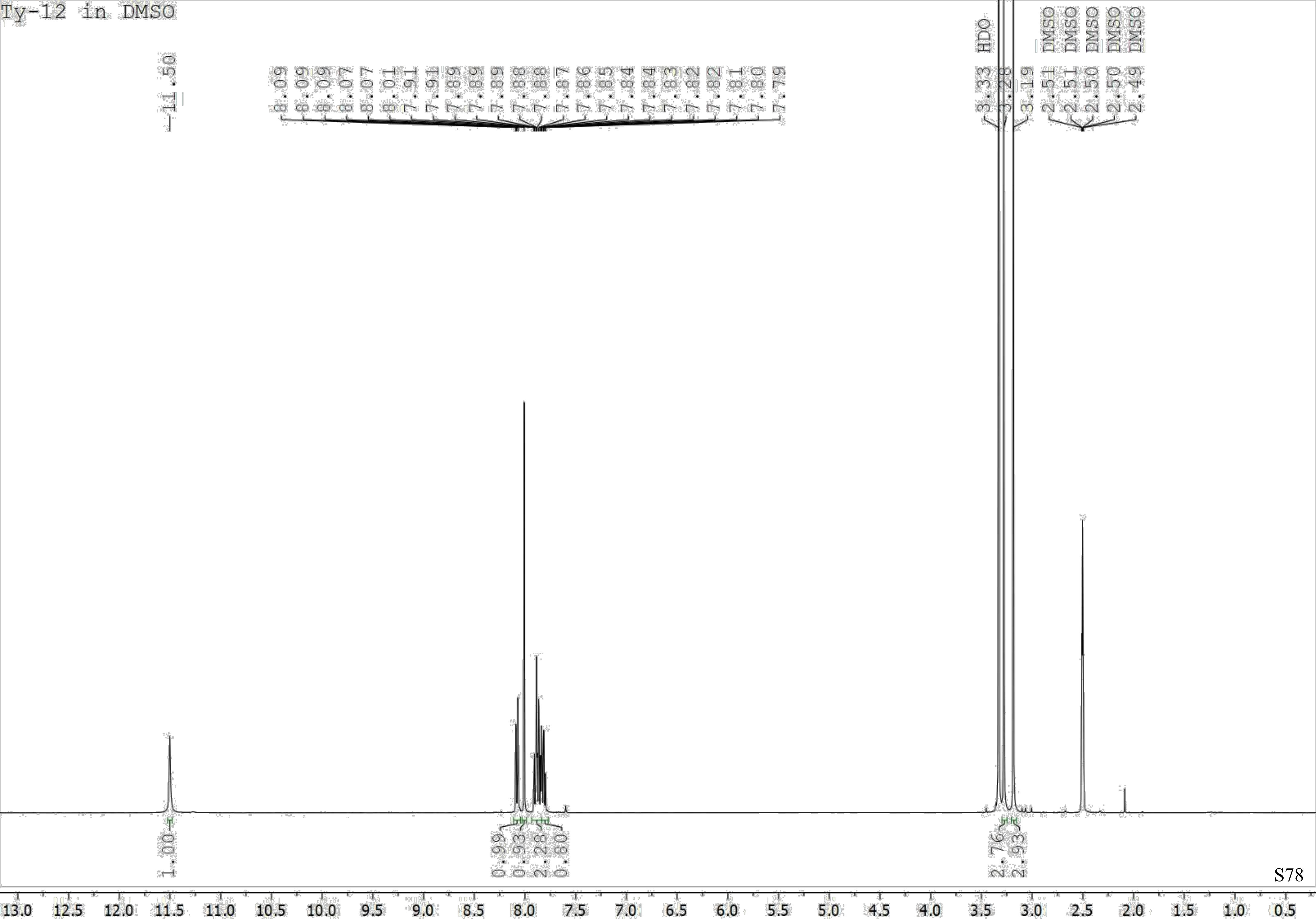
Ty-12 in DMSO
11.50
8.09 8.09 8.09 8.07 8.07 8.01 7.91 7.91 7.89 7.89 7.89 7.88 7.88 7.87 7.86 7.85 7.84 7.84 7.83 7.82 7.82 7.81 7.80 7.79
HDO
3.33 3.28 3.19
DMSO DMSO DMSO DMSO DMSO
2.51 2.51 2.50 2.50 2.49
1.00
0.99 0.93 2.28 0.80
2.76 2.93
13.0 12.5 12.0 11.5 11.0 10.5 10.0 9.5 9.0 8.5 8.0 7.5 7.0 6.5 6.0 5.5 5.0 4.5 4.0 3.5 3.0 2.5 2.0 1.5 1.0 0.5

Ty-12 in DMSO

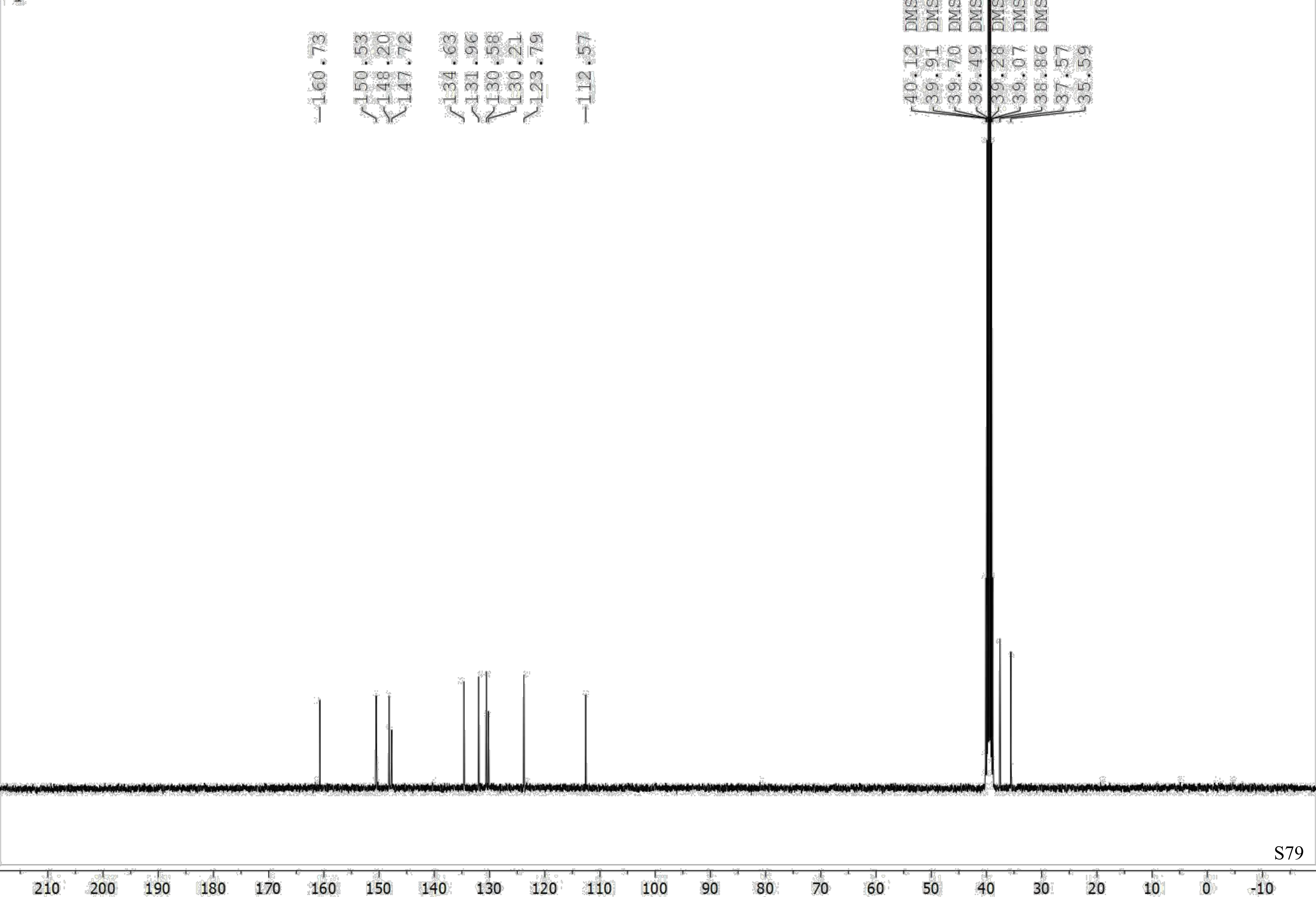
160.73
150.53
148.20
147.72
134.63
131.96
130.58
130.21
123.79
112.57
40.12 DMSO
39.91 DMSO
39.70 DMSO
39.49 DMSO
39.28 DMSO
39.07 DMSO
38.86 DMSO
37.57
35.59
210
200
190
180
170
160
150
140
130
120
110
100
90
80
70
60
50
40
30
20
10
0
-10

Ty-13 in DMSO

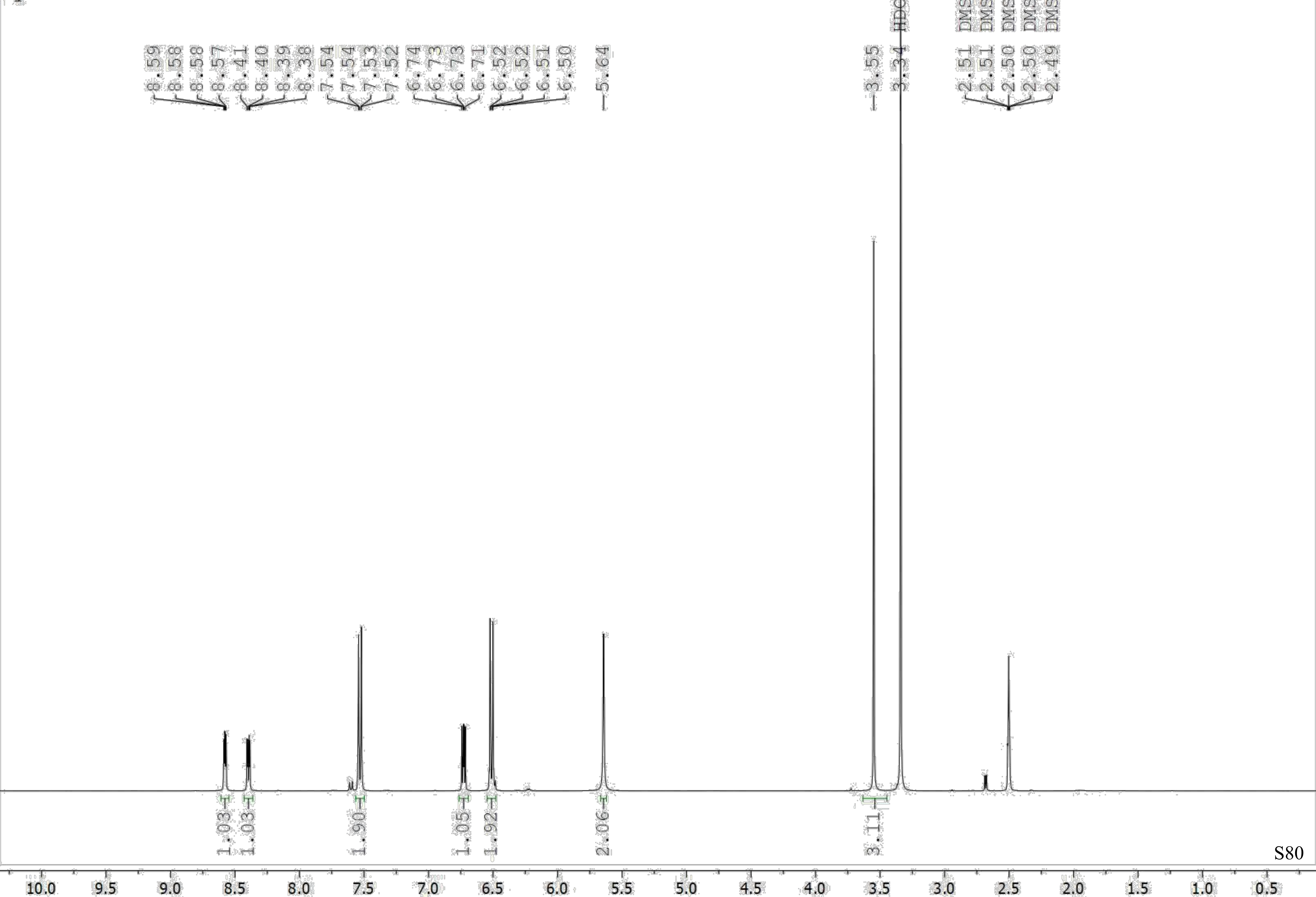

8.59
8.58
8.58
8.57
8.41
8.40
8.39
8.38
7.54
7.54
7.53
7.52
6.74
6.73
6.73
6.71
6.52
6.52
6.51
6.50
5.64
3.55
3.34 HDO
2.51 DMSO
2.51 DMSO
2.50 DMSO
2.50 DMSO
2.49 DMSO
1.03
1.03
1.90
1.05
1.92
2.06
3.11
10.0
9.5
9.0
8.5
8.0
7.5
7.0
6.5
6.0
5.5
5.0
4.5
4.0
3.5
3.0
2.5
2.0
1.5
1.0
0.5

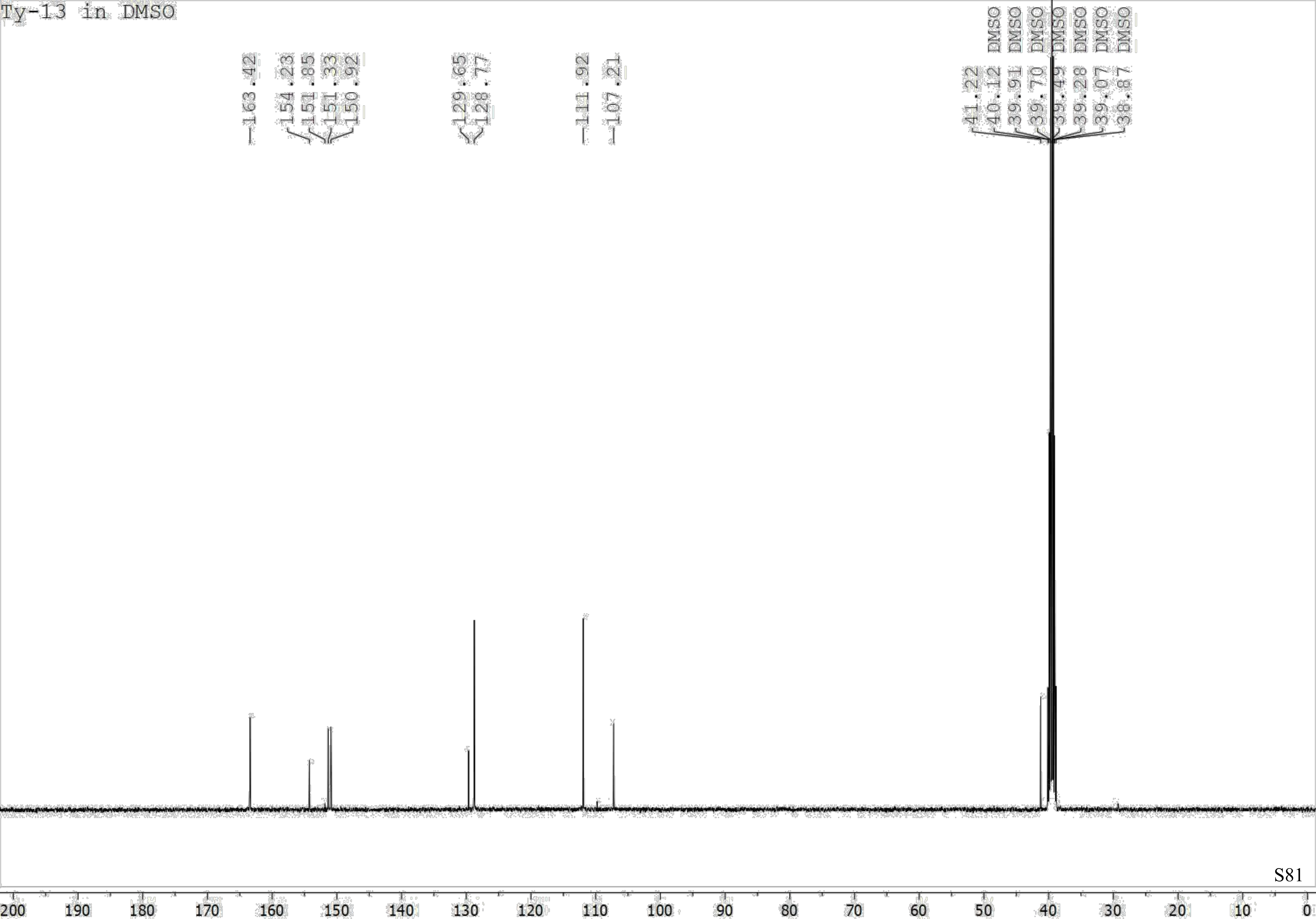
Ty-13 in DMSO
163.42
154.23
151.85
151.33
150.92
129.65
128.77
111.92
107.21
41.22
40.12
39.91
39.70
39.49
39.28
39.07
38.87
DMSO
DMSO
DMSO
DMSO
DMSO
DMSO
DMSO
200 190 180 170 160 150 140 130 120 110 100 90 80 70 60 50 40 30 20 10 0

Ty-14 in MeOD

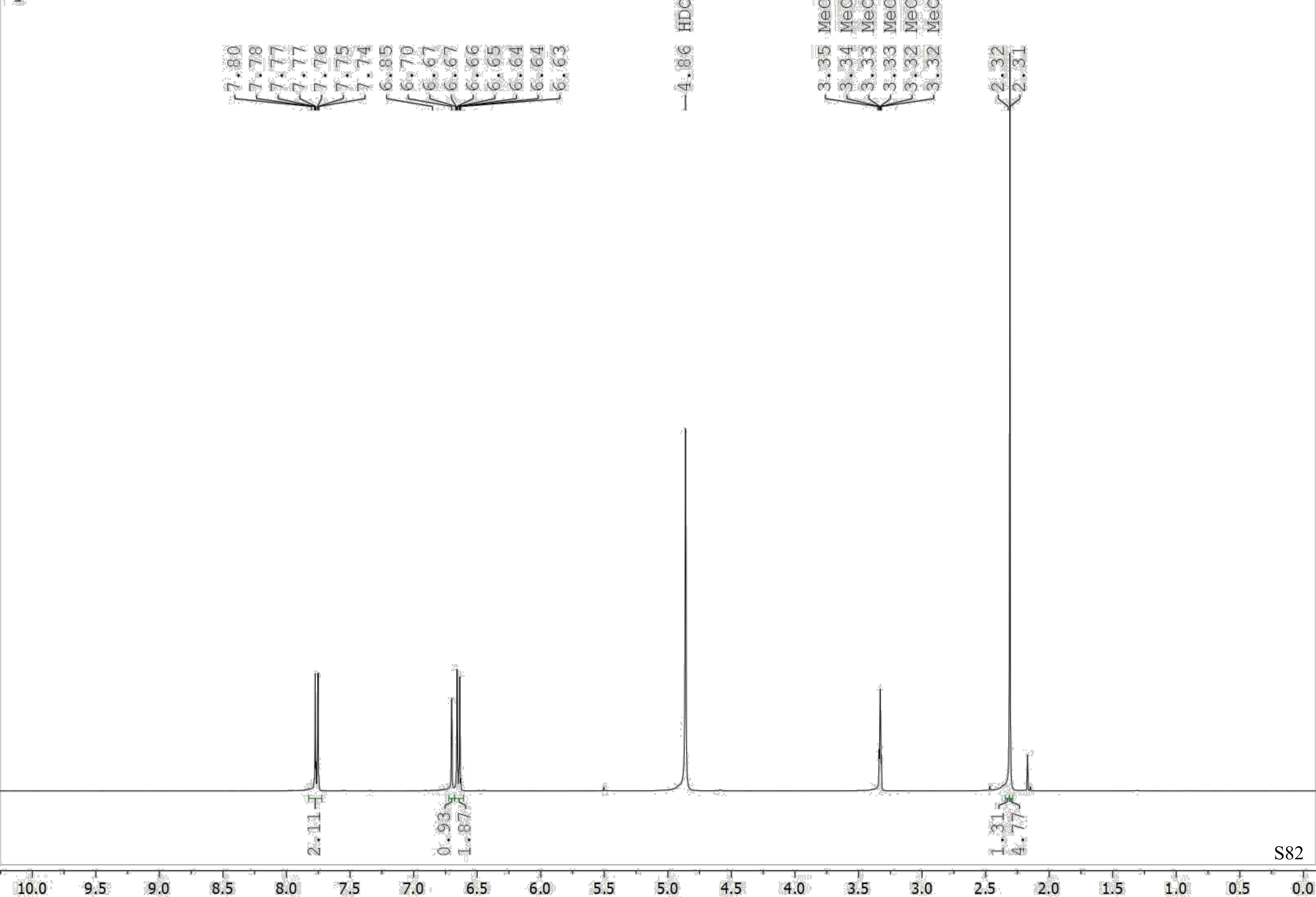

7.80
7.78
7.77
7.77
7.76
7.75
7.74
6.85
6.70
6.67
6.67
6.66
6.65
6.64
6.64
6.63
4.86 HDO
3.35 MeOD
3.34 MeOD
3.33 MeOD
3.33 MeOD
3.32 MeOD
3.32 MeOD
2.32
2.31
2.11
0.93
1.87
1.31
4.77
10.0 9.5 9.0 8.5 8.0 7.5 7.0 6.5 6.0 5.5 5.0 4.5 4.0 3.5 3.0 2.5 2.0 1.5 1.0 0.5 0.0

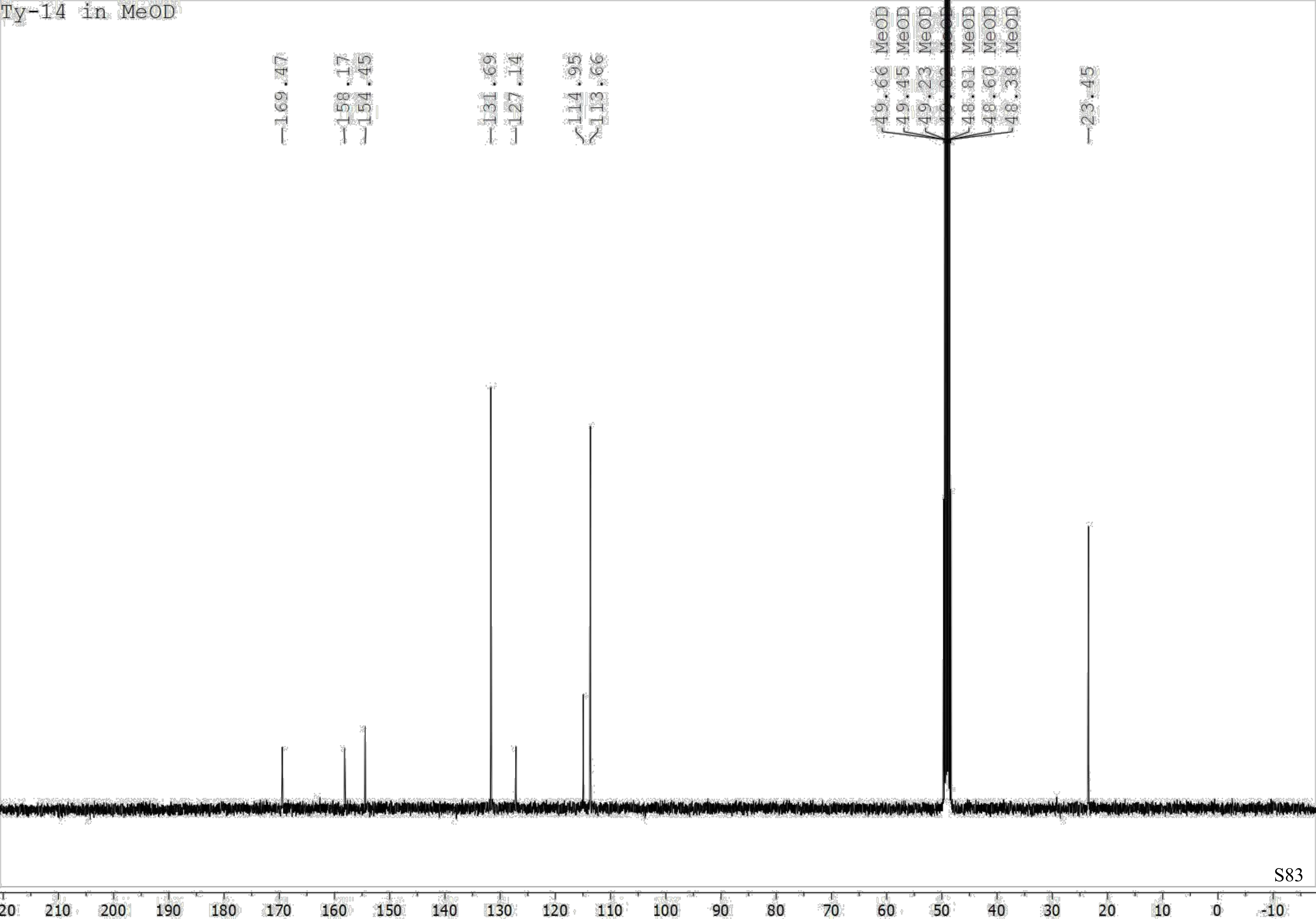

Ty-14 in MeOD
169.47
158.17
154.45
131.69
127.14
114.95
113.66
49.66 MeOD
49.45 MeOD
49.23 MeOD
48.81 MeOD
48.60 MeOD
48.38 MeOD
23.45
220
210
200
190
180
170
160
150
140
130
120
110
100
90
80
70
60
50
40
30
20
10
0
-10

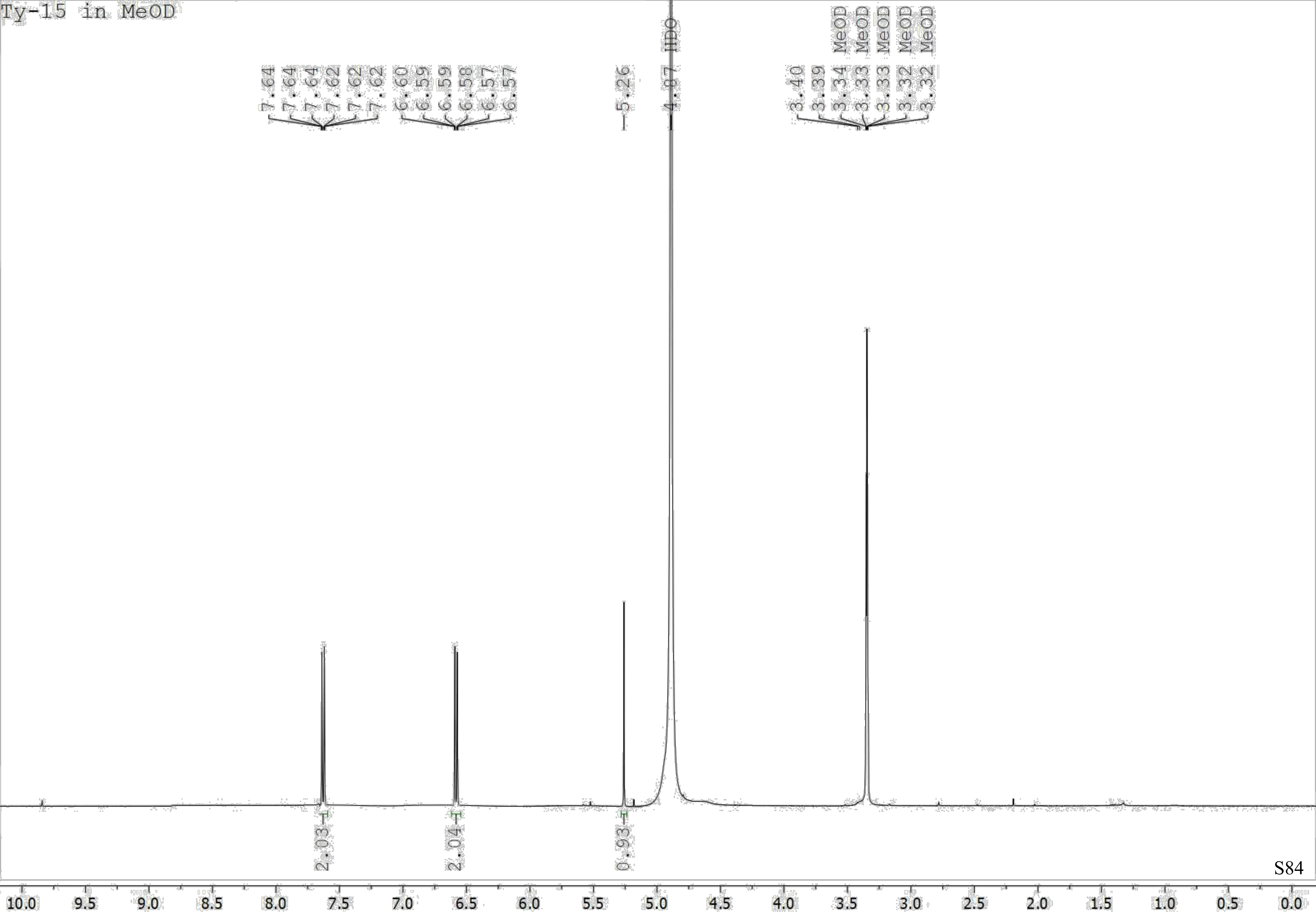
Ty-15 in MeOD
7.64
7.64
7.64
7.62
7.62
7.62
6.60
6.59
6.59
6.58
6.57
6.57
5.26
4.87
HDO
3.40
3.39
3.34
3.33
3.33
3.32
3.32
MeOD
MeOD
MeOD
MeOD
MeOD
2.03
2.04
0.93
10.0
9.5
9.0
8.5
8.0
7.5
7.0
6.5
6.0
5.5
5.0
4.5
4.0
3.5
3.0
2.5
2.0
1.5
1.0
0.5
0.0

Ty-15 in MeOD

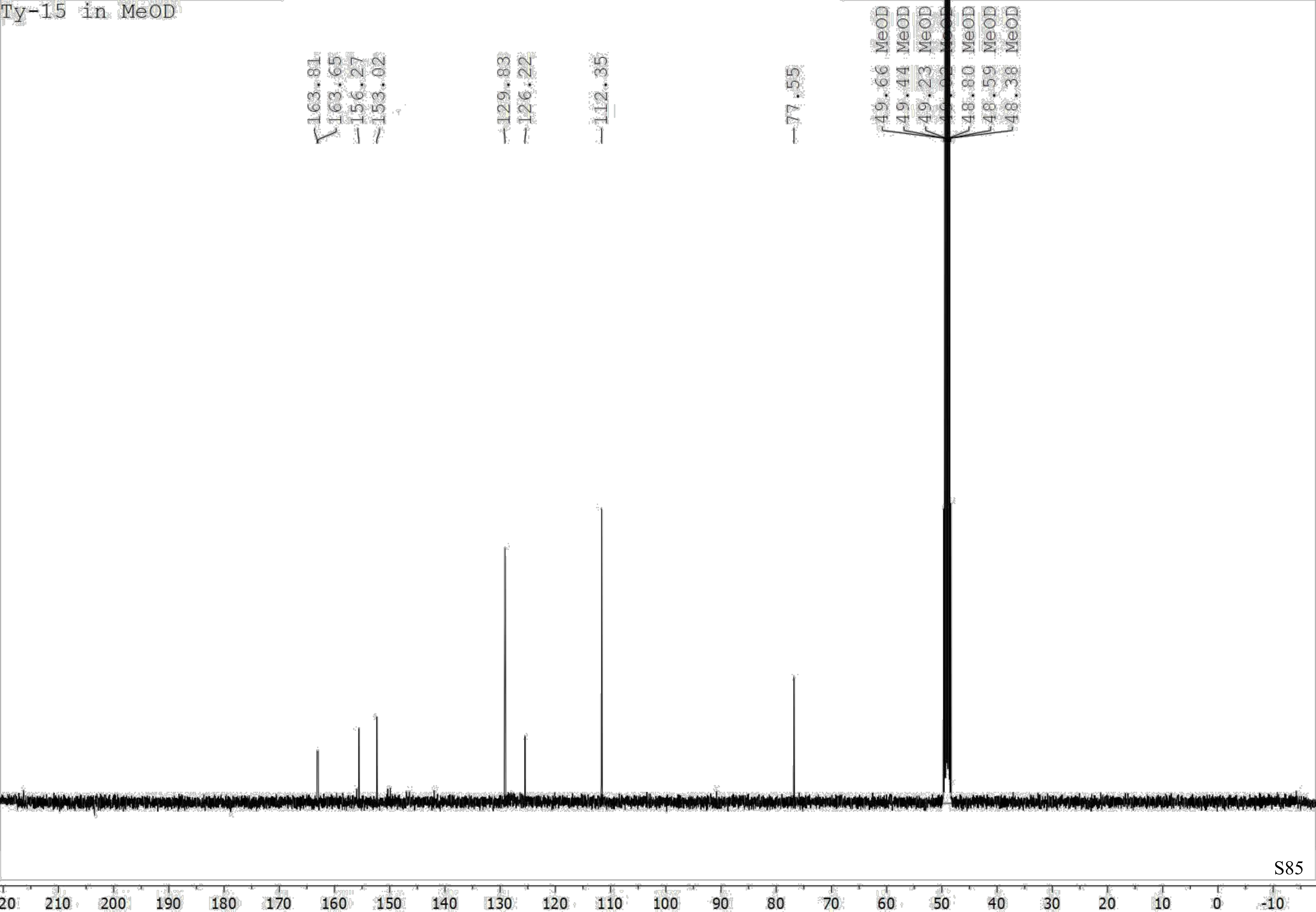
163.81
163.65
156.27
153.02
129.83
126.22
112.35
77.55
49.66 MeOD
49.44 MeOD
49.23 MeOD
48.80 MeOD
48.59 MeOD
48.38 MeOD
210
200
190
180
170
160
150
140
130
120
110
100
90
80
70
60
50
40
30
20
10
0
-10

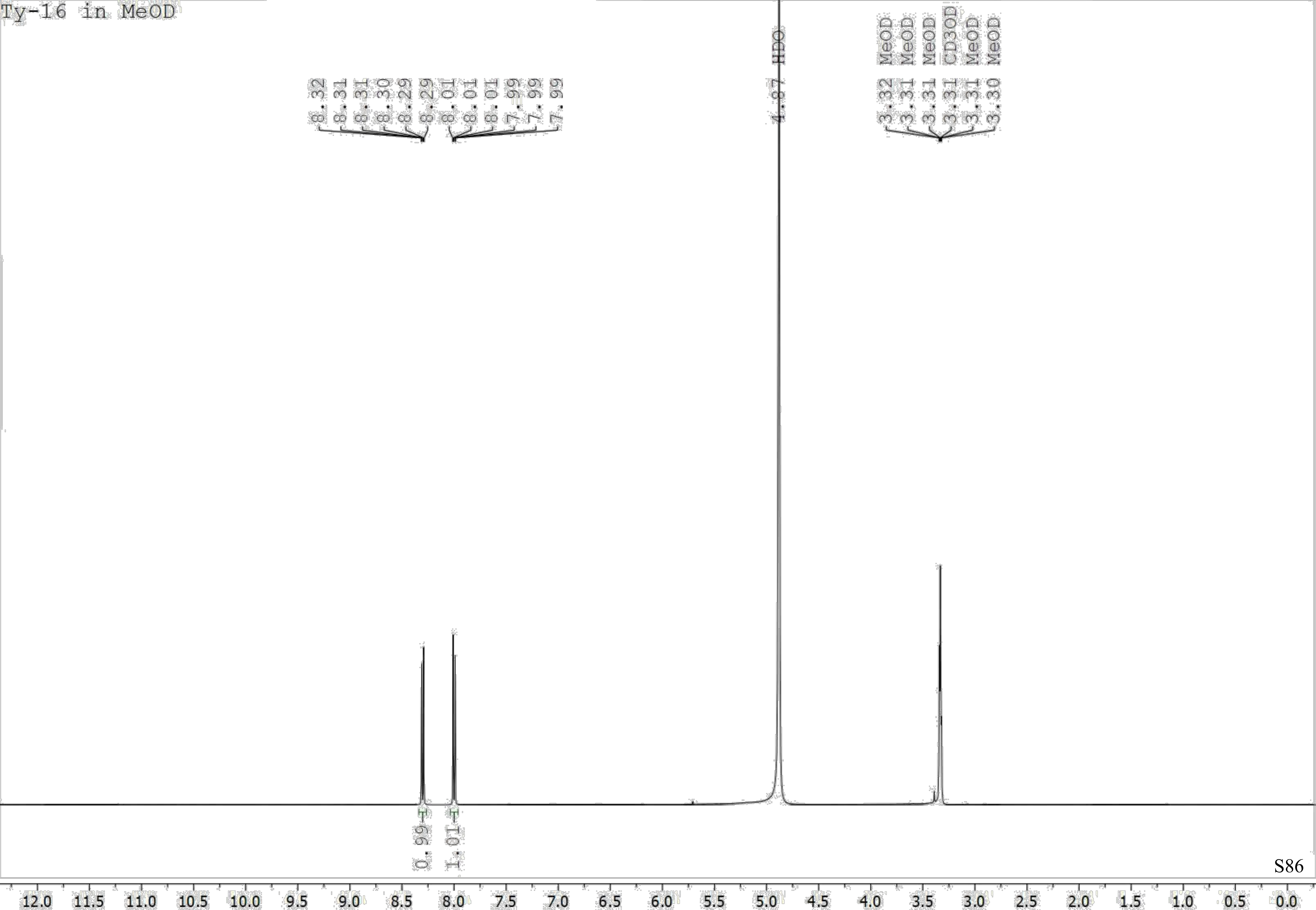
Ty-16 in MeOD
8.32
8.31
8.31
8.30
8.29
8.29
8.01
8.01
8.01
7.99
7.99
7.99
4.87 HDO
3.32 MeOD
3.31 MeOD
3.31 MeOD
3.31 CD3OD
3.31 MeOD
3.30 MeOD
0.99
1.01
12.0
11.5
11.0
10.5
10.0
9.5
9.0
8.5
8.0
7.5
7.0
6.5
6.0
5.5
5.0
4.5
4.0
3.5
3.0
2.5
2.0
1.5
1.0
0.5
0.0

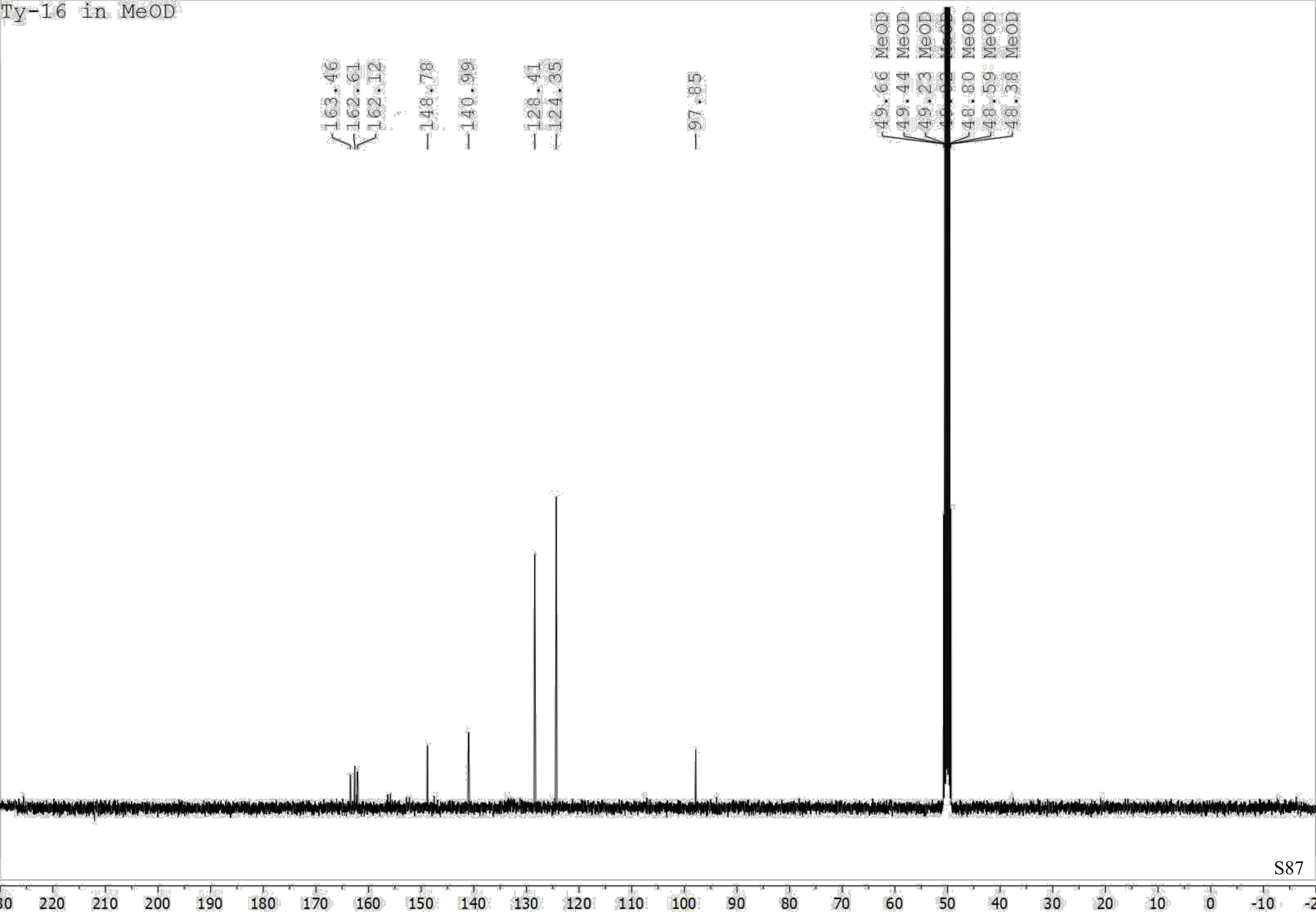
Ty-16 in MeOD
163.46
162.61
162.12
148.78
140.99
128.41
124.35
97.85
49.66 MeOD
49.44 MeOD
49.23 MeOD
MeOD
48.80 MeOD
48.59 MeOD
48.38 MeOD
220 210 200 190 180 170 160 150 140 130 120 110 100 90 80 70 60 50 40 30 20 10 0 -10

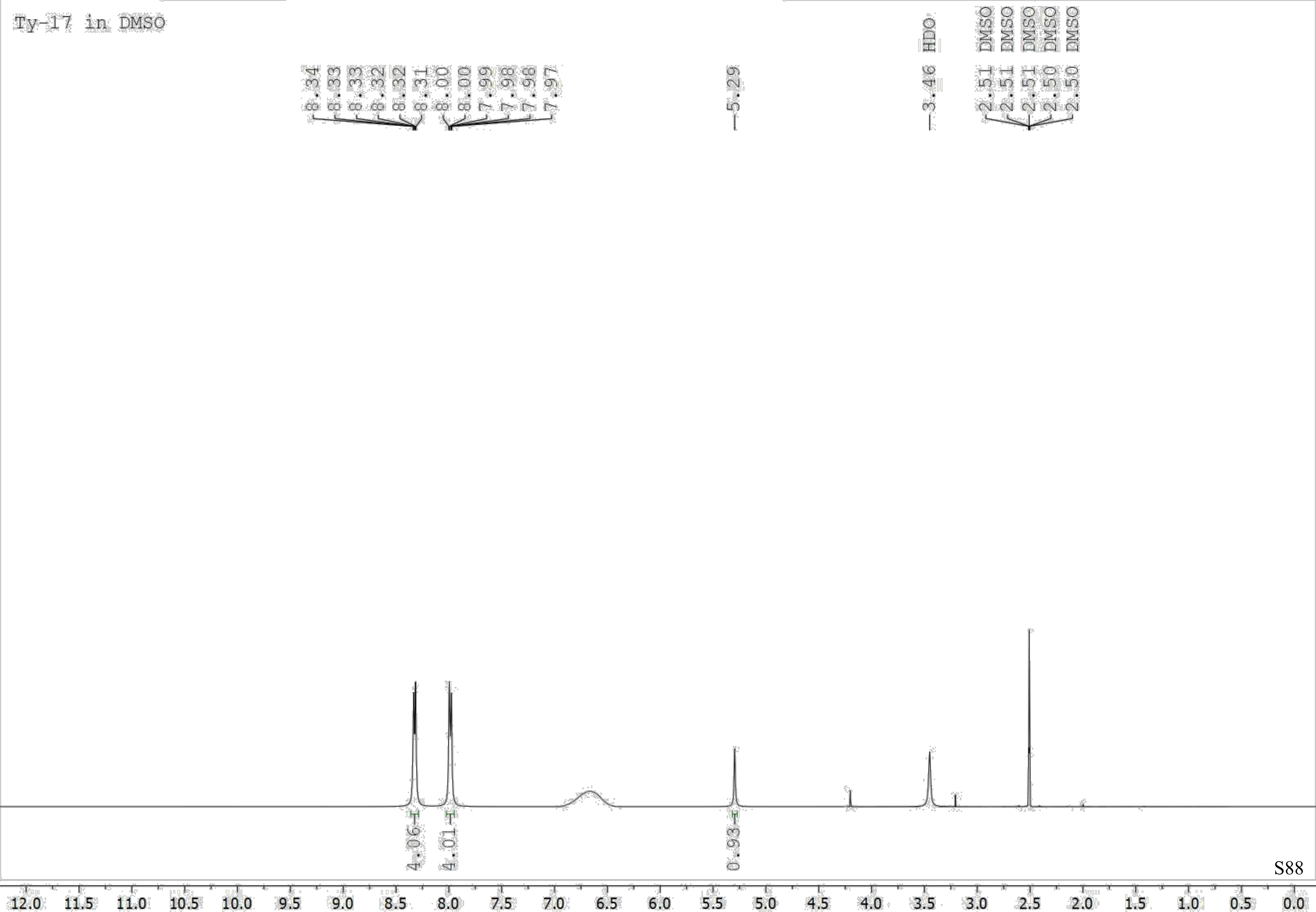
Ty-17 in DMSO
8.34
8.33
8.33
8.32
8.32
8.31
8.00
8.00
7.99
7.98
7.98
7.97
5.29
HDO
3.46
DMSO
DMSO
DMSO
DMSO
DMSO
2.51
2.51
2.51
2.50
2.50
4.06
4.01
0.93
12.0 11.5 11.0 10.5 10.0 9.5 9.0 8.5 8.0 7.5 7.0 6.5 6.0 5.5 5.0 4.5 4.0 3.5 3.0 2.5 2.0 1.5 1.0 0.5 0.0

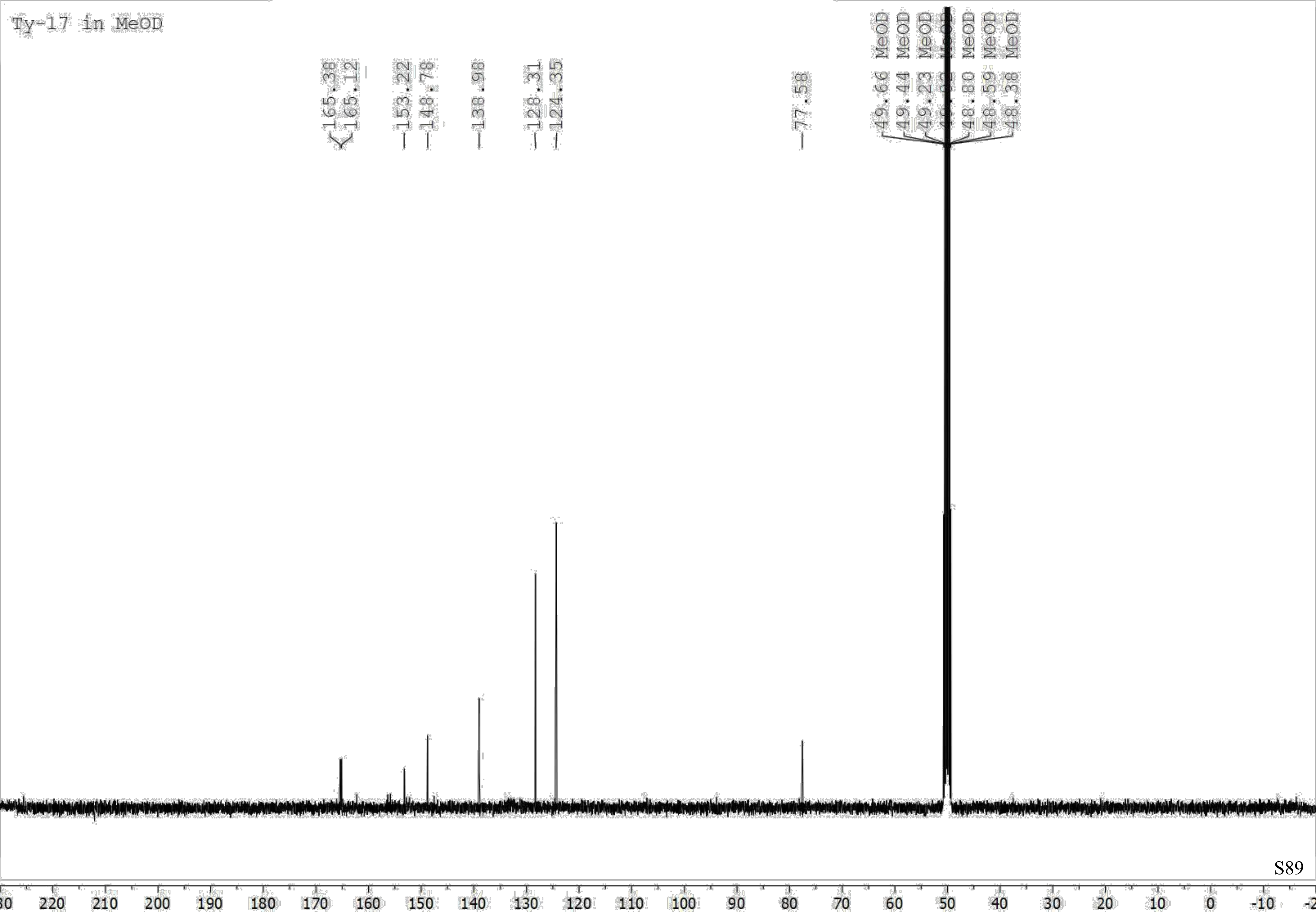
Ty-17 in MeOD
165.38
165.12
153.22
148.78
138.98
128.31
124.35
77.58
49.66 MeOD
49.44 MeOD
49.23 MeOD
48.80 MeOD
48.59 MeOD
48.38 MeOD
220 210 200 190 180 170 160 150 140 130 120 110 100 90 80 70 60 50 40 30 20 10 0 -10

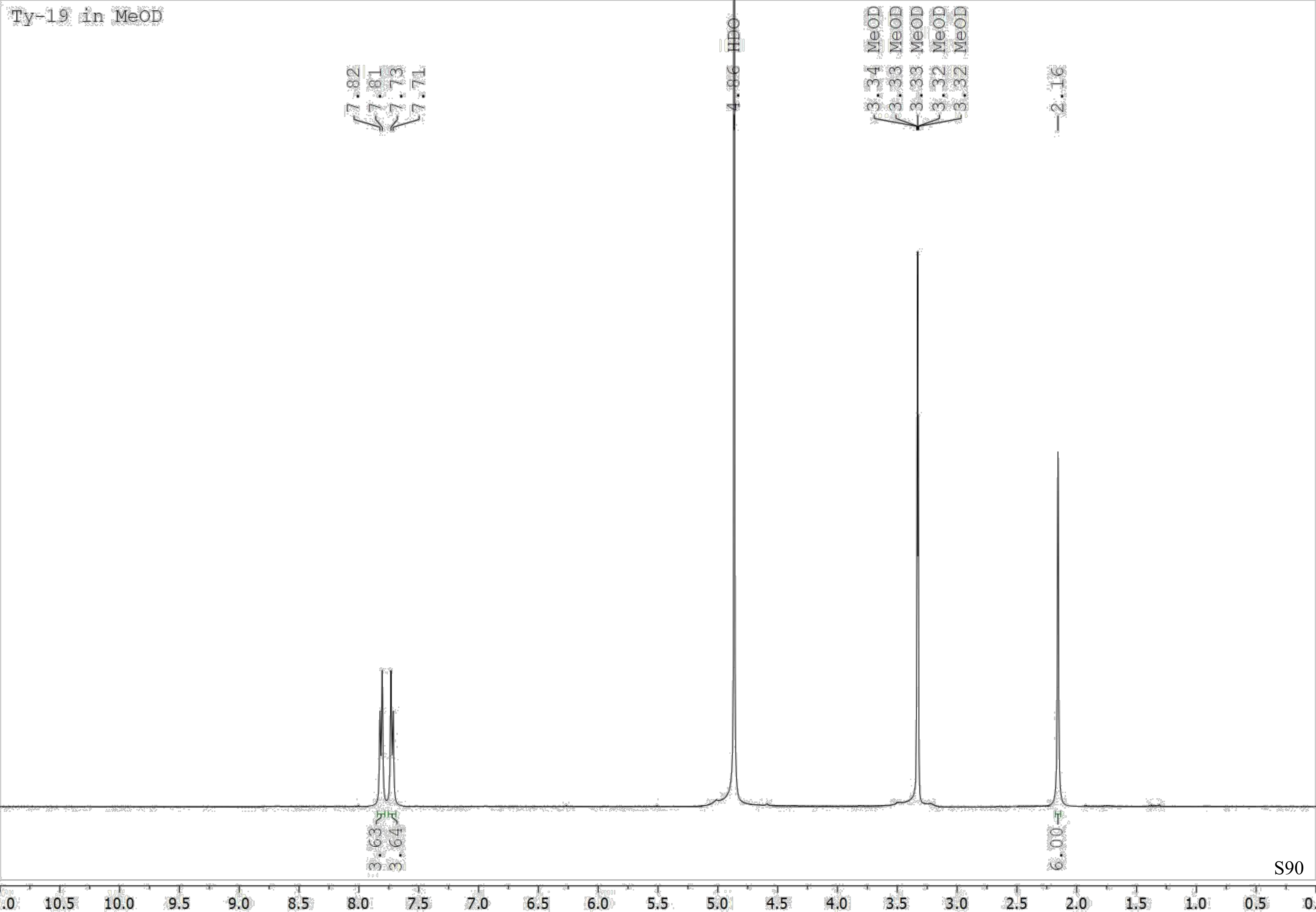
Ty-19 in MeOD
7.82
7.81
7.73
7.71
4.86 HDO
3.34 MeOD
3.33 MeOD
3.33 MeOD
3.32 MeOD
3.32 MeOD
2.16
3.63
3.64
6.00

Ty-18 in MeOD

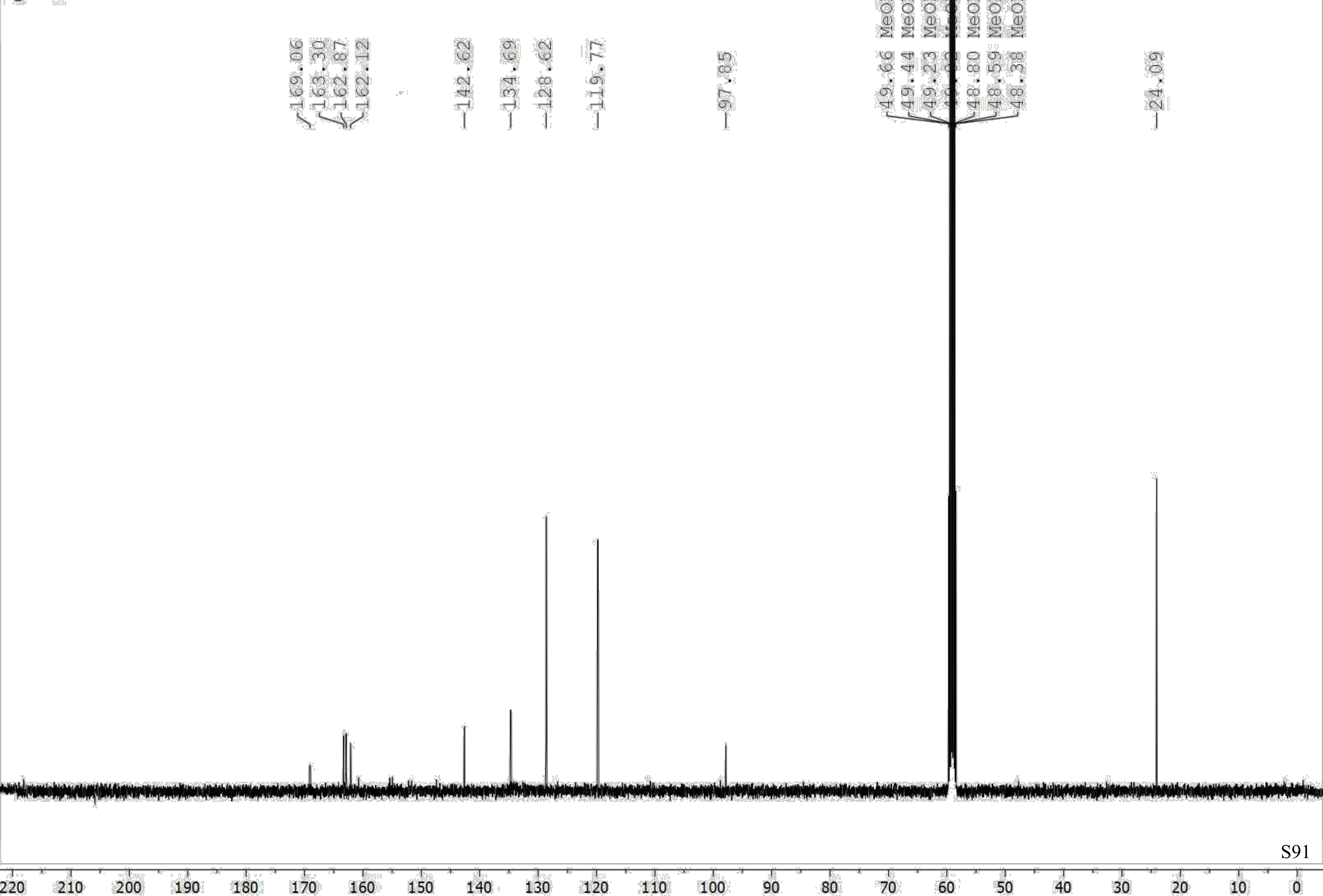
169.06
163.30
162.87
162.12
142.62
134.69
128.62
119.77
97.85
49.66 MeOD
49.44 MeOD
49.23 MeOD
48.80 MeOD
48.59 MeOD
48.38 MeOD
24.09
220 210 200 190 180 170 160 150 140 130 120 110 100 90 80 70 60 50 40 30 20 10 0

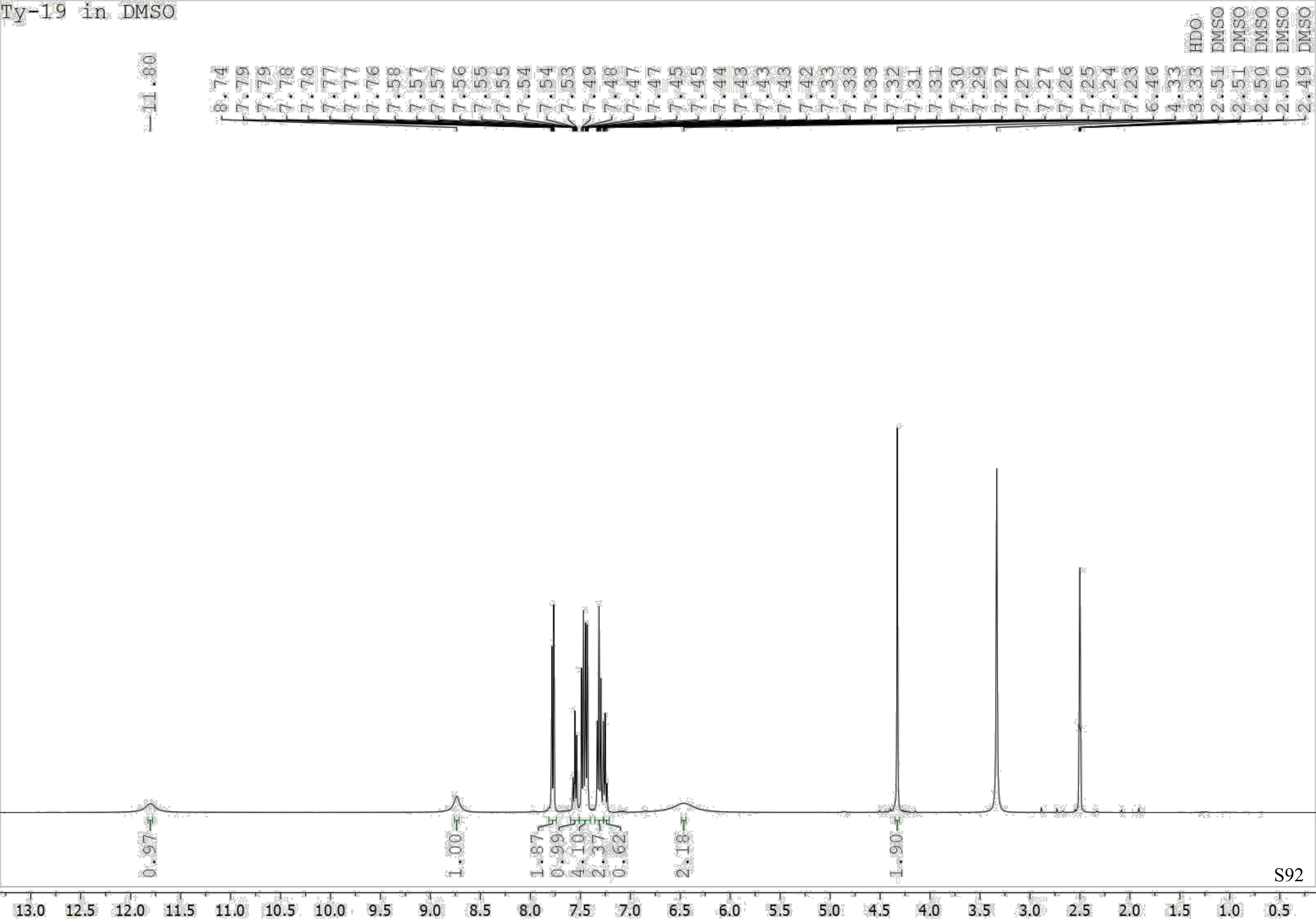
Ty-19 in DMSO
HDO
DMSO
DMSO
DMSO
DMSO
DMSO
11.80
8.74 7.79 7.79 7.78 7.78 7.77 7.77 7.76 7.58 7.57 7.57 7.56 7.55 7.55 7.54 7.54 7.53 7.49 7.48 7.47 7.47 7.45 7.45 7.44 7.43 7.43 7.43 7.42 7.33 7.33 7.33 7.32 7.31 7.31 7.30 7.29 7.27 7.27 7.27 7.26 7.25 7.24 7.23 6.46 4.33 3.33 2.51 2.51 2.50 2.50 2.49
0.97
1.00
1.87
0.99
4.10
2.37
0.62
2.18
1.90
13.0 12.5 12.0 11.5 11.0 10.5 10.0 9.5 9.0 8.5 8.0 7.5 7.0 6.5 6.0 5.5 5.0 4.5 4.0 3.5 3.0 2.5 2.0 1.5 1.0 0.5

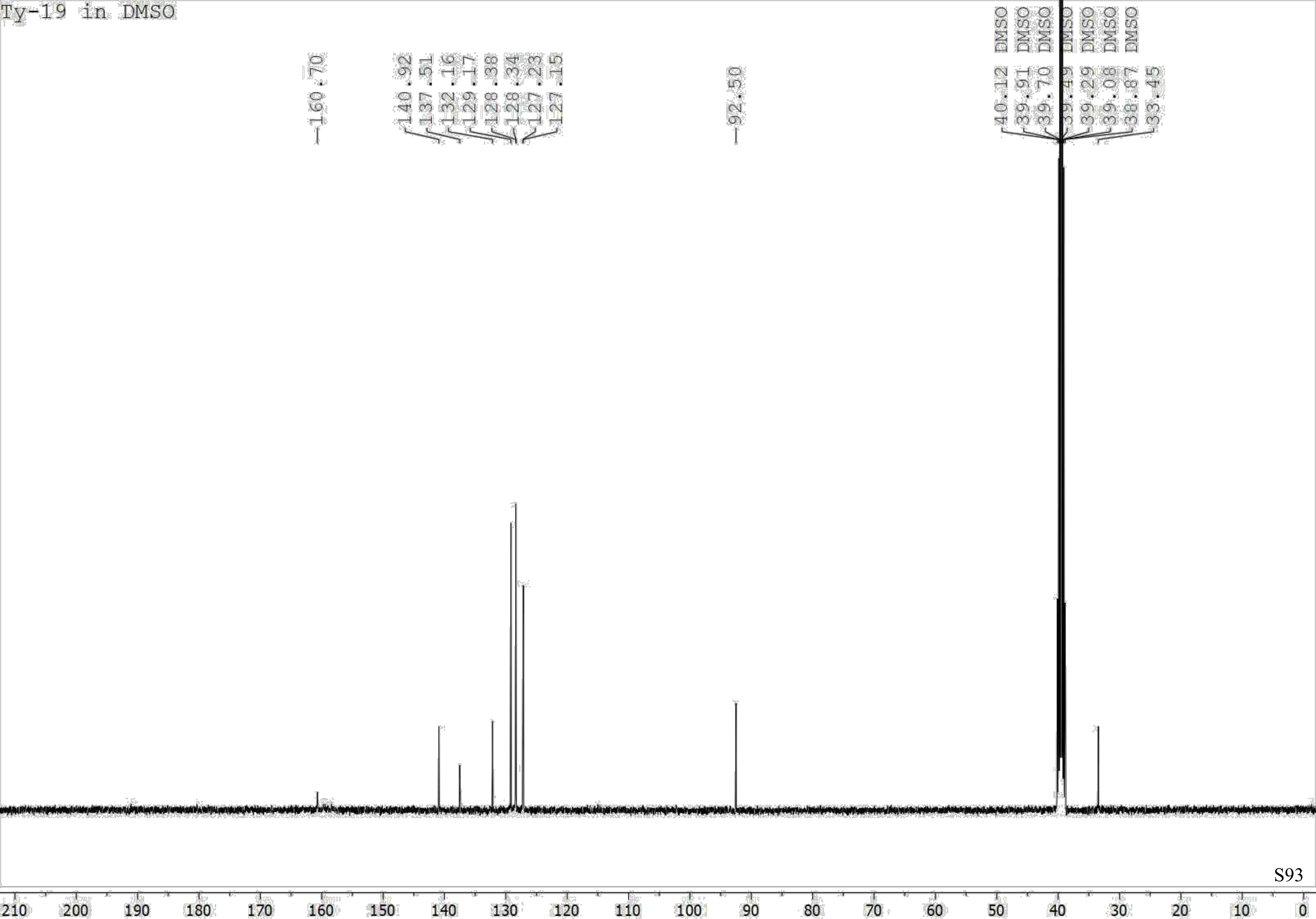
Ty-19 in DMSO
160.70
140.92
137.51
132.16
129.17
128.38
128.34
127.23
127.15
92.50
40.12 DMSO
39.91 DMSO
39.70 DMSO
39.49 DMSO
39.29 DMSO
39.08 DMSO
38.87 DMSO
33.45
210 200 190 180 170 160 150 140 130 120 110 100 90 80 70 60 50 40 30 20 10 0

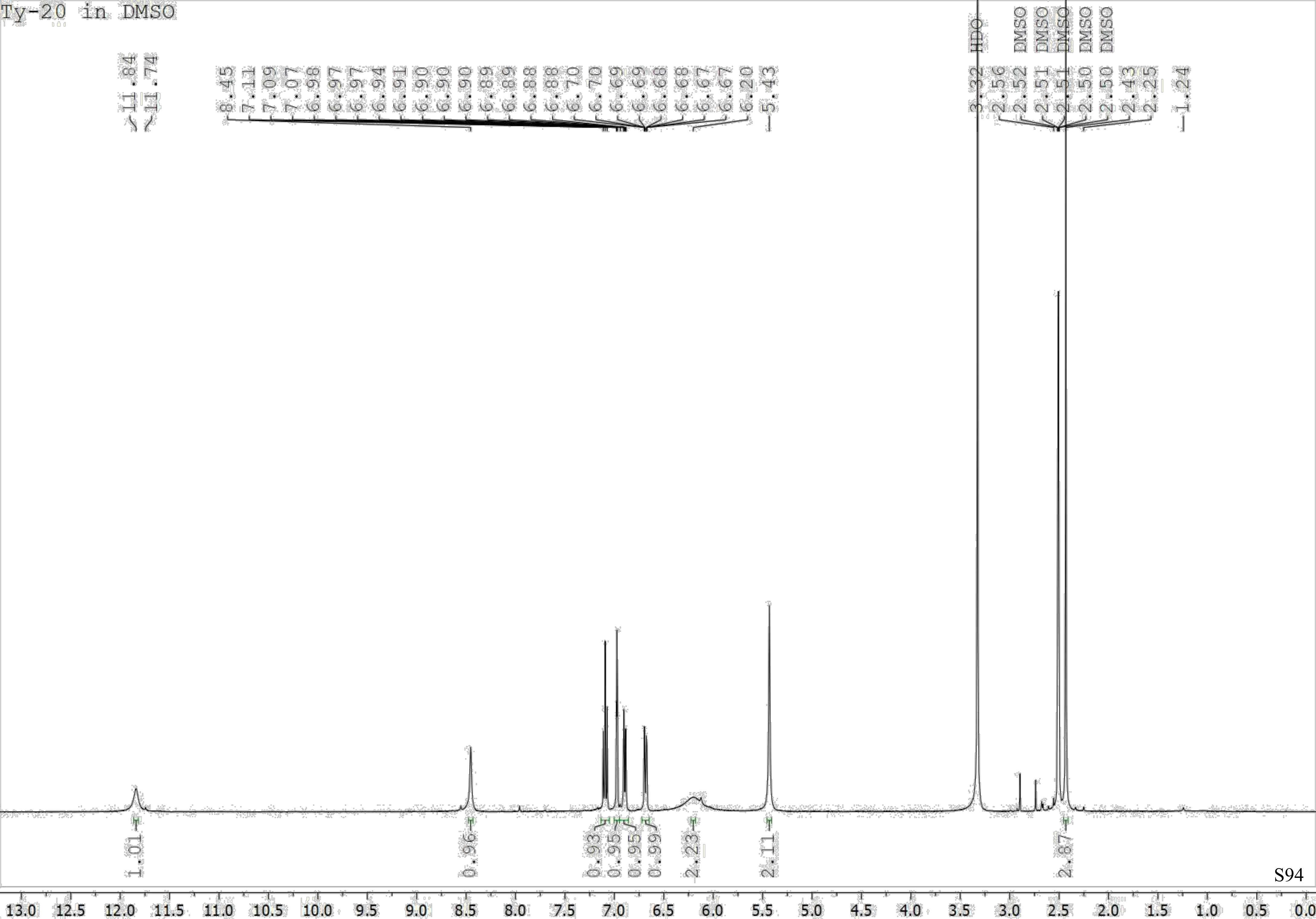

Ty-20 in DMSO
11.84
11.74
8.45
7.11
7.09
7.07
6.98
6.97
6.97
6.94
6.91
6.90
6.90
6.90
6.89
6.89
6.88
6.88
6.70
6.70
6.69
6.69
6.68
6.68
6.67
6.67
6.20
5.43
HDO
DMSO
DMSO
DMSO
DMSO
DMSO
3.32
2.56
2.52
2.51
2.51
2.50
2.50
2.43
2.25
1.24
1.01
0.96
0.93
0.95
0.95
0.99
2.23
2.11
2.87
13.0
12.5
12.0
11.5
11.0
10.5
10.0
9.5
9.0
8.5
8.0
7.5
7.0
6.5
6.0
5.5
5.0
4.5
4.0
3.5
3.0
2.5
2.0
1.5
1.0
0.5
0.0

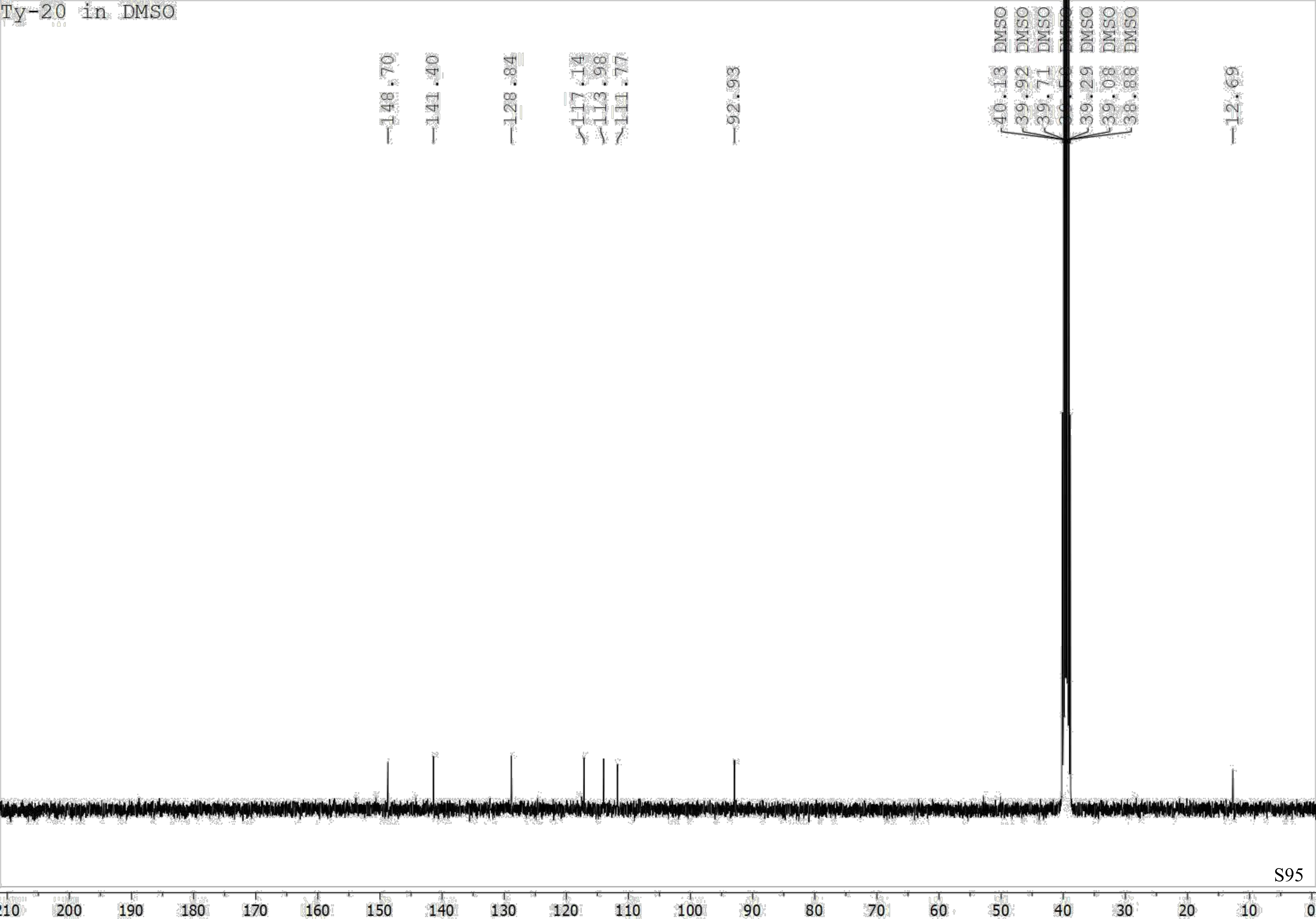
Ty-20 in DMSO
148.70
141.40
128.84
117.14
113.98
111.77
92.93
40.13 DMSO
39.92 DMSO
39.71 DMSO
39.29 DMSO
39.08 DMSO
38.88 DMSO
12.69
210
200
190
180
170
160
150
140
130
120
110
100
90
80
70
60
50
40
30
20
10
0

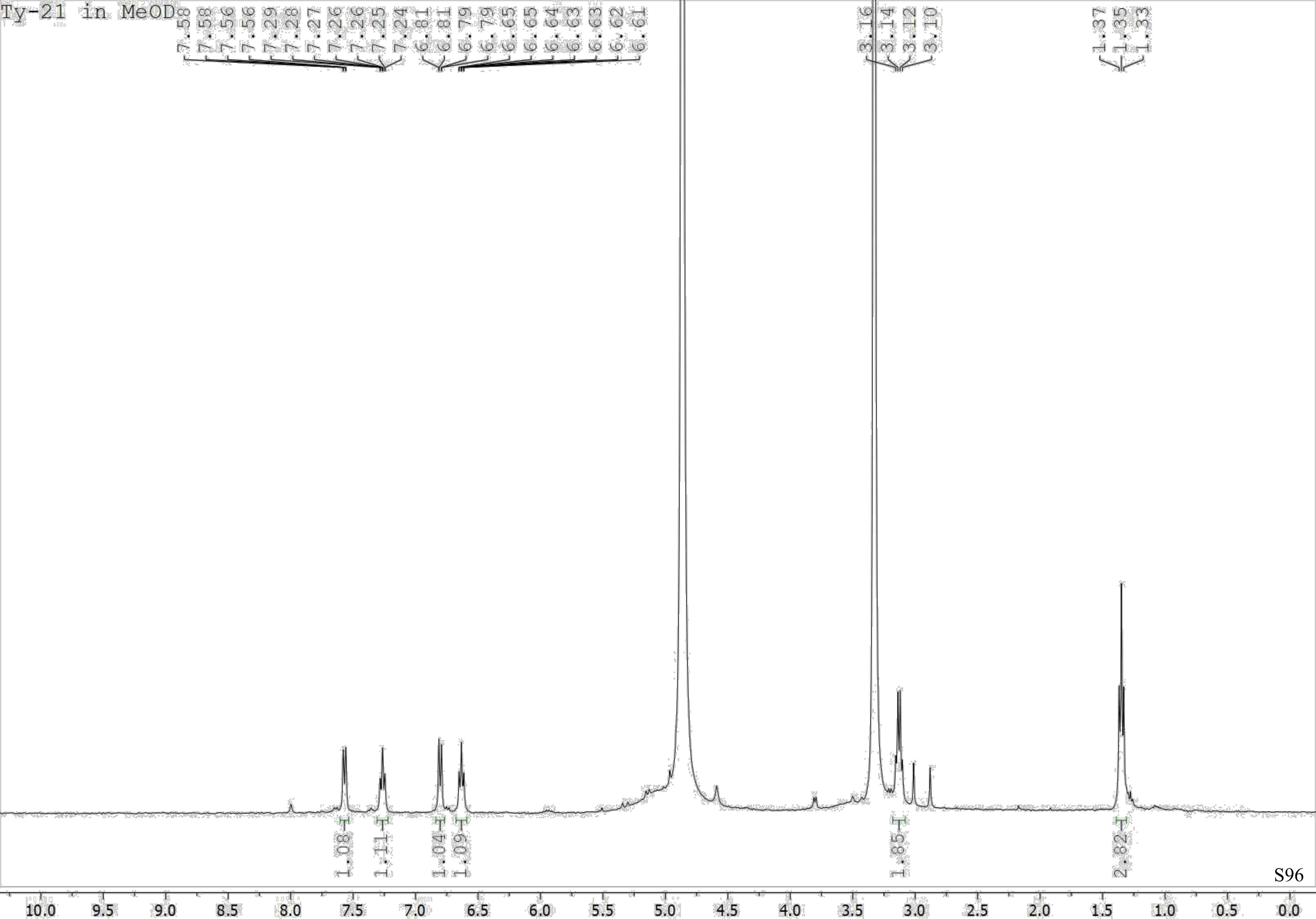
Ty-21 in MeOD
7.58 7.58 7.56 7.56 7.29 7.28 7.27 7.26 7.26 7.25 7.24 6.81 6.81 6.79 6.79 6.65 6.65 6.64 6.63 6.63 6.62 6.61
3.16 3.14 3.12 3.10
1.37 1.35 1.33
1.08 1.11 1.04 1.09 1.85 2.82
10.0 9.5 9.0 8.5 8.0 7.5 7.0 6.5 6.0 5.5 5.0 4.5 4.0 3.5 3.0 2.5 2.0 1.5 1.0 0.5 0.0

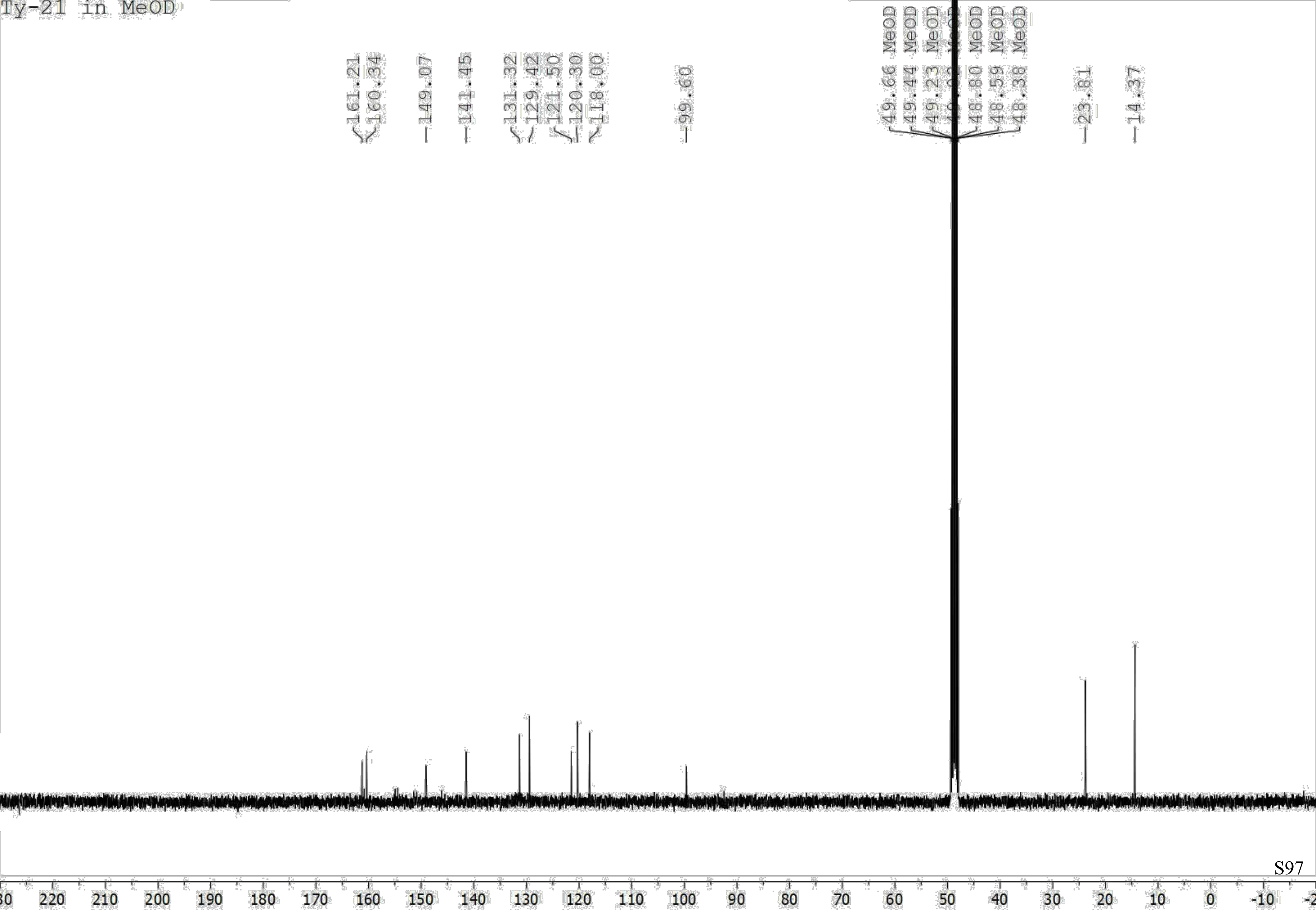

Ty-21 in MeOD
161.21
160.34
149.07
141.45
131.32
129.42
121.50
120.30
118.00
99.60
49.66 MeOD
49.44 MeOD
49.23 MeOD
48.80 MeOD
48.59 MeOD
48.38 MeOD
23.81
14.37
220 210 200 190 180 170 160 150 140 130 120 110 100 90 80 70 60 50 40 30 20 10 0 -10

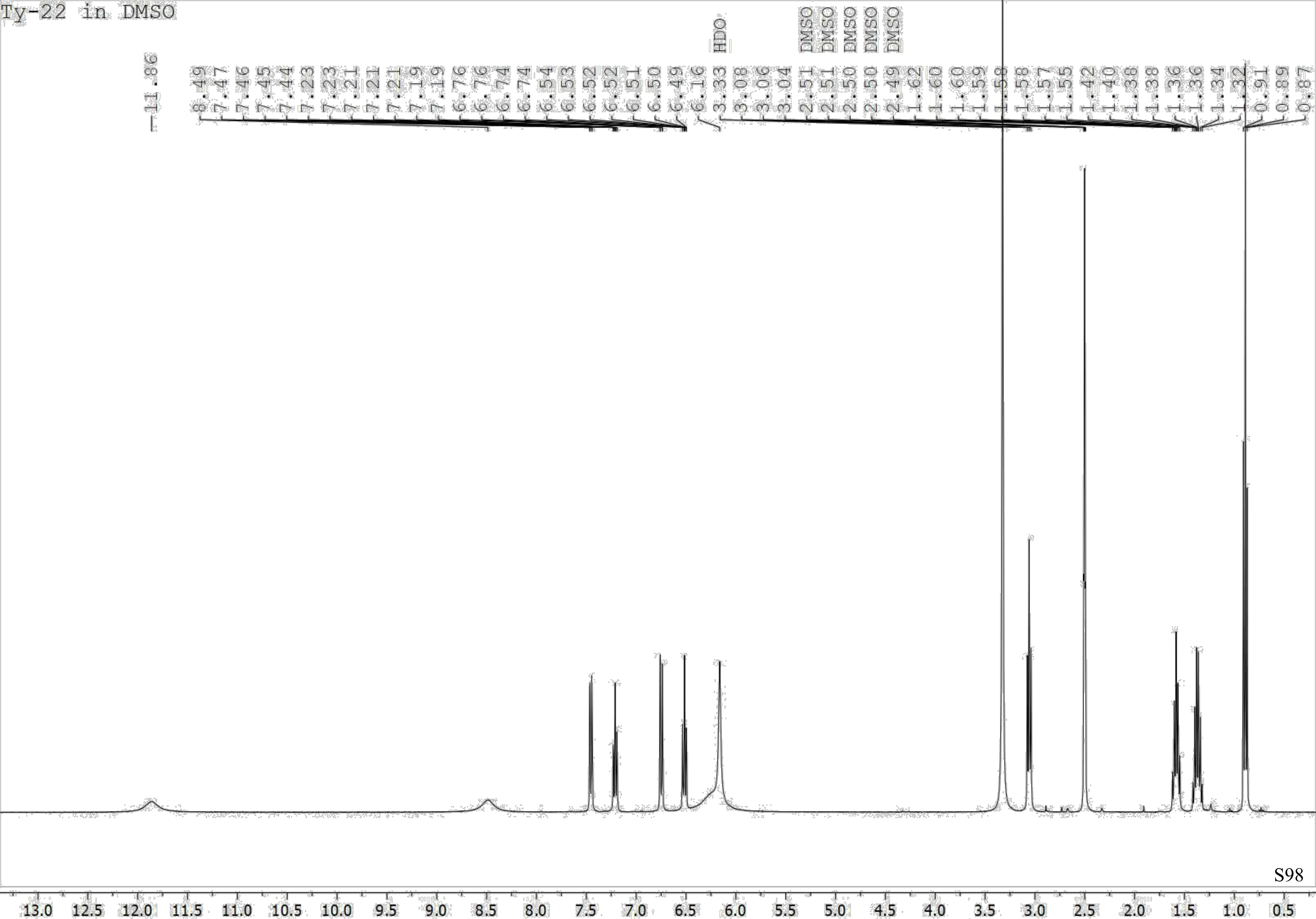
Ty-22 in DMSO
HDO
DMSO
DMSO
DMSO
DMSO
DMSO
11.86
8.49
7.47
7.46
7.45
7.44
7.23
7.23
7.21
7.21
7.21
7.19
7.19
6.76
6.76
6.74
6.74
6.54
6.53
6.52
6.52
6.51
6.50
6.49
6.16
3.33
3.08
3.06
3.04
2.51
2.51
2.50
2.50
2.49
1.62
1.60
1.60
1.59
1.58
1.58
1.57
1.55
1.42
1.40
1.38
1.38
1.36
1.36
1.34
1.32
0.91
0.89
0.87
13.0
12.5
12.0
11.5
11.0
10.5
10.0
9.5
9.0
8.5
8.0
7.5
7.0
6.5
6.0
5.5
5.0
4.5
4.0
3.5
3.0
2.5
2.0
1.5
1.0
0.5

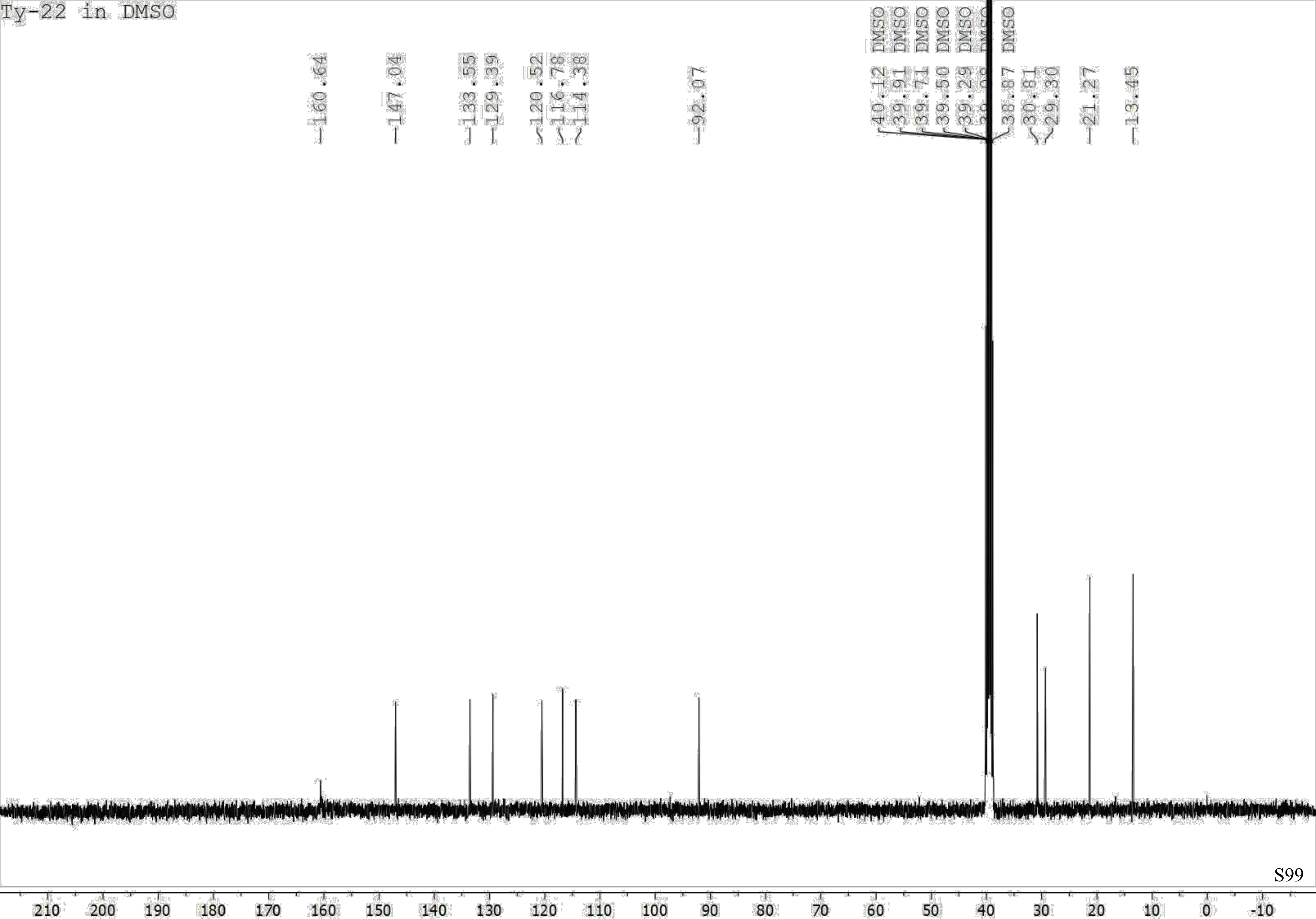
Ty-22 in DMSO
160.64
147.04
133.55
129.39
120.52
116.78
114.38
92.07
40.12 DMSO
39.91 DMSO
39.71 DMSO
39.50 DMSO
39.29 DMSO
38.87 DMSO
30.81
29.30
21.27
13.45
210
200
190
180
170
160
150
140
130
120
110
100
90
80
70
60
50
40
30
20
10
0
-10

Ty-23 in MeOD

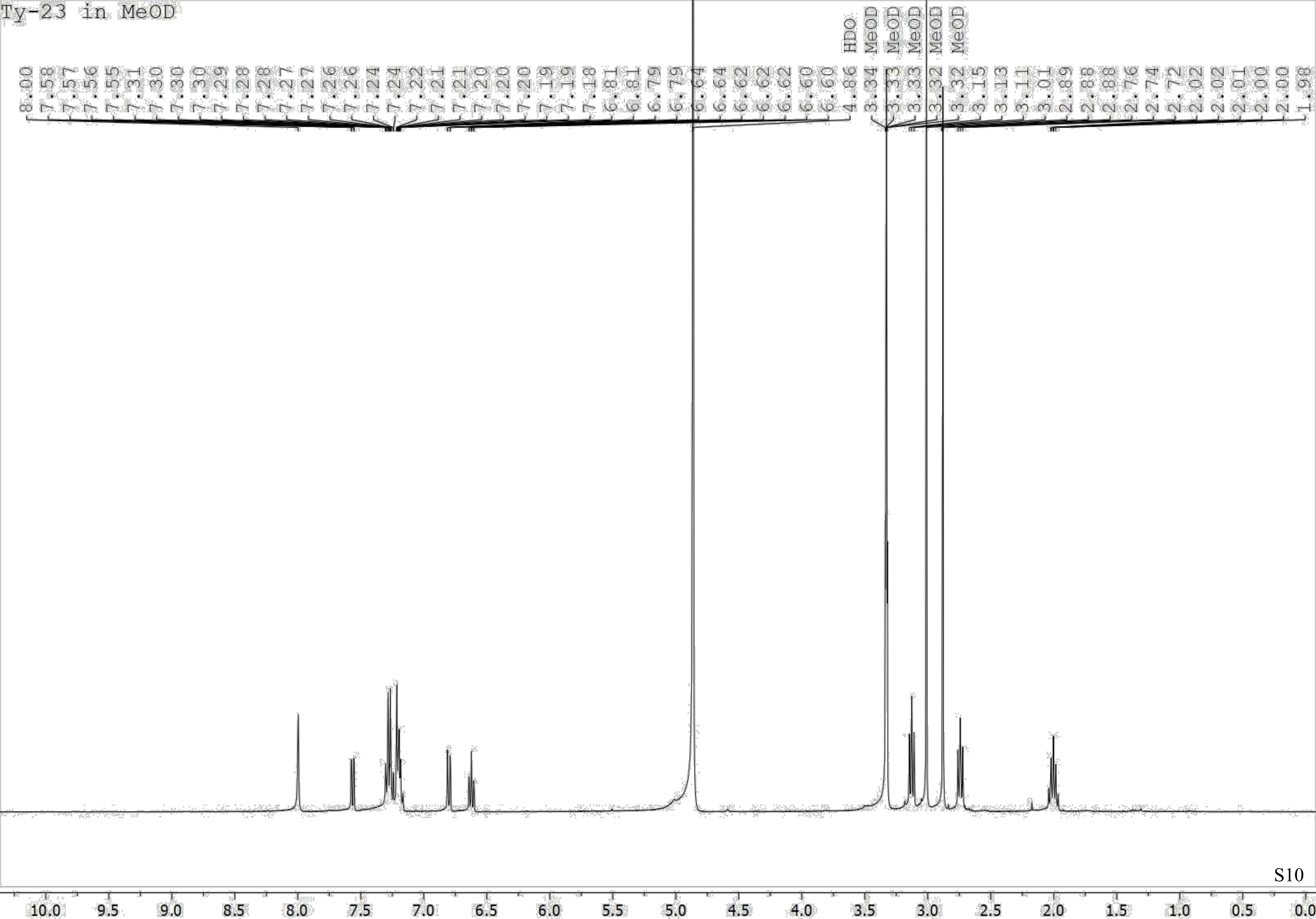
HDO
MeOD
MeOD
MeOD
MeOD
MeOD
8.00 7.58 7.57 7.56 7.55 7.31 7.30 7.30 7.30 7.29 7.28 7.28 7.27 7.27 7.26 7.26 7.24 7.24 7.22 7.21 7.21 7.20 7.20 7.20 7.19 7.19 7.18 6.81 6.81 6.79 6.79 6.64 6.64 6.62 6.62 6.62 6.60 6.60 4.86 3.34 3.33 3.33 3.32 3.32 3.15 3.13 3.11 3.01 2.89 2.88 2.88 2.76 2.74 2.72 2.02 2.02 2.01 2.00 2.00 1.98
10.0 9.5 9.0 8.5 8.0 7.5 7.0 6.5 6.0 5.5 5.0 4.5 4.0 3.5 3.0 2.5 2.0 1.5 1.0 0.5 0.0

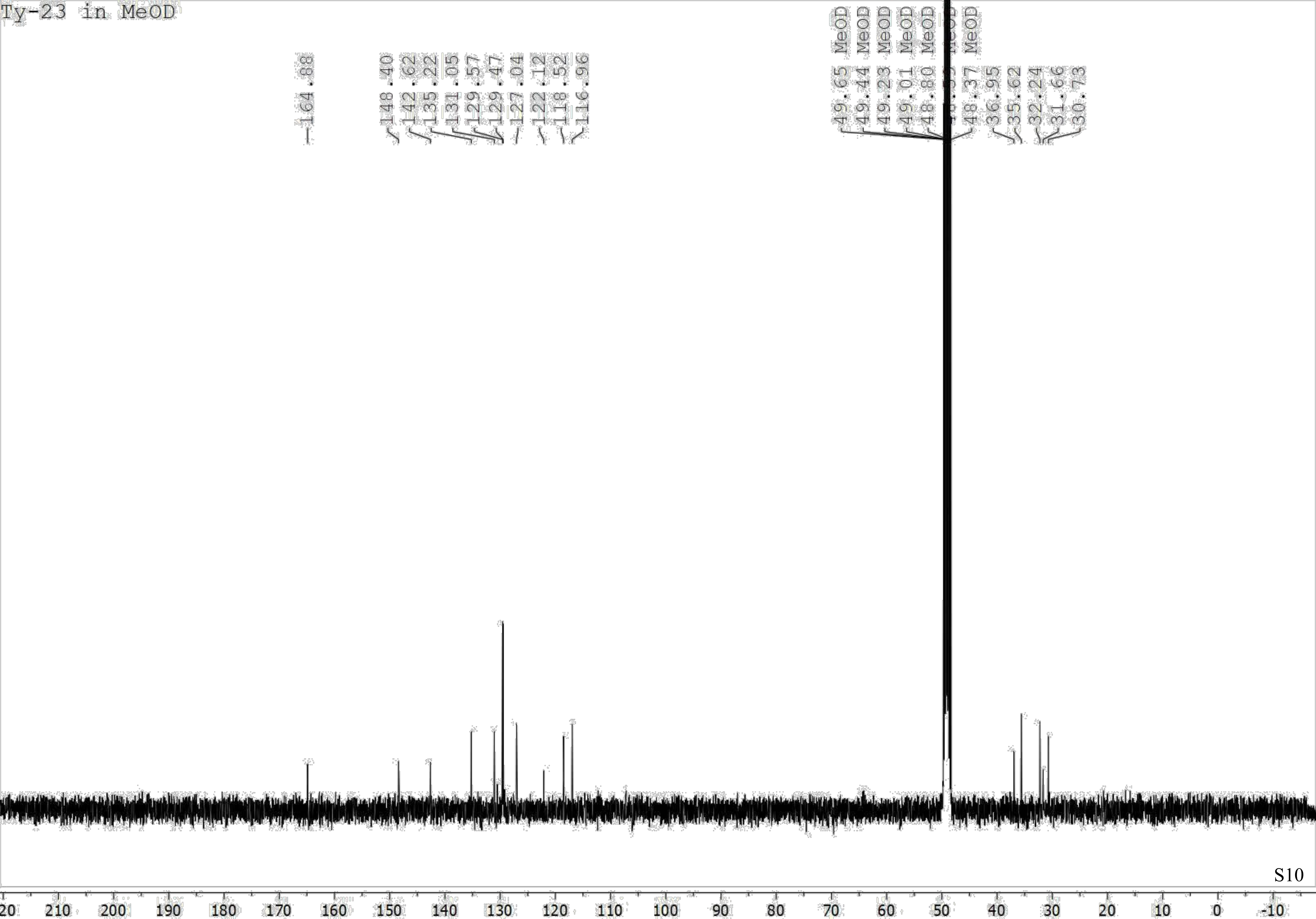
Ty-23 in MeOD
164.88
148.40
142.62
135.22
131.05
129.57
129.47
127.04
122.12
118.52
116.96
49.65 MeOD
49.44 MeOD
49.23 MeOD
49.01 MeOD
48.80 MeOD
48.37 MeOD
36.95
35.62
32.24
31.66
30.73
220
210
200
190
180
170
160
150
140
130
120
110
100
90
80
70
60
50
40
30
20
10
0
-10

Ty-24 in DMSO

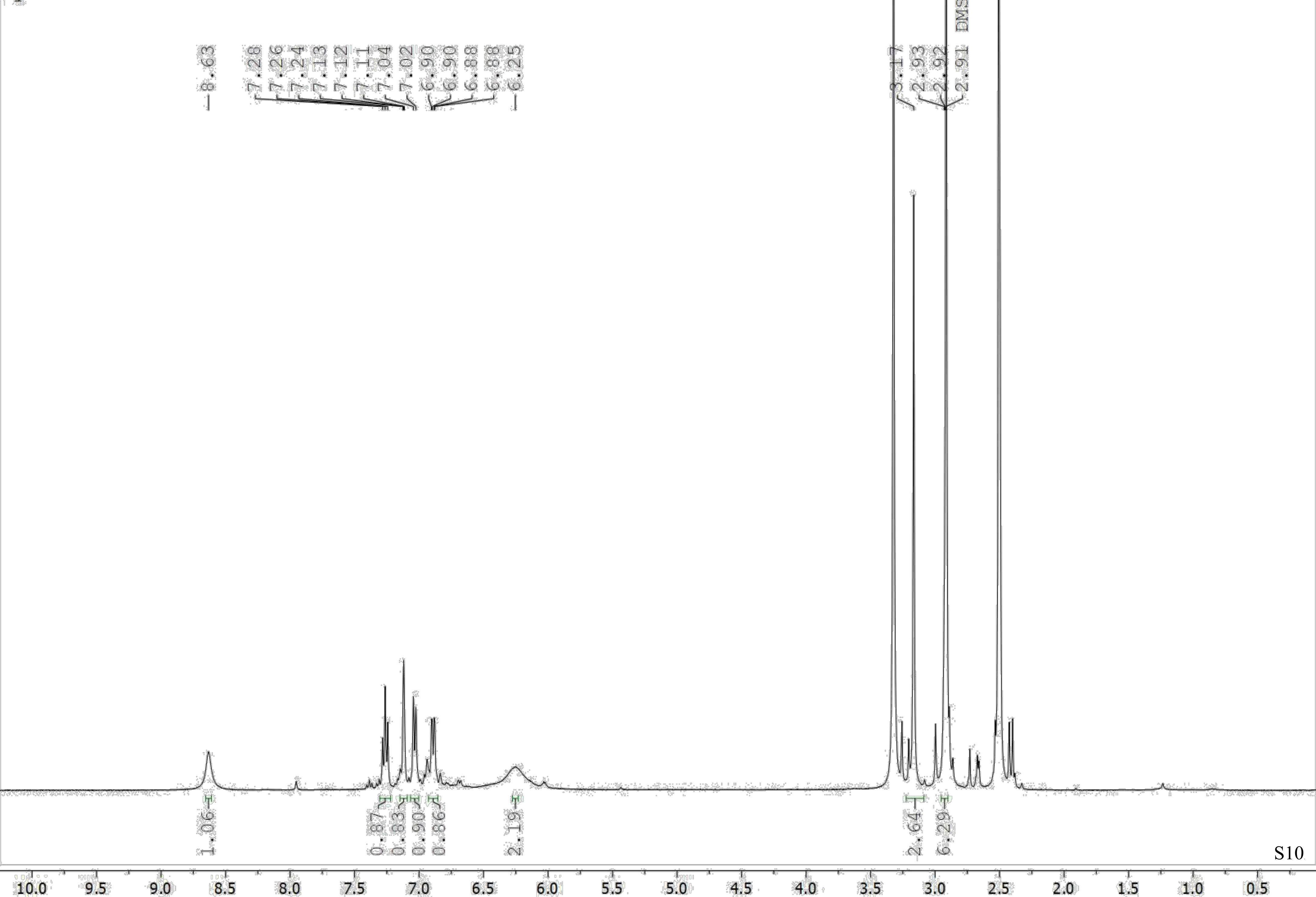
8.63
7.28
7.26
7.24
7.13
7.12
7.11
7.04
7.02
6.90
6.90
6.88
6.88
6.25
3.17
2.93
2.92
2.91
DMSO
1.06
0.87
0.83
0.90
0.86
2.19
2.64
6.29
10.0 9.5 9.0 8.5 8.0 7.5 7.0 6.5 6.0 5.5 5.0 4.5 4.0 3.5 3.0 2.5 2.0 1.5 1.0 0.5

Ty-24 in DMSO

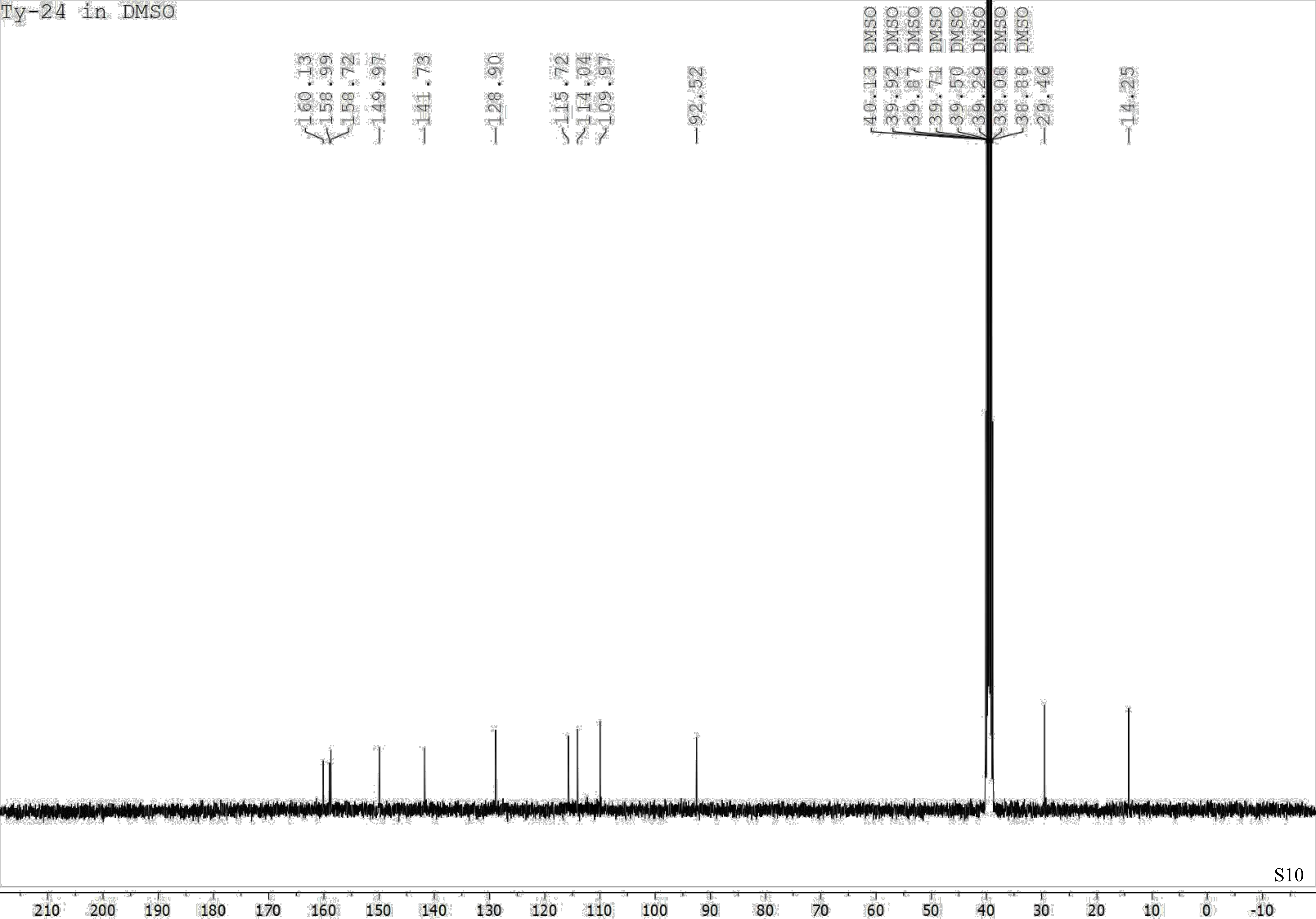
160.13
158.99
158.72
149.97
141.73
128.90
115.72
114.04
109.97
92.52
40.13 DMSO
39.92 DMSO
39.87 DMSO
39.71 DMSO
39.50 DMSO
39.29 DMSO
39.08 DMSO
38.88 DMSO
29.46
14.25
210
200
190
180
170
160
150
140
130
120
110
100
90
80
70
60
50
40
30
20
10
0
-10

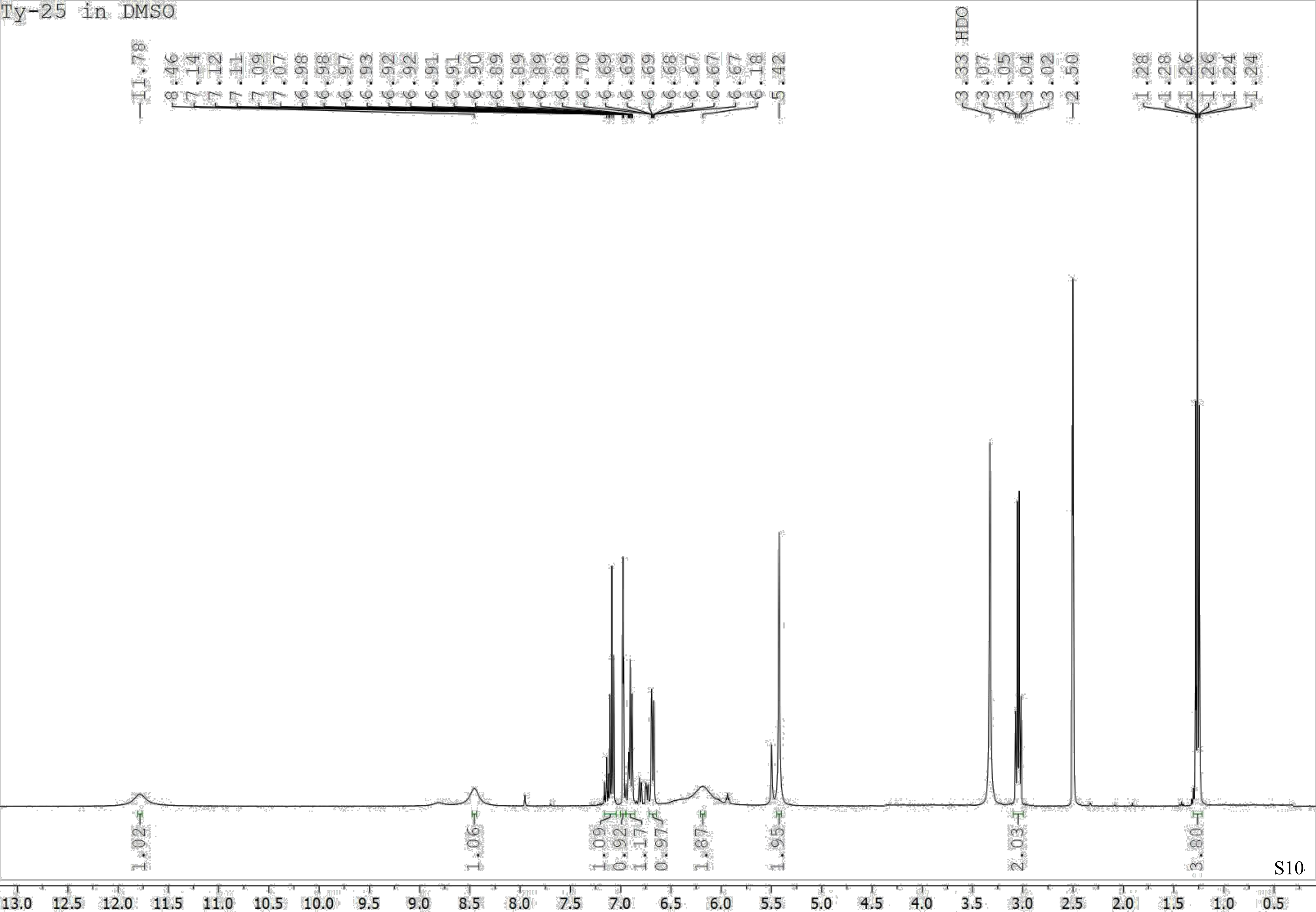
Ty-25 in DMSO
HDO
11.78
8.46
7.14
7.12
7.11
7.09
7.07
6.98
6.98
6.97
6.93
6.92
6.92
6.91
6.91
6.90
6.89
6.89
6.89
6.88
6.70
6.69
6.69
6.69
6.68
6.67
6.67
6.67
6.18
5.42
3.33
3.07
3.05
3.04
3.02
2.50
1.28
1.28
1.26
1.26
1.24
1.24
1.02
1.06
1.09
0.92
1.17
0.97
1.87
1.95
2.03
3.80
13.0
12.5
12.0
11.5
11.0
10.5
10.0
9.5
9.0
8.5
8.0
7.5
7.0
6.5
6.0
5.5
5.0
4.5
4.0
3.5
3.0
2.5
2.0
1.5
1.0
0.5

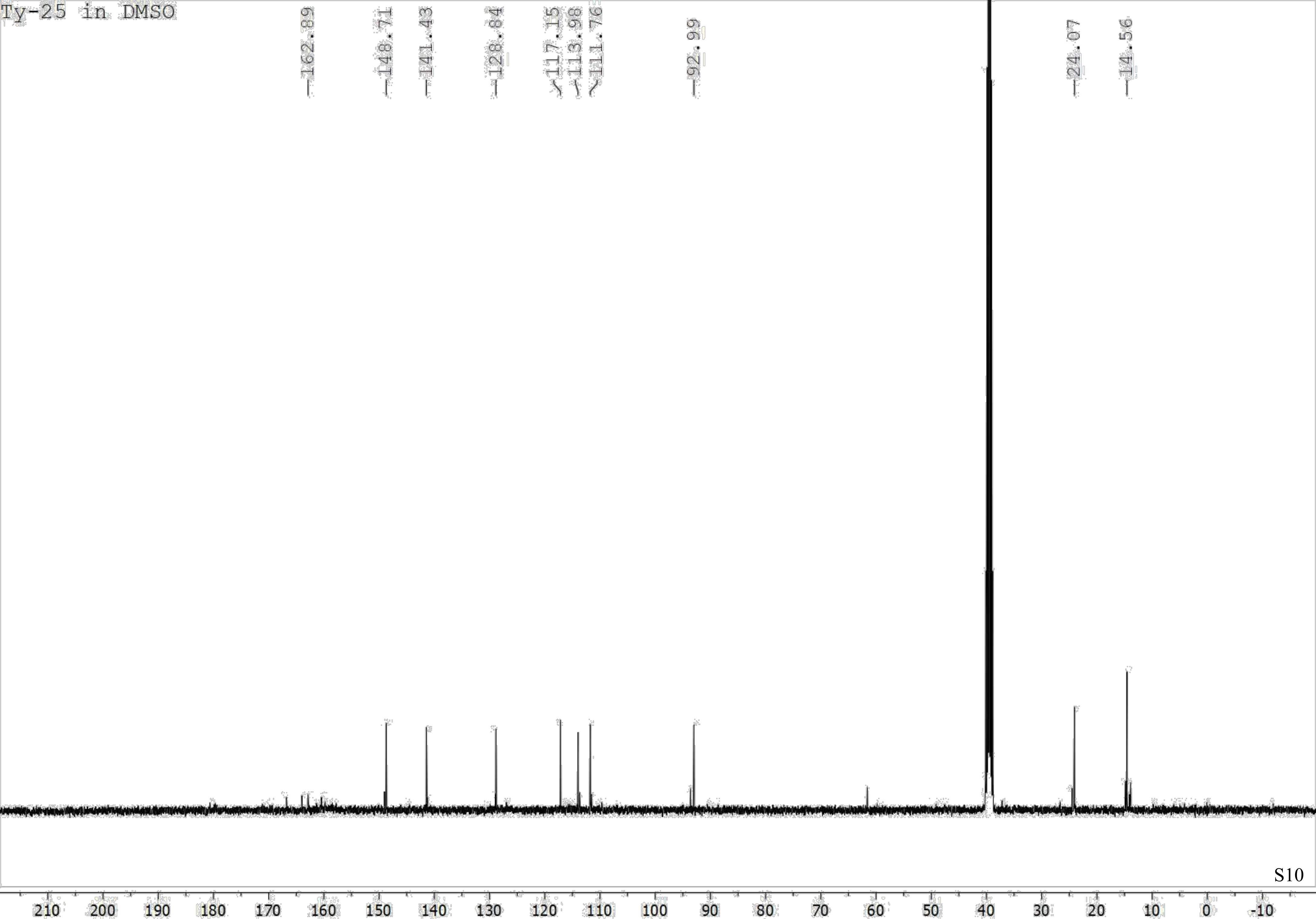
Ty-25 in DMSO
162.89
148.71
141.43
128.84
117.15
113.98
111.76
92.99
24.07
14.56
210 200 190 180 170 160 150 140 130 120 110 100 90 80 70 60 50 40 30 20 10 0 -10

Ty-26 in DMSO

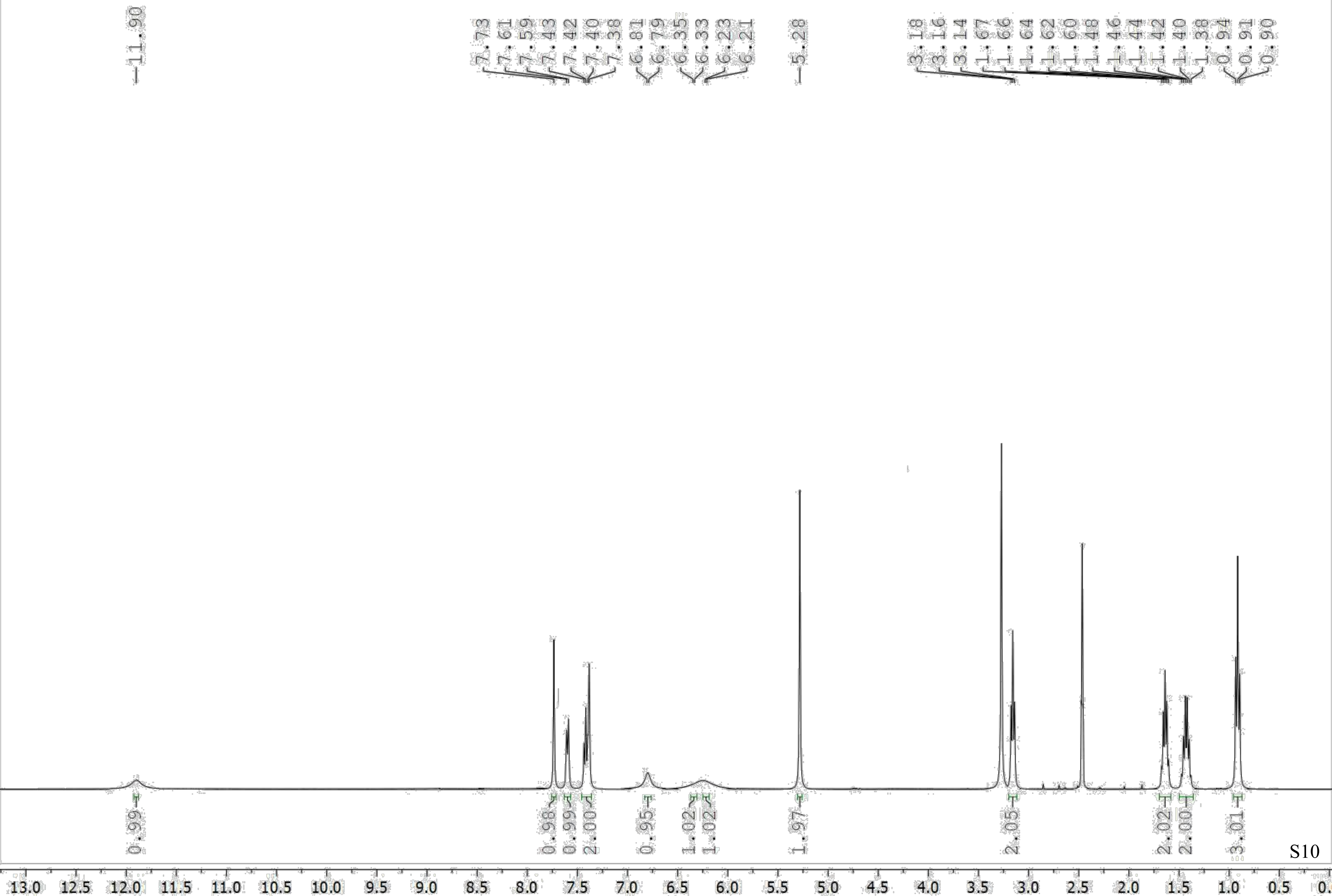
11.90
7.73
7.61
7.59
7.43
7.42
7.40
7.38
6.81
6.79
6.35
6.33
6.23
6.21
5.28
3.18
3.16
3.14
1.67
1.66
1.64
1.62
1.60
1.48
1.46
1.44
1.42
1.40
1.38
0.94
0.91
0.90
0.99
0.98
0.99
2.00
0.95
1.02
1.02
1.97
2.05
2.02
2.00
3.01
13.0
12.5
12.0
11.5
11.0
10.5
10.0
9.5
9.0
8.5
8.0
7.5
7.0
6.5
6.0
5.5
5.0
4.5
4.0
3.5
3.0
2.5
2.0
1.5
1.0
0.5

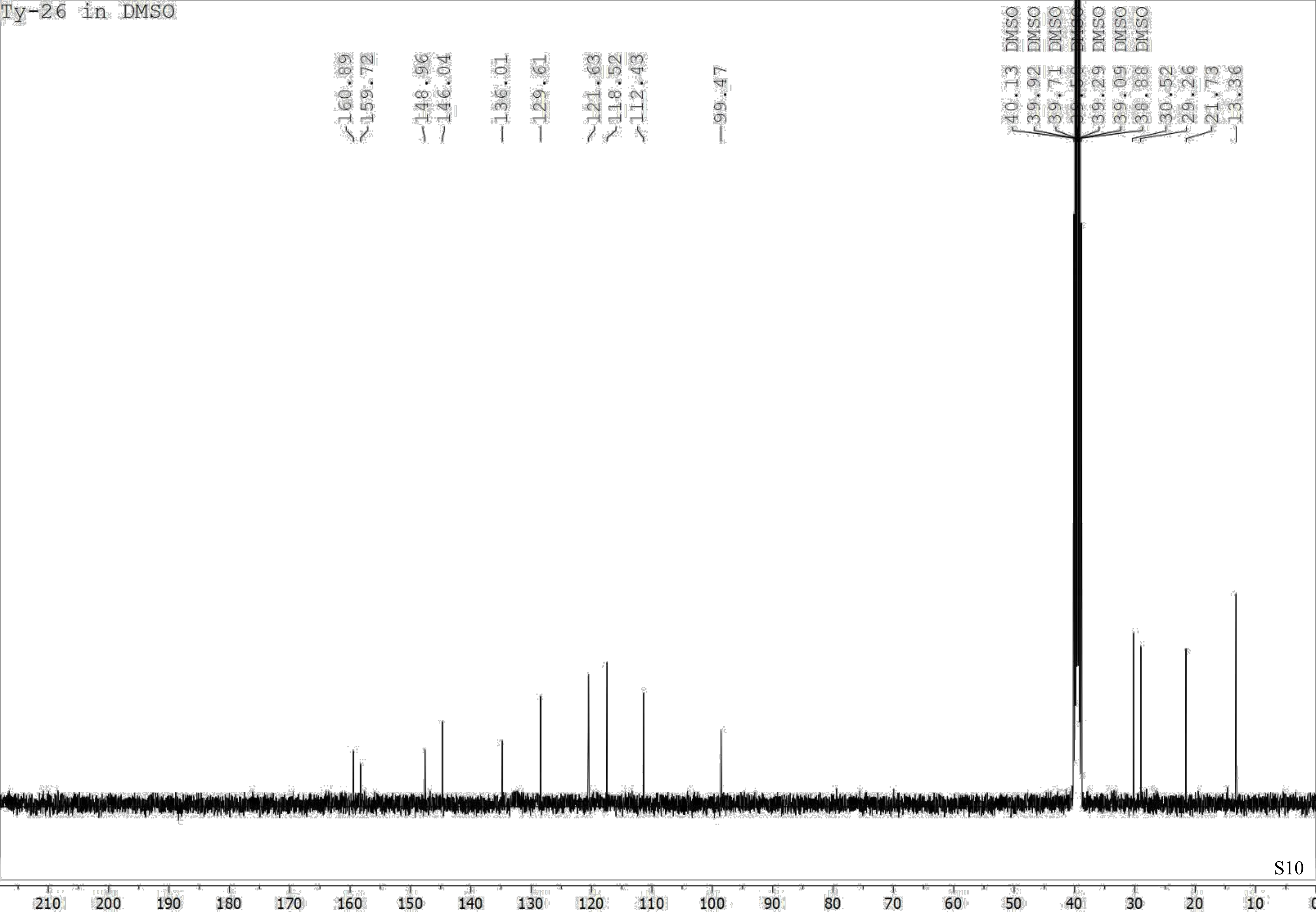
Ty-26 in DMSO
160.89
159.72
148.96
146.04
136.01
129.61
121.63
118.52
112.43
99.47
40.13 DMSO
39.92 DMSO
39.71 DMSO
39.29 DMSO
39.09 DMSO
38.88 DMSO
30.52
29.26
21.73
13.36
210 200 190 180 170 160 150 140 130 120 110 100 90 80 70 60 50 40 30 20 10

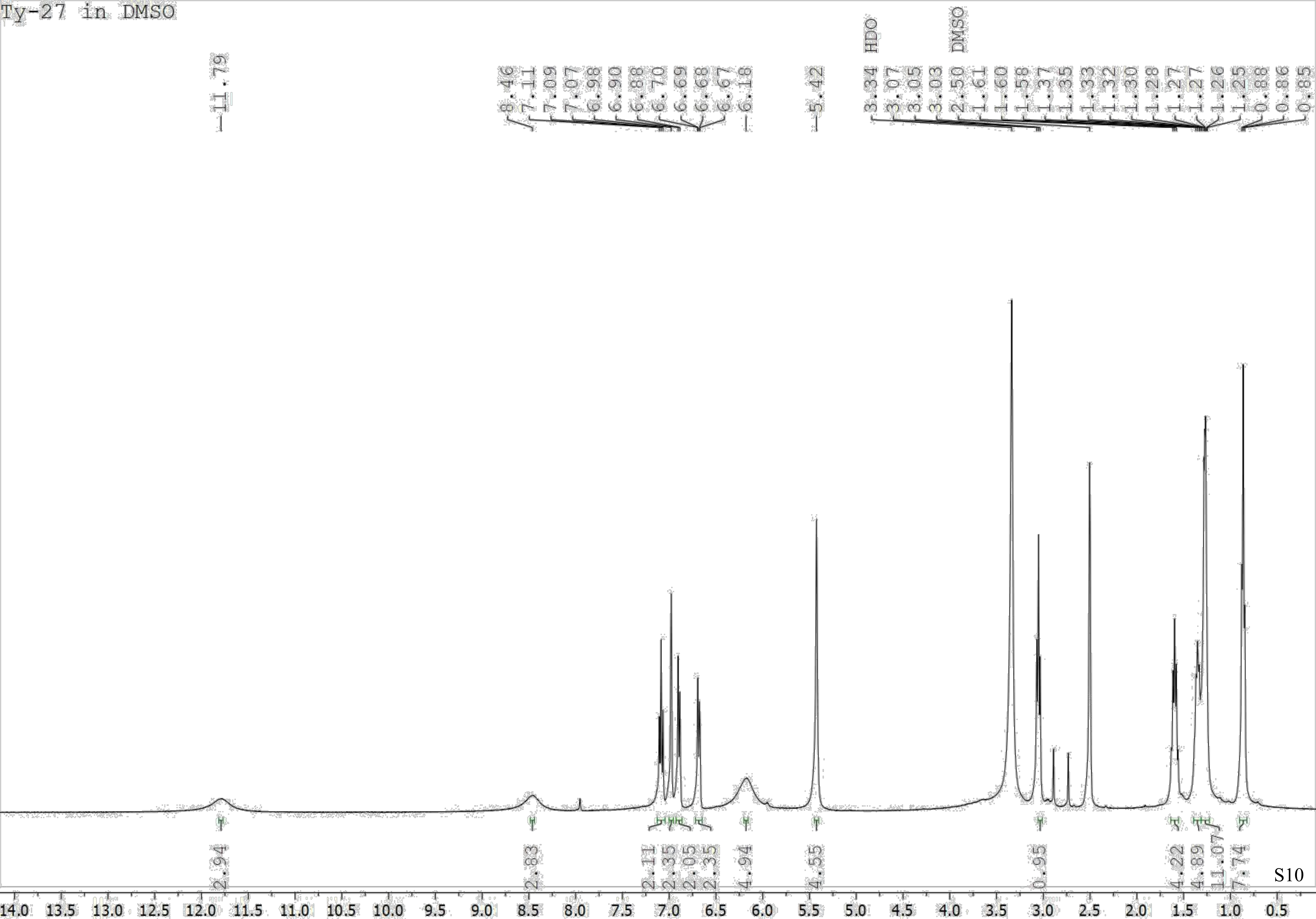

Ty-27 in DMSO
11.79
8.46
7.11
7.09
7.07
6.98
6.90
6.88
6.70
6.69
6.68
6.67
6.18
5.42
HDO
3.34
3.07
3.05
3.03
DMSO
2.50
1.61
1.60
1.58
1.37
1.35
1.33
1.32
1.30
1.28
1.27
1.27
1.26
1.25
0.88
0.86
0.85
2.94
2.83
2.11
2.35
2.05
2.35
4.94
4.55
0.95
4.22
4.89
11.07
7.74

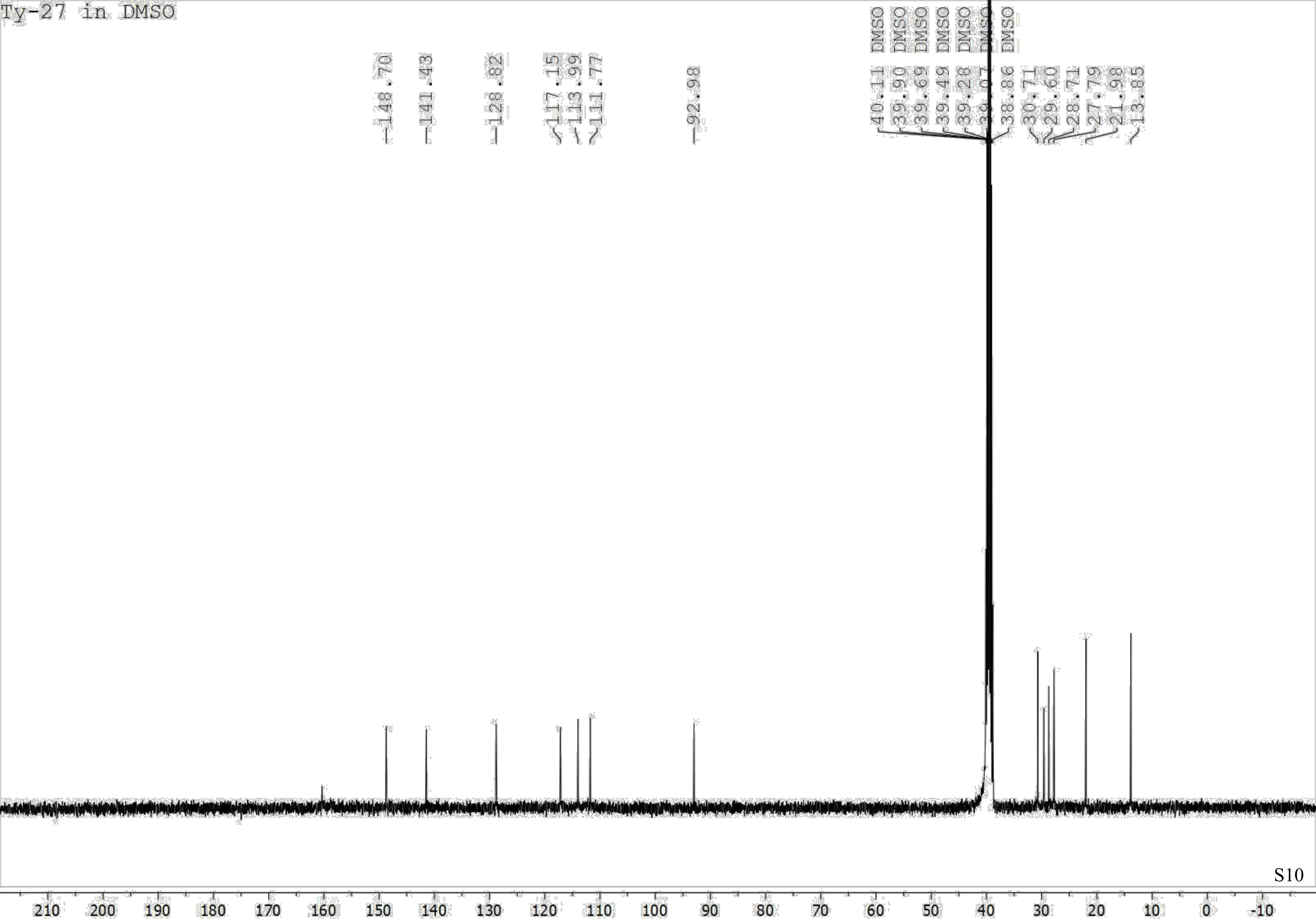
Ty-27 in DMSO
148.70
141.43
128.82
117.15
113.99
111.77
92.98
40.11 DMSO
39.90 DMSO
39.69 DMSO
39.49 DMSO
39.28 DMSO
39.07 DMSO
38.86 DMSO
30.71
29.60
28.71
27.79
21.98
13.85
210
200
190
180
170
160
150
140
130
120
110
100
90
80
70
60
50
40
30
20
10
0
-10

Ty-28 in DMSO

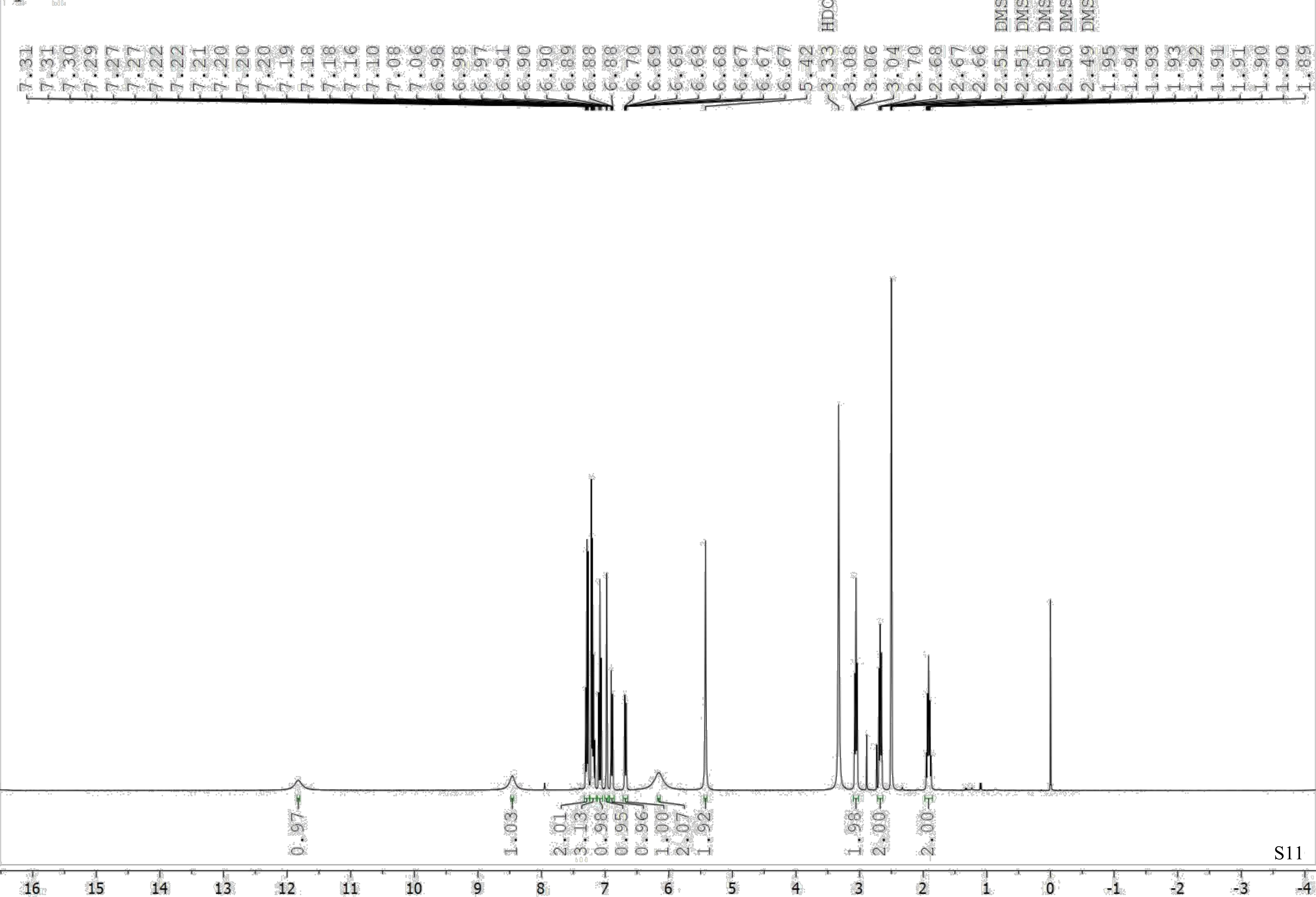
HDO
DMSO
DMSO
DMSO
DMSO
DMSO
7.31 7.31 7.30 7.29 7.27 7.27 7.22 7.22 7.21 7.20 7.20 7.20 7.19 7.18 7.18 7.16 7.10 7.08 7.06 6.98 6.98 6.97 6.91 6.90 6.90 6.89 6.88 6.88 6.70 6.69 6.69 6.69 6.68 6.67 6.67 6.67 5.42 3.33 3.08 3.06 3.04 2.70 2.68 2.67 2.66 2.51 2.51 2.50 2.50 2.49 1.95 1.94 1.93 1.93 1.92 1.91 1.91 1.90 1.90 1.89
0.97 1.03 2.01 3.13 0.98 0.95 0.96 1.00 2.07 1.92 1.98 2.00 2.00
16 15 14 13 12 11 10 9 8 7 6 5 4 3 2 1 0 -1 -2 -3 -4

Ty-28 in DMSO

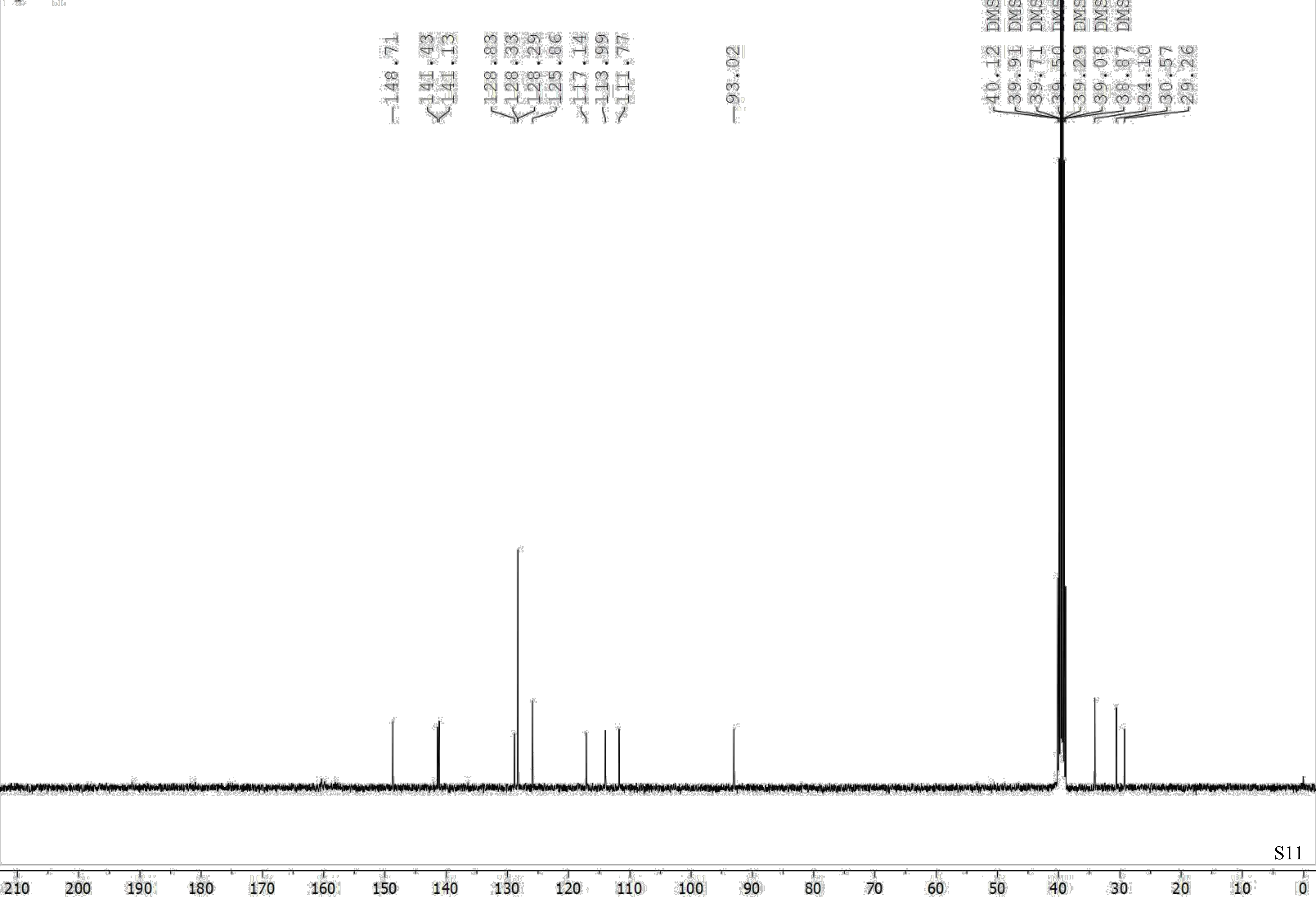
148.71
141.43
141.13
128.83
128.33
128.29
125.86
117.14
113.99
111.77
93.02
40.12 DMSO
39.91 DMSO
39.71 DMSO
39.50 DMSO
39.29 DMSO
39.08 DMSO
38.87 DMSO
34.10
30.57
29.26
210
200
190
180
170
160
150
140
130
120
110
100
90
80
70
60
50
40
30
20
10
0

Ty-29 in DMSO

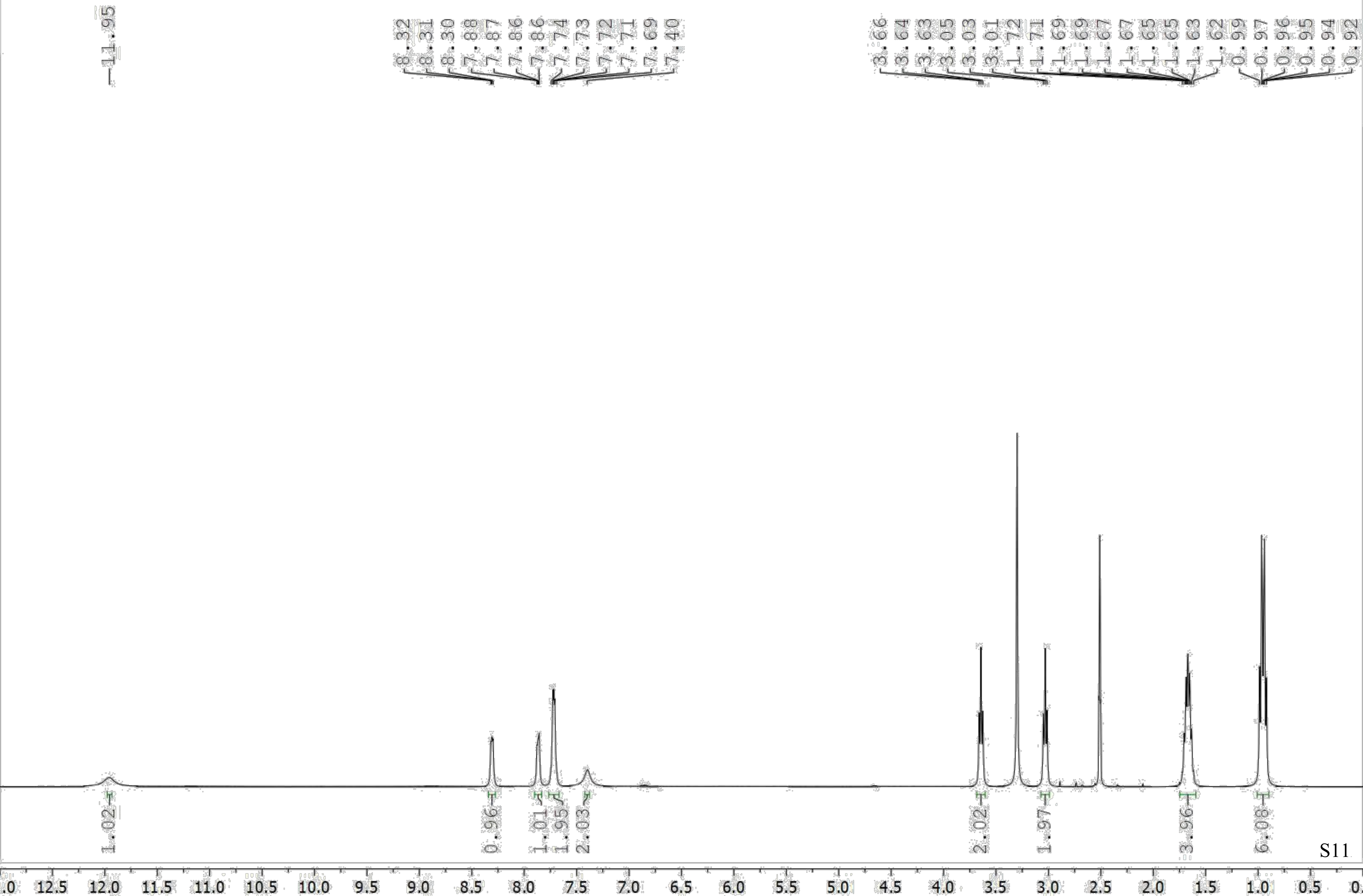

11.95
8.32
8.31
8.30
7.88
7.87
7.86
7.86
7.74
7.73
7.72
7.71
7.69
7.40
3.66
3.64
3.63
3.05
3.03
3.01
1.72
1.71
1.69
1.69
1.67
1.67
1.65
1.65
1.63
1.62
0.99
0.97
0.96
0.95
0.94
0.92
1.02
0.96
1.01
1.95
2.03
2.02
1.97
3.96
6.08
12.5
12.0
11.5
11.0
10.5
10.0
9.5
9.0
8.5
8.0
7.5
7.0
6.5
6.0
5.5
5.0
4.5
4.0
3.5
3.0
2.5
2.0
1.5
1.0
0.5

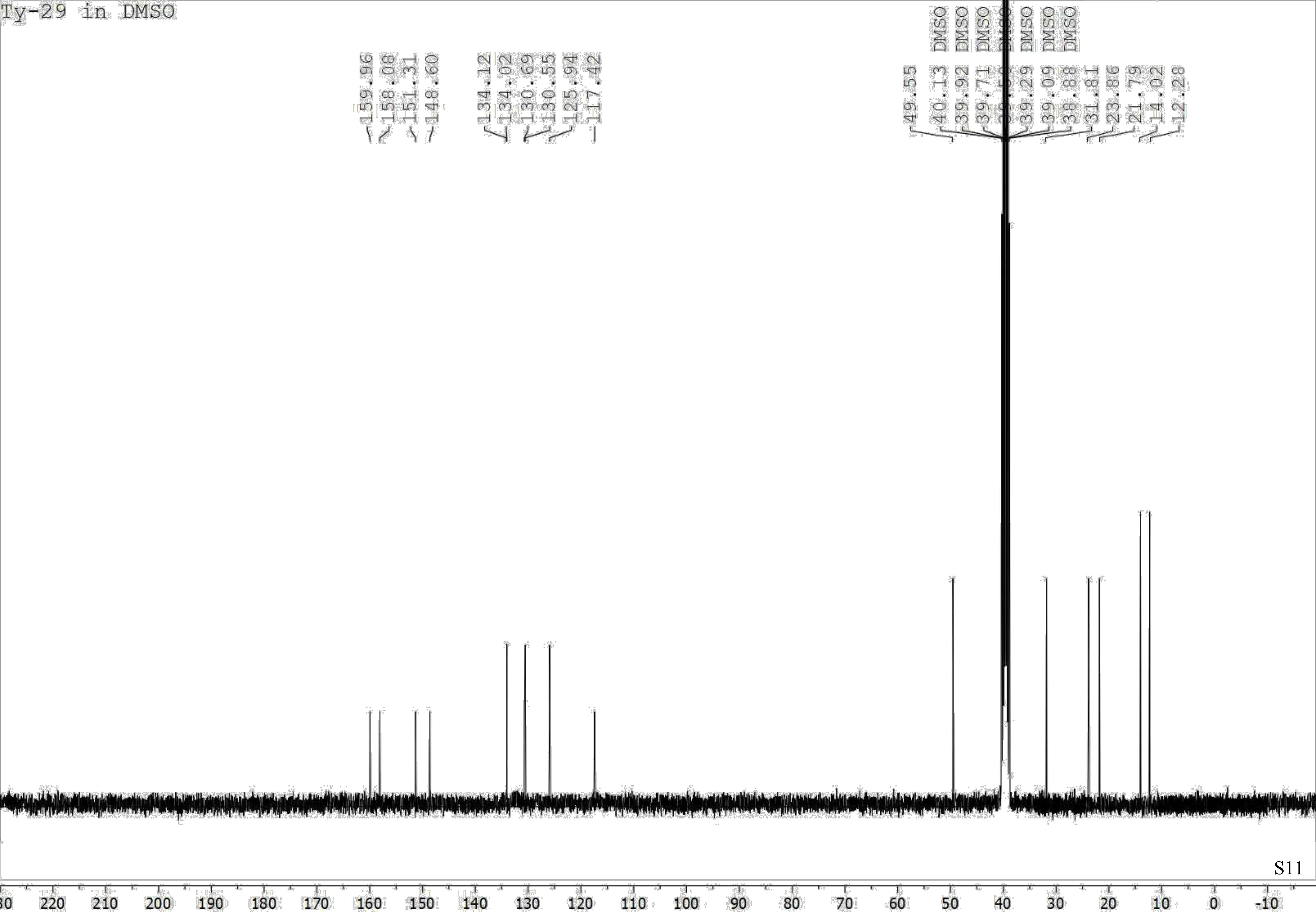

Ty-29 in DMSO
159.96
158.08
151.31
148.60
134.12
134.02
130.69
130.55
125.94
117.42
49.55
40.13 DMSO
39.92 DMSO
39.71 DMSO
39.29 DMSO
39.09 DMSO
38.88 DMSO
31.81
23.86
21.79
14.02
12.28
220
210
200
190
180
170
160
150
140
130
120
110
100
90
80
70
60
50
40
30
20
10
0
-10

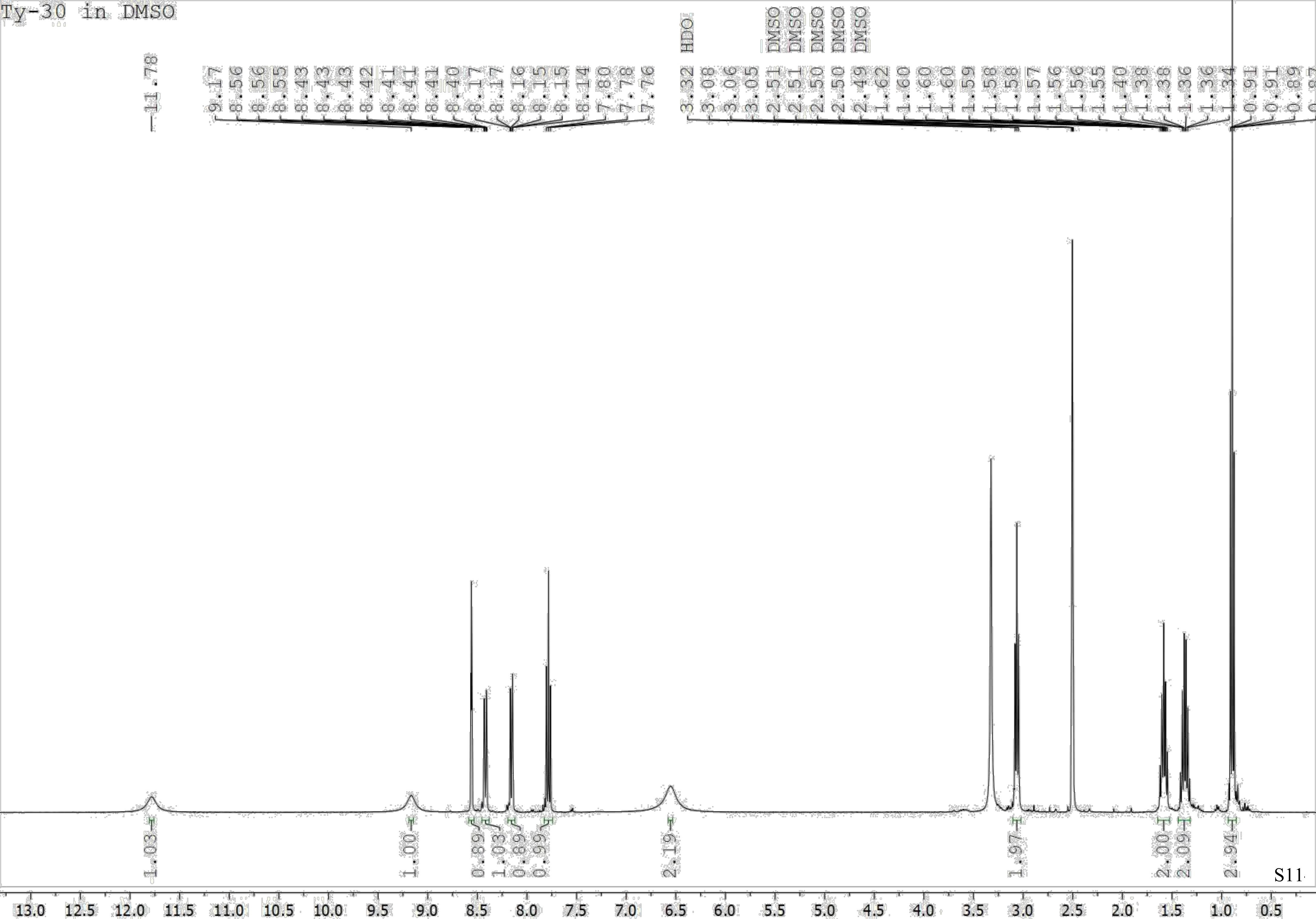
Ty-30 in DMSO
HDO
DMSO
DMSO
DMSO
DMSO
DMSO
11.78
9.17
8.56
8.56
8.55
8.43
8.43
8.43
8.42
8.41
8.41
8.41
8.40
8.17
8.17
8.16
8.15
8.15
8.14
7.80
7.78
7.76
3.32
3.08
3.06
3.05
2.51
2.51
2.50
2.50
2.49
1.62
1.60
1.60
1.60
1.59
1.58
1.58
1.57
1.56
1.56
1.55
1.40
1.38
1.38
1.36
1.36
1.34
0.91
0.91
0.89
1.03
1.00
0.89
1.03
0.89
0.99
2.19
1.97
2.00
2.09
2.94
13.0
12.5
12.0
11.5
11.0
10.5
10.0
9.5
9.0
8.5
8.0
7.5
7.0
6.5
6.0
5.5
5.0
4.5
4.0
3.5
3.0
2.5
2.0
1.5
1.0
0.5

Ty-30 in DMSO

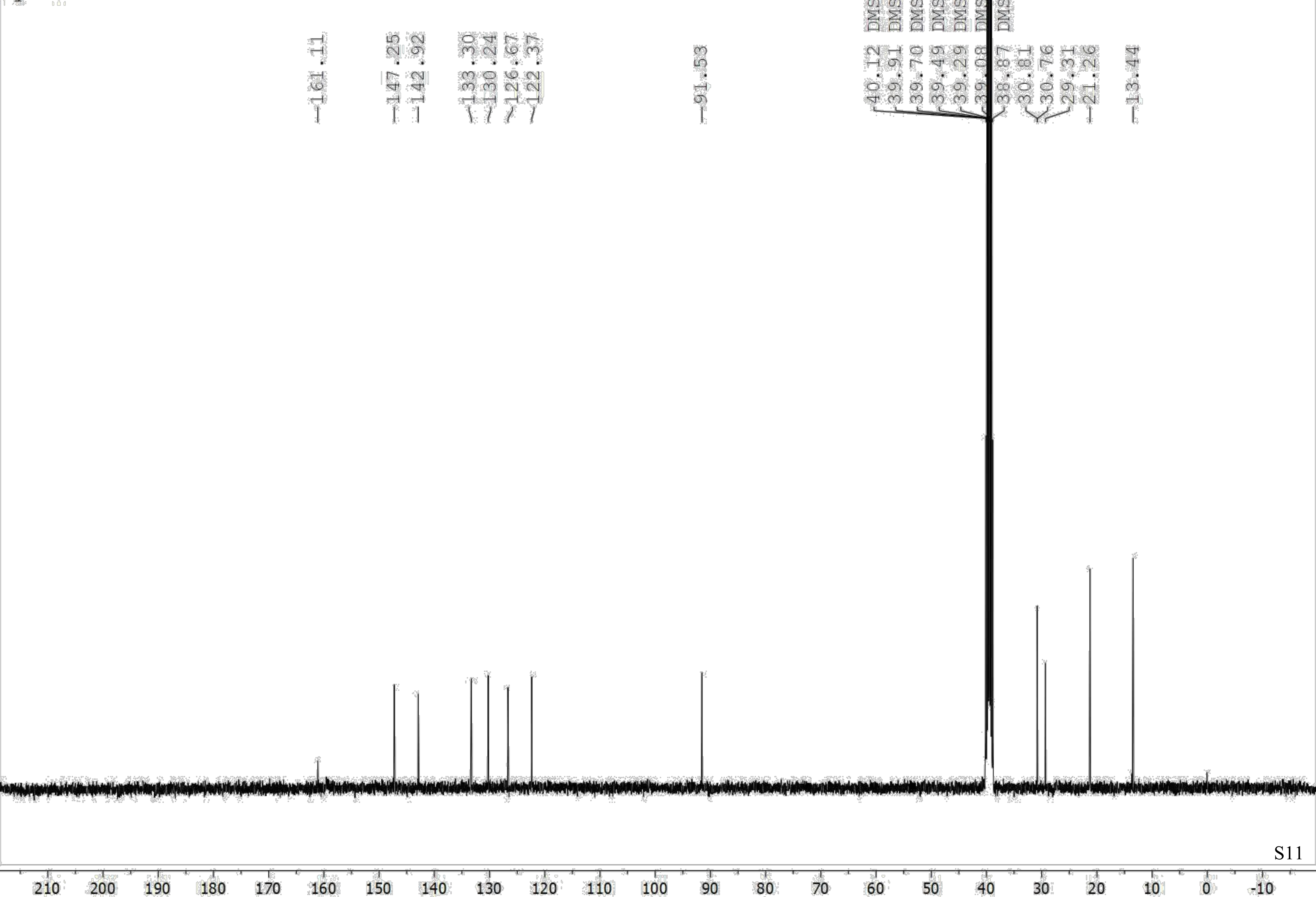
161.11
147.25
142.92
133.30
130.24
126.67
122.37
91.53
40.12 DMSO
39.91 DMSO
39.70 DMSO
39.49 DMSO
39.29 DMSO
39.08 DMSO
38.87 DMSO
30.81
30.76
29.31
21.26
13.44
210 200 190 180 170 160 150 140 130 120 110 100 90 80 70 60 50 40 30 20 10 0 -10

Ty-31 in MeOD

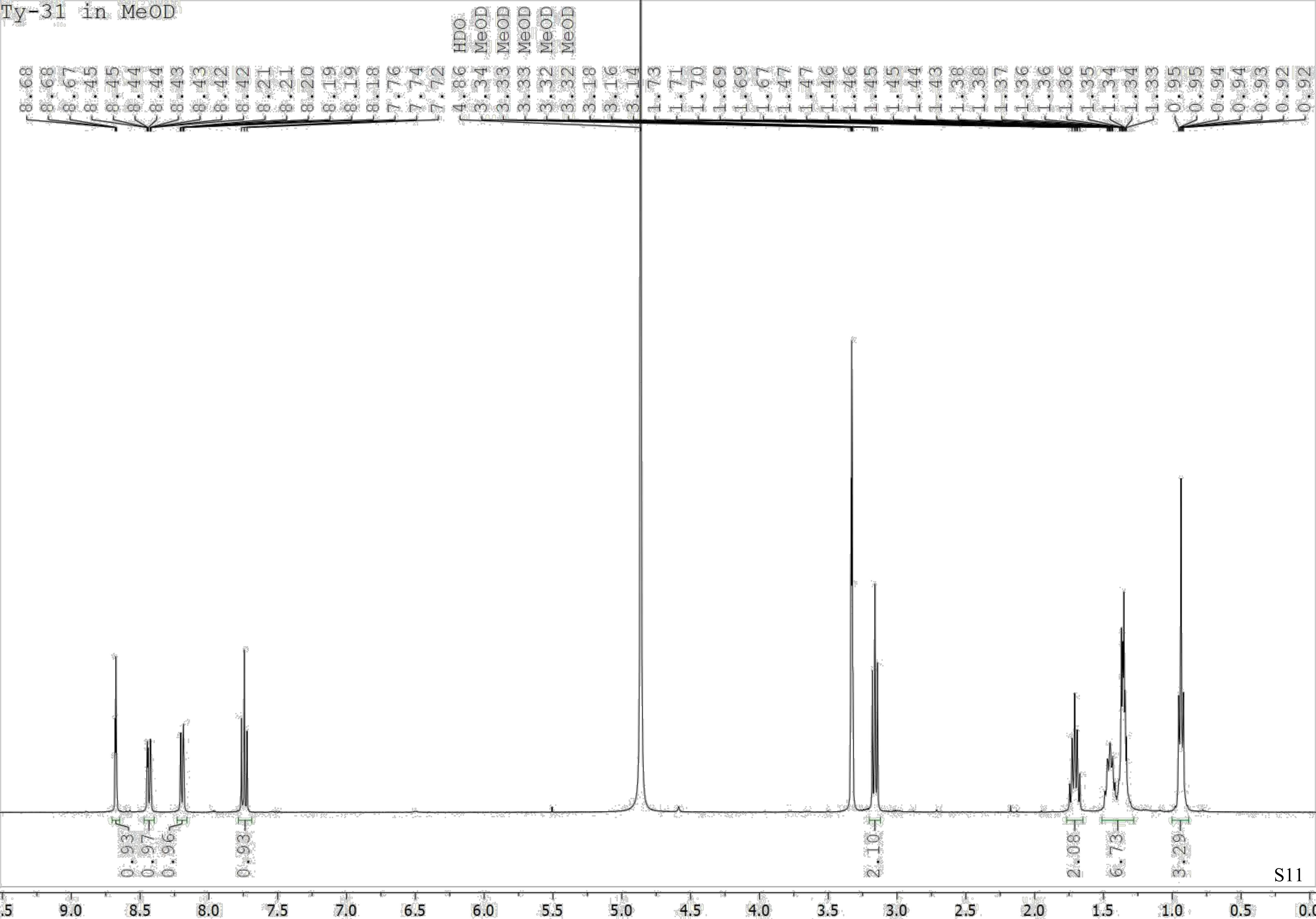

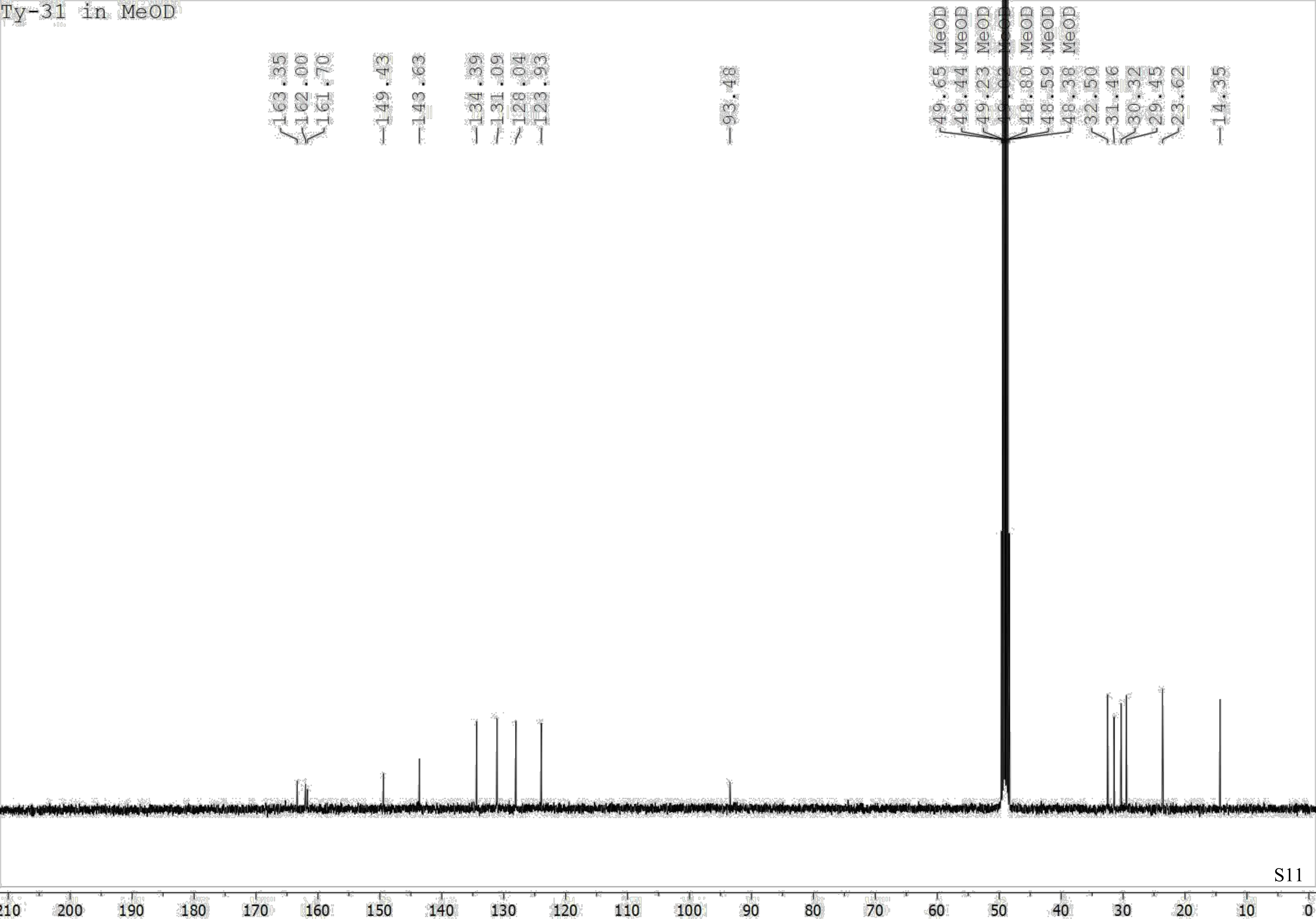
Ty-31 in MeOD
163.35
162.00
161.70
149.43
143.63
134.39
131.09
128.04
123.93
93.48
49.65 MeOD
49.44 MeOD
49.23 MeOD
48.80 MeOD
48.59 MeOD
48.38 MeOD
32.50
31.46
30.32
29.45
23.62
14.35
210
200
190
180
170
160
150
140
130
120
110
100
90
80
70
60
50
40
30
20
10
0

Ty-32 in DMSO

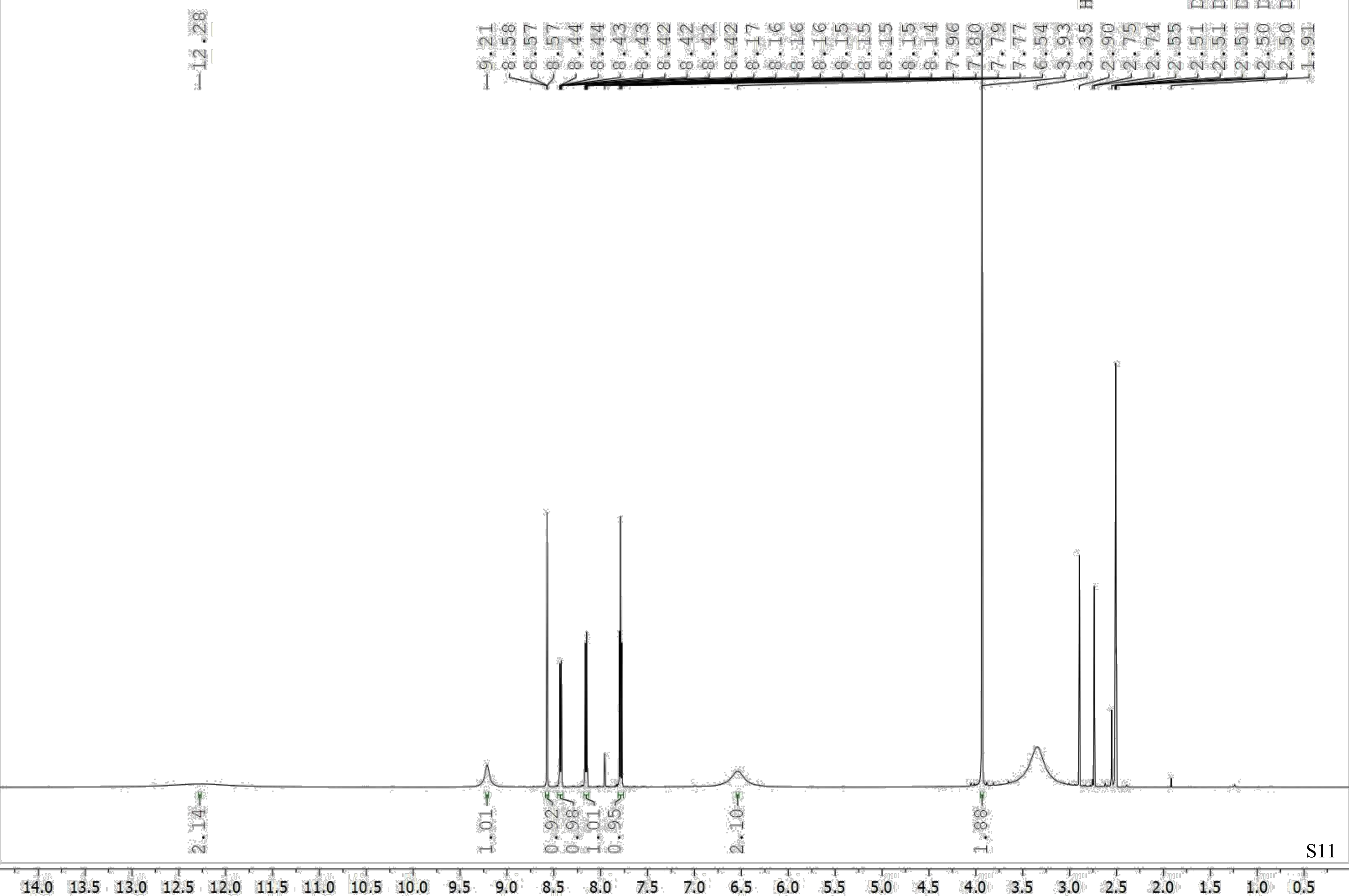

HDO
DMSO
12.28
9.21
8.58
8.57
8.57
8.44
8.44
8.43
8.43
8.42
8.42
8.42
8.42
8.17
8.16
8.16
8.16
8.15
8.15
8.15
8.15
8.14
7.96
7.80
7.79
7.77
6.54
3.93
3.35
2.90
2.75
2.74
2.55
2.51
2.51
2.51
2.50
2.50
1.91
2.14
1.01
0.92
0.98
1.01
0.95
2.10
1.88
14.0
13.5
13.0
12.5
12.0
11.5
11.0
10.5
10.0
9.5
9.0
8.5
8.0
7.5
7.0
6.5
6.0
5.5
5.0
4.5
4.0
3.5
3.0
2.5
2.0
1.5
1.0
0.5

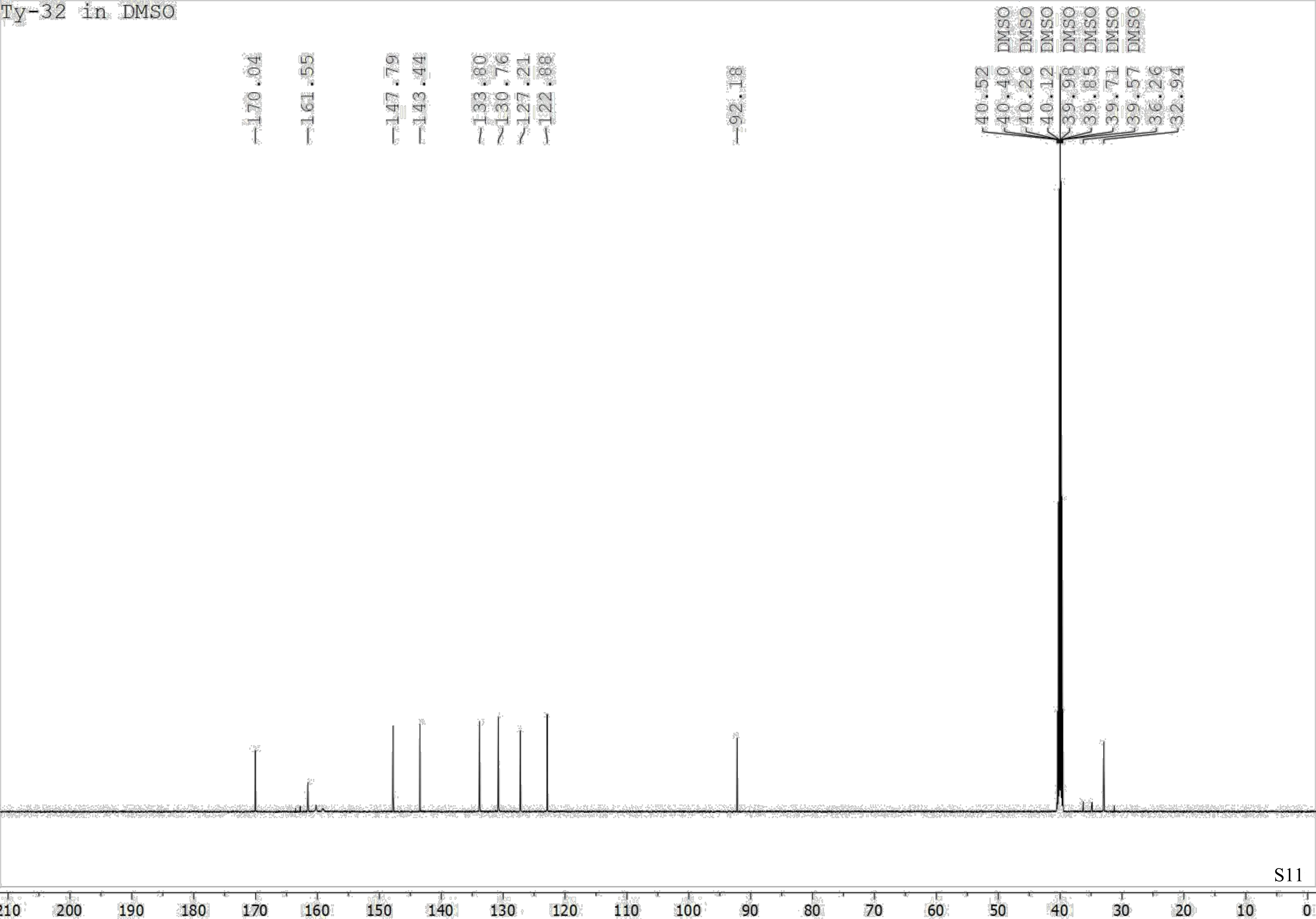

Ty-32 in DMSO
170.04
161.55
147.79
143.44
133.80
130.76
127.21
122.88
92.18
40.52 DMSO
40.40 DMSO
40.26 DMSO
40.12 DMSO
39.98 DMSO
39.85 DMSO
39.71 DMSO
39.57
36.26
32.94
210 200 190 180 170 160 150 140 130 120 110 100 90 80 70 60 50 40 30 20 10 0

Ty-33 in DMSO

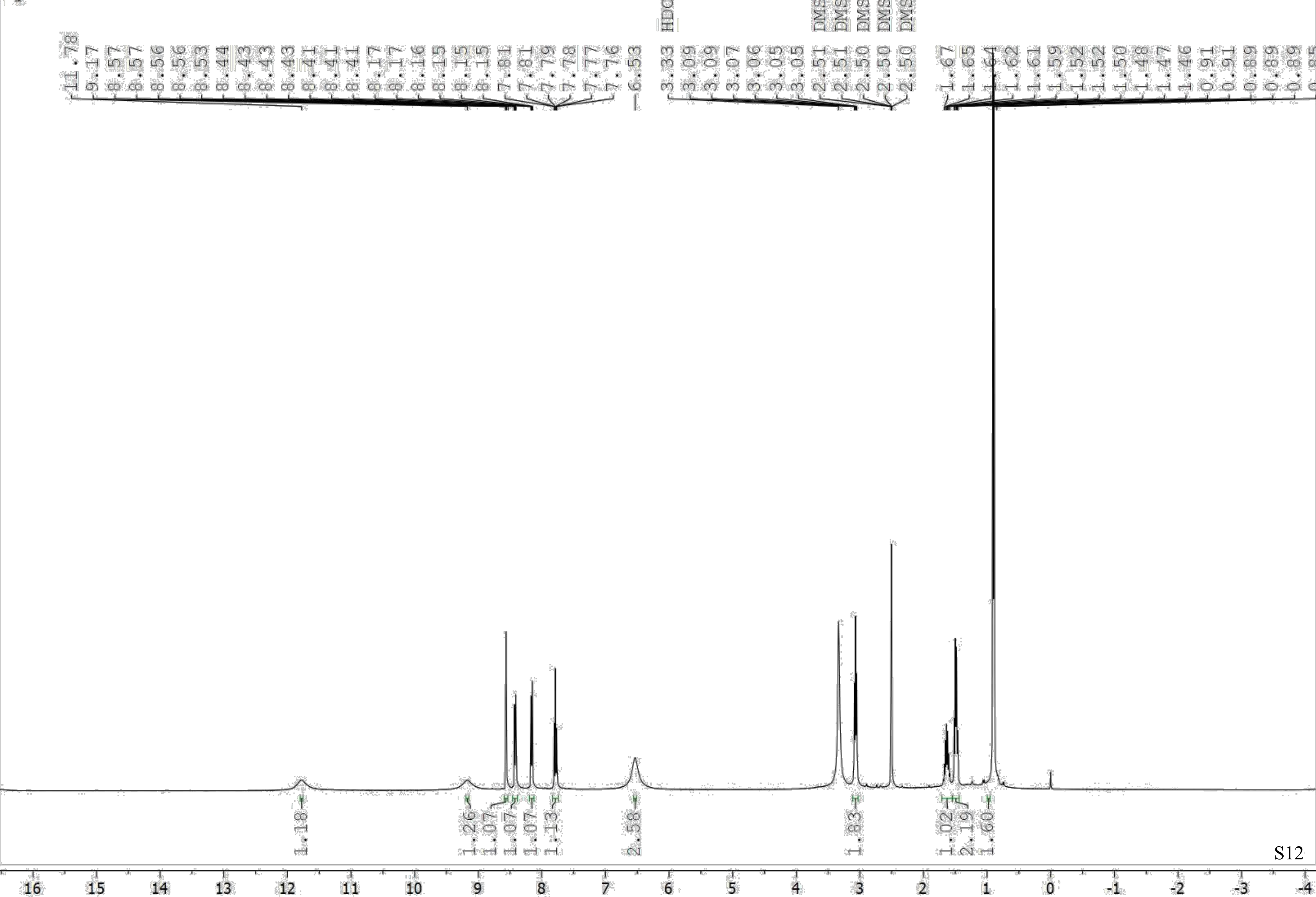
HDO
DMSO
DMSO
DMSO
DMSO
DMSO
11.78
9.17
8.57
8.57
8.56
8.56
8.53
8.44
8.43
8.43
8.43
8.41
8.41
8.41
8.17
8.17
8.16
8.15
8.15
8.15
7.81
7.81
7.79
7.78
7.77
7.76
6.53
3.33
3.09
3.09
3.07
3.06
3.05
3.05
2.51
2.51
2.50
2.50
2.50
1.67
1.65
1.64
1.62
1.61
1.59
1.52
1.52
1.50
1.48
1.47
1.46
0.91
0.91
0.89
0.89
0.89
1.18
1.26
1.07
1.07
1.07
1.13
2.58
1.83
1.02
2.19
1.60
16
15
14
13
12
11
10
9
8
7
6
5
4
3
2
1
0
-1
-2
-3
-4

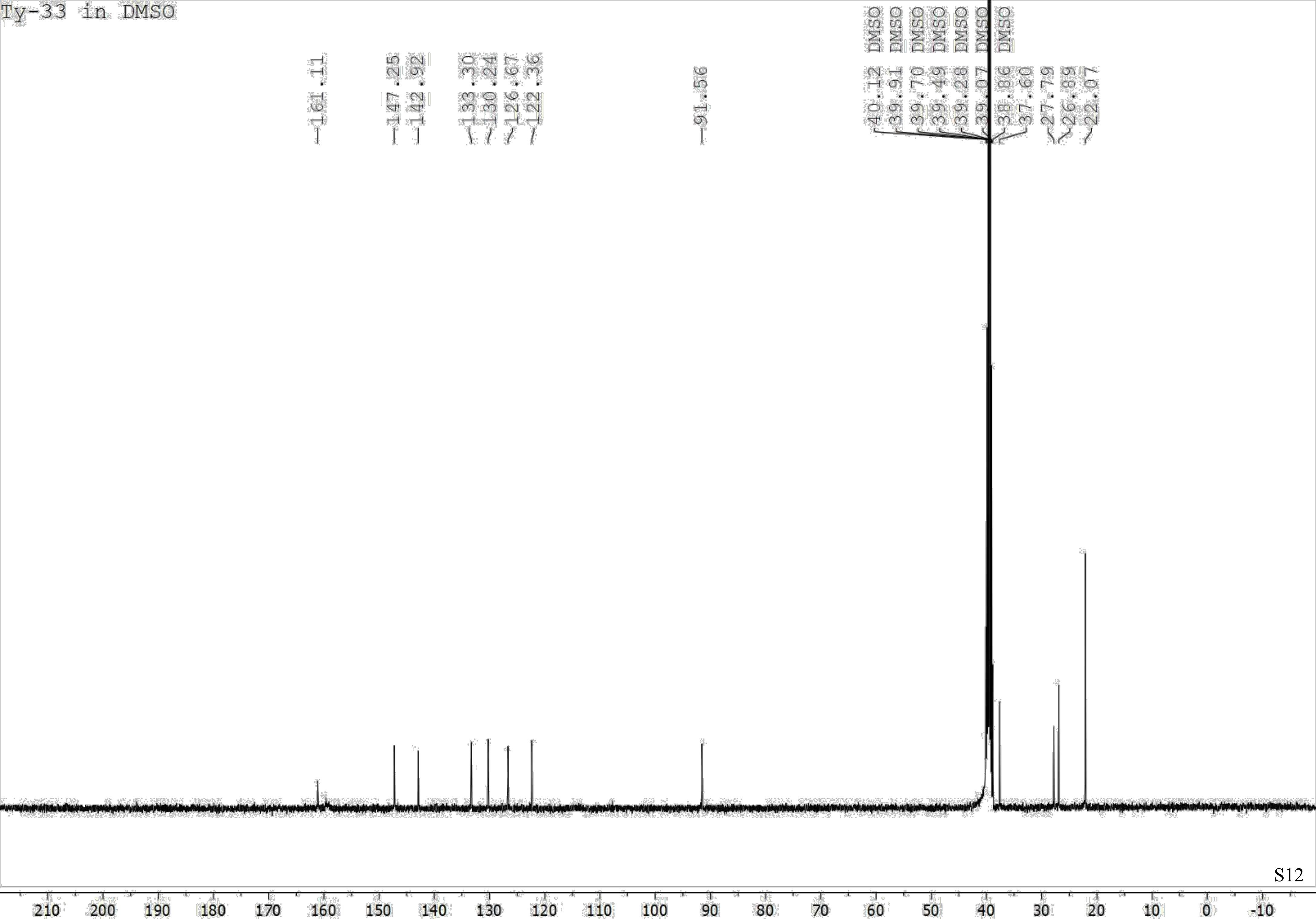
Ty-33 in DMSO
161.11
147.25
142.92
133.30
130.24
126.67
122.36
91.56
40.12 DMSO
39.91 DMSO
39.70 DMSO
39.49 DMSO
39.28 DMSO
39.07 DMSO
38.86 DMSO
37.60
27.79
26.89
22.07
210 200 190 180 170 160 150 140 130 120 110 100 90 80 70 60 50 40 30 20 10 0 -10

Ty-34 in DMSO

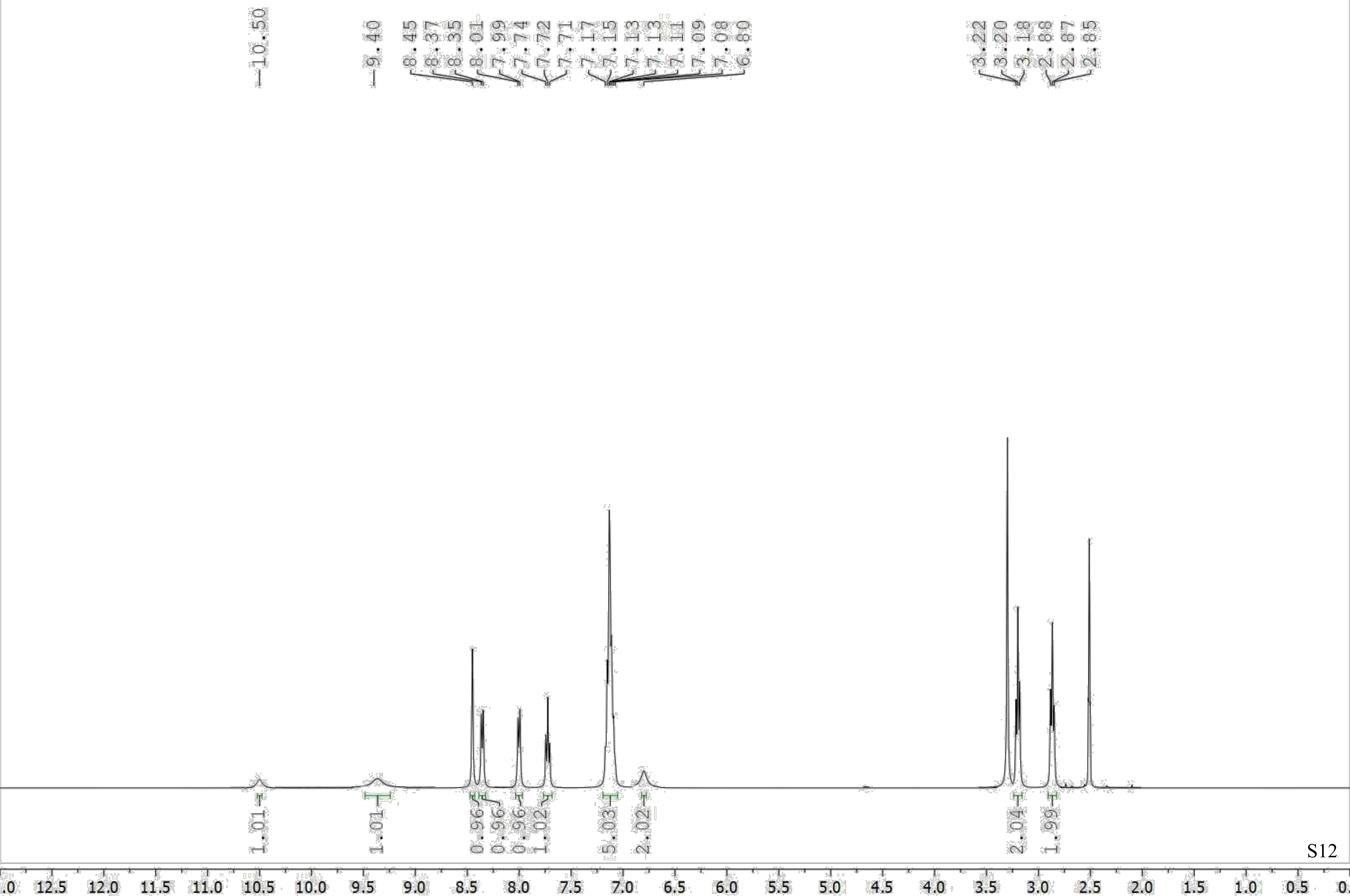

10.50
9.40
8.45
8.37
8.35
8.01
7.99
7.74
7.72
7.71
7.17
7.15
7.13
7.13
7.11
7.09
7.08
6.80
3.22
3.20
3.18
2.88
2.87
2.85
1.01
1.01
0.96
0.96
0.96
1.02
5.03
2.02
2.04
1.99
12.5
12.0
11.5
11.0
10.5
10.0
9.5
9.0
8.5
8.0
7.5
7.0
6.5
6.0
5.5
5.0
4.5
4.0
3.5
3.0
2.5
2.0
1.5
1.0
0.5

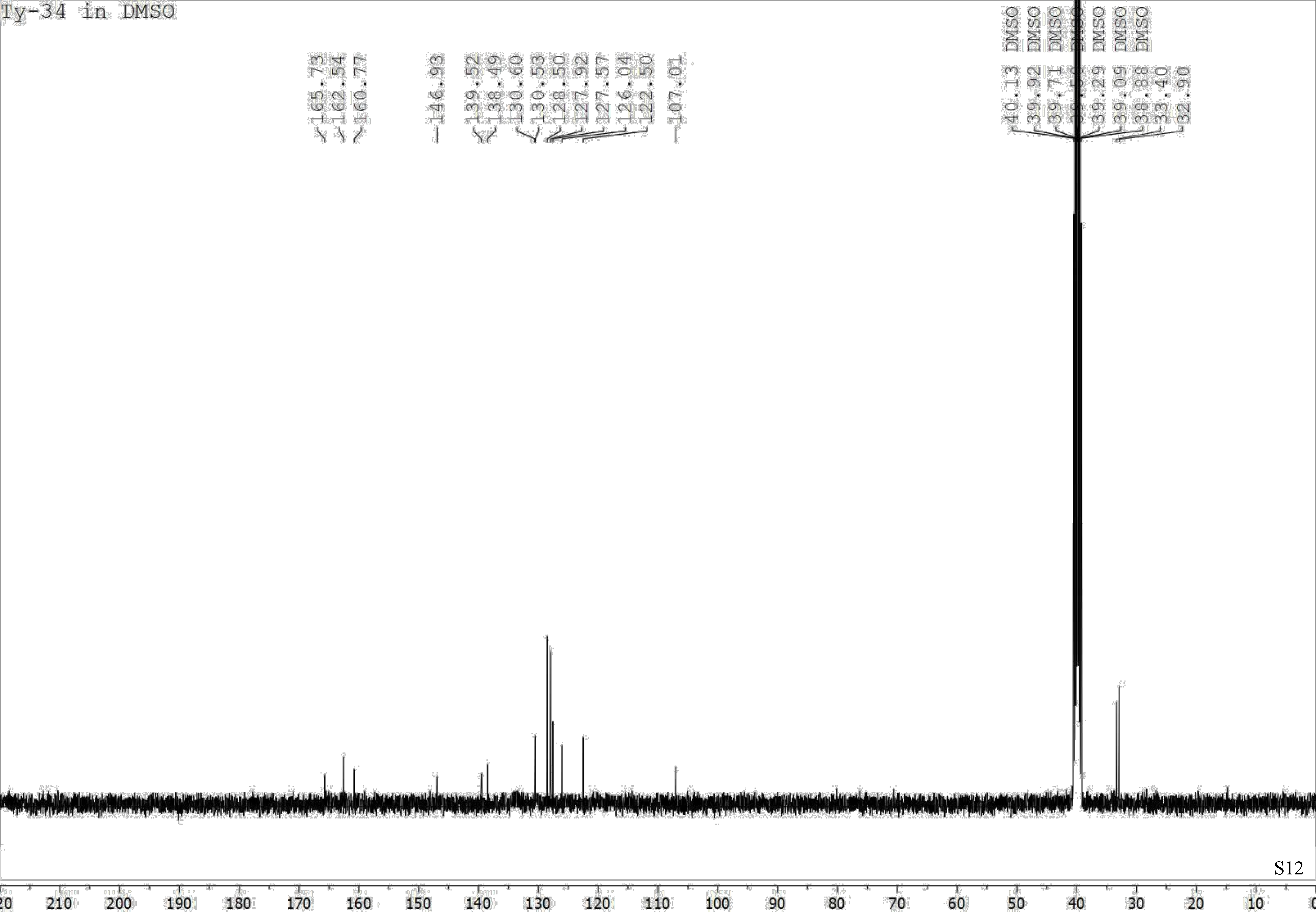

Ty-34 in DMSO
165.73
162.54
160.77
146.93
139.52
138.49
130.60
130.53
128.50
127.92
127.57
126.04
122.50
107.01
40.13 DMSO
39.92 DMSO
39.71 DMSO
39.29 DMSO
39.09 DMSO
38.88 DMSO
33.40
32.90
210
200
190
180
170
160
150
140
130
120
110
100
90
80
70
60
50
40
30
20
10

Ty-35 in DMSO

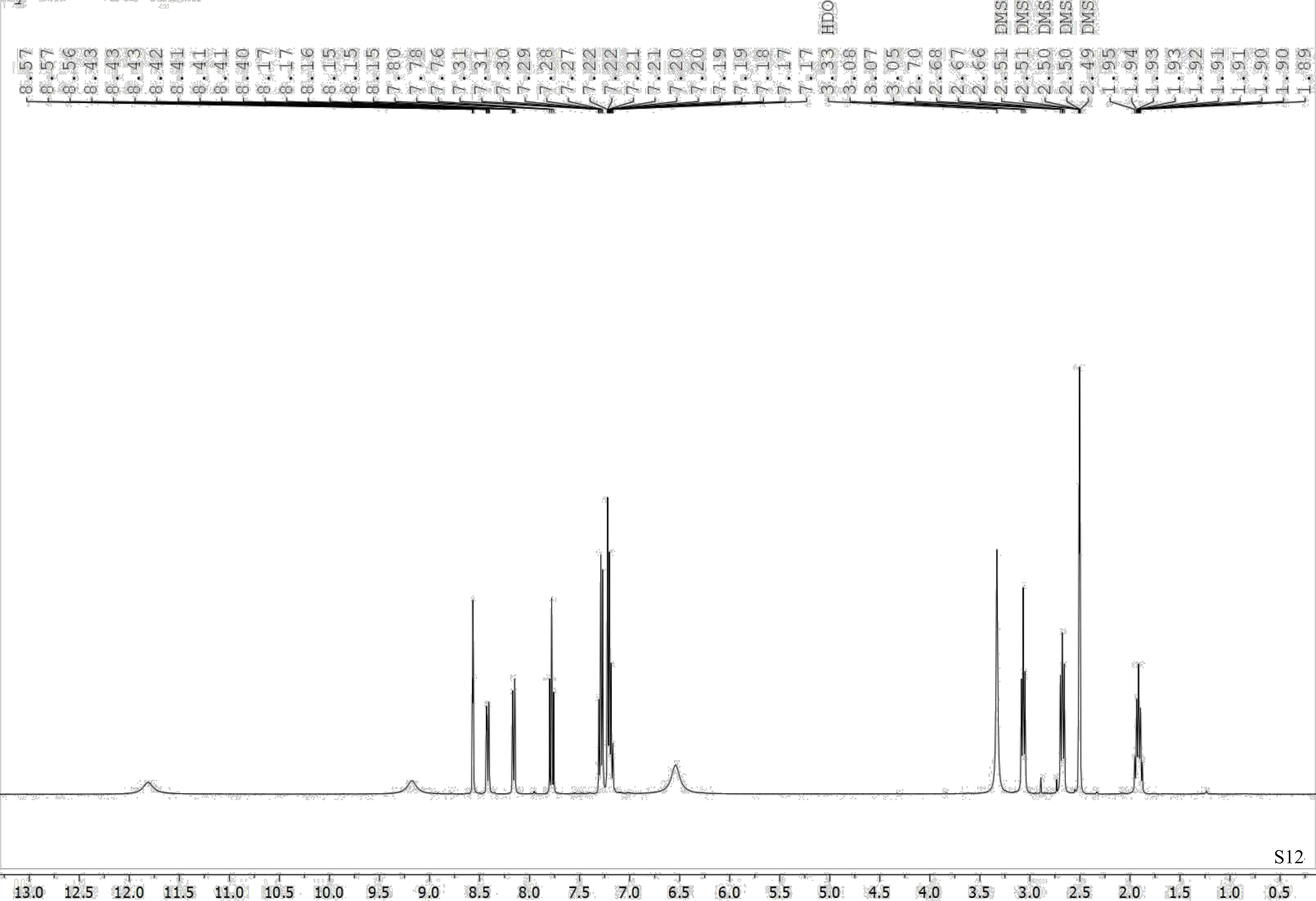
HDO
DMSO
DMSO
DMSO
DMSO
DMSO
8.57 8.57 8.56 8.43 8.43 8.43 8.42 8.41 8.41 8.41 8.40 8.17 8.17 8.16 8.15 8.15 8.15 7.80 7.78 7.76 7.31 7.31 7.30 7.29 7.28 7.27 7.22 7.22 7.21 7.21 7.20 7.20 7.19 7.19 7.18 7.17 7.17 3.33 3.08 3.07 3.05 2.70 2.68 2.67 2.66 2.51 2.51 2.50 2.50 2.49 1.95 1.94 1.93 1.93 1.92 1.91 1.91 1.90 1.90 1.89
13.0 12.5 12.0 11.5 11.0 10.5 10.0 9.5 9.0 8.5 8.0 7.5 7.0 6.5 6.0 5.5 5.0 4.5 4.0 3.5 3.0 2.5 2.0 1.5 1.0 0.5

Ty-35 in DMSO

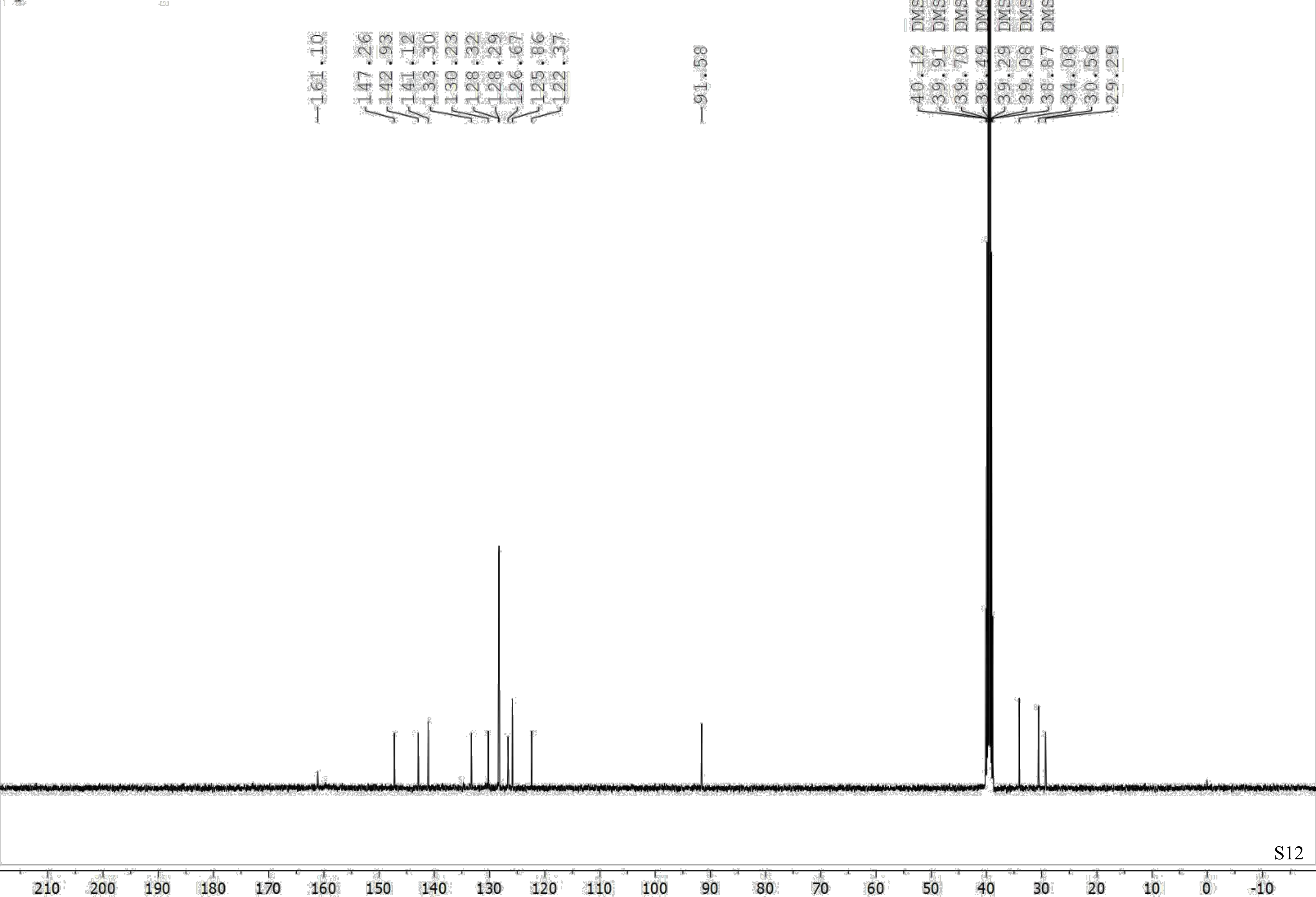

161.10
147.26
142.93
141.12
133.30
130.23
128.32
128.29
126.67
125.86
122.37
91.58
40.12 DMSO
39.91 DMSO
39.70 DMSO
39.49 DMSO
39.29 DMSO
39.08 DMSO
38.87 DMSO
34.08
30.56
29.29
210
200
190
180
170
160
150
140
130
120
110
100
90
80
70
60
50
40
30
20
10
0
-10

Ty-36 in DMSO

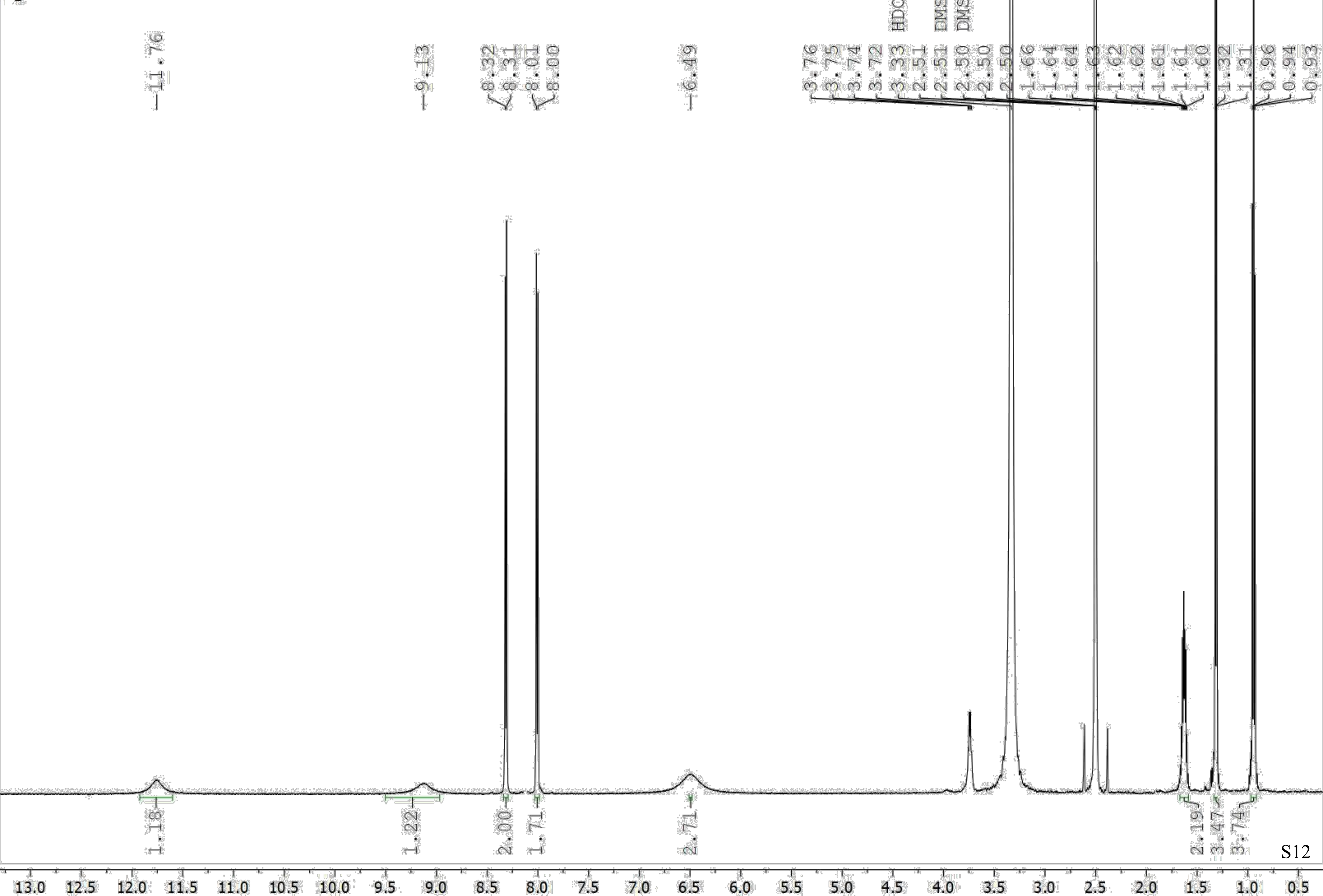
HDO
DMSO
DMSO
11.76
9.13
8.32
8.31
8.01
8.00
6.49
3.76
3.75
3.74
3.72
3.33
2.51
2.51
2.50
2.50
2.50
1.66
1.64
1.64
1.63
1.62
1.62
1.61
1.61
1.60
1.32
1.31
0.96
0.94
0.93
1.18
1.22
2.00
1.71
2.71
2.19
3.47
3.74
13.0 12.5 12.0 11.5 11.0 10.5 10.0 9.5 9.0 8.5 8.0 7.5 7.0 6.5 6.0 5.5 5.0 4.5 4.0 3.5 3.0 2.5 2.0 1.5 1.0 0.5

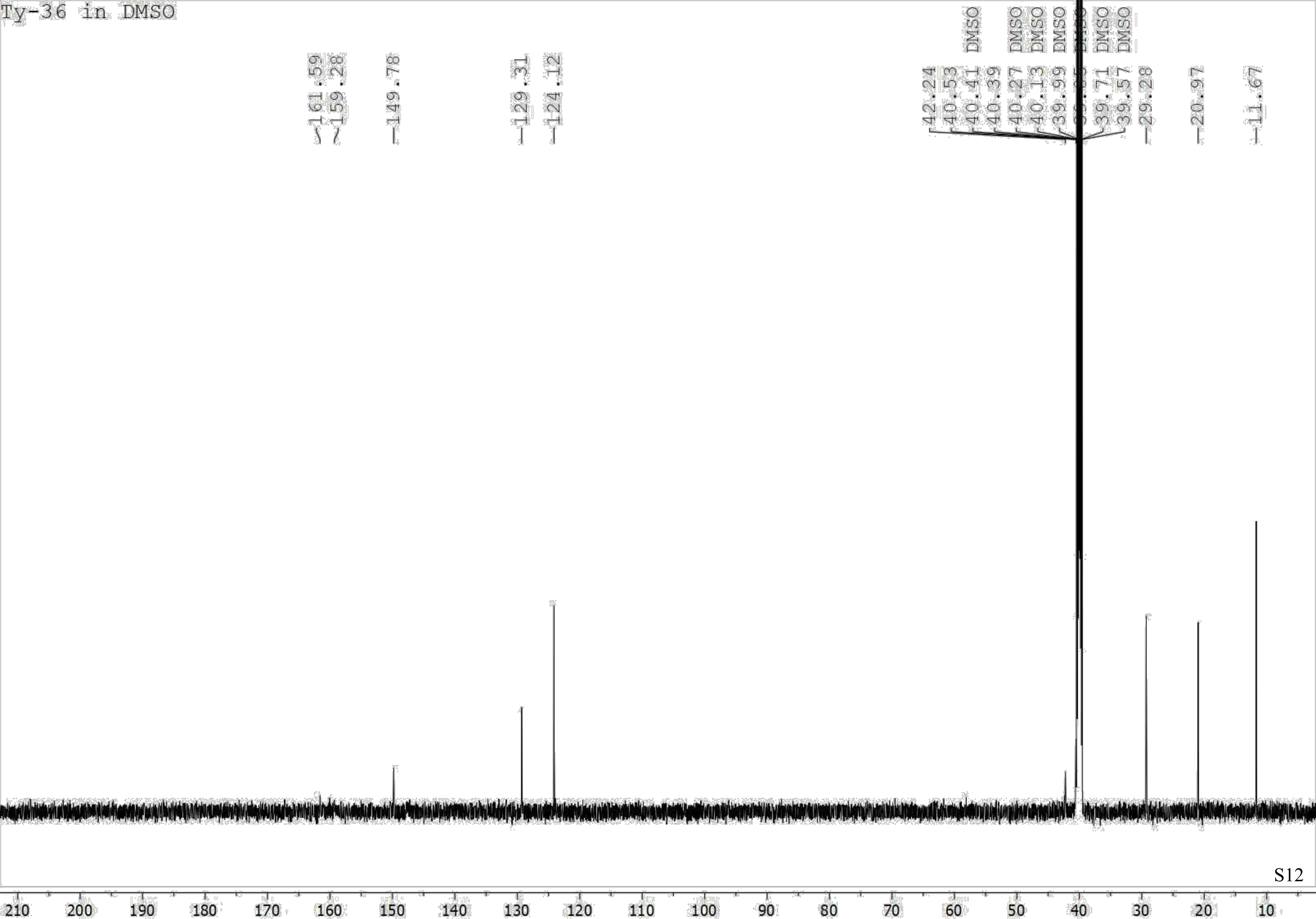
Ty-36 in DMSO
161.59
159.28
149.78
129.31
124.12
42.24
40.53
40.41
40.39
40.27
40.13
39.99
39.71
39.57
29.28
20.97
11.67
DMSO
DMSO
DMSO
DMSO
DMSO
DMSO
210
200
190
180
170
160
150
140
130
120
110
100
90
80
70
60
50
40
30
20
10

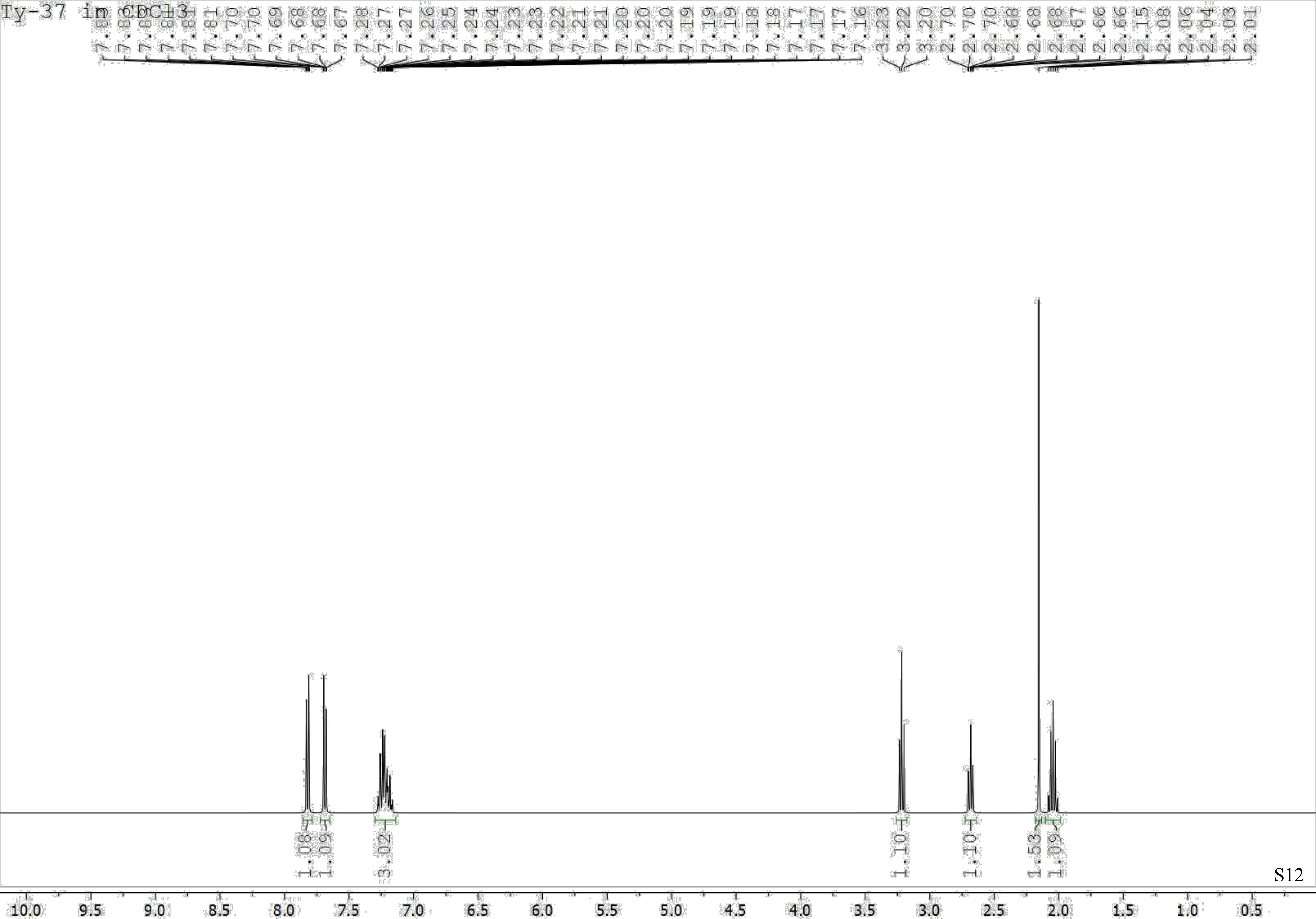
Ty-37 in CDCl3
1.08
1.09
3.02
1.10
1.10
1.53
1.09
10.0 9.5 9.0 8.5 8.0 7.5 7.0 6.5 6.0 5.5 5.0 4.5 4.0 3.5 3.0 2.5 2.0 1.5 1.0 0.5

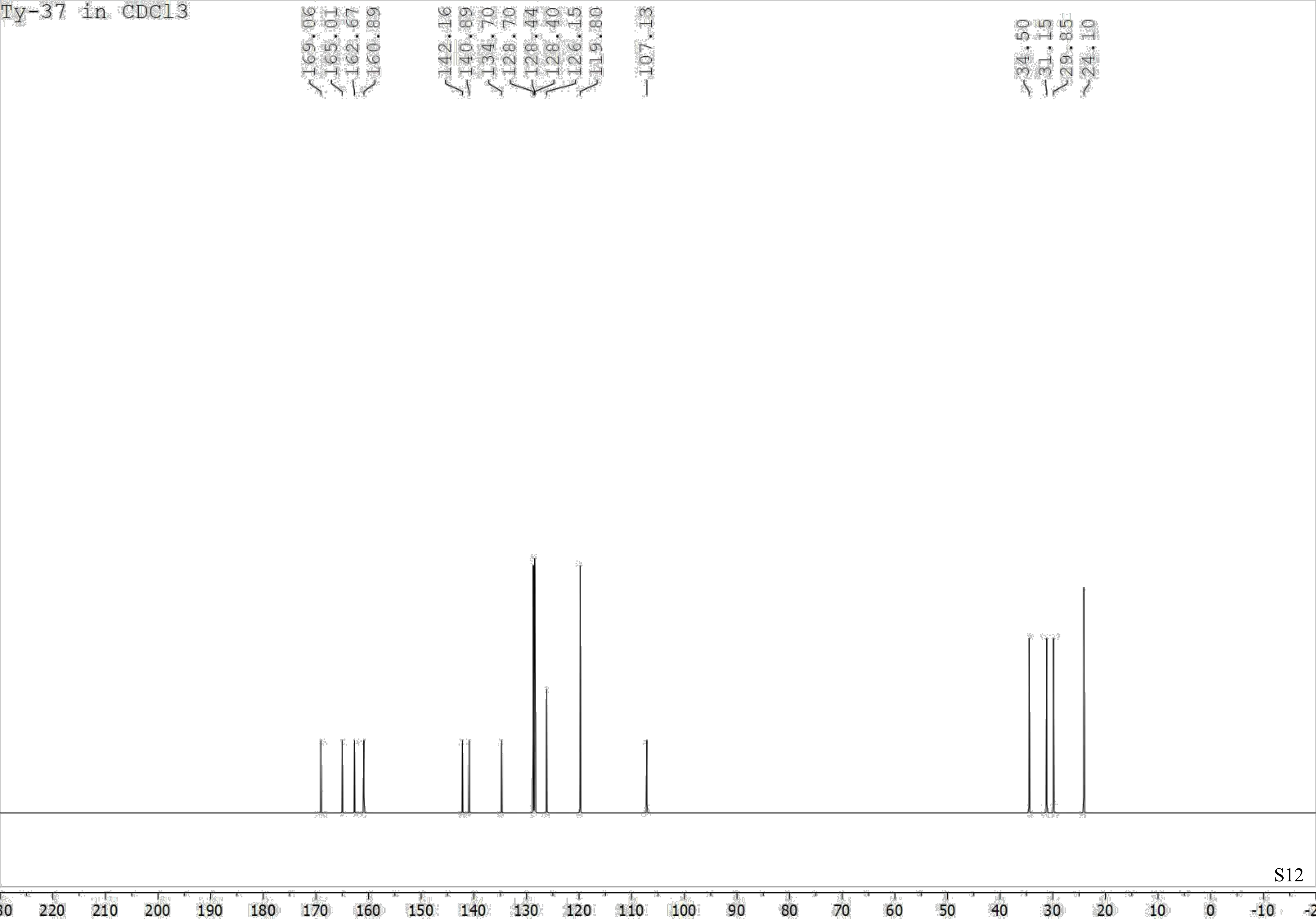

Ty-37 in CDCl3
169.06
165.01
162.67
160.89
142.16
140.89
134.70
128.70
128.44
128.40
126.15
119.80
107.13
34.50
31.15
29.85
24.10
230 220 210 200 190 180 170 160 150 140 130 120 110 100 90 80 70 60 50 40 30 20 10 0 -10 -20

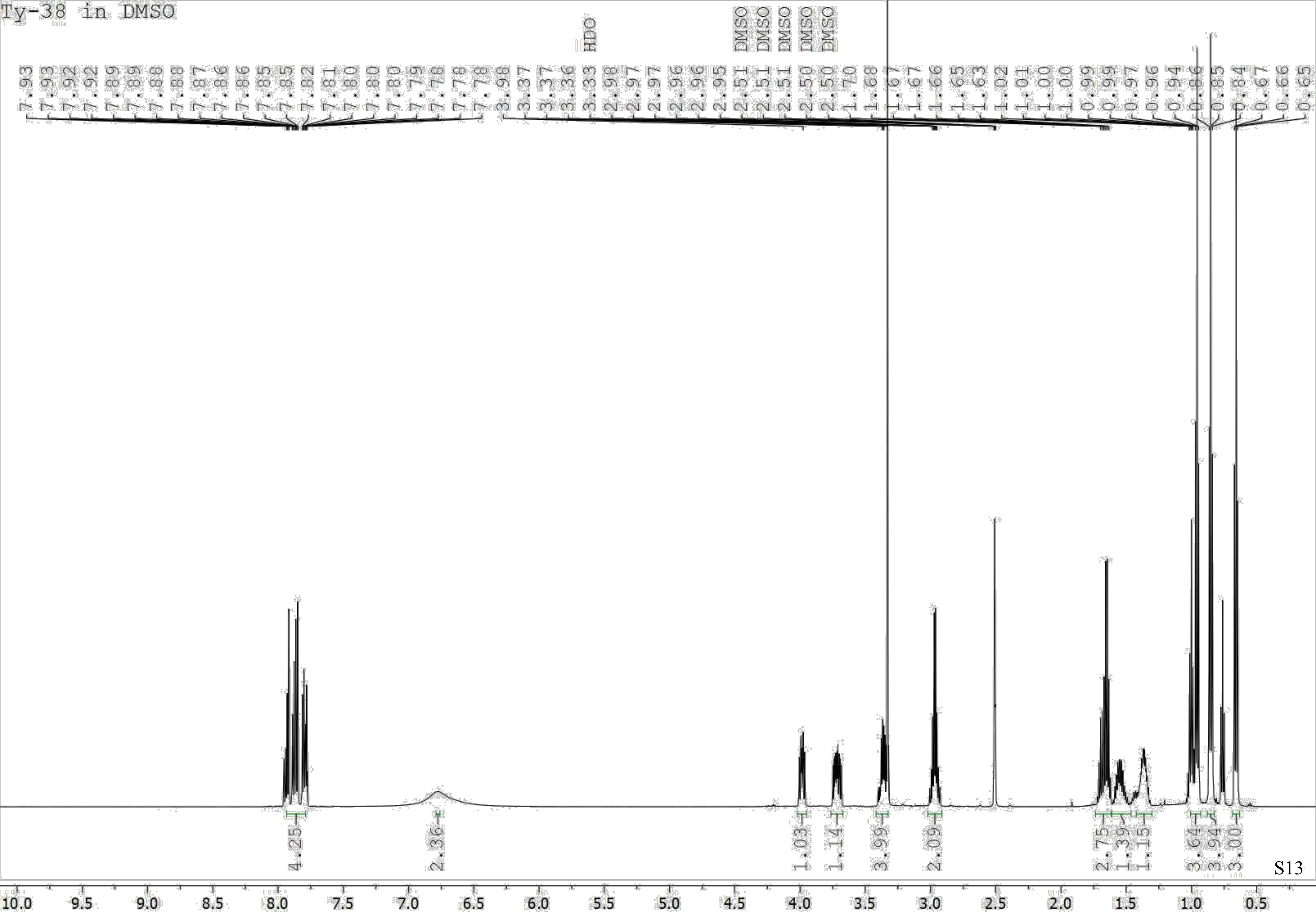
Ty-38 in DMSO
HDO
DMSO
7.93
7.93
7.92
7.92
7.89
7.89
7.88
7.88
7.87
7.86
7.86
7.85
7.85
7.82
7.81
7.80
7.80
7.80
7.79
7.78
7.78
7.78
3.98
3.37
3.37
3.36
3.33
2.98
2.97
2.97
2.96
2.96
2.95
2.51
2.51
2.51
2.50
2.50
1.70
1.68
1.67
1.67
1.66
1.65
1.63
1.02
1.01
1.00
1.00
0.99
0.99
0.97
0.96
0.94
0.86
0.85
0.84
0.67
0.66
0.65
4.25
2.36
1.03
1.14
3.99
2.09
2.75
1.39
1.15
3.64
3.94
3.00
10.0
9.5
9.0
8.5
8.0
7.5
7.0
6.5
6.0
5.5
5.0
4.5
4.0
3.5
3.0
2.5
2.0
1.5
1.0
0.5

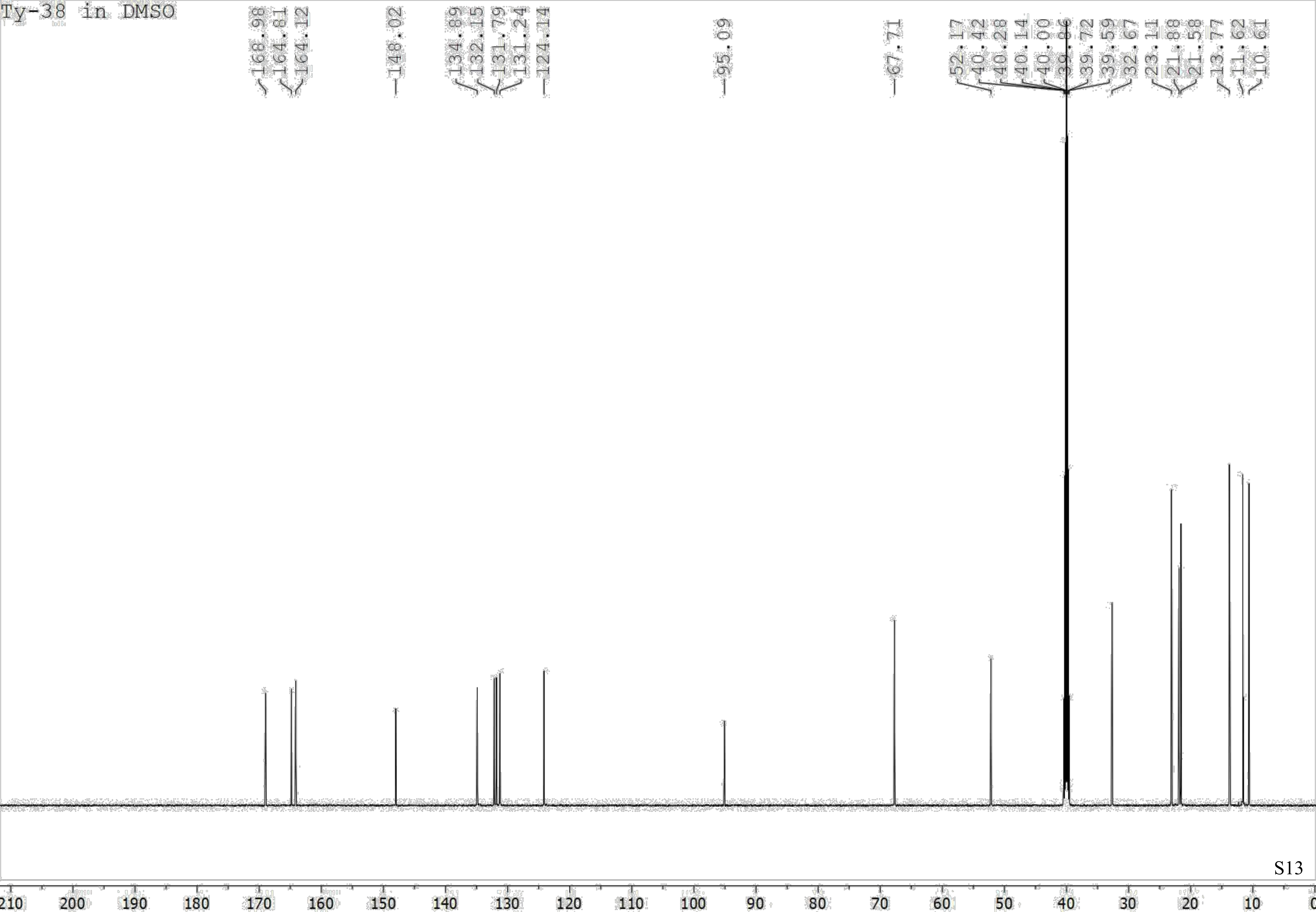
Ty-38 in DMSO
168.98
164.81
164.12
148.02
134.89
132.15
131.79
131.24
124.14
95.09
67.71
52.17
40.42
40.28
40.14
40.00
39.86
39.72
39.59
32.67
23.11
21.88
21.58
13.77
11.62
10.61
210
200
190
180
170
160
150
140
130
120
110
100
90
80
70
60
50
40
30
20
10
0

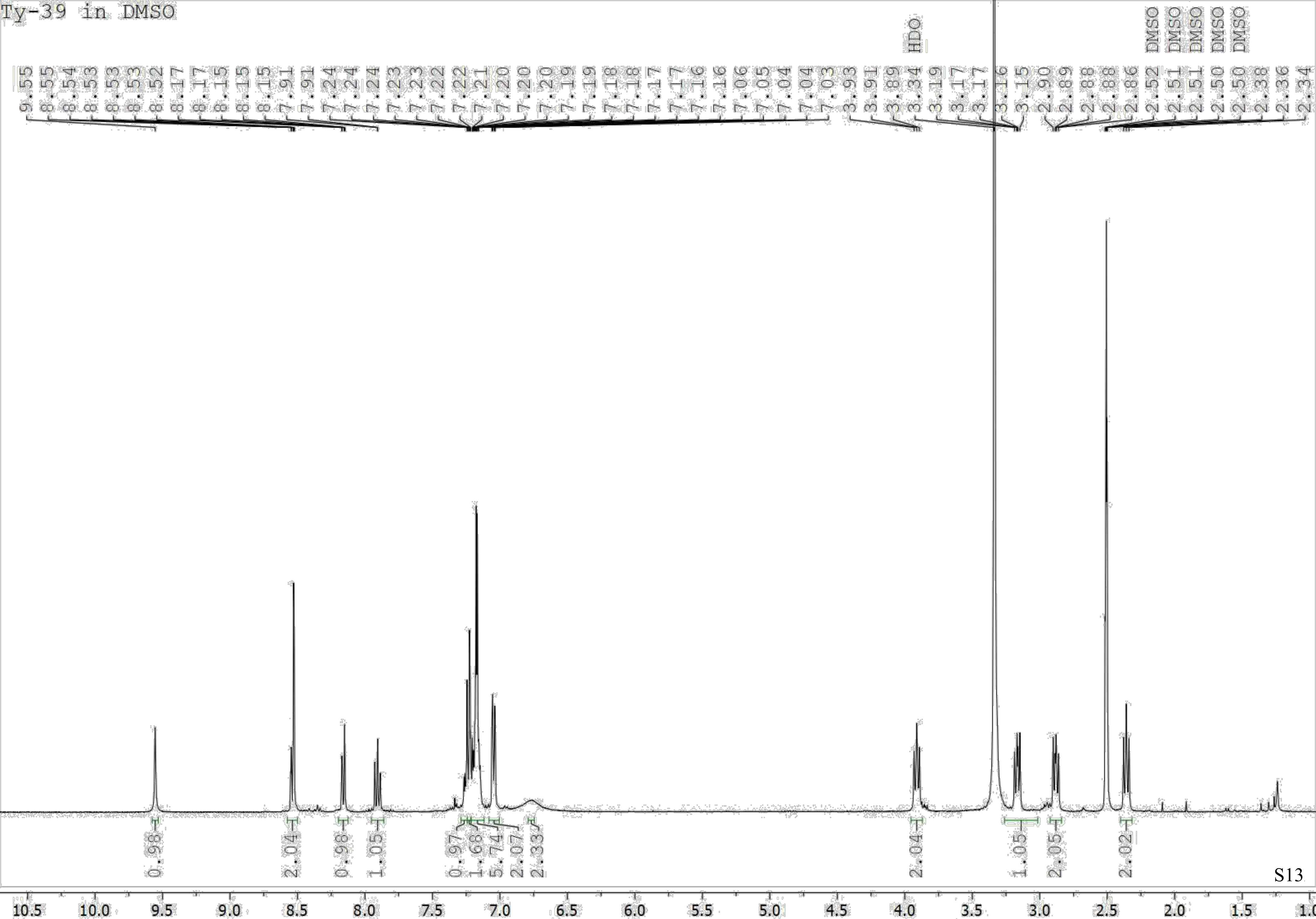
Ty-39 in DMSO
HDO
DMSO DMSO DMSO DMSO DMSO
9.55 8.55 8.54 8.53 8.53 8.53 8.52 8.17 8.17 8.15 8.15 8.15 7.91 7.91 7.24 7.24 7.24 7.23 7.23 7.22 7.22 7.21 7.20 7.20 7.20 7.19 7.19 7.18 7.18 7.17 7.17 7.16 7.16 7.06 7.05 7.04 7.04 7.03 3.93 3.91 3.89 3.34 3.19 3.17 3.17 3.16 3.15 2.90 2.89 2.88 2.88 2.86 2.52 2.51 2.51 2.50 2.50 2.38 2.36 2.34
0.98 2.04 0.98 1.05 0.97 1.68 5.74 2.07 2.33 2.04 1.05 2.05 2.02
10.5 10.0 9.5 9.0 8.5 8.0 7.5 7.0 6.5 6.0 5.5 5.0 4.5 4.0 3.5 3.0 2.5 2.0 1.5 1.0

Ty-39 in DMSO

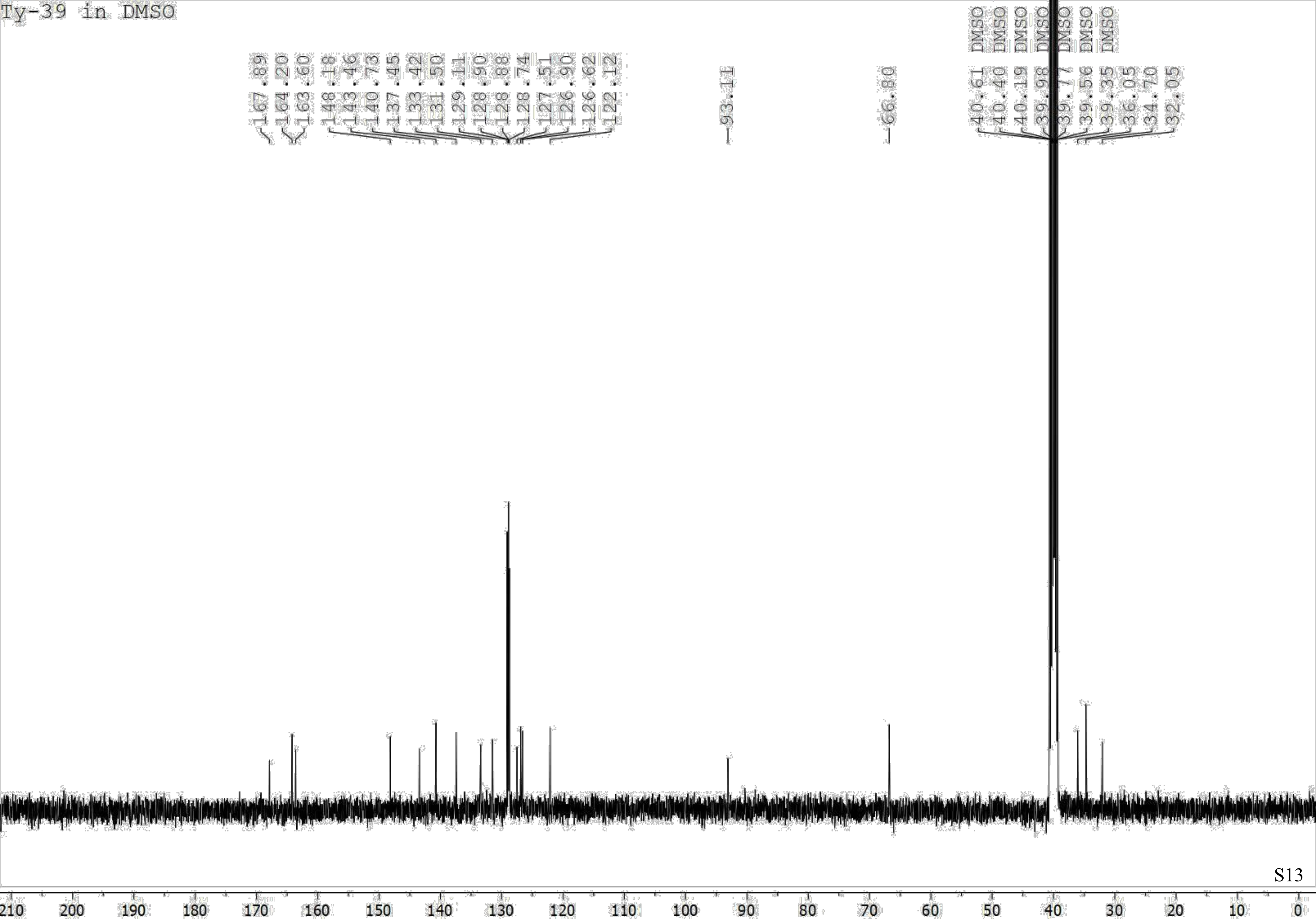
167.89
164.20
163.60
148.18
143.46
140.73
137.45
133.42
131.50
129.11
128.90
128.88
128.74
127.51
126.90
126.62
122.12
93.11
66.80
40.61 DMSO
40.40 DMSO
40.19 DMSO
39.98 DMSO
39.77 DMSO
39.56 DMSO
39.35 DMSO
36.05
34.70
32.05
210
200
190
180
170
160
150
140
130
120
110
100
90
80
70
60
50
40
30
20
10
0

Ty-40 in DMSO

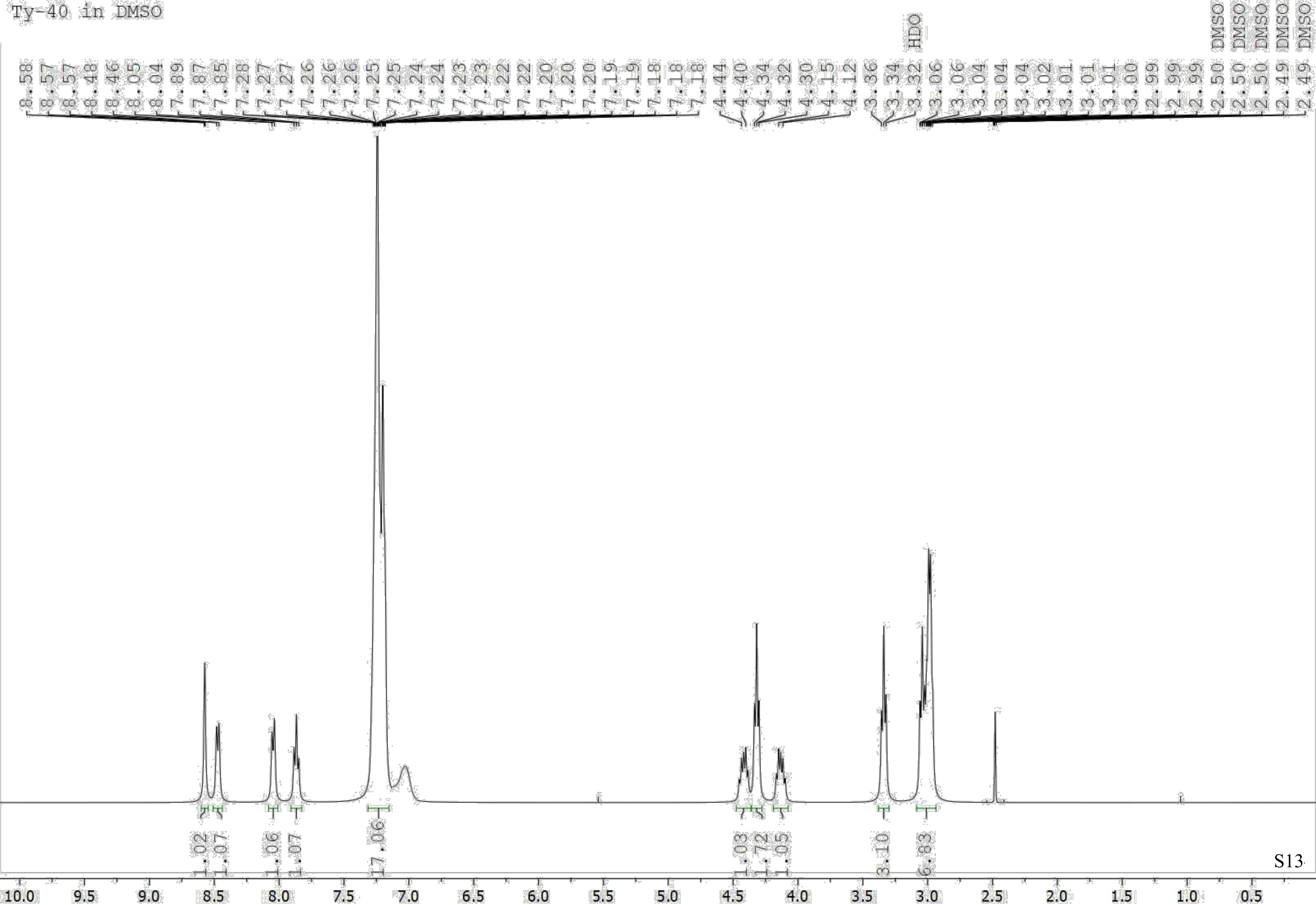

Ty-40 in DMSO

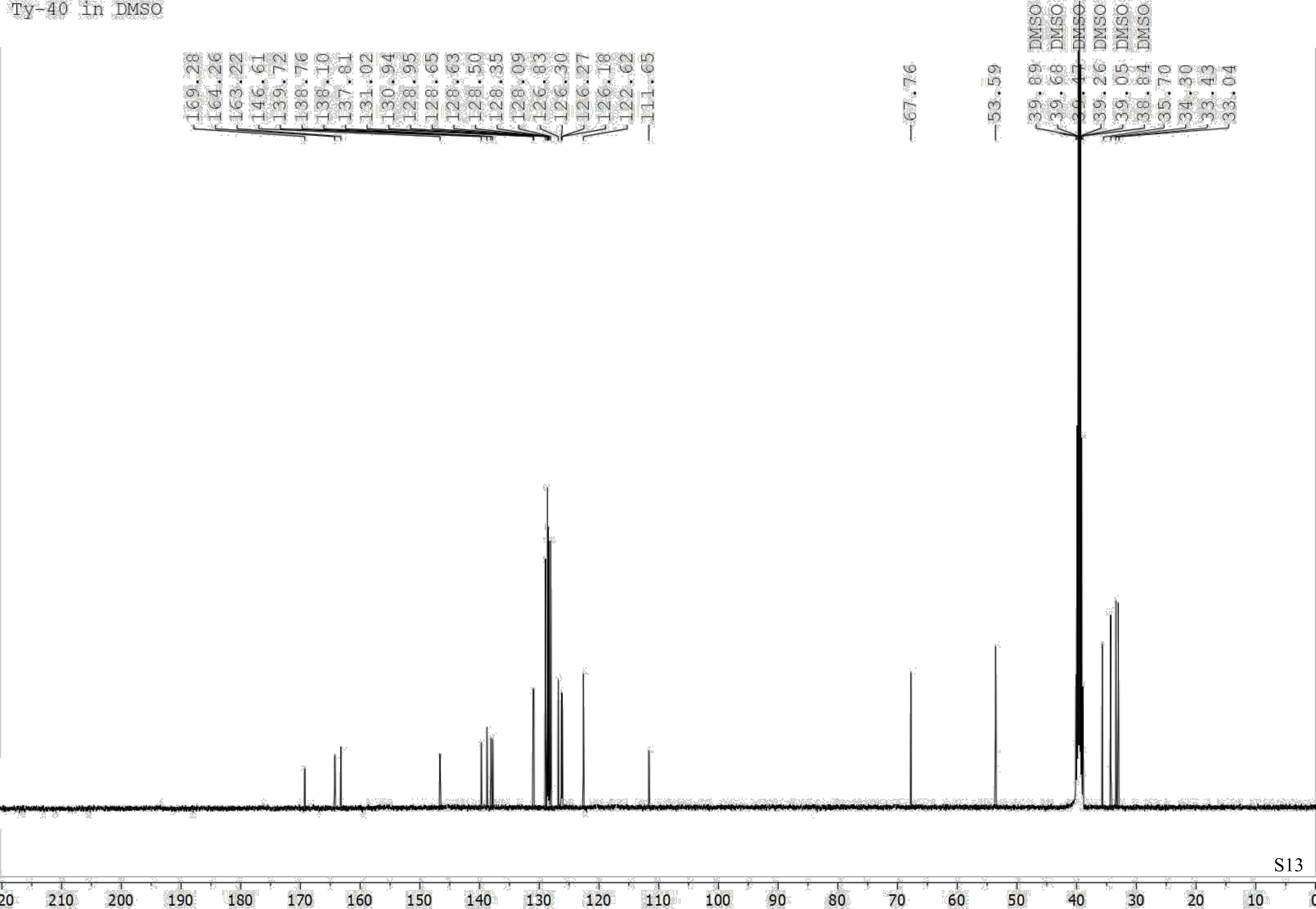
169.28
164.26
163.22
146.61
139.72
138.76
138.10
137.81
131.02
130.94
128.95
128.65
128.63
128.50
128.35
128.09
126.83
126.30
126.27
126.18
122.62
111.65
67.76
53.59
39.89 DMSO
39.68 DMSO
39.47 DMSO
39.26 DMSO
39.05 DMSO
38.84 DMSO
35.70
34.30
33.43
33.04
220 210 200 190 180 170 160 150 140 130 120 110 100 90 80 70 60 50 40 30 20 10 0

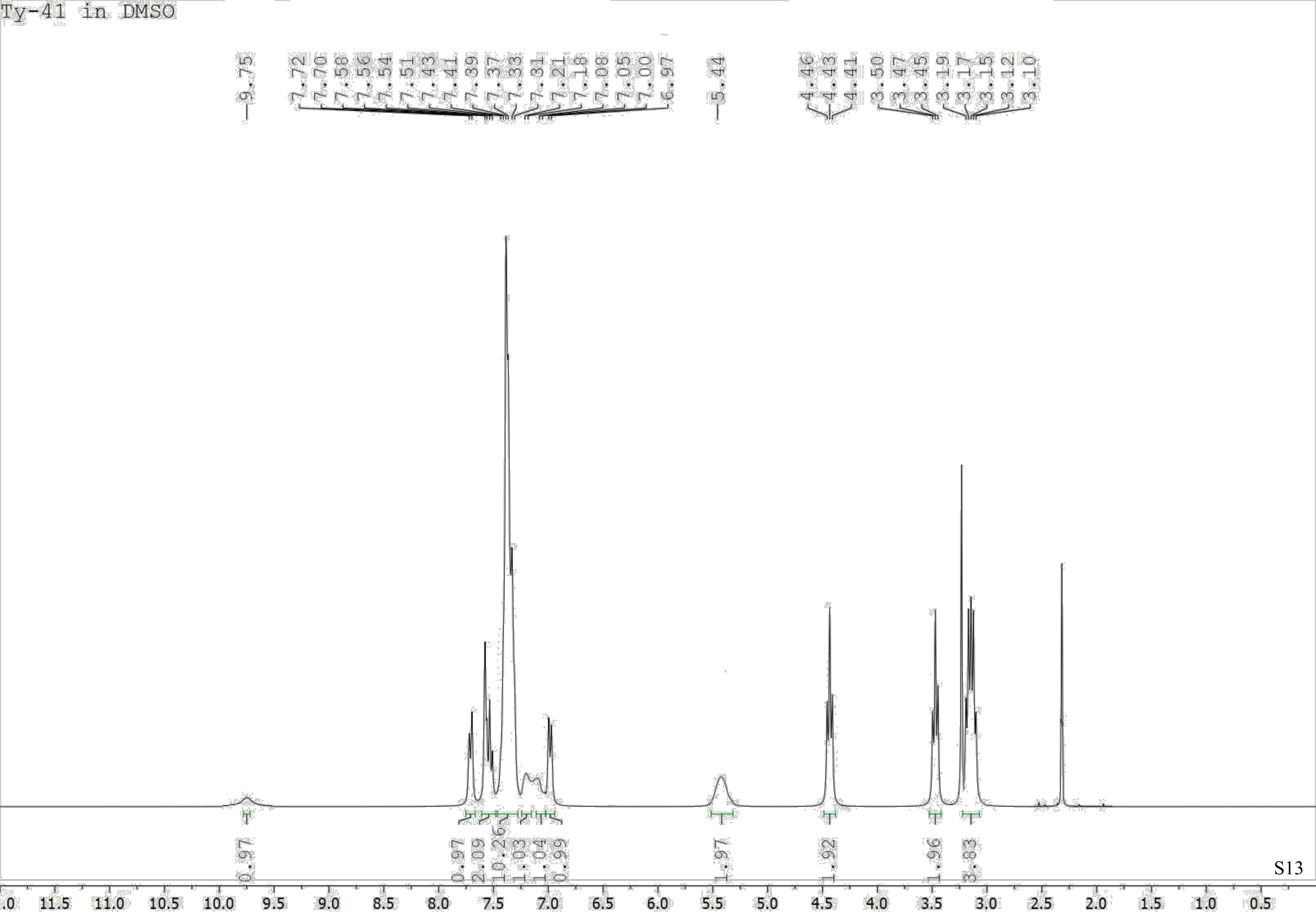
Ty-41 in DMSO
9.75
7.72 7.70 7.58 7.56 7.54 7.51 7.43 7.41 7.39 7.37 7.33 7.31 7.21 7.18 7.08 7.05 7.00 6.97
5.44
4.46 4.43 4.41
3.50 3.47 3.45 3.19 3.17 3.15 3.12 3.10
0.97
0.97 2.09 10.26 1.03 1.04 0.99
1.97
1.92
1.96
3.83
11.5 11.0 10.5 10.0 9.5 9.0 8.5 8.0 7.5 7.0 6.5 6.0 5.5 5.0 4.5 4.0 3.5 3.0 2.5 2.0 1.5 1.0 0.5

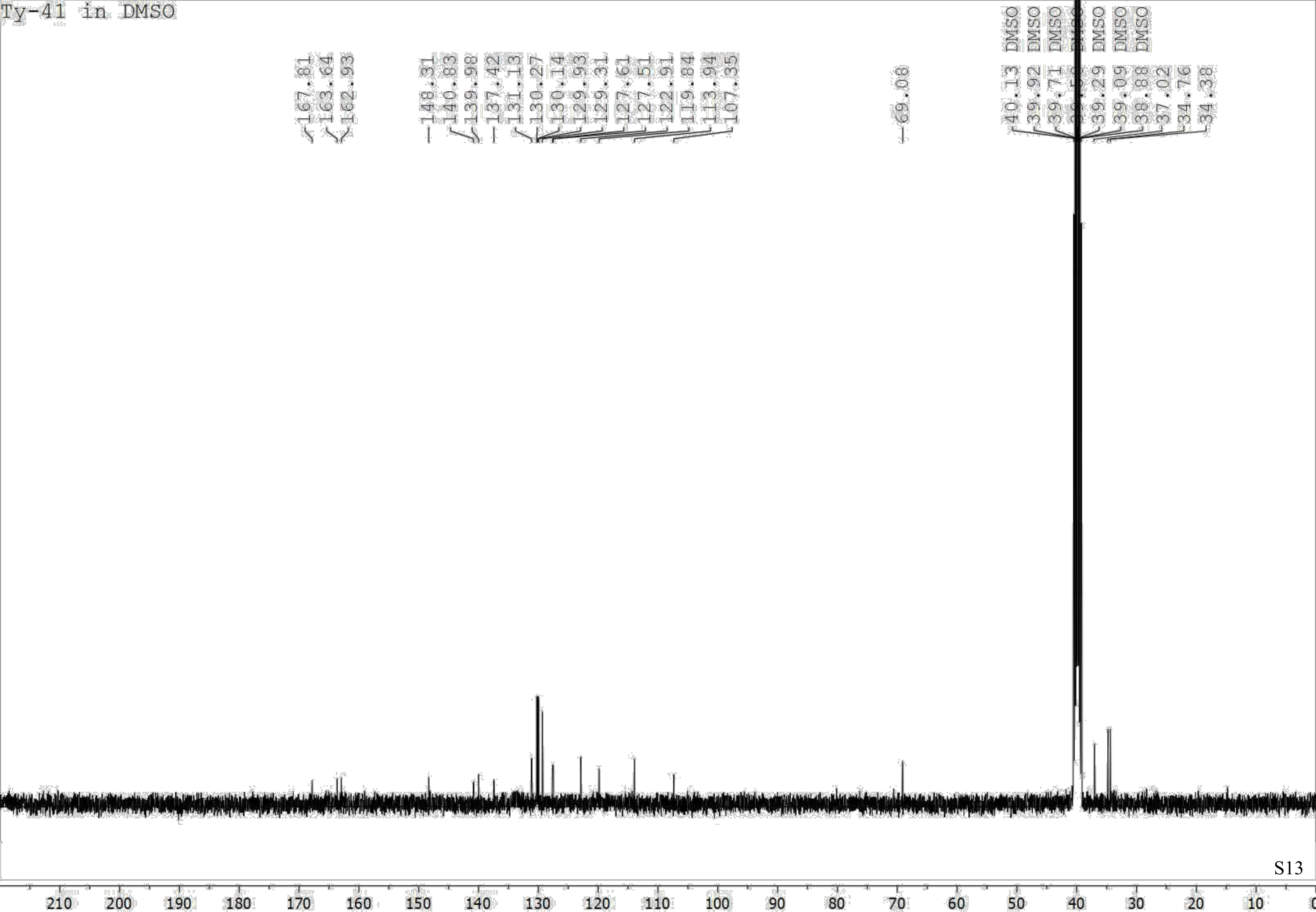
Ty-41 in DMSO
167.81
163.64
162.93
148.31
140.83
139.98
137.42
131.13
130.27
130.14
129.93
129.31
127.61
127.51
122.91
119.84
113.94
107.35
69.08
40.13 DMSO
39.92 DMSO
39.71 DMSO
39.29 DMSO
39.09 DMSO
38.88 DMSO
37.02
34.76
34.38
210 200 190 180 170 160 150 140 130 120 110 100 90 80 70 60 50 40 30 20 10

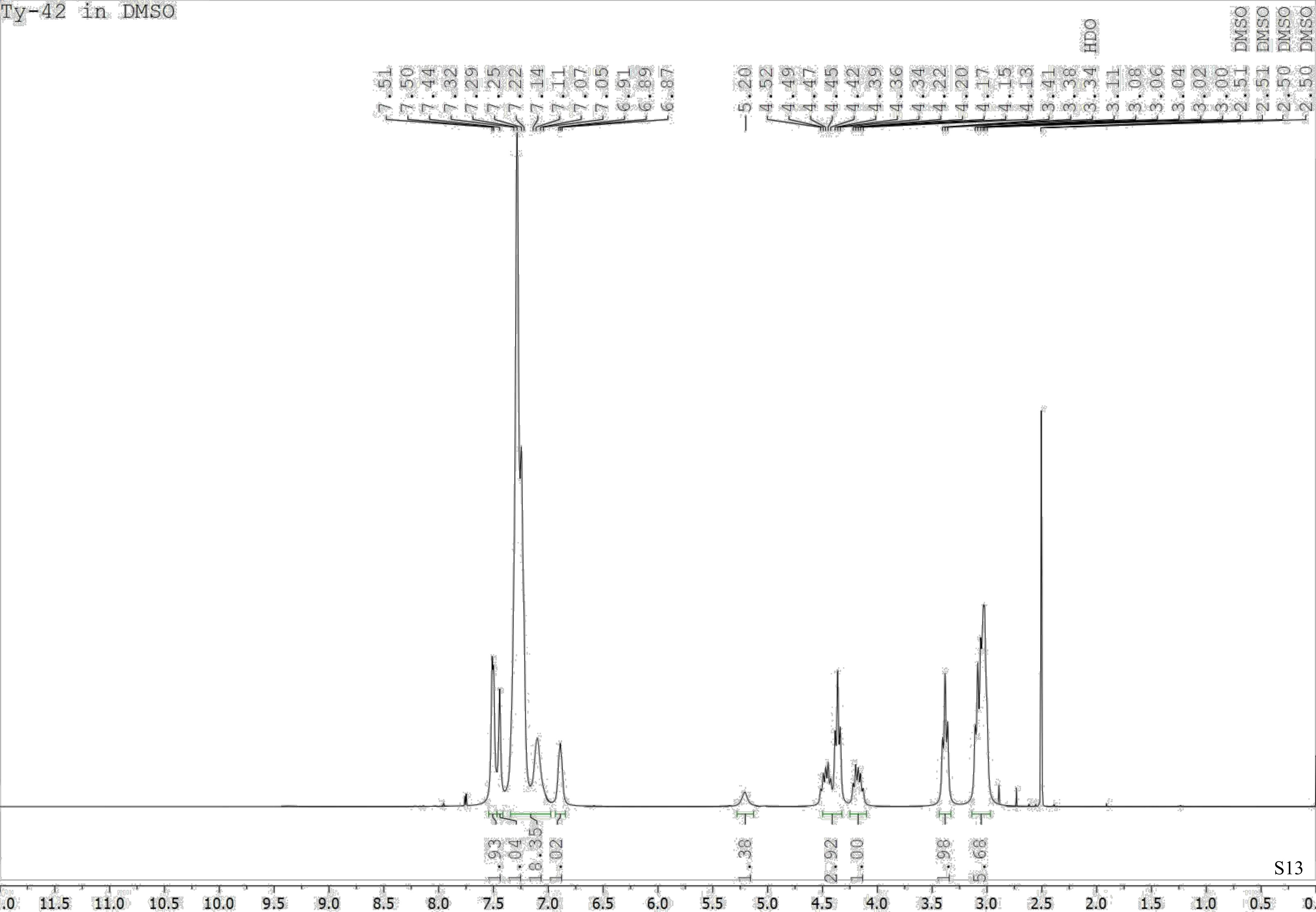

Ty-42 in DMSO
HDO
DMSO
DMSO
DMSO
DMSO
7.51 7.50 7.44 7.32 7.29 7.25 7.22 7.14 7.11 7.07 7.05 6.91 6.89 6.87
5.20
4.52 4.49 4.47 4.45 4.42 4.39 4.36 4.34 4.22 4.20 4.17 4.15 4.13
3.41 3.38 3.34 3.11 3.08 3.06 3.04 3.02 3.00
2.51 2.51 2.50 2.50
1.93 1.04 18.35 1.02 1.38 2.92 1.00 1.98 5.68
11.5 11.0 10.5 10.0 9.5 9.0 8.5 8.0 7.5 7.0 6.5 6.0 5.5 5.0 4.5 4.0 3.5 3.0 2.5 2.0 1.5 1.0 0.5

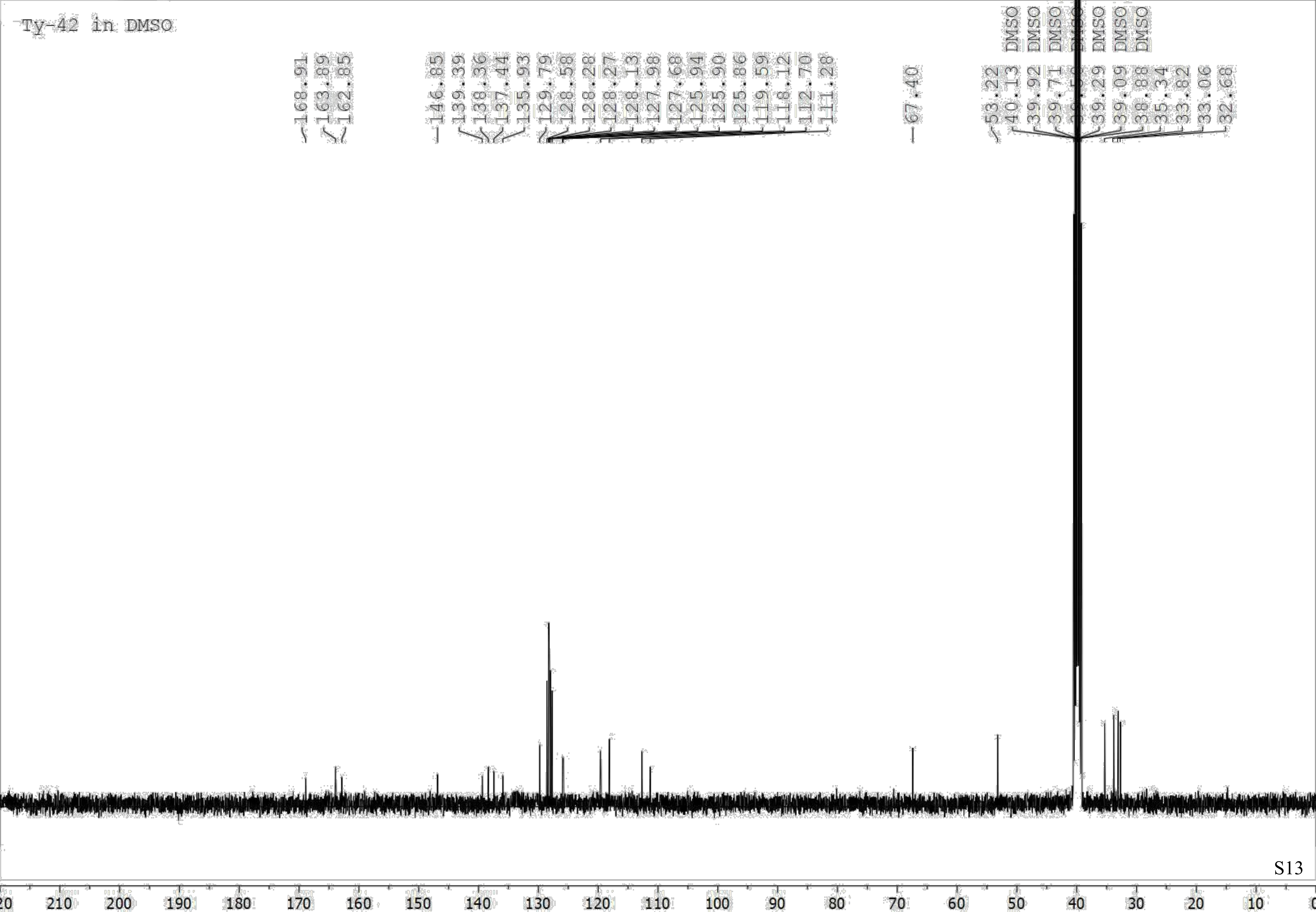

Ty-42 in DMSO
168.91
163.89
162.85
146.85
139.39
138.36
137.44
135.93
129.79
128.58
128.28
128.27
128.13
127.98
127.68
125.94
125.90
125.86
119.59
118.12
112.70
111.28
67.40
53.22
40.13
39.92
39.71
39.29
39.09
38.88
35.34
33.82
33.06
32.68
DMSO
DMSO
DMSO
DMSO
DMSO
DMSO
210
200
190
180
170
160
150
140
130
120
110
100
90
80
70
60
50
40
30
20
10

Ty-43 in DMSO

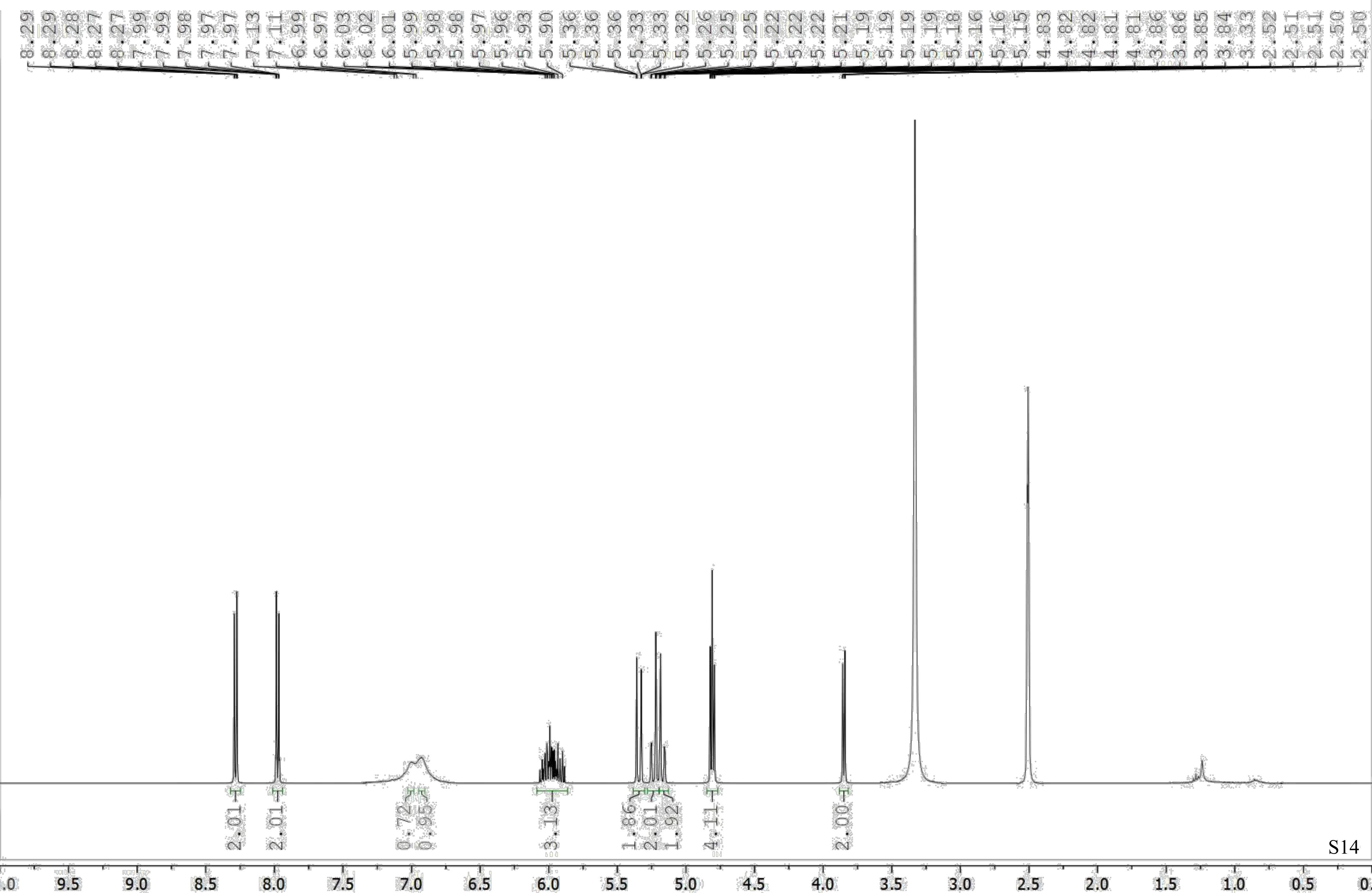
HDO
DMSO
8.29
8.29
8.28
8.27
8.27
7.99
7.99
7.98
7.97
7.97
7.13
7.11
6.99
6.97
6.03
6.02
6.01
5.99
5.98
5.98
5.97
5.96
5.93
5.90
5.36
5.36
5.36
5.33
5.33
5.32
5.26
5.25
5.25
5.22
5.22
5.22
5.21
5.19
5.19
5.19
5.19
5.18
5.16
5.16
5.15
4.83
4.82
4.82
4.81
4.81
3.86
3.86
3.85
3.84
3.33
2.52
2.51
2.51
2.50
2.50
2.01
2.01
0.72
0.95
3.13
1.86
2.01
1.92
4.11
2.00
9.5
9.0
8.5
8.0
7.5
7.0
6.5
6.0
5.5
5.0
4.5
4.0
3.5
3.0
2.5
2.0
1.5
1.0
0.5

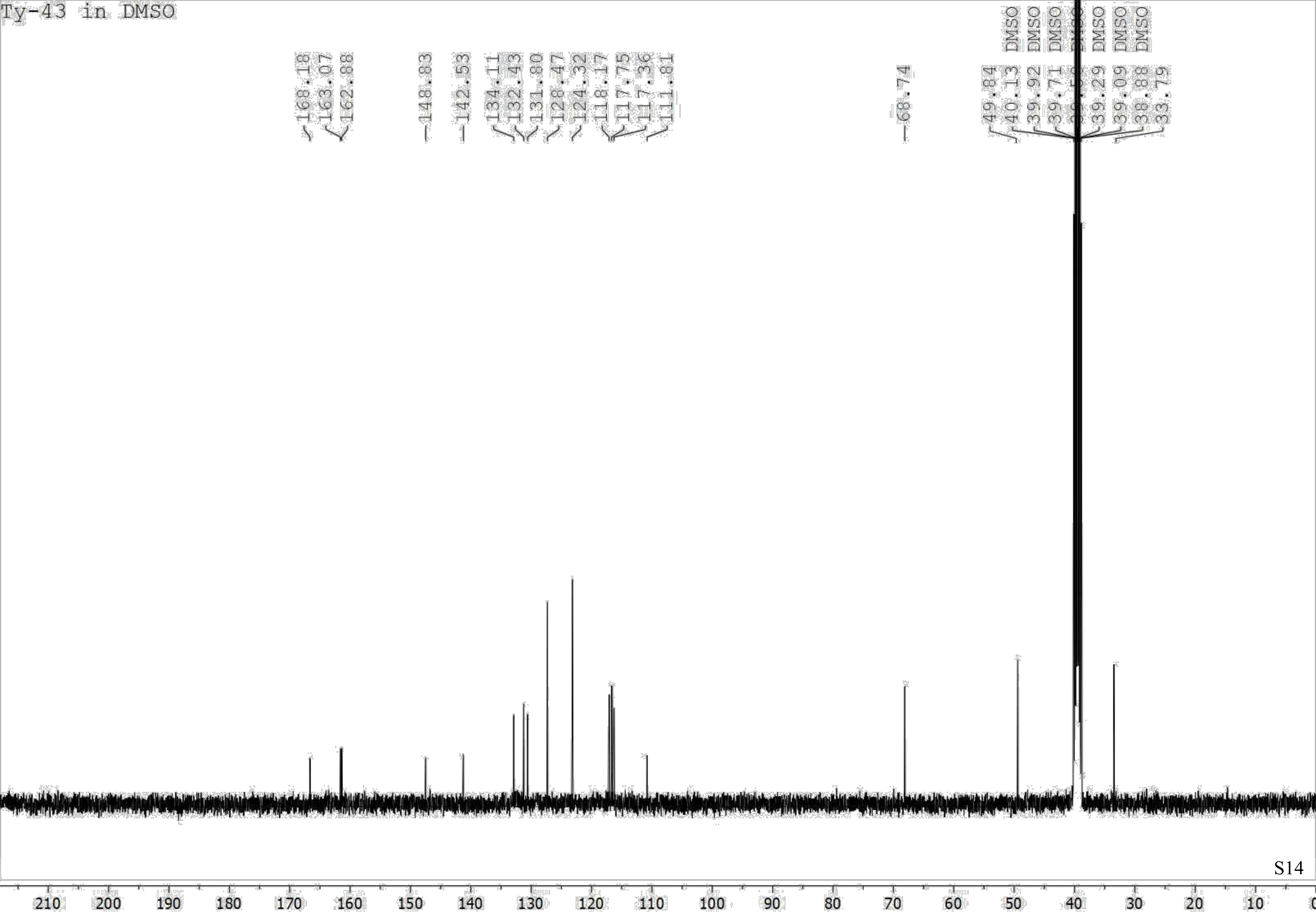

Ty-43 in DMSO
168.18
163.07
162.88
148.83
142.53
134.11
132.43
131.80
128.47
124.32
118.17
117.75
117.36
111.81
68.74
49.84
40.13
39.92
39.71
39.29
39.09
38.88
33.79
DMSO
DMSO
DMSO
DMSO
DMSO
DMSO
210
200
190
180
170
160
150
140
130
120
110
100
90
80
70
60
50
40
30
20
10

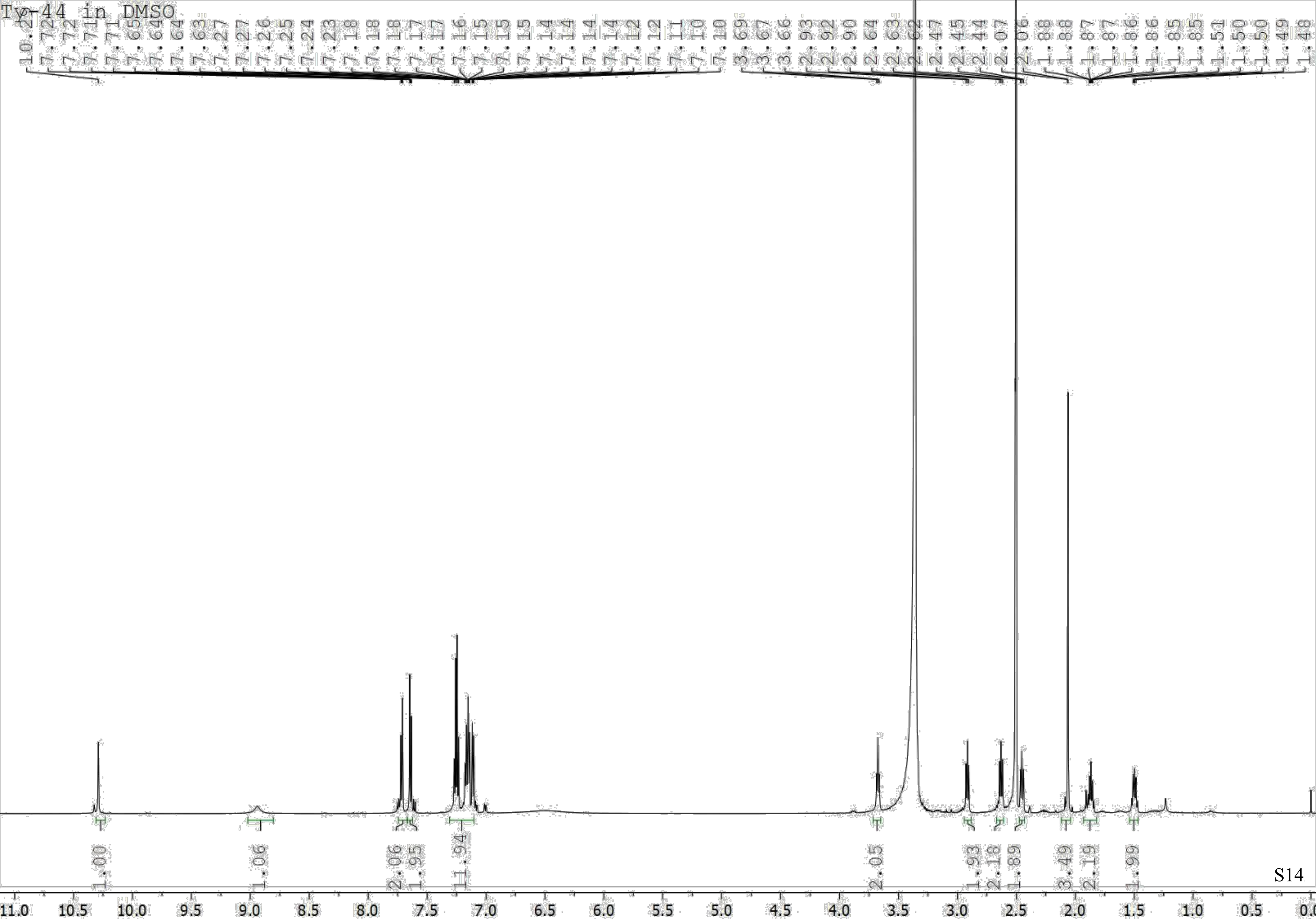
Ty-44 in DMSO
10.27
7.72
7.72
7.71
7.71
7.65
7.64
7.64
7.63
7.27
7.27
7.26
7.25
7.24
7.23
7.18
7.18
7.18
7.17
7.17
7.16
7.15
7.15
7.15
7.14
7.14
7.14
7.14
7.12
7.12
7.11
7.10
7.10
3.69
3.67
3.66
2.93
2.92
2.90
2.64
2.63
2.62
2.47
2.45
2.44
2.07
2.06
1.88
1.88
1.87
1.87
1.86
1.86
1.85
1.85
1.51
1.50
1.50
1.49
1.48
1.00
1.06
2.06
1.95
11.94
2.05
1.93
2.18
1.89
3.49
2.19
1.99
11.0
10.5
10.0
9.5
9.0
8.5
8.0
7.5
7.0
6.5
6.0
5.5
5.0
4.5
4.0
3.5
3.0
2.5
2.0
1.5
1.0
0.5
0.0
